# The Mitogenomes of **Ophiostoma minus** and **Ophiostoma piliferum** and Comparisons With Other Members of the Ophiostomatales

**DOI:** 10.3389/fmicb.2021.618649

**Published:** 2021-02-10

**Authors:** Abdullah Zubaer, Alvan Wai, Nikita Patel, Jordan Perillo, Georg Hausner

**Affiliations:** Department of Microbiology, University of Manitoba, Winnipeg, MB, Canada

**Keywords:** *Ophiostoma*, mobile introns, blue stain fungi, complex introns, homing endonucleases, mitochondria

## Abstract

Fungi assigned to the Ophiostomatales are of economic concern as many are blue-stain fungi and some are plant pathogens. The mitogenomes of two blue-stain fungi, *Ophiostoma minus* and *Ophiostoma piliferum*, were sequenced and compared with currently available mitogenomes for other members of the Ophiostomatales. Species representing various genera within the Ophiostomatales have been examined for gene content, gene order, phylogenetic relationships, and the distribution of mobile elements. Gene synteny is conserved among the Ophiostomatales but some members were missing the *atp9* gene. A genome wide intron landscape has been prepared to demonstrate the distribution of the mobile genetic elements (group I and II introns and homing endonucleases) and to provide insight into the evolutionary dynamics of introns among members of this group of fungi. Examples of complex introns or nested introns composed of two or three intron modules have been observed in some species. The size variation among the mitogenomes (from 23.7 kb to about 150 kb) is mostly due to the presence and absence of introns. Members of the genus *Sporothrix sensu stricto* appear to have the smallest mitogenomes due to loss of introns. The taxonomy of the Ophiostomatales has recently undergone considerable revisions; however, some lineages remain unresolved. The data showed that genera such as *Raffaelea* appear to be polyphyletic and the separation of *Sporothrix sensu stricto* from *Ophiostoma* is justified.

## Introduction

Members of the Ophiostomatales are frequently associates of bark beetles that can serve as vectors for these fungi. Some members are referred to as ambrosia fungi as they exist in symbiotic relationships with wood boring ambrosia species (Vanderpool et al., 2018). Most species of the Ophiostomatales are either non-pathogenic or weak pathogens; some species can kill trees in combination with their beetle vectors or without any contribution by an arthropod vector (Wingfield M.J. et al., 2017). Many members of the Ophiostomatales cause blue-stain of sap wood in hard- and softwood species. Sap-staining fungi are responsible for considerable economic losses in the Forestry sector due to difficulties in exporting stained timbers/lumber products (Uzunovic and Byrne, 2013).

*Ophiostoma minus* is an important agent of blue stain in various pine species (Klepzig, 1998; Gorton and Webber, 2000; Chang et al., 2019) and has been shown to be a potential pathogen of pine (Gorton et al., 2004; Ben Jamaa et al., 2007). *Ophiostoma piliferum* is a serious blue-stain agent on a variety of conifer species but it is not considered to be pathogenic on softwoods (Linnakoski et al., 2012). Both *O. minus* and *O. piliferum* have been reported from many geographic regions and from a variety of hosts and they could represent species complexes (Chakravarty et al., 1994; Gorton and Webber, 2000; Hafez and Hausner, 2011; Jankowiak and Bilański, 2013; Bilto and Hausner, 2016).

Only a few mitochondrial genomes have been characterized so far for members of the Ophiostomatales (Abboud et al., 2018; Zhang et al., 2019). Fungal mitochondrial genomes encode genes involved in translation, such as the small and large ribosomal subunit RNAs (*rns* and *rnl*) and a set of tRNAs, and protein components involved in electron transport chain and oxidative phosphorylation. This includes parts of Complex I (subunits of NADH dehydrogenase: *nad1* to *nad6* and *nad4L*; except for members of the Taphrinomycota and some members of the Saccharomycetales), components of Complex III (*cob*) and Complex IV (*cox1*, *cox2*, and *cox3*), plus members of Complex V (ATP synthase components: *atp6*, *atp8*, and usually *atp9*). Many fungi encode a ribosomal protein (*rps3*) (Hausner, 2003; Freel et al., 2015; Wai et al., 2019) and the RNA (*rnpB* gene) component for RNaseP has also been recorded in some fungal mitochondrial genomes (Lang, 2014). In addition, fungal mitogenomes can encode potential orphan genes (genes with unknown functions and a lack of detectable homologs) and in some members of the Ascomycota mitochondrial open reading frames (ORFs) have been detected that appear to encode putative N-acetyltransferases and amino-transferases (Wai et al., 2019).

Organellar introns in plants and fungi can be self-splicing (ribozymes). However, intron splicing is enhanced by intron- and/or host genome-encoded (nuclear or mitochondrial) factors (Lang et al., 2007; Hausner, 2012; Schmitz-Linneweber et al., 2015). Based on intron RNA folds (secondary structure) and their splicing mechanisms fungal mitochondrial introns can be assigned to either group I or group II introns (Michel and Westhof, 1990; Lambowitz et al., 1999). There are a few instances of complex introns where an intron has inserted into another intron, and these are sometimes referred to as twintrons or nested introns (Hafez and Hausner, 2015; Deng et al., 2016, 2018; Guha et al., 2018; Zumkeller et al., 2020). Nested introns can be composed of group I intron modules or a combination of group I and group II intron modules (Hafez et al., 2013; Guha and Hausner, 2016; Guha et al., 2018). Group I and group II introns can encode intron-encoded proteins (IEPs) that can catalyze the movement of an intron from an intron-containing allele to cognate alleles that do not have introns (Dujon, 1989), a process that is referred to as intron homing or retro-homing, if mediated by reverse transcriptase activity. Group I intron IEPs typically are homing endonucleases (HEs), which are DNA-cutting enzymes that facilitate intron homing or maturases that facilitate intron splicing. There are examples of intron IEPs that have maturase and HE activity (Belfort, 2003; Caprara and Waring, 2005). Two families of HEs, named after the presence of conserved amino-acid motifs, are found in fungal mitochondrial genomes: the LAGLIDADG and the GIY-YIG families of HEs (Stoddard, 2014). HEs can be encoded by independent free-standing genes or their genes (HEGs) are embedded within intronic sequences. It has been reported that HEGs can move independently from their ribozyme partners (Mota and Collins, 1988), although recent studies suggest that intron-encoded HEG co-evolve with their ribozyme partners (Megarioti and Kouvelis, 2020). Finally, there are instances where group II introns encode HEGs; typically group II introns can be ORF-less or encode reverse transcriptases (Toor and Zimmerly, 2002; Mullineux et al., 2010; Hafez and Hausner, 2012; Zimmerly and Semper, 2015).

Herein, we report the mitochondrial genomes for *O. minus* and *O. piliferum*. As more sequences for members of the Ophiostomatales become available mitochondrial DNA could provide a resource for developing markers that allow for distinguishing among various *Ophiostoma* species and allow for resolving some of the taxonomic issues that still need to be addressed with regards to circumscribing species complexes and lineages within *Ophiostoma sensu lato*.

## Materials and Methods

### Fungal Strains, Cultivation, and Preparation of DNA

A strain of *Ophiostoma minus* C262 [= WIN(M)495] [Northern Forest Research Centre, Edmonton, AB, Canada; isolated from *Pinus contorta*; WIN(M) = University of Manitoba]; and a strain of *Ophiostoma piliferum* UAMH 7459 [= NoF1929, = WIN(M)959; isolated near Nelsen, BC, Canada from *Populus tremuloides*; UAMH = UAMH Centre for Global Microfungal Biodiversity, Dalla Lana School of Public Health, University of Toronto] were grown at 20°C on Malt extract agar plates (per 1 L: 20 g Agar, 30 g Malt extract, and 1 g Yeast extract). After 7 days of growth, small agar plugs (20 plugs: ∼ 2 mm × 2 mm) were transferred into 500 ml of YPD broth (per 1 L: 1 g Yeast extract, 1 g Peptone, 3 g Dextrose) medium. The cultures were incubated for 7 days at 20°C and the mycelium was harvested using vacuum filtration. Approximately four grams (wet weight) of mycelium were collected and DNA was extracted and quantified using the DNA extraction protocol described previously in Abboud et al. (2018).

### Sequencing and Assembly of Mitochondrial Genomes

One hundred ng of DNA in 75 μl of H_2_O was supplied to McGill University and Génome Québec Innovation Centre (McGill University, QC, Canada) for shotgun Illumina sequencing using the MiSeq platform. The DNA preparations were part of a set of 20 DNA samples that were individually barcoded and thereafter pooled for sequencing (Abboud et al., 2018; Zubaer et al., 2018). Average size reads were 250 nt and average quality was 35 and quality offset was 33. The paired-end reads were trimmed to remove the barcodes/adaptor sequences and assembled *de novo* into contigs and scaffolds with the A5-miseq pipeline [(Coil et al., 2015); McGill University and Génome Québec Innovation Centre; Canadian Center for Computational Genomics (C3G)]. For *O. minus*, a scaffold of 91,847 nt and for *O. piliferum* a scaffold of 69,966 nt could be recovered. These scaffolds, based on BLAST searches against the NCBI non-redundant database, contained mitochondrial sequences that showed matches with mitochondrial gene sequences previously deposited for *O. novo-ulmi* subsp. *novo-ulmi* (GenBank accession number: MG020143.1).

### Annotation of Mitochondrial Genomes

The mitochondrial DNA sequences were annotated with the aid of the following programs: MFannot and RNAweasel (Gautheret and Lambert, 2001; Lang et al., 2007). The MFannot program (setting genetic code 4; the mold, protozoan, and coelenterate mitochondrial code) predicts protein-encoding genes, ribosomal RNAs (rRNAs), transfer RNAs (tRNAs), potential intron/exon junctions, and intron types. In addition, the online tRNAscan-SE2 (Chan and Lowe, 2019) program was applied to the data set to verify the prediction of tRNA genes. For a precise annotation, gene sequences were individually verified starting with aligning gene sequences to similar sequences (acquired via BLASTn) in closely related fungi using the MAFFT (Katoh and Standley, 2013) and AliView (Larsson, 2014) programs. Intron/exon boundaries were predicted based on alignments with intron-less versions of cognate alleles from other fungal species. The RNAweasel program^1^ was used to identify tRNAs, rRNAs, and intron types. All ORFs, introns, and intergenic spacers were identified using ORF Finder^2^ and nucleotide 6-frame translation-protein BLAST (BLASTx) searches against NCBI databases^3^. Feature tables were generated and, along with the mitochondrial genome sequences, these were analyzed further with Artemis (Rutherford et al., 2000) to refine the annotation (GenBank accession numbers MW122509.1 and MW122508.1 for *O. minus* and *O. piliferum*, respectively). The output from Artemis was adjusted and applied to Circos program (Krzywinski et al., 2009) for visualization of the annotated mitogenomes showing the genes, tRNA, introns, and a feature-wise GC plot (calculating GC for every genetic feature such as exon, intron, intergenic region, etc.).

Intron nomenclature for *rns* and *rnl* introns was based on Johansen and Haugen (2001); for mtDNA protein coding sequence (CDS) we used the *Saccharomyces cerevisiae* CDS sequences to map introns (Guha et al., 2018), and for the *nad* genes we applied the *nad* sequences from *Neurospora crassa* (Zubaer et al., 2019).

### Comparative Mitogenomic Analysis

The *O. minus* and *O. piliferum* mitogenomes were compared with other available sequences for members of the Ophiostomatales. In some instances, sequences were available in NCBI as whole genome sequence data sets; see Supplementary Table 1 for accession numbers (Van der Nest et al., 2014; Wingfield et al., 2015a,b; [Bibr B104][Bibr B106]; Vanderpool et al., 2018). The whole genome assemblies were searched for mitochondrial sequences with BLASTn using the mitogenome of *O. novo-ulmi* subsp. *novo-ulmi* (Abboud et al., 2018) as the query. The recovered scaffolds were examined, annotated, and validated as described above utilizing MFannot, RNAweasel, and MAFFT (assemblies and annotations are presented in Supplementary Data File 1). A panintronic landscape was visualized with the aid of the Circos program.

### RNA Folding of Selected Complex Introns

Group I and II intron classifications and their secondary core elements/folds were predicted by the RNAweasel program (Lang et al., 2007). For group I introns the P1, P2, P5, P6, P7.1 (and stem), and P9 helices were predicted by Mfold (Zuker, 2003) and these helices were supported by comparative sequence analysis (including multiple sequence alignments) with related intron sequences. Group II introns were identified based on the highly conserved domain V sequence and the group II intron RNAs were drawn with the aid of Mfold to optimize the expected secondary and tertiary interactions known to stabilize group II introns RNAs in a splicing competent fold (Toor et al., 2001; Michel et al., 2009; Marcia et al., 2013). Secondary structure of introns and their features were based on existing models by Michel and Westhof (1990); Jaeger et al. (1991), Deng et al. (2016), and Guha et al.(2018; and citations herein). Sequence alignments with related introns and their flanking boundaries sequences confirmed the intron boundaries and classifications. The final introns folds were drawn using CorelDRAW Graphics Suite X6 (Corel Corporation, Ottawa, ON, Canada).

### Phylogenetic Analysis

A phylogenetic tree was generated to infer the phylogenetic position of *O. minus* and *O. piliferum* among other members of the Ophiostomatales. Twenty-three mitogenomes were available for the Ophiostomatales from the NCBI genome and GenBank databases. In addition, sequences utilized in Abboud et al. (2018) and Zubaer et al. (2018) were also included to evaluate the phylogenetic distribution of members the Ophiostomatales. The analysis was based on concatenated amino-acid sequences of 13 mitochondrial proteins encoded by the following genes: *atp6*, *8*, *cob*, *cox1*–*3*, *nad1*–*6*, and *nad4L*. The scaffold representing the mitogenome of *Ophiostoma ips* (NTMB01000349.1; scaffold_143) appears to be missing a small segment that includes *atp8*, therefore a separate analysis based on 12 concatenated protein sequences was conducted to confirm the position of *O. ips*. Forty-eight mitogenomes represented by concatenated amino-acid sequences were aligned with the MAFFT program using its iterative refinement method (FFT-NS-i). The aligned dataset was used for tree construction with the MrBayes program (Ronquist et al., 2012) applying the mixed model setting, which finally determined and used the best fit model as cpREV (Adachi et al., 2000). The analysis was performed by running 1 million generations with sample frequency set at 1000. For the sampled trees, the burn-in value was 25% to construct the majority rule consensus tree and for assessing posterior probability values. Two Eurotiales mitogenomes (*Aspergillus fumigatus* and *Penicillium digitatum*) were used as outgroup of the tree and re-rooted accordingly.

## Results

### Organization and Features of the Mitochondrial Genomes

**FIGURE 1:**
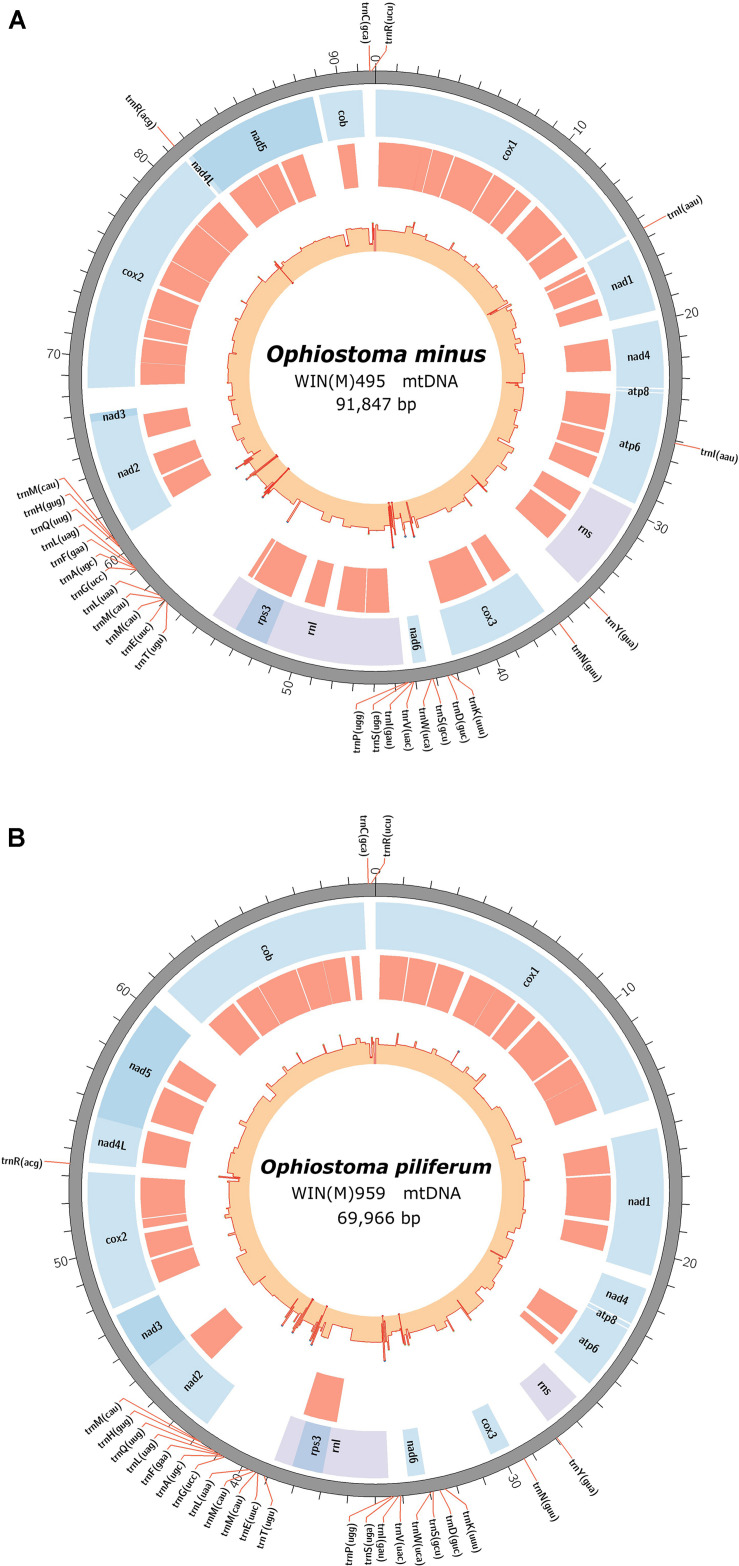
Circular representation of the mitochondrial genomes showing the tRNA (pointed outward on the scale), protein coding genes (blue track), introns (red track), and genetic-feature-wise GC graph (GC% was calculated for annotated features instead of fixed window, showed in the innermost track). **(A)** The annotated mitochondrial genome of *Ophiostoma minus*; total size of the circular genome is 91,847 bp (GenBank accession: MW122509.1). **(B)** The annotated mitochondrial genome of *Ophiostoma piliferum*; total size of the circular genome is 69,966 bp (GenBank accession: MW122508.1).

The newly obtained mitogenomes of *O. minus* and *O. piliferum* can be represented as circular molecules of 91,847 and 69,966 nt, respectively (Figures 1A,B). The genomes encode the following 14 protein coding genes: *atp6*, *atp8*, *cob*, *cox1*–*cox3*, *nad1*–*nad6*, *nad4L*, and *rps3*. In addition, the genomes encode the following RNA structural genes: 27 tRNAs in *O. minus* and 25 tRNAs in *O. piliferum*, plus the small and large ribosomal RNA subunit genes (*rns* and *rnl*, respectively). The ribosomal protein RPS3 is encoded by a group IA type intron inserted within the *rnl* gene. Other features noted are the fusion of the *nad2* and *nad3* genes and the overlap between the *nad4L* and *nad5* ORFs by one nucleotide, i.e., the last nucleotide of the *nad4L* stop codon serves as the first nucleotide of the *nad5* ORF. These gene arrangements have been previously observed in other members of the Ophiostomatales and Ascomycota (Aguileta et al., 2014; Abboud et al., 2018). The mitogenomes of *O. minus* and *O. piliferum* do not appear to encode the *atp9* and *rnpB* genes. For both mitogenomes all genes are encoded by the same strand and the gene order is as follows: *cox1*, *nad1*, *nad4*, *atp8*, *atp6*, *rns*, *cox3*, *nad6, rnl* (including *rps3*), *nad2*, *nad3*, *cox2*, *nad4L*, *nad5*, and *cob*. Gene order is conserved across the 25 examined members of the Ophiostomatales and in 22 species we noted the presence of the *atp9* gene, located between *nad3* and *cox2*.

**FIGURE 2:**
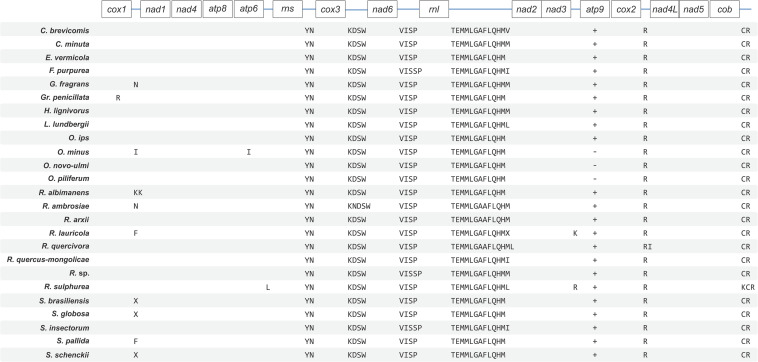
Schematic representation of gene order and position of tRNA gene clusters for members of the Ophiostomatales. Gene order is conserved across the 25 sampled Ophiostomatales with minor variations in tRNA composition and the presence or absence of the *atp9* gene. Genes encoding for tRNAs is represented using their respective single-letter amino acid codes. Intron-encoded tRNAs are represented by placing them under the gene that encodes them. Plus (+) and minus (–) signs represent presence and absence of a gene, respectively, and only applies to the *atp9* gene. See Supplementary Table 1 for GenBank NCBI accession numbers.

The synteny with regards to the tRNA genes are conserved among the examined members of the Ophiostomatales (see Figure 2). Most tRNAs are arranged in clusters located between the *cox3* and *nad6* genes (a cluster of 4–5 tRNA genes), the *nad6* and *rnl* genes (a cluster of 4–5 tRNA genes), and the largest grouping of tRNA genes was detected between the *rnl* and *nad2* genes (a cluster of 12–14 tRNA genes). In *O. minus*, one putative tRNA gene appears to be encoded within an intron in the *atp6* gene (*atp6*-i1 or *atp6*–173). Between the *cox2* and *nad4L* gene, all examined members of this order contain the tRNA gene for Arg (R); between the *rns* and *cox3* gene, the tRNA genes for Tyr (Y) and Asn (N), and the tRNA genes for Cys (C) and Arg (R) are positioned between the *cob* and *cox1* gene. The intergenic region separating the *cox1* and *nad1* genes appears to be quite diverse with regards to tRNA genes with 16 members showing no indication for the presence of tRNA genes and others showing the presence of tRNA genes for Asn (N), Arg (R) (intron-encoded, *cox1*–1281), Ile (I), Lys (K), Asn (N), Phe (F), or X (a highly derived tRNA gene predicted to bind to phenylalanine; Lang et al., 2012).

### Ophiostomatales and Their Mitogenome Intron Complement

For the examined members of the Ophiostomatales the mitogenomes range in size from 23,830 bp (*Raffaelea* sp. RL272) to >150 kb (*Raffaelea quercivora*). The smallest mitogenomes belong to members of *Sporothrix sensu stricto* (*Sporothrix schenckii*: 26,095 bp; *Sporothrix globosa*: 26,671 bp), and the available sequences for *Graphilbum fragrans*: 25,567 bp and *Hawksworthiomyces lignivorus*: 27,092 bp. These smaller genomes are devoid of introns except for the RPS3 encoding group IA intron located in the *rnl* gene. In addition, the mitogenomes of *H. lignivorus* and *G. fragrans* contain one additional intron in the *cox1* gene.

**FIGURE 3:**
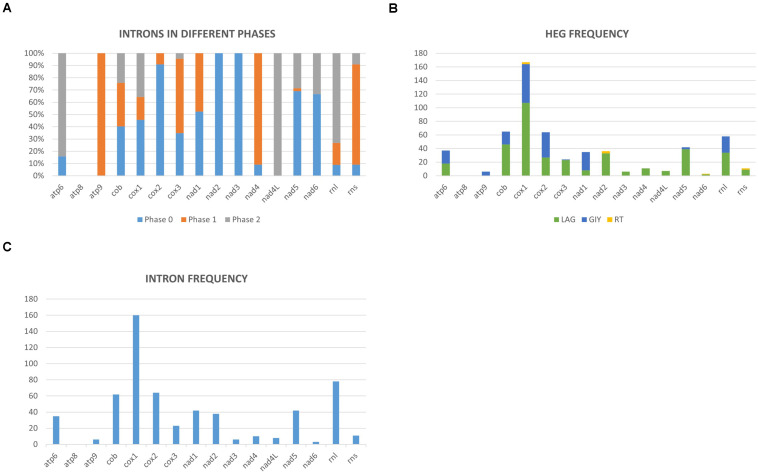
**(A)** Mitochondrial introns of the Ophiostomatales categorized according to intron phasing on a gene by gene basis. **(B)** Representation of the relative frequencies of different types of introns encoded open reading frames based on gene by gene basis (LAG, LAGLIDADG type homing endonucleases/maturases; GIY, GIY-YIG type homing endonucleases; RT, reverse transcriptases). **(C)** Relative distribution and number of mitochondrial introns recorded on a gene-by-gene basis among the examined members of the Ophiostomatales.

Mitochondrial intron numbers range from 1 to 64 introns per genome across the examined Ophiostomatales mitogenomes (Supplementary Table 1 and Figure 3). Combined, 594 putative introns (complex/nested introns were treated as one item) were recorded based on structural features, 573 could be assigned to be group I introns, 15 introns are group II type introns, and six introns could not be assigned to any category. A total of 118 intron insertion sites were identified across the various mtDNA genes. Among these, 94 insertion sites were noted to be in protein coding genes with 55 sites in phase 0 (intron does not disrupt a codon), 19 in phase 1 (intron position after the first nucleotide of the codon), and 20 sites occupied a phase 2 position (intron insertion after the second nucleotide in the codon) (Figure 3A). With regards to the observed 594 introns, 505 were inserted in protein coding genes and among those 270 where in phase 0, 109 in phase 1, and 126 introns in phase 2 (Figure 3A). The rRNA genes had 24 intron insertion sites (18 within the *rnl* gene and six within the *rns* gene); these sites accounted for 89 introns. Twenty intron insertion sites among the 118 intron insertion sites had 10 or more introns present accounting for 246 introns.

Group I introns were either ORF-less or encoded LAGLIDADG or GIY-YIG type ORFs. Among the 15 group II introns, three were ORF-less, 9 encoded reverse transcriptase-like ORFs, and three encoded LAGLIDADG type ORFs (see Supplementary Table 2 and Figure 3B). Among the Ophiostomatales, the mS722 and mL952 group II introns encoded LAGLIDADG type ORFs. The mL2450 group I A intron encodes the RPS3 protein and in a few instances the rps3-coding sequence was fused in-frame to a LAGLIDADG HE-coding sequence (Gibb and Hausner, 2005).

**FIGURE 4:**
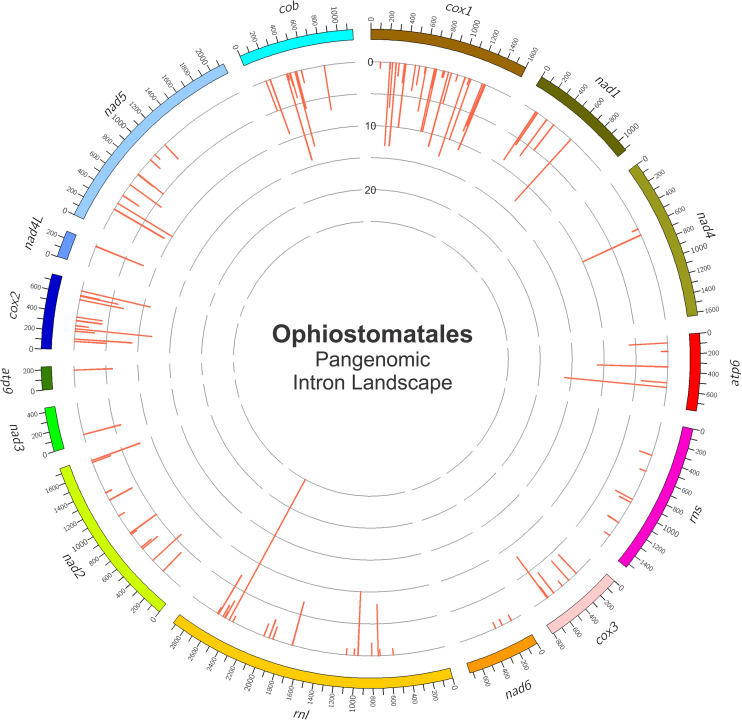
The panintronic landscape for the studied members of the Ophiostomatales. The landscape was generated by Circos and shows all intron insertions sites and their frequencies. More detailed intron landscapes showing intron types, intron-encoded protein types, and introns phasing are shown in Supplementary Figures 1A–D.

Among the 25 mitogenomes examined, the intron-rich genes were as follows (with total intron/insertion numbers listed in brackets): *cox1* (161), *rnl* (78), *cox2* (65), *cob* (62), *nad1* (42), *nad5* (42), *nad2* (38), and *atp6* (38). The *cox1* gene was observed to have the most intron insertion sites at 29 (one element at *cox1*–264 could not be classified) with the *rnl* gene having 18 insertion sites (Figure 3C). The panintronic landscape for the studied members of the Ophiostomatales is illustrated in Figure 4 and more detailed intron landscapes showing intron types, IEPs, and introns phasing are shown in Supplementary Figures 1A–D.

### Phylogenetic Groupings Observed With Mitogenome Analysis

**FIGURE 5:**
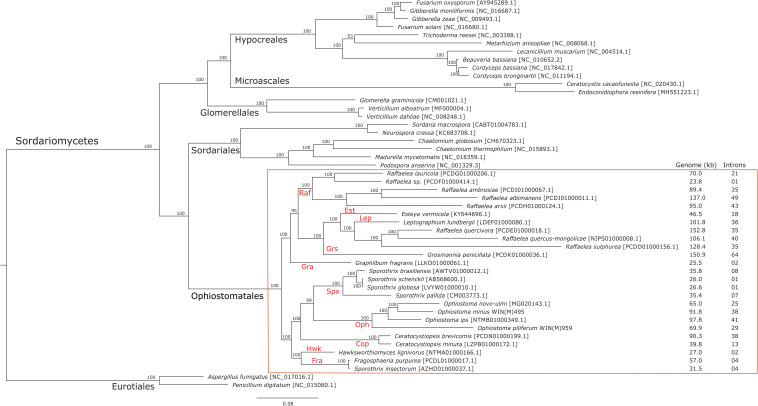
Phylogenetic tree of mitogenomes showing the position of *Ophiostoma minus and Ophiostoma piliferum* among members of the Ophiostomatales. The tree topology (50% majority-rule consensus tree) was generated by the MrBayes program and involved 48 concatenated amino acid sequences. The tree is drawn to scale with branch length measured in the number of substitutions per site. Posterior probability values are indicated at the nodes. NCBI and GenBank accession numbers (except for *Verticillium alboatrum*, which refers to MitoFun database) are indicated in square brackets. For the members of the Ophiostomatales, mitogenome sizes, and total numbers of introns are listed for each genome. Taxonomic designations (Orders, and Genera for the Ophiostomatales) are indicated on the relevant branches. Raf, *Raffaelea*; Gra, *Graphilbum*; Est, *Esteya*; Grs, *Grosmannia*; Lep, *Leptographium*; Spx, *Sporothrix sensu stricto*; Cop, *Ceratocystiopsis*; Oph, *Ophiostoma sensu stricto*; Hwk, *Hawksworthiomyces*; Fra, *Fragosphaeria*.

The phylogenetic analysis of 48 concatenated mitochondrial protein sequences including 25 species that belong to the Ophiostomatales yielded a topology showing the following monophyletic groupings: Microascales, Hypocreales, Glomerellales, Sordariales, and Ophiostomatales (Figure 5). Within the Ophiostomatales, several lineages could be identified representing the following genera: *Ceratocystiopsis*, *Graphilbum, Hawksworthiomyces*, *Raffaelea sensu stricto*, *Ophiostoma sensu stricto*, and *Sporothrix* (Figure 5). However, the mitochondrial sequences failed to show monophyly for species assigned to *Sporothrix* and *Raffaelea*.

### Phylogeny vs. Mitogenome Size and Intron Numbers

**FIGURE 6:**
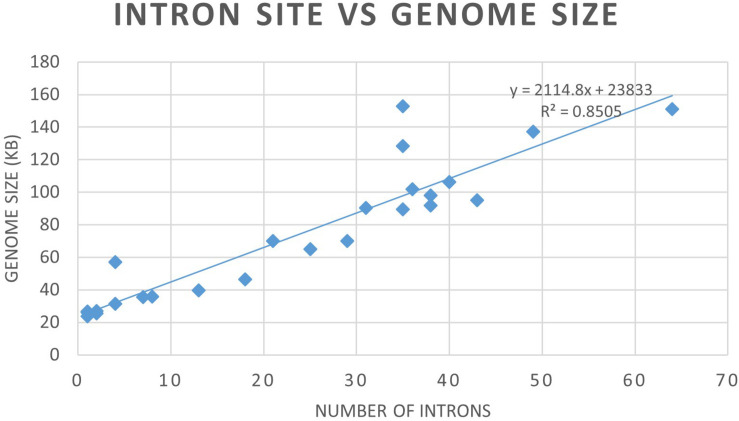
Graph depicting the relationship between mitogenome sizes and intron numbers per mitogenome for the examined members of the Ophiostomatales.

Mitogenome sizes and intron content are quite variable among the examined members of the Ophiostomatales and do not necessarily correspond do the phylogenetic position of the species examined. Genome sizes do correspond to the number of introns they contain with smaller genomes containing few introns and larger mitogenomes being intron-rich (Figures 5, 6). Members of the genus *Sporothrix sensu stricto* appear to have smaller mitogenomes ranging from 26.1 to 35.9 kb, whereas members of *Raffaelea sensu stricto* have mitogenomes ranging from 23.8 to 137 kb. Mitogenome sizes for the clade that includes *Esteya*, *Leptographium*, members of *Raffaelea sensu lato*, and *Grosmannia* range from 46.5 to >150 kb. This clade also includes the two largest mitogenomes, *Grosmannia penicillata* and *Raffaelea quercivora*, both >150 kb. Members of *Ophiostoma sensu stricto*, which include *O. minus* and *O. piliferum*, range from 65 to 97.8 kb. Only two members of *Ceratocystiopsis* were available and their mitogenome sizes range from 39.8 to 90.3 kb, but these two examples demonstrate that sharing a recent common ancestor does not imply similar genome sizes or intron content. Intron numbers can be quite variable between or within the various clades that comprise the Ophiostomatales (Figure 5), with the possible exception for species belonging to *Sporothrix sensu stricto*. Plotting the intron number against the genome size for each genome shows a linear relationship with a strong (86%) correlation between intron numbers and genome sizes (Figure 6). Gene synteny and gene content is conserved among the Ophiostomatales, so intron content is a significant factor with regard to mitogenome size and variability (Figures 5, 6).

### Complex and Novel Introns

**FIGURE 7:**
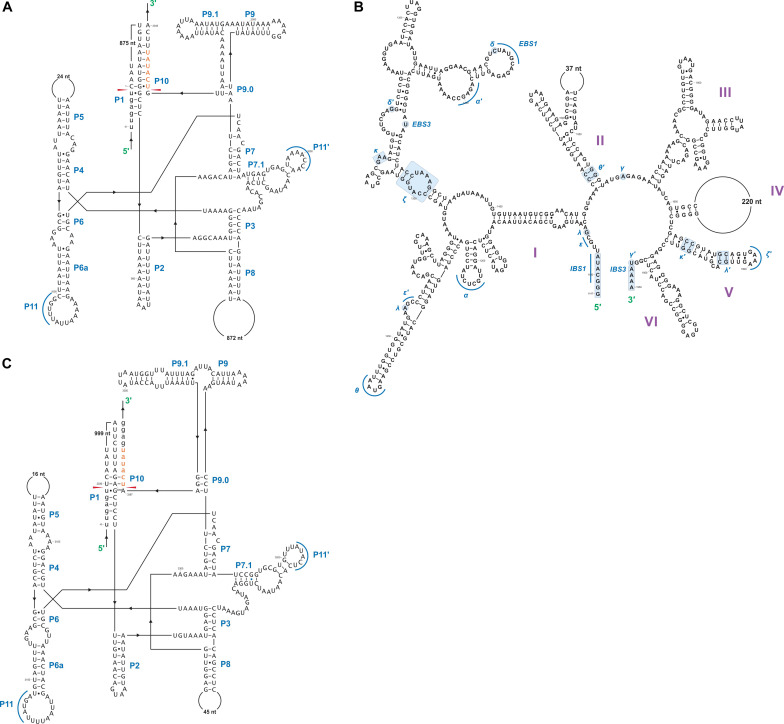
Predicted *cob* I4 (*cob*-490) RNA fold composed of three intron modules **(A–C)**. Pairing regions [for group I introns: P1–P11; and domains (D) I–VI for the group II intron] are labeled by purple text; tertiary interactions are shown by blue lines. Exon and intron sequences are represented by lowercase and uppercase letters, respectively. Red arrows indicate intron-exon/pseudoexon boundaries. Orange subsequence in uppercase letters is exon-mimicking (pseudoexon) sequence, which is annotated as within *cob* I4’s downstream group IA1 intron component (*cob* I4-C). Orange subsequence in lowercase letters is annotated as downstream exon sequence (*cob*-EB). **(A)** *cob* I4’s upstream group IA1 intron (*cob* I4-A) RNA secondary structure model. **(B)** *cob* I4-B RNA secondary structure model. IBS, intron binding sequence; EBS, exon binding sequence. Helical domains I–VI branching from a central linker sequence (“six fingered hand”) shown. Potential tertiary interactions (Greek letters) are indicated. **(C)** *cob* I4’s downstream group IA1 intron (cob I4-C) RNA secondary structure model.

**FIGURE 8:**
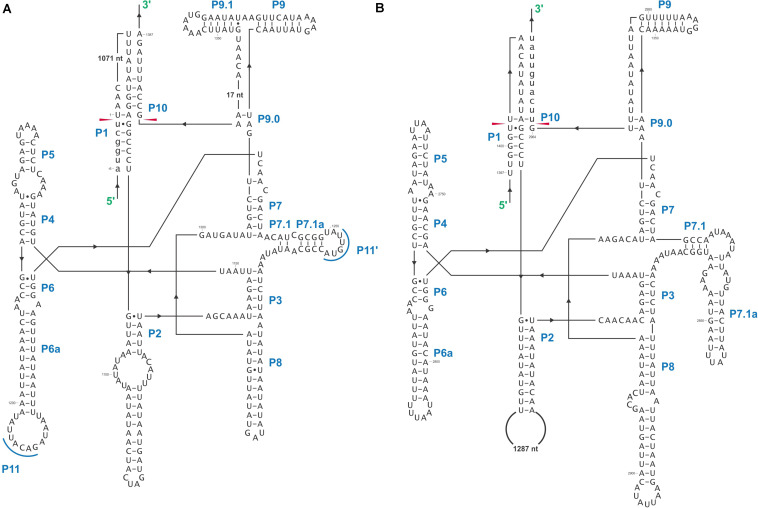
Predicted *cox3* I2 (*cox3*–640) RNA secondary structure model composed of two intron modules. Pairing regions (P1–P11) are labeled by blue text; tertiary interactions are shown by blue lines. Exon and intron sequences are represented by lowercase and uppercase letters, respectively. Red arrows indicate intron-exon/pseudoexon boundaries. **(A)** *cox3* I2-A RNA secondary structure model. **(B)** *cox3* I2-B RNA secondary structure model.

In this study we found five potential novel intron arrangements that have not yet been reported for members of the Ophiostomatales (Gibb and Hausner, 2005; Sethuraman et al., 2009; Mullineux et al., 2010; Rudski and Hausner, 2012; Hafez et al., 2013). In *O. minus*, the first intron in the *atp6* gene (*atp6* i1 at position 81) encodes a double-motif LAGLIDADG ORF followed by a tRNA for I and this intron contains a segment at its 5′ end that is a partial duplication of the downstream exon. The *cox1*–281 intron in *Leptographium lundbergii* (LDEF01000080.1) and in *Raffaelea albimanens* (PCDJ01000011.1) is composed of a group IB type intron that encodes a double-motif LAGLIDADG is interrupted by a group IC2 intron module encoding a double-motif LAGLIDADG. The *cox2*–657 intron in *Raffaelea quercus-mongolicae* (NIPS01000008.1) appears to be composed of two group IC1 intron modules. Based on comparative analysis the original “resident” IC1 intron encodes a GIY-YIG type ORF in the P9 loop and near the N-terminal coding region of this ORF an IC1 intron has been inserted. More complex or nested introns were observed in the *cob* (cytochrome b; *cob* i4) and *cox3* (cytochrome 3; *cox*3 i2) genes in *Ophiostoma ips* (NTMB01000349.1) and these are described below in more detail. Plausible RNA folds for the *O. ips* complex introns are presented in Figures 7, 8.

**FIGURE 9:**
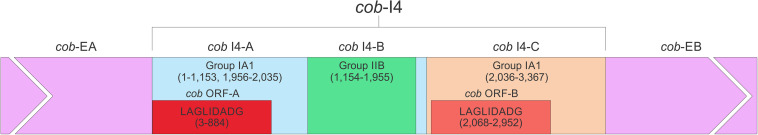
*cob-* 490 intron schematic diagram. *cob* i4, the entire complex intron at *cob* 490 position. *cob* i4-A, cob i4’s upstream group IA intron; *cob*-EA, upstream exon; *cob* ORF-A, *cob* i4-A’s ORF; *cob* i4-B, *cob*i4’s middle group IIB intron; *cob* i4-C, *cob* i4’s downstream group IA intron; *cob* ORF B, *cob* i4-C’s ORF; *cob*-EB, downstream exon. LAGLIDADG represents type of homing endonuclease ORF encoded by group I intron. The numbers in brackets represent the position and length of each intron element relative to the start of *cob* i4. As the two group I introns are of the same subtype, their interactions can be interchangeable; components of the internal members can interact with components of the external member to form paired regions.

**FIGURE 10:**
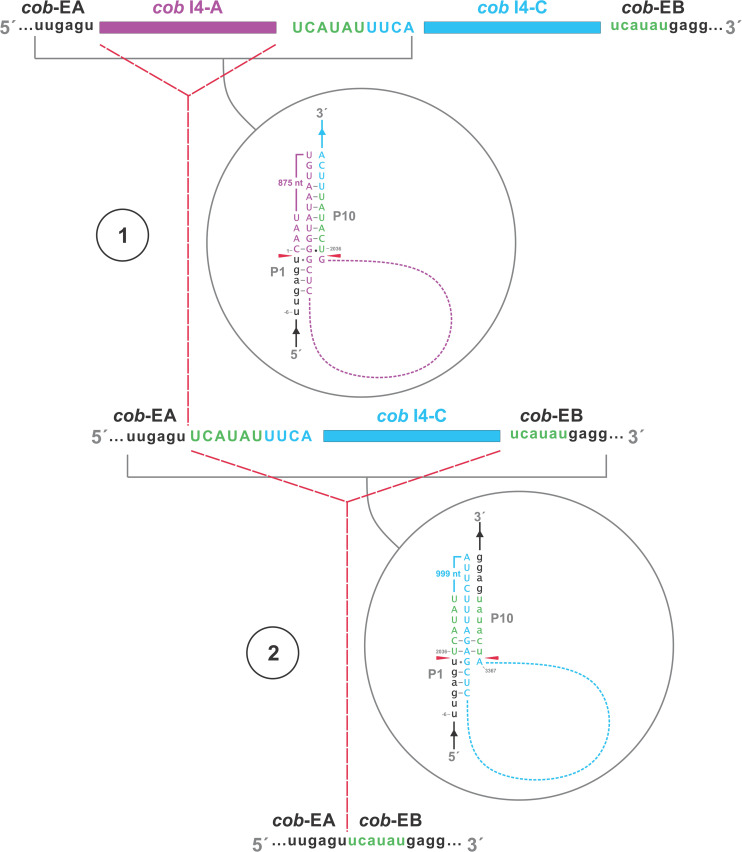
Proposed RNA “ratchet-like” splicing model for *cob* I4 (*cob*-490). As the group I intron modules are both 1A types they have similar sequence elements that allow for the formation of the helical regions between the two modules (P1–P9), thus functionally they can act like tandem introns, i.e., side-by-side introns. Splicing occurs via a two-step process: (1) The “upstream” intron initially splices out using a sequence, referred to as a potential pseudoexon, located between the “upstream” and “downstream” introns, and identical to the first six nucleotides of the downstream exon. (2) Subsequent splicing of “downstream” intron results in joining of the upstream and downstream exons. *cob*-EA and *cob*-EB refer to upstream and downstream exons, respectively. The pseudoexon and downstream exon are represented in green in uppercase and lowercase, respectively. *cob* I4-A (purple) and *cob* I4-C (blue) refer to the “upstream” and “downstream” introns, respectively. Proposed P1 and P10 interactions are shown in gray circles.

A schematic overview of the *O. ips cob* i4 intron inserted at position 490 (relative to the *S. cerevisiae cob* coding sequence; GenBank accession number: KP263414.1) is shown in Figure 9. This complex intron consists of three distinct modules that contain all the necessary components for splicing. The three modules are a group I intron that is interrupted by a group II intron module and this composite element is inserted within the P1 loop of a group I intron module (presumable the resident intron). The group II intron appears to be ORF-less and is located within the P8 loop of the host group I intron module. The group I intron components contain ORFs that encode double motif LAGLIDADG type homing endonucleases. There is a short sequence separating the two group I intron modules. This so called “inter-intron module sequence” could be used as a “pseudoexon” by the internal group I intron component for the formation of the P10 helix or for the resident intron module for its P1 formation (Figures 9, 10). “Pseudoexon” is a term to describe intronic sequences that might be utilized during splicing by serving as “temporary exon” sequences; ultimately “pseudoexon” sequence are assumed to be removed when all intron components have been spliced out. The P1 and P10 helices are essential in aligning sequences that are to be spliced out or spliced together. The resident intron module can also form a P10 interaction with the downstream exon; this would allow the entire complex intron to splice out as one unit. The two group I intron modules belong to the same subtype (IA) and therefore the possibility exists that at the RNA level the two intron module components (P1–P9) can interact with each other in various combinations that may allow for various splicing pathways.

**FIGURE 11:**
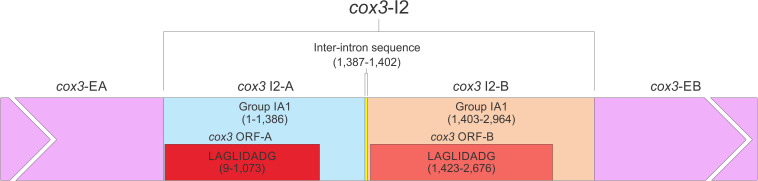
The *cox3*–640 intron schematic diagram. *cox3* i2, the entire complex intron at *cox3–*640 position. *cox3* i2-A, *cox3* i2’s upstream group IA intron; *cox3*-EA, upstream exon; *cox3* ORF-A, *cox3* i2-A’s ORF; *cox3* i2-B, *cox3* i2’s downstream group IA intron; *cox3* ORF-B, *cox3* i2-B’s ORF; *cox3*-EB, downstream exon. Inter-intron sequence: sequence separating *cox3* i2-A and *cox3* i2-B. LAGLIDADG designation represents the type of homing endonuclease ORF encoded by group I introns. The numbers in brackets represent the position and length of each intron element relative to the start of *cox3* i2.

The *O. ips cox3* i2 was confirmed as being *cox3*–640 relative to the *S. cerevisiae cox3* sequence (GenBank accession number: KP263414.1) with the total length of the intron being 2964 nt. The *cox3* i2 based on MFannot and RNAweasel analysis combined with BLASTn analysis was noted to be composed of two group I intron modules in a tandem arrangement (Deng et al., 2016): an upstream group IA1 intron module (*cox3* i2-A) corresponding to *cox3* i2 nucleotides 1–1386, and a downstream group IA1 intron module (*cox3* I2-B) corresponding to *cox3* i2 nucleotides 1403–2964 (see *cox*3 intron schematic) (see Figure 11). This complex intron also appeared to contain a sequence separating *cox3* i2-A and *cox3* i2-B intron modules, referred to as the inter-intron module sequence. The inter-intron module sequence was annotated as corresponding to *cox3* i2 nucleotides 1387–1402, and BLASTn did not show any related or similar sequences in GenBank. It does not appear to be part of either intron module; in addition, careful examination failed to reveal any sequences that could be utilized to form a suitable P10 interaction for the upstream intron module. That implies that the upstream intron module utilizes the downstream exon for the formation of the P10 interaction, which results in the splicing of the entire composite intron element. Similar tandem intron arrangements for the *cox3*–640 intron were observed in *Ceratocystiopsis brevicomis* (PCDN01000199.1) and *Grosmannia penicillata* (PCDK01000036.1).

## Discussion

### Mitogenomes of the Ophiostomatales

Fungal mitogenomes are usually represented as circular molecules and reported to range in size from 12.055 to >500 kb (James et al., 2013; Zubaer et al., 2018; Liu et al., 2020a,b). Linear versions have also been observed and some circular version could actually exist as linear concatemers generated by a rolling circle type DNA replication mechanism (Bendich, 1993; Hausner, 2003, 2012; Bullerwell and Lang, 2005; Baidyaroy et al., 2011; Valach et al., 2011; Chen and Clark-Walker, 2018). Mitochondrial genome architecture and size are highly variable among the fungi due to recombination events promoted by repeats and by the presence and activities of mobile elements such group I and group II introns and intron-encoded proteins (IEPs) (Aguileta et al., 2014; Wu and Hao, 2014, 2019; Franco et al., 2017; Repar and Warnecke, 2017; Deng et al., 2018; Stone et al., 2018; Zubaer et al., 2018; Kolesnikova et al., 2019; Kulik et al., 2020; Liu et al., 2020b). Like other fungi, most of the mitogenome variation observed among the examined members of the Ophiostomatales is due to the absence and presence of introns.

Gene order is conserved among the Ophiostomatales, and some minor variation was observed with regards to the tRNA gene set and the absence or presence of the *atp9* gene. It has been suggested that the loss of the mtDNA-encoded *atp9* gene can be compensated for by the presence of a nuclear-encoded version of this gene (Kanzi et al., 2016; Sellem et al., 2016; Franco et al., 2017; Zubaer et al., 2018).

Among the Ophiostomatales the number of mitochondrial introns and intron insertion sites is variable, except for mL2450. The mL2450 group IA intron encodes *rps3*, a configuration observed among many filamentous members of the Ascomycota. However, in some members of the Ascomycota *rps3* is a free-standing gene or missing from the mitochondrial genome (Korovesi et al., 2018; Wai et al., 2019). For the latter, such as in some members of the Capnodiales, Wai et al. (2019) identified a nuclear-encoded ortholog of the mitochondrial-encoded version of *rps3*. Among the metazoans and the fungi, the nuclear and mitochondrial versions of *rps3* have been shown to be required for initiating protein translation and appear to have “moonlighting activities” by being involved in DNA repair, cell signaling, apoptosis, and potentially in gene regulation (Neu et al., 1998; Kim et al., 2013; Wang et al., 2019; Seshadri et al., 2020). Maintaining the *rps3* gene within the *rnl* group I intron might be a fortuitous association that provides the *rps3* gene a locus embedded within an essential gene (*rnl*) presumable transcribed at a high rate, and this may “protect” the intron from being eroded by drift.

### Phylogenetic Analysis of Mitogenomes and Taxonomic Implications

The taxonomy of the Ophiostomatales has undergone considerable revisions in recent years; currently the Order Ophiostomatales includes two Families, Kathistaceae and the Ophiostomataceae. The latter includes the following genera: *Aureovirgo*, *Ceratocystiopsis*, *Fragosphaeria*, *Graphilbum, Hawksworthiomyces*, *Raffaelea sensu stricto*, *Ophiostoma sensu stricto*, and *Sporothrix sensu stricto* (Hyde et al., 2020). In addition, there are groupings for which monophyly is not certain such as *Leptographium sensu lato* (includes *Grosmannia* species, the *Raffaelea sulphurea* species complex, and *Esteya vermicola*) and *Ophiostoma sensu lato* (De Beer and Wingfield, 2013; De Beer et al., 2013a,b; De Beer et al., 2016a,b); the latter includes several smaller lineages with uncertain taxonomic positions (referred to as lineages A, B, C, and D; De Beer et al., 2016b).

The concatenated mitochondrial protein sequence data set confirmed the monophyly of the Ophiostomatales but it shows unresolved relationships among members that belong to *Leptographium*, *Raffaelea*, *Sporothrix*, and *Grosmannia*, suggesting that future work should include more members of *Leptographium*, *Grosmannia*, *Graphilbum*, *Hawksworthiomyces*, *Fragosphaeria*, and *Aureovirgo* in order to resolve phylogenetic relationships among the Ophiostomataceae. The original concept of *Sporothrix* was viewed to be a polyphyletic grouping, and this lead to the designation of the Genus *Hawksworthiomyces* with *Hawksworthiomyces lignivorus* (formerly *Sporothrix lignivora*) as the type species (De Beer et al., 2016a). *Sporothrix insectorum* was shown (Zhang et al., 2019) not to group with species of *Sporothrix sensu stricto*, and the current analysis aligns this species with *Hawksworthiomyces lignivorus*.

The analysis confirmed that the circumscription of *Raffaelea sensu lato* does not define a natural grouping (De Beer and Wingfield, 2013) with *Raffaelea quercivora*, *R. quercus-mongolicae*, and *R. sulphurea* possibly awaiting the designation of a new genus that can accommodate these ambrosia fungi. The placement of *Fragosphaeria purpurea* is unexpected as it groups with *Sporothrix insectorum* and *Hawksworthiomyces lignivorus*. This finding needs to be confirmed as previous studies based on nuclear markers placing *Fragosphaeria purpurea* very distantly from species of *Hawksworthiomyces* (De Beer et al., 2016b). The internal transcribed rDNA spacer sequences (ITS) recovered from the same genomic data set from which the *Fragosphaeria purpurea* mitogenome was derived matches (at 100% identity) ITS data available in GenBank for *F. purpurea* (AB278192.1 -mitogenome; and MN511357.1 for ITS). The ITS data for the *Sporothrix insectorum* (MK482392.1) strain included in this mitogenome survey shares 99.47% identity with those available for *F. purpurea*; however, a strain of *Sporothrix insectorum* (CBS 756.73; MH860798.1 – ITS) sequenced by Vu et al. (2019) deviates from the above (MK482392.1), suggesting the species concept of *Sporothrix insectorum* needs to be re-evaluated.

### The Mitogenome Intron Complement

Like a previous study (Zubaer et al., 2019), we observed that phase 0 introns outnumber introns positioned in phase 1 or 2. We speculated that this was due to core creep where the IEP coding region eventually extends to include the 5′ terminal intron sequence to fuse (in frame) to the upstream exon (Edgell et al., 2011). This would enhance the expression of the IEP as it would benefit from the host genes transcription and translation signals. This would entwine the intron and the HEG that may have started out as independent elements but now coevolve to maintain splicing and homing/mobility activities (Guha et al., 2018; Megarioti and Kouvelis, 2020).

The sizes of the mitochondrial genomes among members of the Ophiostomatales appear to be linked to the number of introns they contain. Similar observations have been made for other fungal groups (Kanzi et al., 2016; Liang et al., 2017; Zubaer et al., 2018). Introns can be gained by events that allow for cytoplasm to be exchanged between members of the same species or different species that would allow for the fusion of mitochondrial organelles enabling intron homing events and/or recombination between the different mtDNAs. The maintenance of introns has been assumed to be a matter of drift (neutral evolution) as a lack of selection would lead to an accumulation of mutations that could be deleterious to the intron and/or its protein coding components, leading to the eventual loss of the composite element (Goddard and Burt, 1999). Survival of these elements depends on inserting into new sites or reinvading sites where the intron was lost, a continuous cycle of gain, degeneration, loss, and reinvasion. We noted that members of the genus *Sporothrix sensu stricto* that are known to be pathogens on mammals have the smallest mitogenomes. Proliferation of introns may not be compatible with the life histories of these fungi.

Some introns could be beneficial such as those that encode proteins like RPS3 or in some instances N-acetyltransferases or aminotransferases (Wai et al., 2019). The latter two examples may provide metabolic flexibility for certain fungi, providing adaptive advantages (Duò et al., 2012). Mitochondrial introns have been associated with resistance to fungicides (Cinget and Bélanger, 2020) and hypovirulence (Baidyaroy et al., 2011). Introns are also a vehicle for modulating gene expression as their removal can be a rate limiting step for the expression of the genes (Rose, 2019), for example, in *Saccharomyces cerevisiae* mitochondrial functioning is linked to mitochondrial intron splicing (Rudan et al., 2018). Introns could be environmental sensors (Belfort, 2017), whereby splicing activity is influenced by environmental conditions and allows for gene expression to be fine-tuned to certain environmental cues.

### Complex Introns

Complex introns are composed of several intron modules possible, the result of one mobile intron invading another intron. We characterized the *cox3* i2 and *cob* i4 introns of *O. ips* (GenBank accession number: NTMB01000349.1) using computational strategies. These complex introns were composed of two and three intron modules, respectively, and could provide a platform for alternative splicing that may optimize intron-encoded protein expression and/or modulate host gene expression. For *cox3* i2, a tandem group I intron, splicing was predicted to occur as a composite unit, and the downstream intron was presumed to be the native intron.

The *O. ips cob* i4 intron was more complex; here the resident group I intron within its P1 component houses another group I intron that has been invaded by a group II intron module. A homing endonuclease ORF was detected within each of the two group I intron modules. For the three intron modules, many possible splicing patterns could be envisioned. The group II intron could modulate the splicing and expression of the internal group I intron component of this complex intron (see Hafez et al., 2013; Guha and Hausner, 2016; Guha et al., 2018) possibly having been co-opted to regulate the expression of the IEP. Plausible RNA interactions can be recognized that would allow for the group I modules to splice separately or as one composite intron. Detailed deep RNA sequencing analysis combined with RT-PCR based experiments are required to investigate if this insertion is a “zombie” intron (splices as one unit; Zumkeller et al., 2020) or a trintron where each module can splice individually or in various arrangements (isoforms) during RNA processing of the *cob* transcript. As the group I intron modules have similar sequence elements that allow for the formation of the helical regions (P1–P9) functionally they can act like tandem introns, i.e., side-by-side introns. A plausible pathway is shown in Figure 10; a ratchet-like (see Hafez and Hausner, 2015) mechanism could operate that removes the first intron module thereby generating an intermediate RNA molecule that regenerates suitable sequences for the second intron module to assume a splicing competent RNA fold including P1 and P10 interactions that allow for its removal and joining of the flanking exons. Splicing of the upstream intron component could generate a transcript whereby the downstream located ORF is fused in frame with the upstream exon, optimizing the expression of the downstream intron-encoded LAGLIDADG protein, a scenario we refer to as splicing-mediated core creep (Guha et al., 2018) where transcripts are generated that fuse the downstream located ORF sequence with the upstream exon. Similar splicing patterns demonstrating the “plasticity” of intron RNA folds have been previously observed (Sellem and Belcour, 1994; Turk et al., 2013). Tandem type complex introns, such as *O. ips cox3* i2, have been observed and described in the literature (Deng et al., 2016; Zubaer et al., 2018). These configurations need closer examinations in future studies to understand their splicing pathways and impact on mitochondrial gene expression.

## Conclusion

Comparing the mitogenomes of *O. minus* and *O. piliferum* with other available sequences for members of the Ophiostomatales showed that gene synteny is conserved and variability is mostly due to introns. The mitochondrial sequences show potential for resolving taxonomic questions among members of the Ophiostomatales, and as more genomes become available mitochondrial data will complement phylogenetic data based on nuclear markers. These insect-vectored fungi are potentially invasive and are a concern with regards to the biosecurity of forests; mitogenomics could provide a valuable tool in the identification and tracking of species belonging to the Ophiostomatales. Exploring fungal mitogenomes is important as some introns have the potential to serve as agents that can module gene expression and impact the phenotypes of the fungi that accommodate them (Cinget and Bélanger, 2020; Medina et al., 2020). These types of introns could be considered the result of constructive neutral evolution whereby complex systems evolve by non-adaptive mechanisms (such as drift) (Stoltzfus, 1999; Gray et al., 2010; Lukeš et al., 2011). In addition, ribozymes, complex introns (i.e., potentially co-operating ribozymes), and intron-encoded proteins have applications in biotechnology as genome editing tools and/or regulatory switches to control gene expression (Takeuchi et al., 2011; Guha et al., 2017; Belfort and Lambowitz, 2019).

## Data Availability Statement

The datasets presented in this study can be found in online repositories. The names of the repository/repositories and accession number(s) can be found in the article/ Supplementary Material.

## Author Contributions

AZ, AW, NP, and JP have been working under the supervision of GH and obtained data and contributed toward the analysis. AZ and AW took the lead with regards to assembling the datasets and the final analysis of the data. GH assembled the final version of the manuscript. All authors have contributed to the work, design the project, and worked on the manuscript.

## Conflict of Interest

The authors declare that the research was conducted in the absence of any commercial or financial relationships that could be construed as a potential conflict of interest.

## Footnotes

1 http://megasun.bch.umontreal.ca/cgi-bin/RNAweasel/RNAweaselInterface.pl

2 https://www.ncbi.nlm.nih.gov/orffinder/

3 https://www.ncbi.nlm.nih.gov/

## Supplementary Material

Supplementary Figure 1 Intron landscapes visualized with Circos generated diagrams. An overview of the intron landscape is illustrated in Figure 4 in **(A–D)** more detailed maps are presented maintaining the gene order as shown in Figure 4. Each panel shows one segment of the mitogenome: **(A)** *cox1*, *nad1*; **(B)** *nad4*, *atp6*, *rns*, *cox3*; **(C)** *rnl*, *nad2*, *nad3*, *atp9*, *cox2*; **(D)** *nad4L*, *nad5*, *cob*. Details were added with regards to intron types (subtypes), types of intron-encoded proteins (L, LAGLIDADG type ORFs; G, GIY-YIG type ORFs), and intron phasing [phase 0 = black lines, phase 1 = orange lines, and phase 2 = green lines, no phasing (for RNA gene) = purple line].

Supplementary Table 1 Comparison of the mitochondrial genomes and their intron complement for the studied members of the Ophiostomatales.

Supplementary Table 2 Intron subtypes and intron open reading frames (ORF) recorded noted within the mitogenomes of the examined members of the Ophiostomatales.

Supplementary Data File 1 Mitogenome assemblies and annotations.
