# Supplementary material for: The Mitogenomes of Ophiostoma minus and Ophiostoma piliferum and Comparisons With Other Members of the Ophiostomatales
Source: Front Microbiol. 2021 Feb 10;12:618649. doi: 10.3389/fmicb.2021.618649 (PMC7902536; doi:10.3389/fmicb.2021.618649)
Supplement: Supplementary Table 1 — Comparison of the mitochondrial genomes and their intron complement for the studied members of the Ophiostomatales. [file Table_1.docx]

**Table1: Comparison of the mitochondrial genomes and their intron complement for the studied members of the Ophiostomatales.**

| **Name of the Organism** | **NCBI Accession** | **Genome Size** | **Number of Introns in Genes** | | | | | | | | | | | | | | | | **Total Introns by Species** |
| --- | --- | --- | --- | --- | --- | --- | --- | --- | --- | --- | --- | --- | --- | --- | --- | --- | --- | --- | --- |
|  |  |  | *atp6* | *atp8* | *atp9* | *cob* | *cox1* | *cox2* | *cox3* | *nad1* | *nad2* | *nad3* | *nad4* | *nad4L* | *nad5* | *nad6* | *rnl* | *rns* |  |
| *Ceratocystiopsis brevicomis* | PCDN01000199.1 | 90,376 | 3 | 0 | 1 | 5 | 10 | 5 | 1 | 3 | 3 | 0 | 1 | 1 | 2 | 0 | 3 | 0 | 38 |
| *Ceratocystiopsis minuta* | LZPB01000172.1 | 39,800 | 1 | 0 | 1 | 1 | 6 | 0 | 0 | 1 | 0 | 0 | 0 | 1 | 1 | 0 | 1 | 0 | 13 |
| *Esteya vermicola* | KY644696.1 | 46,507 | 1 | 0 | 0 | 3 | 4 | 2 | 3 | 1 | 0 | 0 | 1 | 0 | 1 | 0 | 2 | 0 | 18 |
| *Fragosphaeria purpurea* | PCDL01000017.1 | 57,056 | 0 | 0 | 0 | 1 | 3 | 0 | 0 | 0 | 0 | 0 | 0 | 0 | 0 | 0 | 1 | 0 | 5 |
| *Graphilbum fragrans* | LLKO01000061.1 | 25,567 | 0 | 0 | 0 | 0 | 1 | 0 | 0 | 0 | 0 | 0 | 0 | 0 | 0 | 0 | 1 | 0 | 2 |
| *Grosmannia penicillata* | PCDK01000036.1 | 150,891 | 4 | 0 | 1 | 7 | 15 | 8 | 4 | 4 | 7 | 0 | 1 | 1 | 5 | 0 | 6 | 1 | 64 |
| *Hawksworthiomyces lignivorus* | NTMA01000166.1 | 27,092 | 0 | 0 | 0 | 0 | 0 | 0 | 0 | 0 | 0 | 1 | 0 | 0 | 0 | 0 | 1 | 0 | 2 |
| *Leptographium lundbergii* | LDEF01000080.1 | 101,879 | 2 | 0 | 0 | 1 | 13 | 5 | 2 | 3 | 2 | 1 | 0 | 0 | 2 | 0 | 3 | 2 | 36 |
| *Ophiostoma ips* | NTMB01000349.1 | 97,849 | 3 | N/A^a^ | 0 | 5 | 13 | 4 | 2 | 4 | 3 | 1 | 1 | 0 | 1 | 0 | 4 | 1 | 42 |
| *Ophiostoma minus* [WIN(M)495] | MW122509.1 | 91,847 | 3 | 0 | N/A^b^ | 1 | 9 | 7 | 2 | 3 | 3 | 0 | 0 | 0 | 3 | 0 | 5 | 2 | 38 |
| *Ophiostoma novo-ulmi* | MG020143.1 | 65,095 | 2 | 0 | N/A^b^ | 3 | 8 | 5 | 0 | 1 | 1 | 0 | 0 | 0 | 1 | 0 | 4 | 0 | 25 |
| *Ophiostoma piliferum* [WIN(M)959] | MW122508.1 | 69,966 | 2 | 0 | N/A^b^ | 6 | 9 | 4 | 0 | 3 | 1 | 0 | 0 | 1 | 2 | 0 | 1 | 0 | 29 |
| *Raffaelea albimanens* | PCDJ01000011.1 | 137,049 | 2 | 0 | 1 | 6 | 8 | 4 | 3 | 2 | 5 | 0 | 1 | 1 | 9 | 1 | 6 | 0 | 49 |
| *Raffaelea ambrosiae* | PCDI01000067.1 | 89,461 | 3 | 0 | 0 | 5 | 8 | 3 | 1 | 3 | 1 | 0 | 1 | 0 | 3 | 0 | 6 | 1 | 35 |
| *Raffaelea arxii* | PCDH01000124.1 | 95,042 | 1 | 0 | 1 | 4 | 15 | 3 | 2 | 2 | 3 | 1 | 2 | 0 | 4 | 0 | 5 | 0 | 43 |
| *Raffaelea lauricola* | PCDG01000206.1 | 70,090 | 2 | 0 | 0 | 2 | 6 | 3 | 0 | 3 | 1 | 0 | 0 | 1 | 0 | 0 | 3 | 0 | 21 |
| *Raffaelea quercivora* | PCDE01000018.1 | 152,890 | 3 | 0 | 0 | 4 | 6 | 3 | 0 | 2 | 3 | 0 | 0 | 1 | 5 | 0 | 7 | 1 | 35 |
| *Raffaelea quercus-mongolicae* | NIPS01000008.1 | 106,194 | 3 | 0 | 0 | 2 | 12 | 5 | 2 | 3 | 4 | 1 | 1 | 1 | 3 | 0 | 3 | 0 | 40 |
| *Raffaelea* sp. | PCDF01000414.1 | 23,830 | 0 | 0 | 0 | 0 | 0 | 0 | 0 | 0 | 0 | 0 | 0 | 0 | 0 | 0 | 1 | 0 | 1 |
| *Raffaelea sulphurea* | PCDD01000156.1 | 128,408 | 2 | 0 | 1 | 3 | 6 | 4 | 1 | 3 | 1 | 0 | 1 | 0 | 0 | 2 | 9 | 3 | 36 |
| *Sporothrix brasiliensis* | AWTV01000012.1 | 35,826 | 1 | 0 | 0 | 0 | 4 | 0 | 0 | 1 | 0 | 1 | 0 | 0 | 0 | 0 | 2 | 0 | 9 |
| *Sporothrix globosa* | LVYW01000010.1 | 26,671 | 0 | 0 | 0 | 0 | 0 | 0 | 0 | 0 | 0 | 0 | 0 | 0 | 0 | 0 | 1 | 0 | 1 |
| *Sporothrix insectorum* | MK482392.1 | 31,500 | 0 | 0 | 0 | 0 | 3 | 0 | 0 | 0 | 0 | 0 | 0 | 0 | 0 | 0 | 1 | 0 | 4 |
| *Sporothrix pallida* | CM003773.1 | 35,458 | 0 | 0 | 0 | 2 | 2 | 1 | 0 | 0 | 0 | 0 | 1 | 0 | 0 | 0 | 1 | 0 | 7 |
| *Sporothrix schenckii* | AB568600.1 | 26,095 | 0 | 0 | 0 | 0 | 0 | 0 | 0 | 0 | 0 | 0 | 0 | 0 | 0 | 0 | 1 | 0 | 1 |
| **Total Introns by Genes** | | | 38 | 0 | 6 | 62 | 161 | 65 | 23 | 42 | 38 | 6 | 11 | 8 | 42 | 3 | 78 | 11 | 594 |

**^a^** Mitochondrial scaffold incomplete and segment with *atp8* is missing.

**^b^** *atp9* is absent (as previously observed in *Ophiostoma novo-ulmi* (Abboud et al., 2018).
