# Supplementary material for: The Mitogenomes of Ophiostoma minus and Ophiostoma piliferum and Comparisons With Other Members of the Ophiostomatales
Source: Front Microbiol. 2021 Feb 10;12:618649. doi: 10.3389/fmicb.2021.618649 (PMC7902536; doi:10.3389/fmicb.2021.618649)
Supplement: Supplementary Table 2 — Intron subtypes and intron open reading frames (ORF) recorded noted within the mitogenomes of the examined members of the Ophiostomatales. [file Table_2.docx]

**Table S2.** Intron subtypes and intron open reading frames (ORF) recorded within the mitogenomes of the examined members of the Ophiostomatales.

| **Gene** | **Position^a^** | **Phase** | **Subgroup^b^** | **Count^c^** | **ORF^d^** | **ORF count^e^** |
| --- | --- | --- | --- | --- | --- | --- |
| *atp6* | 521 | 2 | IC2 | 4 | GIY |  |
|  | 572 | 2 | IC2 | 16 | GIY |  |
| *atp8* | N/A | N/A | N/A | N/A | N/A | N/A |
| *atp9* | 187 | 1 | IA | 6 | GIY |  |
| *cob* | 155 | 2 | IB | 5 | LAG |  |
|  | 201 | 0 | IB | 9 | LAG |  |
|  | 393 | 0 | ID | 14 | GIY |  |
|  | 406 | 1 | IA | 1 | - |  |
|  | 429 | 0 | ID | 2 | LAG |  |
|  | 437 | 2 | IB | 4 | LAG |  |
|  | 490 | 1 | IA | 12 | LAG |  |
|  | 506 | 2 | IB | 6 | LAG |  |
|  | 562 | 1 | IA | 2 | LAG |  |
|  | 820 | 1 | IB | 7 | LAG |  |
| *cox1* | 105 | 0 | II | 1 | RT | 1 |
|  | 212 | 2 | IB | 13 | GIY |  |
|  | 216 | 0 | II | 1 | - | 0 |
|  | 240 | 0 | IB | 7 | LAG |  |
|  | 278 | 2 | GIY | 1 | GIY |  |
|  | 281 | 2 | IB | 13 | LAG |  |
|  | 313 | 1 | II | 2 | RT | 2 |
|  | 372 | 0 | IB | 3 | LAG |  |
|  | 386 | 2 | IB | 10 | LAG |  |
|  | 493 | 1 | IB | 4 | GIY |  |
|  | 540 | 0 | IC2 | 3 | LAG |  |
|  | 607 | 1 | IB | 1 | LAG |  |
|  | 615 | 0 | IB | 7 | LAG |  |
|  | 709 | 1 | ID | 10 | LAG |  |
|  | 720 | 0 | IB | 3 | LAG |  |
|  | 731 | 2 | IB | 10 | LAG |  |
|  | 807 | 0 | IB | 1 | LAG |  |
|  | 821 | 2 | IB | 1 | LAG |  |
|  | 867 | 0 | IB | 12 | LAG |  |
|  | 900 | 0 | IB | 5 | LAG |  |
|  | 971 | 2 | IB | 1 | LAG |  |
|  | 1057 | 1 | IB | 13 | GIY |  |
|  | 1059 | 0 | U | 1 | GIY |  |
|  | 1107 | 0 | IB | 1 | LAG |  |
|  | 1125 | 0 | IB | 10 | LAG |  |
|  | 1262 | 2 | IB | 8 | GIY |  |
|  | 1281 | 0 | IB | 7 | GIY |  |
|  | 1296 | 0 | IB | 11 | GIY |  |
| *cox2* | 93 | 0 | IC2 | 5 | LAG |  |
|  | 120 | 0 | IC2 | 9 | LAG |  |
|  | 207 | 0 | IC2 | 3 | LAG |  |
|  | 234 | 0 | IB | 12 | GIY |  |
|  | 267 | 0 | IC2 | 2 | LAG |  |
|  | 324 | 0 | IA | 4 | LAG |  |
|  | 363 | 0 | ID | 4 | LAG |  |
|  | 558 | 0 | IC2 | 7 | GIY |  |
|  | 598 | 1 | IC2 | 6 | GIY |  |
|  | 606 | 0 | IC1 | 3 | GIY |  |
|  | 657 | 0 | IC1 | 11 | GIY |  |
| *cox3* | 219 | 0 | IB | 4 | LAG |  |
|  | 333 | 0 | IC2 | 4 | LAG |  |
|  | 428 | 2 | ID | 1 | LAG |  |
|  | 550 | 1 | IA | 4 | LAG |  |
|  | 631 | 1 | IA | 2 | LAG |  |
|  | 640 | 1 | IA | 8 | LAG |  |
| *nad1* | 144 | 0 | IC1 | 2 | GIY |  |
|  | 145 | 1 | IA | 9 | GIY |  |
|  | 166 | 1 | IB | 6 | GIY |  |
|  | 291 | 0 | IC2 | 7 | LAG |  |
|  | 388 | 1 | IA | 5 | GIY |  |
|  | 636 | 0 | IB | 13 | GIY |  |
| *nad2* | 258 | 0 | IC2 | 4 | LAG |  |
|  | 420 | 0 | IC2 | 6 | LAG |  |
|  | 591 | 0 | IC2 | 3 | LAG |  |
|  | 612 | 0 | II | 2 | RT | 2 |
|  | 792 | 0 | IC2 | 1 | LAG |  |
|  | 810 | 0 | IC2 | 5 | LAG |  |
|  | 1038 | 0 | II | 1 | RT | 1 |
|  | 1242 | 0 | IC2 | 4 | LAG |  |
|  | 1332 | 0 | IB | 1 | LAG |  |
|  | 1698 | 0 | U | 3 | LAG |  |
|  | 1719 | 0 | IA | 8 | LAG |  |
| *nad3* | 90 | 0 | IC2 | 6 | LAG |  |
| *nad4* | 585 | 0 | IC2 | 1 | LAG |  |
|  | 658 | 1 | IC2 | 10 | LAG |  |
| *nad4L* | 239 | 2 | IC1 | 8 | LAG |  |
| *nad5* | 248 | 2 | ID | 9 | LAG |  |
|  | 324 | 0 | IC2 | 10 | LAG |  |
|  | 426 | 0 | ID | 3 | LAG |  |
|  | 570 | 0 | IB | 6 | LAG |  |
|  | 710 | 2 | ID | 3 | LAG |  |
|  | 717 | 0 | IB | 5 | LAG |  |
|  | 924 | 0 | IB | 2 | LAG |  |
|  | 1000 | 1 | IB | 1 | GIY |  |
|  | 1152 | 0 | IC2 | 3 | LAG |  |
| *nad6* | 120 | 0 | U | 1 | LAG |  |
|  | 233 | 2 | ID | 1 | LAG |  |
|  | 312 | 0 | II | 1 | RT | 1 |
| *rnl* | 576 | N/A | II | 1 | - | 0 |
|  | 722 | N/A | II | 1 | LAG | 1 |
|  | 742 | N/A | IA/IB | 8 | GIY |  |
|  | 812 | N/A | IC1 | 2 | GIY |  |
|  | 965 | N/A | IC1 | 10 | GIY |  |
|  | 1006 | N/A | IC2 | 1 | LAG |  |
|  | 1096 | N/A | IC2 | 1 | LAG |  |
|  | 1700 | N/A | IA | 7 | LAG |  |
|  | 1924 | N/A | IB | 2 | LAG |  |
|  | 1968 | N/A | IC2 | 3 | GIY |  |
|  | 2029 | N/A | ID | 2 | LAG |  |
|  | 2406 | N/A | IC2 | 1 | LAG |  |
|  | 2450 | N/A | IA | 25 | RPS3 |  |
|  | 2501 | N/A | IB | 3 | LAG |  |
|  | 2507 | N/A | IC2 | 1 | - |  |
|  | 2529 | N/A | IC2 | 2 | LAG |  |
|  | 2585 | N/A | IA | 7 | LAG |  |
|  | 2597 | N/A | II | 1 | - | 0 |
| *rns* | 379 | N/A | II | 2 | RT | 2 |
|  | 569 | N/A | IC2 | 1 | LAG |  |
|  | 913 | N/A | ID | 3 | LAG |  |
|  | 952 | N/A | II | 2 | LAG | 2 |
|  | 1210 | N/A | IC2 | 2 | LAG |  |
|  | 1383 | N/A | IC2 | 1 | LAG |  |
| **Total** | 119 | 95 | N/A | 594 | N/A | 12 |

**^a^** Position of intron insertion (insertion site) with respect to a reference sequence without introns; see Materials and Methods for more details

**^b^** Refers to most observed intron subgroup within the insertion site and was based mainly on predictions by MFannot/RNAweasel and manually identified for those missed by the programs; U = undefined (i.e. no intron subgroup predicted by MFannot/RNAweasel and manually identified); more than one intron subgroup can be associated with one insertion site

**^c^** Refers to the number of fungi containing the insertion; some insertion sites may encode for more than one intron, where “intron” refers to (at least) the presence of elements composing the intron core

**^d^** Refers to the most observed Open Reading Frame (ORF) encoded within the intron insertion site; LAG = LAGLIDADG homing endonuclease, GIY = GIY-YIG homing endonuclease, RT = reverse transcriptase, - = no ORF containing identifiable (based on BLASTx) motif

**^e^** ORF count was restricted to only ORFs encoded within group II introns where ORFs could be unambiguously identified; several cases where a group I intron encoded multiple ORFs in various configurations (ex. one followed by another, one within another)

N/A = not applicable
