# Supplementary material for: The Mitogenomes of Ophiostoma minus and Ophiostoma piliferum and Comparisons With Other Members of the Ophiostomatales
Source: Front Microbiol. 2021 Feb 10;12:618649. doi: 10.3389/fmicb.2021.618649 (PMC7902536; doi:10.3389/fmicb.2021.618649)
Supplement: Supplementary Data File 1 — Mitogenome assemblies and annotations. [file Table_3.DOCX]

LOCUS Ceratocystiopsis 90376 bp DNA linear 17-JAN-2019

DEFINITION .

ACCESSION

VERSION

KEYWORDS .

SOURCE Unknown.

ORGANISM Unknown.

Unclassified.

REFERENCE 1 (bases 1 to 90376)

AUTHORS Wai, Zubaer, Hausner

TITLE Direct Submission

JOURNAL Submitted (17-JAN-2019) Department of Microbiology, University of

Manitoba, 66 Chancellors Circle, Winnipeg R3T 2N2, Canada

FEATURES Location/Qualifiers

source 1..90376

/organism="Ceratocystiopsis brevicomis"

/mol_type="genomic DNA"

gene 1..17615

/gene="cox1"

CDS join(1..212,2622..2649,3878..3918,5200..5533,8026..8119,

9421..9442,10455..10590,11711..11968,14176..14331,

15887..15901,17325..17615)

/gene="cox1"

/EC_number="1.9.3.1"

/codon_start=1

/transl_table=4

/product="cytochrome c oxidase subunit 1"

/translation="MSIERWFLSTNAKDIGVLYLMFALFSGLLGTAFSVLIRMELSGP

GVQYISDNQLYNSIITAHAILMIFFMVMPALIGGFGNFLLPLVIGGPDMAFPRLNNIS

FWLLPPSLILLVFSACIEGGAGTGWTIYPPLSGIQSHSGPSVDLAIFALHLSGISSLL

GAMNFITTVINMRTPGIRLHKLSLFGWAVVITAVLLLLSLPVLAGGITMVLTDRNFNT

SFFETAGGGDPILFQHLFWFFGHPEVYILIIPAFGIISTTISANSNKSVFGYIGMVYA

MMSIGILGFIVWSHHMYTVGLDVDTRAYFTAATLIIAVPTGIKIFSWLATCYGGSIKL

TPSMLFALGFVFMFTIGGLSGVLLANAALDTAFHDTYYVVAHFHYVLSMGAVFALFSG

WYFWIPKILGLSYNILLSKVHFWVLFIGVNLTFFPQHFLGLQGMPRRISDYPDAFTGW

NLVSSFGSIVSVIATVLFLYIVYKQLIDNNPVGRFPWLMPQYFTDALQALLNRNYPSL

EWALTSPPKPHAFVSLPLQS"

exon 1..212

/gene="cox1"

/number=1

intron 213..2621

/gene="cox1"

/note="Group IB"

/number=1

gene 448..984

/gene="orf178"

CDS 448..984

/gene="orf178"

/codon_start=1

/transl_table=4

/product="hypothetical protein"

/translation="MDPYWVTGFVDAEGCFGFRIRKNPNLKVGWEVIPYFFINLHSKD

LTILSGLANFFGVGNIVVVSKNDSVQYQVNSTGDIFKAIIPHFDNYPLITKKTRRLFK

QALELIKNKEHLTLDGIKKLVAIKASLNTKTLTEDLKSTFKDIVPENIVKPLLPELLN

PYWFAGFTSGDGSFSVEI"

gene 1165..2019

/gene="orf284"

/note="copy 1"

CDS 1165..2019

/gene="orf284"

/codon_start=1

/transl_table=4

/product="hypothetical protein"

/translation="MPLFNLYPIIGIKKRDFEDWCKIAELMNNKAHFTLEGLDKIRNI

KVGMNKNRKFTNTSSDFLEENTPSTIIPQRGNIDTEIETETEIVTKTGKSNTLIFISN

NQDNGYIPNEIYMNNINFTKVIVENPYNNRNYIQKLTKEKRGCYVWESNNHVYVGHSI

NLYNRISSYFMPSILKTKARRVLRYFNKHGFQDANLTIYIMNENSTLDEVVRLEQHFI

DTLNPSLNVDLIASSSGYHEPMSQEIRERLRKQRGTPVYVYDAKDFTLYLNQNNIHMI

QLIFIIKL"

exon 2622..2649

/gene="cox1"

/number=2

intron 2650..3877

/gene="cox1"

/note="Group IB"

/number=2

gene 2650..3591

/gene="orf313"

CDS 2650..3591

/gene="orf313"

/codon_start=1

/transl_except=(pos:2650..2652,aa:Lys)

/transl_table=4

/product="hypothetical protein"

/translation="KNRIQTLTTLVSSNDEKSGLNLLRSKLGPYLAGLIEADGSFAVH

DKNSRAKKYAPKIIIVFSLNDSPLAEKLASITQVGKIYKRENQGCILWSIQNSGDVIK

IVHIINGYMRTPKIEALHRTITWYNDNINTDIQPLGLDLSPIDSNSWLAGFTDGDGNF

SINLTDRKKKGVVTSKRVQAFFRIELRQNYHREVSVVQGGVSYFEILDKIARYLKVNL

YSRSREQKDKIFYAYMVISHSLESHLKTMEYFDRYPLYSSKHLAYKDWKSVVKQIQLR

NGKPLTAENIKEIQRIKDQFNNNRVQFDFSHLDTIMN"

exon 3878..3918

/gene="cox1"

/number=3

intron 3919..5199

/gene="cox1"

/note="Group IB"

/number=3

gene 3920..4795

/gene="orf291"

CDS 3920..4795

/gene="orf291"

/codon_start=1

/transl_except=(pos:3920..3922,aa:Asn)

/transl_table=4

/product="hypothetical protein"

/translation="NKKAHLGINIRNYSSNLKTDNFKSYLAGLFEGDGHIWVQKPSEK

KKQNPRFCITFGMKNEPLAKRLLELIGSGFIRYKLQDNACVLVVSPVIGLKKIVNLIN

GELRTPKIHQLHNLIDWLNKNHSTNITKLPLKTSFLSEDGWLSGFIDSDGSFSIVHTK

LENGAKKRKIACRLRIEQRMLDPITNESYEQVLSSIANFLNCSLLTKTQISTGNSYYT

LAASSKISLKIIIDYLEKYPLFSSKYLDYKDWKKVVLLILDCKHLTEESLVIISTAKN

SMNRQRTFFNWDHLN"

exon 5200..5533

/gene="cox1"

/number=4

intron 5534..8025

/gene="cox1"

/note="Group IB"

/number=4

gene 5534..7453

/gene="orf639"

CDS 5534..7453

/gene="orf639"

/codon_start=1

/transl_except=(pos:5534..5536,aa:Gly)

/transl_table=4

/product="hypothetical protein"

/translation="GILPALYLANCWKEIDILSLSAGCFIILFLWSFFRDYMPKFICY

NKLYSTISTFNNNFDSNFASYLAGLIEGDGTIVTPKVERSPKGKLYYPTIQIVFDIRD

FPLAQIIQSKLKHGSLARKKGTNAYILTINSFEGMILIVNIINGYMRTPKIISLYKLI

DFLNHKFDLKIEKKDKDSSSIGSNSWLAGFIDADGHFSVRTTLEGKYPKIECKFEISI

FCPYNDLYIEICKNLMKGIATFLEIKDDSFKQVKTSKGFVFSVRTQSIKNNEILLNYL

SSFPLFSSKYLDCQDFSLIFEILKKYYKTRLELDNVDLELINTHKKRIYQRTEFIWDH

LGNLYNGLDTTQFRISGLRIKEEFFTINTLKSSGFTRGFSSCSCLNTRSINDNNLNKS

CLYVSSVIYTDMLLEKKLLLDDNKEKSGIYLLTHLESKKMYVGSSADLYRRLKYYFSK

VNLTRNTNSRIYNALLHYGHSAFSLTILEYVNTSGLTKSEAKNIIIGKEQFYIDLLKP

EYNILNTAGSLLGFKHSYDTILKFKEAKNKENNPMFSKQHTEEAILKMRLRRLGKLLT

EETRLKIGSTLSRKVFIFKYDSISDKKVFIAKFNNYSEAAQYFNCSIRTLSRYIDKDI

LYKKNFYIYTKNISK"

gene 7507..7905

/gene="orf132"

CDS 7507..7905

/gene="orf132"

/codon_start=1

/transl_table=4

/product="hypothetical protein"

/translation="MDMLRVGRRTLLSQRQNDHNNENNFEFLNLIADFLGANVKEIRR

YKPNPEYRVRTTNLKSNLVLVGYLDKFPLFSSKYLNYKDWKTILNYFEAKLHTNPDTI

QKIVDIKAEMNNNRTNFNWDHLEYFYNLYK"

exon 8026..8119

/gene="cox1"

/number=5

intron 8120..9420

/gene="cox1"

/note="Group ID"

/number=5

gene 8122..9033

/gene="orf303"

CDS 8122..9033

/gene="orf303"

/codon_start=1

/transl_except=(pos:8122..8124,aa:Thr)

/transl_table=4

/product="hypothetical protein"

/translation="TKQTYIIIFILLSSLTLYTVYNSTLSTFNFKSFYLEYSNLYPNC

KLPDSKFLEWFIGFTEGEGSFTVAKRGDLSFVITQSTTDIECLNFIKDNLGFGKVIKQ

SIKSNTHRFVVQDIKNLILICKLFNGNMVFPTRNAKFLIFLSAINEKLLKKNMNTISI

IDNTVLPSLNDGWLSGITDGEGSFTCSILSEPNSGYRFRYILTQKWDSNKPILEFINR

LFGDKVGAIYSHHESKNNFNIWELRINGVKNCESLFEYFDTFTLNSKKRLSYIKWKEV

HSKLKNGEHLISDKRLEIKDLCKKINK"

exon 9421..9442

/gene="cox1"

/number=6

intron 9443..10454

/gene="cox1"

/note="Group IB"

/number=6

exon 10455..10590

/gene="cox1"

/number=7

intron 10591..11710

/gene="cox1"

/note="Group IB"

/number=7

gene 10591..11628

/gene="orf345"

CDS 10591..11628

/gene="orf345"

/codon_start=1

/transl_except=(pos:10591..10593,aa:Trp)

/transl_table=4

/product="hypothetical protein"

/translation="WVTMASPYSDIGITINFAICWNSLVLISPLYGKNLISYTQSADN

LSLYSLKDNKQSVPETTRETSLKFSTFHAYYNTLFKNRNSISDDWLTWFIGFAEGAIQ

TYDNGKRVRFVLTQSDILNKIQFKFNIGVVKHFPQGKSGKNNDFHRWMVDNPSDILLL

AHLFNGNLAQSHRIEQLALWANALNNRFGSDTIKLNNTPATITLQDAWLSGFTDAEGC

FNVSITENSRYTLGHVIKMRYLLDQKDSIILNKVYELFGFGKVTLRSGTDNVYRYTAT

GFKALNDIIAYFKLFPLKTKKAISFEKWLTIHNQVQNKLHLTEEGLSQIRTMQKKINL

NNSMTNKTGKA"

exon 11711..11968

/gene="cox1"

/number=8

intron 11969..14175

/gene="cox1"

/note="Group IB"

/number=8

gene 11969..12943

/gene="orf324"

CDS 11969..12943

/gene="orf324"

/codon_start=1

/transl_except=(pos:11969..11971,aa:Glu)

/transl_table=4

/product="hypothetical protein"

/translation="EVGLYNLPLDYFATDYMLGTLGWWYYLLIIKTFSNRNKFSFNSK

KSNASSKVVIYPNLSAENCEGFSETIRQSLNRVNFWNRFASVLDTKGYFDFISFNKKK

ILKQIIIKLDYNDIRILKYLQNYTHVGRIISIKNKSMYIIEVEDDLRYILNNLNGFIR

IKVPEFKEACSFFGIEFKEANYTIKKNDKTYFSGLIDANGKIVFNQDSCIIECNLILK

NNEYINKLNLNHAIPRSKPTRIENAFLFQKFRDMSSIYKYFMKSKLYSNIKFYRVNKI

NYYLNIFLNKSNLKYKKKYNDSYYFLKNWIKYNNIYWYKNTYVKDIYI"

gene 13233..14078

/gene="orf281"

CDS 13233..14078

/gene="orf281"

/codon_start=1

/transl_table=4

/product="hypothetical protein"

/translation="MLLFDVVYLKVINDKLDLKGSQYFENKWDSKLNLLYNTIYNYTK

LINPFFLLLSVYFINFFFGDINSIYFYTLKLLLQFSFLINLIYLNKIWIDQKIIKNDF

PVFHMLIKLILGGCLIYNVAVIIYTLYVICVLIYNYVLKFNFWNKIKGWRLSFEYNKF

KGPNDPKDSTLLYSEDKKKNKKTASELKDLILKAQTKKAKSNLNPDLKLESSNFNRNW

EKKINIDKLPDFSIEDQIRNLEKEYVAYDLQEKKFKDIVVNIGKKKKNFILPMHQIYL

KNMWI"

exon 14176..14331

/gene="cox1"

/number=9

intron 14332..15886

/gene="cox1"

/note="Group IB"

/number=9

gene 14848..15852

/gene="orf334"

CDS 14848..15852

/gene="orf334"

/codon_start=1

/transl_table=4

/product="hypothetical protein"

/translation="MLINKKDSNRFYIGSSTNLARRMEEYNKLTKGLRSPRSSSELEI

SKTSALDWKLEFIYITTPQTSLVFEQFAIIKNKPTINGFIQVIPRFNPQWGKLDGAIV

TIKNLLSLFPKCSEGYNRLAAFDSVYSAANNIKYTAEDLDNKYYCFLIYAYDLNLPDK

NPVIYSSINRALKGLQISHSTLLNHIYNKYIFVTESNTLLSFEPLSKEEFFEYQEKPA

GDNQLRKHIEVFNQDNELVVSFKSGREMARFFKIDGKVARAAITNGEYQDFLLISKEV

SNRKTIYVFDSSSHELLEEIKGVSKALKYAKVNFYTLKSLIETGKSQNGKIYSYEDKL

"

exon 15887..15901

/gene="cox1"

/number=10

intron 15902..17324

/gene="cox1"

/note="Group IB"

/number=10

gene 16327..16950

/gene="orf207"

/note="copy 1"

CDS 16327..16950

/gene="orf207"

/codon_start=1

/transl_table=4

/product="hypothetical protein"

/translation="MKILKNKNNNFVEQDWDNDDLVEKTINNNNLITIKPEHKFSQEC

KLNLVNLSSTPDSPLYNFLFDKEIILKEYKNKSGIYLLHNNVNGKEYVGSAKDLSKRL

ATYYFPSRLIDSRFISNSILKYGHDNFSLVILHVLGDSNSSSKSDIISKEQEYIDLYK

PILNLNPVAGSSMGFKHSEESKRLISEFHKGKPLSEDTKKKLSLQVN"

exon 17325..17615

/gene="cox1"

/number=11

gene 17752..24758

/gene="nad1"

CDS join(17752..17896,20094..20239,21626..21970,24324..24758)

/gene="nad1"

/EC_number="1.6.5.3"

/codon_start=1

/transl_table=4

/product="NADH dehydrogenase subunit 1"

/translation="MLRFPTLVSIIEVVLVLVPALLAVAYVTVAERKTMASMQRRLGP

NAVGYYGLLQAFADALKLILKENIAPNQANLTLFFLGPVITLVFSLLGYGVIPYGPGL

AISDLSLGIFYLLAVSSLSTYGILLAGWSANSKYAFLGSLRSTAQLISYELVLSSAIL

LVVFITGSLNLTVNMEAQRAVWFILPLLPIFIIFFIASVAETNRAPFDLAEAESELVS

GFMTEHAAVVFVFFFLAEYGSIVLMCILTSILFIGGYLIIGSSIIDISLYIYDSNFIV

NYIINSPIIEGLLYGISLGIKSSIMIFVFIWTRASFPRIRFDQLMSFCWTVLLPVLFA

IIILIPAILYNFILFPINISLL"

exon 17752..17896

/gene="nad1"

/number=1

intron 17897..20093

/gene="nad1"

/note="Group IA"

/number=1

gene 19102..19569

/gene="orf155"

CDS 19102..19569

/gene="orf155"

/codon_start=1

/transl_table=4

/product="hypothetical protein"

/translation="MLSKSDIYKSMGIKTKIWVYDPNTLSLLNNTYFSSIQSAMDYLK

TSRRSVLKYLDSGLILKSNNSLFYLFSQETNREKLEMLNNNPLSKSLVKSDVWVYSNS

SGTLVLLNENLPFSSINRTAVALGLSSHTIKKYLDKNISFKNYYFYSNKQDNI"

exon 20094..20239

/gene="nad1"

/number=2

intron 20240..21625

/gene="nad1"

/note="Group IC2"

/number=2

gene 21035..21604

/gene="orf189"

/note="copy 1"

CDS 21035..21604

/gene="orf189"

/codon_start=1

/transl_table=4

/product="hypothetical protein"

/translation="MFKEIASIMQLKEHLTTEGIQKIVNLKATLNFGLSKELQLMFPE

TIPVARPLREPCVIPHSQWIAGFTAGEGNFSVSLDKGVFKSLLFKITQHKRDEVLLMA

IKDYFNCGNCYLRKIEYTMDLKVTKFSDVTDIIVPFFIKNSILGVKSLDFKDWCLVSE

IVKKREHKSEEGIKRIQEIQKGMNRGRSF"

exon 21626..21970

/gene="nad1"

/number=3

intron 21971..24323

/gene="nad1"

/note="Group IB"

/number=3

gene 22800..23141

/gene="orf113"

CDS 22800..23141

/gene="orf113"

/codon_start=1

/transl_table=4

/product="hypothetical protein"

/translation="MSDTSYKVWLYFQITQHNRDIALMERIVTFFGCGSVKKRNTDAV

DFKLNKFELLENIIIPFFQKYPLQSAKNLDFSGFVEAADAIKSKTARQWTTEQFDKIK

NIQNNMNKYIK"

gene 23928..24296

/gene="orf122"

CDS 23928..24296

/gene="orf122"

/codon_start=1

/transl_table=4

/product="hypothetical protein"

/translation="MLGYKHTDEAKQKMVERFKDITNHPMYGKKHSEDILKLISKPGK

LNPMYGKTHSDETKKAISIKMSKYPNGVGIFDLNNNLIKKFNNNTEIANYLGISKVTV

AKYLNNDLIYQDMYKFKPIL"

exon 24324..24758

/gene="nad1"

/number=4

gene 24866..27651

/gene="nad4"

CDS join(24866..25376,26699..27651)

/gene="nad4"

/EC_number="1.6.5.3"

/codon_start=1

/transl_table=4

/product="NADH dehydrogenase subunit 4"

/translation="MLLSFLLLVPIVGIFLIAGAISYQDNVIKTTYYKNIALITSIVN

LIISLVVYMLFDSNTNQFQFVQEHYNLSYFDIYLGVDGISIYFVLLTTIIMPIAILAN

WNSITDNIKSYLIIMLLLETLLLAIFLVLDILLFYIFFESTLPPLFLLVGLFGSNNKV

RASYYLFLYTLWGSLFLLIGILAISSIMGTTDFDALFKTNFDYITQLVLFSGIFLSFA

VKTPTIFLNSWLLKAHVESPLGGSIVLAAIVLKTSLYGIFRLILPILPKASINFTYIV

YVIGVITIIYASFSTLRTTDIKELIAYSSVSHAAVYLMGVFSNTIQGIEGGILLGLGH

GFVSSGLFICAGGILYDRSGTRSIFFYKGIAQIMPLFAILFLILSLGNCGVPLTLNFV

GEFMSLYGIFERLPLLGVFASSSIILSAAYTMYMFNRIGFGGTFSKYFKENIADLTKR

EFFILLTLVIFTVVLGVYPSIVLDGLHYSVSGLIYNQ"

exon 24866..25376

/gene="nad4"

/number=1

intron 25377..26698

/gene="nad4"

/note="Group IC2"

/number=1

gene 25379..26617

/gene="orf412"

CDS 25379..26617

/gene="orf412"

/codon_start=1

/transl_except=(pos:25379..25381,aa:Asn)

/transl_table=4

/product="hypothetical protein"

/translation="NLKCKRAKHRGSPKALVTKALKEILKLAWLMPQGMVKSLVFYLE

KLFYITDMWVIAVLSHLFFKGVKEQRVDGSSVSSNLDIVRCTLVAGKPVLSRKIYTDY

NKSIIINKRFSSSKSNLNPWFVTGITDAEGCFMLGIFKSNNYKMGYQVQGIFKITLHK

KDYDLLSQIKDYFKVGTITNHGDTTLQYTVKSIKDLDKIIYHFDKFTLLSQKSVDYLL

FKNGIMLIKNKEHLSYEGLKKILSLRAAMNLGLPEELKFNFPDVIPFSRPSTLRLNSI

DYNYISGLTSGDGCFYVSIRNAPSTKTGKSVILKFQIVQHSRDIELMRKLISTLNCGR

IELNLKQSAVYFVVTNFQDIVNQIIPLFDKYPIKGVKSLDYADFKSVVNLMQTKEHLT

QEGLSIIQSLKLNMNLFRKL"

exon 26699..27651

/gene="nad4"

/number=2

gene 27877..28035

/gene="atp8"

CDS 27877..28035

/gene="atp8"

/EC_number="3.6.3.14"

/codon_start=1

/transl_table=4

/product="ATP synthase F0 subunit 8"

/translation="MPQLVPFYFLNEVIFTFSIITIIVYLFSKYILPRFVRLFLSRTF

ISKLFDNK"

gene 28106..34375

/gene="atp6"

CDS join(28106..28461,29794..29970,31716..31766,34168..34375)

/gene="atp6"

/EC_number="3.6.3.14"

/codon_start=1

/transl_table=4

/product="ATP synthase F0 subunit a"

/translation="MNTLSINTVNLETLSPLSQFEIKDLLSIDAPLLGNLHISLTNIG

FYLSIGAFIILTLSLLSTNYNKLISNNWSISQETLYATVHSIVTNQINPRNGQMYFPF

IYTLFIFILINNLIGMVPYSFASTSHFVLTFSLSFTIVLGATILGFQKHGLEFFSLLV

PAGCPLALLPLLVLIELISYLARNISLGLRLAANIMSGHMLLHILAGFTYNIMSSGII

FFFLGLLPLAFIIAFSGLEIGIAFIQAQVFVVLTSSYIKDGLDLH"

exon 28106..28461

/gene="atp6"

/number=1

intron 28462..29793

/gene="atp6"

/note="Group IB"

/number=1

gene 28463..29515

/gene="orf350"

CDS 28463..29515

/gene="orf350"

/codon_start=1

/transl_except=(pos:28463..28465,aa:Asn)

/transl_table=4

/product="hypothetical protein"

/translation="NRSLCNLSLFISNIFEVLMLHNNLVFQTQNYSSFHSSTQVNKSN

HDPRYSFNNKNTFYLNPNYITGFVDGEGCFSISIYKQDKNLTGWQVKPVFSISLHNKD

IGLLEAIQRTFKTGKIYKHGIDSVQYRVSSLKNLQIITNHFDAYPLITQKRADYILFK

QAISMIKNKEHLCTEGLLKLVGIKATLNWGLSDKFKESFPSVKPAIRPSVMYNNLKIG

DFNWIRGFIEAEGSFQVIIQNSKNVSLRFSITQHAKDEELLKDIVSYLNCGRYYKSPT

RDEGQYLVTVFTDIDNKIIPFFNEYSLLGIKKYDCRDFLLIFELIKSKAHLTDEGLEK

IKHIKDNMNKNRIIEE"

exon 29794..29970

/gene="atp6"

/number=2

intron 29971..31715

/gene="atp6"

/note="Group IC2"

/number=2

gene 30303..31574

/gene="orf423"

CDS 30303..31574

/gene="orf423"

/codon_start=1

/transl_table=4

/product="hypothetical protein"

/translation="MCSLFIISLVLKLRLILCNDSLDTYVEPTRKYNRTQFKLYYSPV

SCIYTCKRGISTSSNVEPVKVYLYPDKEKVEIIRENKDRVGIYRWVNLHSGKSYIGSS

SNLSIRFKQYFNYNHITYPKRNLRIYKALLKYGYSGFRLEILEYCYPEILLQREQFYF

DTCNPEYNILKVAGSPLGYRHSEAAKKLISLASKDRNVSEETREIKRGILLGKSLDRE

HVENMRLGNTLRKPVLVTDIETGDTKEFISLTEAGEYLGISRVTVGKYLLNGTYYKNY

KISSSSSSAGRTNETSVSNNTLSYSQQAIFFNNIETGDVKEFVSMSDAAKYLDISRAR

LWYFFNKTDSSMNDTIKGYSVSKLDSTDVKATRKTKELEVTDLETNKVTIYPSFTLAA

KDLNISTSSISGYLAKQRTNPFRKRYIFKLV"

exon 31716..31766

/gene="atp6"

/number=3

intron 31767..34167

/gene="atp6"

/note="Group IC2"

/number=3

gene 32717..33418

/gene="orf233"

CDS 32717..33418

/gene="orf233"

/codon_start=1

/transl_table=4

/product="hypothetical protein"

/translation="MILKDNKNKVGVYRWVNKLNGDSYIGSSVDLTRRFRVYYDFSYL

SVRIQKSKSIIYSAILKHGYSNFQLEILEYCCKETVISREQYYIDLFKPRYNINPTAS

SRLGTTHSESTKEKMSNLVKGRKLTEHTKNLLSLANKGINNANFGKRHSAETKALISL

ARIGKSFLSESIKDKMSAEKGVSIKVLDLKTSTVSVYTSITRAAKEMRVTQPSLSKRL

KETQGPIKNVSKLKK"

gene 33487..34143

/gene="orf218"

CDS 33487..34143

/gene="orf218"

/codon_start=1

/transl_table=4

/product="hypothetical protein"

/translation="MPICSALLKYGYNNFSLTILEFCNIDSLMSREKHYFEVYSPEYN

VLKTPGSPDRGSGWKHSEATVEIMRLAAQKRIESPEYINKISEAQLNRIEIEVTDLKT

NTSTKYHAIRAAARALNIDKRYIENYVYLNQNKPVLGRYTFKLLSKEETNYIPKVQST

SIKVEVTNIETKEITIYPSIGAAAKELGYHQASISLYLKENRTKPFKGLHLFKLVKDN

"

exon 34168..34375

/gene="atp6"

/number=4

gene 34662..36162

/gene="rns"

rRNA 34662..36162

/gene="rns"

/product="small subunit ribosomal RNA"

gene 36200..36286

/gene="trnY(gta)"

tRNA 36200..36286

/gene="trnY(gta)"

/product="tRNA-Tyr"

/anticodon=(pos:36235..36237,aa:Tyr,seq:gta)

gene 37049..37756

/gene="orf235"

CDS 37049..37756

/gene="orf235"

/codon_start=1

/transl_table=4

/product="hypothetical protein"

/translation="MICKALLKNGYNNFTLEILEYCEPSEAILREQYYIDLLKPGYNI

LKFAGSTLGYKHSEDTKAKLSSVLKAVWKSEISDTLKAKLSASWTKERRLKMILLKEG

KPLSDETRAKISAALKEVWQSEEKRAKYLASKKKRGVIVTEETKAKMSAANKGVKKSE

ETKAKMSAAHMGNKHGPGYHGQIEILDTVTEIKTVYSSMSVLSKALSIPKPSISAYFS

RNTGKPYKGRYIMTKVN"

gene 37811..37881

/gene="trnN(gtt)"

tRNA 37811..37881

/gene="trnN(gtt)"

/product="tRNA-Asn"

/anticodon=(pos:37843..37845,aa:Asn,seq:gtt)

gene 37957..41876

/gene="cox3"

CDS join(37957..38596,41707..41876)

/gene="cox3"

/EC_number="1.9.3.1"

/codon_start=1

/transl_table=4

/product="cytochrome c oxidase subunit 3"

/translation="MDIKQRSLFQDHPFHLVSPSPWPLYTSLSLLALTSNAALFVHNF

EYASYLSFVSLLCLITSMFFWFKDITTEGTYLGDHTLVVQQGINIGIMLFIISEVFFF

LAIFWAFFHSALSPTVELGAQWPPFGIQPVNPFELPLLNTVILLSSGATVTYAHHSLI

SGVRSTAVYYTLISIWLAIIFTFFQGVEYEVSSFTISDGVFGACFFFGTGFHGLHVII

GTLFLLVALALIVSYHATDSHHQGYESAILYWHFVDVVWLFLYIAMYYWGS"

exon 37957..38596

/gene="cox3"

/number=1

intron 38597..41706

/gene="cox3"

/note="Group I(derived),IA"

/number=1

gene 38599..39657

/gene="orf352"

CDS 38599..39657

/gene="orf352"

/codon_start=1

/transl_except=(pos:38599..38601,aa:Thr)

/transl_table=4

/product="hypothetical protein"

/translation="TTLVLLLSEYSFINFTYPFNFYFKTVKNFYYIMINKRFFSTDEL

LSYQPKNKGLEQLCPYWVTGFCDAESSFSLKVSKKSTSKSGWGVIPDFRIELHSRDTL

LLRKIQSFFGVGIISERLDRNTIVYSVQSFKDIFNIIIPHFDKYPLITKKQADYILFK

EAILLLCTKAQHSIEGIHKILSIKAAMNTGLSDSLKNQFPTVFSVARPYITLKDIPSP

SWVAGFTDGEGCFYVNTKKAKTLTGYQIIMSFSISQHVRDEVLLTKFIDYFGCGNIEK

VSTRPSTVTFVVYKYSDIKDKIIPVFEKYSLQGIKNLDYLDFCKIANVMENKSHLTAD

GVKKIKSLKSGMNSGRTL"

gene 40546..41373

/gene="orf275"

CDS 40546..41373

/gene="orf275"

/codon_start=1

/transl_table=4

/product="hypothetical protein"

/translation="MLTKNKEHLTDKGKLAIIDYKKEMQSMSGKWVPSFIYNKINITK

PWLAGFIDGEGSFSTNKYVPRFKLENHIKETELYHKIKEFMGTPKLIYTSTGSREDSN

PTIVLEINKIKYIKETLIPLMYDSNNKLLLKTLKSNNFLLWVNLVDMYYLGYHTTLEG

KHLFDLIKNNINKYSLTTNSHLKREFKTLSEINLLLSELYTLNSPYEIIDGLRYYRGT

NKLVSESTQIVSIDENNCKVEFNSMSEAANALNISRKYIKECLASGKSYKGYTFVLK"

exon 41707..41876

/gene="cox3"

/number=2

gene 42471..42971

/gene="orf166"

CDS 42471..42971

/gene="orf166"

/codon_start=1

/transl_table=4

/product="hypothetical protein"

/translation="MVHHYTDDHGYRCSTFSTNKDIHGPYQEVPPYPANTYLWNLEYK

ILYRVKTQFYHDNMIEYVKNIPVDGAPQNPRLPTNYPFYYKGFNRPMNQMEINQALHT

LVTVHHHNSQRICFYQSPLVTDGTYSDRDYNNLMTASLVNLRYRNKYASIDHYLLLNK

LKRTPW"

gene 43023..43094

/gene="trnK(ttt)"

tRNA 43023..43094

/gene="trnK(ttt)"

/product="tRNA-Lys"

/anticodon=(pos:43055..43057,aa:Lys,seq:ttt)

gene 43106..43178

/gene="trnD(gtc)"

tRNA 43106..43178

/gene="trnD(gtc)"

/product="tRNA-Asp"

/anticodon=(pos:43139..43141,aa:Asp,seq:gtc)

gene 43186..43265

/gene="trnS(gct)"

tRNA 43186..43265

/gene="trnS(gct)"

/product="tRNA-Ser"

/anticodon=(pos:43218..43220,aa:Ser,seq:gct)

gene 43269..43339

/gene="trnW(tca)"

tRNA 43269..43339

/gene="trnW(tca)"

/product="tRNA-Trp"

/anticodon=(pos:43301..43303,aa:Trp,seq:tca)

gene 43404..44051

/gene="nad6"

CDS 43404..44051

/gene="nad6"

/EC_number="1.6.5.3"

/codon_start=1

/transl_table=4

/product="NADH dehydrogenase subunit 6"

/translation="MFIINENFTNGYRSEVLDILCLLIILSGIFVIVSKNPIISLLFL

IGLFAGVSCYLLIIGLNFLGLSYLVVYVGAVSILFLFILMLINIRMSELQSNTSNSIP

LAISIAILFNYPLFQLLPYNVAILNNNNIINNILYNISFNKIDNGLKTNLDINNNDTL

FVTSKIWDGNLAEVGHISSIGNIFYTNYNIWLIIVSFILLLAMVGAITINIKQKN"

gene 44058..44129

/gene="trnV(tac)"

tRNA 44058..44129

/gene="trnV(tac)"

/product="tRNA-Val"

/anticodon=(pos:44090..44092,aa:Val,seq:tac)

gene 44130..44200

/gene="trnI(gat)"

tRNA 44130..44200

/gene="trnI(gat)"

/product="tRNA-Ile"

/anticodon=(pos:44162..44164,aa:Ile,seq:gat)

gene 44206..44290

/gene="trnS(tga)"

tRNA 44206..44290

/gene="trnS(tga)"

/product="tRNA-Ser"

/anticodon=(pos:44240..44242,aa:Ser,seq:tga)

gene 44298..44369

/gene="trnP(tgg)"

tRNA 44298..44369

/gene="trnP(tgg)"

/product="tRNA-Pro"

/anticodon=(pos:44331..44333,aa:Pro,seq:tgg)

gene 44923..45279

/gene="orf118"

CDS 44923..45279

/gene="orf118"

/codon_start=1

/transl_table=4

/product="hypothetical protein"

/translation="MVRTIKVRTGYCCKNIRGIVVSIVKDNSDCISWFSAKPILVGNL

NNILAGTELNLRQDVLNIFFLYKSGNHEDFTGEFVDSEWQRWILNYQTYNDKVEIQPI

CRSEPSAINHQIAGTP"

gene 45567..46316

/gene="orf249"

CDS 45567..46316

/gene="orf249"

/codon_start=1

/transl_table=4

/product="hypothetical protein"

/translation="MSQLNNKNIVPVKSYDNTFLNKKTILVENKGKSGIYRWINKLNN

NTYIGSGLDLSKRLSEYYSQSELKRNPRPIHAALLKYGYENFILEILEYCEVDELIER

EQYYLDLLDPEYNILKYAYSLLGYKHTPENIAKFKLRKVSQELKDILSSAHSGKEVSQ

ETRDKLSLAITNYKKNNPLSPEALANIRAKTTEREGVTVILLNTETNEELEFPTLTKA

GEYLGIKRQAIRSAIKVLLKDYIVFQKKNKQ"

gene 48228..49529

/gene="rps3"

CDS 48228..49529

/gene="rps3"

/codon_start=1

/transl_table=4

/product="ribosomal protein S3"

/translation="MNIKNNFIGENNYFPSDYKEWSNNIYYYNNNNIKNIPAYDLVIN

KLLKGYFNSYLNPNINFLYSNKRYTSLNKIFISKANIRHTNSKAIITIYVYDREKISL

SKFFIKYSHIVLAAIKELKEFVLENNNFIQNKLKSFQGDNELLKLLNFFKSRKLKAWL

NELRYKDIFIYKLSKLISKFYRKKIEFNIIRLTSIAYNSDILVDVMRKSYKKTGHVRN

SMKFILNQGLIEKIDNTNDRVRATKNVDFNLLANKYQNLNINYIACVRSAVKDVNINE

TIKNLYNTKNKNNEEIIFNSIKYKILGGIRIDIKGRLTRRFRADRSVYNTILKGSFRN

IDSSYKGLTILGYRGCASSSVDYSMSISKRHVGAFAIKGWISGRSYSTSTRNNSNLDT

INLIDYLGNIDPWTITGFTDAEGSFMLRIKKNDQSSIKYSV"

gene 50763..51134

/gene="orf123"

CDS 50763..51134

/gene="orf123"

/codon_start=1

/transl_table=4

/product="hypothetical protein"

/translation="MTNTTMIRFINSLNKLETYKKELRIKTKLSLYLNDSLIGLLLSD

GYIERTSPTSGARLTISFGAKYEGYFNHLYKLFEPYINTEPSLISVYNKKTDDTYKVW

RFKTASLPQLIYYHDLFYKPN"

gene 52082..52152

/gene="trnT(tgt)"

tRNA 52082..52152

/gene="trnT(tgt)"

/product="tRNA-Thr"

/anticodon=(pos:52114..52116,aa:Thr,seq:tgt)

gene 52155..52227

/gene="trnE(ttc)"

tRNA 52155..52227

/gene="trnE(ttc)"

/product="tRNA-Glu"

/anticodon=(pos:52188..52190,aa:Glu,seq:ttc)

gene 52413..53774

/gene="orf453"

CDS 52413..53774

/gene="orf453"

/codon_start=1

/transl_table=4

/product="hypothetical protein"

/translation="MKNVFKLRNKVVYLSTLKDKSIVANLECFALKGYLVHRPFTHKR

YSSTTQPLVFNLPSFEFLEWFRGFTDAEGCFLIAKSANSFAFRFIIKLHKDDLNILNF

IRASLNNIGNVSVYEDSASFKVTSLSEIKFIIEMFSTYPLNSDKHLNFLDFKKAYELY

TLPVDGPQDINNEIIKLKINMNSKRAKHDIDVNSEYLINSDNHEIIITDSWLLGFIEG

DGSFSVAKENNMLLFSISQKGNLVLMEAIKIYLLELAKKMGHGTLNTVYLTKSKNSTG

NIAYVLIIKSKEFISHVLIPYFDTLTFYSKKKLDYFDWKSIDKLKSLGLHYLPEGQKL

INLITGQMNNNRLSTFKSSDLRPVDRDLIDSELTRLLNGPSNYELIEGKIFIKSLNKF

LPNRLKTQVKLQDNKGLVFKEFESKNKCAEFLGISTHTVSKRIITNKPVNYKNKEYLI

KYA"

gene 53800..53872

/gene="trnM(cat)"

/note="copy 1"

tRNA 53800..53872

/gene="trnM(cat)"

/product="tRNA-Met"

/anticodon=(pos:53833..53835,aa:Met,seq:cat)

gene 53904..53974

/gene="trnM(cat)"

/note="copy 2"

tRNA 53904..53974

/gene="trnM(cat)"

/product="tRNA-Met"

/anticodon=(pos:53936..53938,aa:Met,seq:cat)

gene 53978..54060

/gene="trnL(taa)"

tRNA 53978..54060

/gene="trnL(taa)"

/product="tRNA-Leu"

/anticodon=(pos:54012..54014,aa:Leu,seq:taa)

gene 54068..54138

/gene="trnG(tcc)"

tRNA 54068..54138

/gene="trnG(tcc)"

/product="tRNA-Gly"

/anticodon=(pos:54100..54102,aa:Gly,seq:tcc)

gene 54143..54214

/gene="trnA(tgc)"

tRNA 54143..54214

/gene="trnA(tgc)"

/product="tRNA-Ala"

/anticodon=(pos:54176..54178,aa:Ala,seq:tgc)

gene 54221..54293

/gene="trnF(gaa)"

tRNA 54221..54293

/gene="trnF(gaa)"

/product="tRNA-Phe"

/anticodon=(pos:54254..54256,aa:Phe,seq:gaa)

gene 54306..54387

/gene="trnL(tag)"

tRNA 54306..54387

/gene="trnL(tag)"

/product="tRNA-Leu"

/anticodon=(pos:54340..54342,aa:Leu,seq:tag)

gene 54430..54502

/gene="trnQ(ttg)"

tRNA 54430..54502

/gene="trnQ(ttg)"

/product="tRNA-Gln"

/anticodon=(pos:54463..54465,aa:Gln,seq:ttg)

gene 54900..55547

/gene="orf215"

CDS 54900..55547

/gene="orf215"

/codon_start=1

/transl_table=4

/product="hypothetical protein"

/translation="MFTINLNKKDLTLLEQIKNYFGNGSISPSGNNCLQLKIADLNVL

INNVLPHFDKYPLVTQKRADYLLVKKVIFLMKDKKHLGPNYQEGLNEIISIKASINLG

LPDRLKTLFPNITPINRPLVEFSNIPDPNWLTGFVSGDGSFEINISKSSSSILNKRVI

LRFRVTQHIRDIDLMKSLILYFNCGSVYETKDAVWFQITKISDIDEKIIPFNTRS"

gene 55760..55833

/gene="trnH(gtg)"

tRNA 55760..55833

/gene="trnH(gtg)"

/product="tRNA-His"

/anticodon=(pos:55794..55796,aa:His,seq:gtg)

gene 55898..55970

/gene="trnM(cat)"

/note="copy 3"

tRNA 55898..55970

/gene="trnM(cat)"

/product="tRNA-Met"

/anticodon=(pos:55932..55934,aa:Met,seq:cat)

gene 56026..56097

/gene="trnV(cac)"

tRNA 56026..56097

/gene="trnV(cac)"

/product="tRNA-Val"

/anticodon=(pos:56059..56061,aa:Val,seq:cac)

gene 56140..62541

/gene="nad2"

CDS join(56140..56709,59075..59263,60577..62541)

/gene="nad2"

/EC_number="1.6.5.3"

/codon_start=1

/transl_table=4

/product="NADH dehydrogenase subunit 2"

/translation="MLLISILSLLLSNAVTIRRDISILFNRVAIIALIYSILHSVTTL

FILGKGIGLHGGLLNVTSITQVFNIFIFLVSILILQLTSFFPRKVWIPEHSSLMQLLF

NNLVLYRTKIINKMGEHLRIIEYPLILLFIISGAVFLISTSDLISVFLAIELQSYGLY

LLSTIYRNSELSTTGGLMYFLLGGLSSCFILLGTSLLYANSGTTNLDAIYAITSLSDS

SDIWYKPYYINFAFLIFSIGFLFKVSAAPFHFWSPDVYDAIPTIVTTFVAIVAKISIF

IFLLEIVYYTKNYFTDFNWTYGLLISSFLSLIIGTVVGLTQFRIKRLFAYSTISHVGF

ILLALSISSVESTQAFIFYLMQYSISNLNAFLILVAIGFSLYCYINNNKEYKDLLDKN

NSPIQLISQLKGYFYINPTLALSLTITLFSFAGIPPLAGFFAKQMVLSAAIDNGYIFI

TLVAISTSVIGAVYYLNIIKEIFFFSPEYKINPLLENLNFNGNIYNNKNVLIKSINFK

YNNIAISSSISITISIITLIILLFIFVNKEWLSMSTIYCLFFLCAEVHFVRNLLLLLN

KKEPLSSNYSKNTYFPQGLYNTCVSNLPRSLYYKVSLRSYSSYASKKQIYPVSPWFIS

GFTDAEGCFNVGIQKNSNGKYYVKPSFQIKVHSRDNLLLMSIKHYLGNIGNIYISNTD

SNFTVKSLADLLKIISHFNNYPLMTKKKADFLLFEEIVSKIVEGEHLSAKGLQEIVNI

RASINLGLSPALKTNFPNTIPVARPDIEKIDTIHPEWMSGFVTGEGCFLVNLSKYGKD

RLDGVSLSFKVSQHSRDELLLRSFISFFGCGLFNYHDKNKKAGIFIVRKFSDIYDKIL

PFFEKHQILGIKKEDFEDWSKVAKLINSKDHLTEEGLEKIRKIKSGMNTLR"

exon 56140..56709

/gene="nad2"

/number=1

intron 56710..59074

/gene="nad2"

/note="Group II"

/number=1

gene 56710..58968

/gene="orf752"

CDS 56710..58968

/gene="orf752"

/codon_start=1

/transl_except=(pos:56710..56712,aa:Val)

/transl_table=4

/product="hypothetical protein"

/translation="VRLCALCYYVFKEYRYFSNVSPATELGKGEILMLNIASLLKGRN

GRNQISLRTYRAESPMRRAILPELHSLGALTWGLNHFWDRIYDLCITLRWRVLQGNRF

NLGTISTLNVKSGVSKGDIKITLRTGGLPTALKGHGNRGVVVLLAGRAPASGVCMMST

SADGISTVSTDGLNKIQKINELCSENKSFIVTDKLYNIMYSKDVFSAAYHKLKSKPGN

MTPGIVPTTLDGMSDEEITRIISTLRDGSFKFNPGRRVYIPKSNGGERPLTIAPPRDK

LVQEVMRMILEAIYEPAFQKCSHGFRPNKSCHTALRDVRQKFGMAKWFIEGDISKCFD

SVDHNILMGIISSRIKDQRFLDLIRKALKAGYMEAREYSHSLAGTPQGSIISPILANI

YLDKLDSFVMELKTGFDRGVKASINPEYKRLSSRKDRAKDTLMKRMINLIMLKTASKL

HIDPNFKKLEYVRYADDWIIGVRGSRKDCEVLIMEIRDFLDKNLNLRLSEEKTKITNA

SKDLATFLSVGIQRKTHRTLRRMVQGHNRRNVNNLRLLAPINKVTSKLTENGFMKNGT

PYPKFRWMSNEKDTIILLYNSVYRGIMNYYRFVDNYNNLSSKVHYILKNSCARLLAAK

YKSTQAKIYKEYGKNMKGDNKHGFIEIILGIKLAAFSPKTNDVLFRFNAEGISKTSLE

DLSCSVCQSEYRVEMHHVRMMKDLDPKKSLVDRLMIKRRRKQIPLCRSCHMELHRKDN

RDNNKNFKNKRK"

exon 59075..59263

/gene="nad2"

/number=2

intron 59264..60576

/gene="nad2"

/note="Group IC2"

/number=2

gene 59958..60527

/gene="orf189"

/note="copy 2"

CDS 59958..60527

/gene="orf189"

/codon_start=1

/transl_table=4

/product="hypothetical protein"

/translation="MIMQRKEHLTVEGLQKIVNIRASLNRGLTPLLSEAFPNSIPFTR

PSLPVLSDKLDPQWVAGFTSGDGCFKISIRESKLHKAGSRVTLLFIVTQHIRDELLLK

SLVDFFGCGQTYSYKDYIEFRCQSFKDNYENILPFFNKYPILGVKSLDFEDWAKVAKM

IQTKVHLTNEGFDQIRLIRKGVNKGRYIK"

exon 60577..62541

/gene="nad2"

/number=3

gene 62943..63353

/gene="nad3"

CDS 62943..63353

/gene="nad3"

/EC_number="1.6.5.3"

/codon_start=1

/transl_table=4

/product="NADH dehydrogenase subunit 3"

/translation="MSSMSIFFIFVIIIAILFLALNLIFAPSNPYQEKYSAFECGFHS

FSQSRSQFNITFFIYALVFLLLDLEILLLYPYAVSSYSNDIYGLIIVLIFTTIVTIGF

VFELGKGALKIGSRQDVESISKKNTIIISLIGTK"

gene 63465..63662

/gene="atp9"

CDS 63465..63662

/gene="atp9"

/EC_number="3.6.3.14"

/codon_start=1

/transl_table=4

/product="ATP synthase F0 subunit c"

/translation="MIQVAKIIGTGLATTGLIGAGVGIGVVFGALILGVARNPSMRGQ

LFAYAILGFAFSEATGSNGSV"

gene 64289..64735

/gene="orf148"

CDS 64289..64735

/gene="orf148"

/codon_start=1

/transl_table=4

/product="hypothetical protein"

/translation="MMLLNVCFAQKWIDFLKPSYNLSPTAGNTKGYKHSAESIEKMRY

KALGRKHTEEVKQSMSESRKGENNPFYGKTHSEDTIELLKTTAANRLKSPVPGIEVEI

TDIETKLTHTCESIRKAASFMGSDIKTILRREETLKVLILPIKKST"

gene 64916..73856

/gene="cox2"

CDS join(64916..65026,66488..66607,67470..67598,69994..70188,

71737..71835,73758..73856)

/gene="cox2"

/EC_number="1.9.3.1"

/codon_start=1

/transl_table=4

/product="cytochrome c oxidase subunit 2"

/translation="MYYFFINLMIKLDAPSAWGIYFQDSATPQMEGLVELHDNIMYYL

VIILFGVGWVLLSITKNFIISASPISHKYLNHGTLIELIWTITPAVILILIAFPSFKL

LYLMDEVSDPSMSILAEGHQWYWSYAYPDFLDSSEEFIEFDSYIVPESDLEEGGLRML

EVDTRVIIPELTHIRFIITSGDVIHSFACPSLGIKTDAYPGRLNQVSVFVNREGVFYG

QCSEICGILHSSMPIVIESVSLEKFLTWLEEQ"

exon 64916..65026

/gene="cox2"

/number=1

intron 65027..66487

/gene="cox2"

/note="Group IC2"

/number=1

gene 65472..66077

/gene="orf201"

CDS 65472..66077

/gene="orf201"

/codon_start=1

/transl_table=4

/product="hypothetical protein"

/translation="MENYKNKYSGLNPHYVTGFSDGEACFHLAIGKNSKYKIGYYVNP

GFSIVLHKKDEQLLRNIQSFFGGIGNLKVKSNIVQYRIFSLKELDILLEHFDNYPLIT

KKFVDYELFKEALIIMKNKEHLTIEGFNKIIALRASMNLGLPETLKEAFPDVKIREII

PKDLPSLLNPYWVAGFTDAEGCFWIKTLKDSTKNKVSTVFK"

exon 66488..66607

/gene="cox2"

/number=2

intron 66608..67469

/gene="cox2"

/note="Group IB"

/number=2

gene 66608..67231

/gene="orf207"

/note="copy 2"

CDS 66608..67231

/gene="orf207"

/codon_start=1

/transl_except=(pos:66608..66610,aa:Arg)

/transl_table=4

/product="hypothetical protein"

/translation="RYVPTRKYFKFNILNKMRLYTTIPPSGIKFYENAYLMRRLIINE

NKNKSGIYKWTNKLTNDIYIGQSTNLSRRFIRYFNYSYLKIWDTPAISRVLVTHGYIN

FSLEILEYCKKFDLREREQYYIDKLNPKYNTLNIAGSSSYYNHIEETKGSHVLIIVKN

GLERPKLIGSFLSIRKAARFLGISSNTIRFYINSGKWIRGRYKLISN"

exon 67470..67598

/gene="cox2"

/number=3

intron 67599..69993

/gene="cox2"

/note="Group ID"

/number=3

gene 67599..68420

/gene="orf273"

CDS 67599..68420

/gene="orf273"

/codon_start=1

/transl_except=(pos:67599..67601,aa:Phe)

/transl_table=4

/product="hypothetical protein"

/translation="FIICGLKLFILYVFYKIKDASNIINISGEKNNTYIYKRLAQVKY

IGYNSYAKNARFTLELKRAFHTKIRASSRIGPHDKDVLSVIIGSLLGDAYANARTIEG

TRISYRQSNVHKEYLFWLYDFFFQRGYCSNLKPRKYTRTLKGKEFYGYEFNTFTFRSF

NWIHKLFYKKGIKYINPNLESYLTPLALAIWIMDDGTWAGNGVRIATNSFKFEEVKIL

GNMLVKLYGLNYTIQTIEGRHSIYITKESIPKLINLTLPHIIPSMKYKLGIKDNQ"

gene 69014..69874

/gene="orf286"

CDS 69014..69874

/gene="orf286"

/codon_start=1

/transl_table=4

/product="hypothetical protein"

/translation="MLNFSKSSESKHGFRIRGIFQIELHEKDFELLKTIQSFFGGIGY

ITAATKNCVAFRARSIEDLQIIIAHFDKYPLKTKKRADFDLFRIAVNKLSLKEHLKLE

GFKEIVGIRASMNNGLTDTLKFAFPDVTPALRPTLFDELGINSSWLAGFVSGEGCFFI

NLATSASNKIGYRISLLFTISQHTRDRELMNSVVNFLECGDIKESSSRVNILTYKVTD

FSKIVEKIIPFFRDNRVLGVKSDDFSSWCQAIELMKDKRHLTEEGLNKIRLIKSNMNK

GRVLDPEAEV"

exon 69994..70188

/gene="cox2"

/number=4

intron 70189..71736

/gene="cox2"

/note="Group IC2"

/number=4

exon 71737..71835

/gene="cox2"

/number=5

intron 71836..73757

/gene="cox2"

/note="Group IC1"

/number=5

gene 72312..73700

/gene="orf462"

CDS 72312..73700

/gene="orf462"

/codon_start=1

/transl_table=4

/product="hypothetical protein"

/translation="MHNMIDKNIFNNRFLQLCHKSSGLGLIATRRCFSTSTVKANKFN

PESLALDHINSNKPTTSSVINKILLNQNISLTESKLEELLKVNGVELDLPISTSEDKN

LLSELTGKSNYKGFFGVYMFIHKNTGDKYVGSSNLLRRRLDYYFKSLESQAADLSYTG

KFLPFLKKEGLSAFKLIIFKLDNKKFNVKDALILEQYHLLNKEFNLNTLRVVNAGSSK

GESIYVYDLSCKILYYQSSSKIGLKRVLKIHPETVAKYLDSKIPYLKKFLLLSFPIPG

VSFSDMSVTKLLEMMQEERKNLYILGTRRSISVLLEIKEGNTKVADNCWGQTLNFESL

TSCIEYLKNIGLYIKRDTLSKYIKTGKEFHKFLCRYSDKNLPSNFEEIGLILDEYKQT

LAYFDVYLAKENKKNKPVLVKSENYNNEFNSITDTIKYFDSINVKLDRKTLYLRLKDG

KPYKGYYFGHKL"

exon 73758..73856

/gene="cox2"

/number=6

gene 73891..73963

/gene="trnR(acg)"

tRNA 73891..73963

/gene="trnR(acg)"

/product="tRNA-Arg"

/anticodon=(pos:73924..73926,aa:Arg,seq:acg)

gene 74060..75331

/gene="nad4L"

CDS 74060..75331

/gene="nad4L"

/EC_number="1.6.5.3"

/codon_start=1

/transl_table=4

/product="NADH dehydrogenase subunit 4L"

/translation="MNITLILFLIGILGFVLNRKNIILMLISIEIMLLSITFLILISS

LNIDDIIGQTYAIYIIVVAGAESAIGLGILVAFYRLNRSLFSYYLSYNYTPMSFYKKI

PQQIRYYSTNAYDRNISNHYIDPWFITGIFDAESSFVVTVLKNPRYKTGWNVQARVQI

KMHEIDRVLIESIRNYFGNIGYISKPNNKNLTVEFRISTLKDLIDIIIPHFDKYPLKT

KKYIDFLLFKDIVLLMSKKEHNTIEGIKKIIYIKTSLNTGLTENLKEAFPNAFPLNNL

DYNNRPVGPIHPKWMAGFCTGESNFFITVQKSNTKTGLAISLRFSISQHSRDLLLLES

FVKFFNCGYVSNYKTRLVTEFIVTKIGDIYKHIIPFFEENLILGSKYSNYLDFNQAAK

IIINKEHFNKEGLEKILFLKNKMTTFYKEKL"

gene 75788..81507

/gene="nad5"

CDS join(75788..76035,78438..78513,79870..81507)

/gene="nad5"

/EC_number="1.6.5.3"

/codon_start=1

/transl_table=4

/product="NADH dehydrogenase subunit 5"

/translation="MYLSIIILPLLGSIVSGFFGRKVGVSGAQIITCSSITITTILAI

IAFFEVGINNIVLSINLFRWIDSEWFNIIWGFQFDSLTVSMLIPVLIISTLVHFYSIG

YMSGDPHNQRFFSYLSLFTFMMIILVTANNYLLMFVGWEGVGVCSYLLVSFWFTRIAA

NQSSMSAFITNRVGDCFLTIGMFAILWSLGNLDYSTVFSLAPYVNENIIIIIGVCLLI

GAMAKSSQVGLHVWLPMAMEGPTPVSALIHAATMVTAGVYLLMRSSPLIEYSSTILLL

CLWLGAITTVFSSLVGFFQQDIKKVIAYSTMSQLGMMVIAVGLSSYNVALFHLVNHAF

YKGLLFLGAGAVIHAMADNQDFRKYGGLISFLPLSYSVILIASLSLVAFPFMTGFYSK

DFILESAYGQYYFSSITVYIIAVIGAIFTTLYSVKVLYLTFLTNPNGPLINYKHAHES

DIFMSLPLVILAIFSIFFGFLTKDIFIGLGSGFFIDNSIFIHPNSEIMIDTEFGVSTY

WKLLPFVFTVSFSTIAIILSEFLSENIVNFKLSTTGKTIFGFFNQRFLVEFFYNKYIT

NLIFKLGGQTVKVLDKGSIELLGPYGLEKKLINSSKNISSLNKGIVTNYALFILVGFI

LYMFSITLGFTNNLFLIILLLIFIISSIY"

exon 75788..76035

/gene="nad5"

/number=1

intron 76036..78437

/gene="nad5"

/note="Group ID"

/number=1

gene 76037..77635

/gene="orf532"

CDS 76037..77635

/gene="orf532"

/codon_start=1

/transl_except=(pos:76037..76039,aa:Lys)

/transl_table=4

/product="hypothetical protein"

/translation="KDMGLLEGTNAVLVLIQLCEMSIINFYLHMFMYPPFSSITGFRS

YILGAEPTKCYKSNISELKIYPRVNNKLNIGLCSPRRQYSTLKNSNIVAGGSEENFQS

SIKLPSDSTFLQWFVGFTDAEGNFIINPLKNTKLDISRFSFMFKIALHKDDSEVLIYI

KDKLGVGGVRYYKDECIFNVTNKEGIALLISIFDKYNLNTTKHLDFLDFKEAFYLYWN

RKENSDVCLGGASAPYPLDQKQAVYSAEKEKILDLKNKMNTNRVDFDRPNNSPVLITR

SWLLGFIEGDGSFFIRRDTLTPTFAIEVSGVQFPVLVEIKQFLESSLGFDKYSLFKLK

NSSIISVNMNKPRNNSKSSTSIIINNISILNNYFIPFFNETEFLTKKGKDFKDFKLIS

KIIYIGGHRKEDIRSLILKLTNTMNNFRLSTNKEKVQSLTSEEMDLLLKSSPTVERLF

DGRVIDSSTKKILPKLNSCVYEIIGERGDQYLANSLSEAASIINIYPDTLSKYLDIEA

SASNLEVFVDVKTYKVRRVRVLGC"

exon 78438..78513

/gene="nad5"

/number=2

intron 78514..79869

/gene="nad5"

/note="Group IC2"

/number=2

gene 78514..79803

/gene="orf429"

CDS 78514..79803

/gene="orf429"

/codon_start=1

/transl_except=(pos:78514..78516,aa:Arg)

/transl_table=4

/product="hypothetical protein"

/translation="RGCVKGKHTYGGKLSNSGEPLKLLIPNHVWKYMSGWINYSGTVT

SQEICENKMGNRGSKSNKGNTLFVKEQRVDGNWFIKFNLINLRCTLVGFERNSKISYG

LNSFKCLNPCVKIPSNQFGLTKKYSTYSYSPVNPGVWSGLIDGEGSFSIIIDKVQARK

LGWRVQLKFQLGLHAKDYNLLCLLKQDLGNIGSIHLAKNRNIANYSIDSIEDLNKLMT

HFKNYPLLTQKAADLMLFKQAIVLVNNKAHLTVEGLNKIINIKASMNLGLSDKLKSEF

PNFIPVERPIINLDNLNLDPYWISGFVSAEGNFDVRVPATNSKLGHRVQLRFRVTQHN

RDIELMNKIVEYFNAGKIYKYNGGSAVNITIVDFTNITETIVPFLKKYPIIGVKFYDY

IDWCKIHELMVNKAHLTIDGINSIKLIKSGMNTGRES"

exon 79870..81507

/gene="nad5"

/number=3

gene 81608..89870

/gene="cob"

CDS join(81608..81762,83050..83095,84663..84854,86036..86132,

87309..87641,89524..89870)

/gene="cob"

/EC_number="1.10.2.2"

/codon_start=1

/transl_table=4

/product="apocytochrome b"

/translation="MRVLKNNSILKLANSYLIDASQPSNISYLWNFGSLLAICLGIQI

ITGVTLAMHYSPSILEAFNSVEHIMRDVNNGWLVRYLHSNTASAFFFLVYLHIGRGIY

YGSYRSPRVLVWTIGVVILILMIGIGFLGYVLPYGQMSLWGATVITNLISAIPWIGQD

IVEFIWGGFSVSNATLNRFFALHFVLPFVLAALVLMHLIALHDTAGSGNPLGVAGTYD

RIPFAPYYLFKDLITIFIFIFVLSFFVFFMPNVLGDSDNYIMANPMQTPAAIVPEWYL

LPFYAILRSIPNKLLGVIAMFGALVILLTLPYADLGRSRGFQFRPLSKLAFFLFVVIF

LTLGFLGAKHVESPYIIMGQIYAILYFSYFLIILPTVSVLENSLMDVAYLKNINK"

exon 81608..81762

/gene="cob"

/number=1

intron 81763..83049

/gene="cob"

/note="Group IB"

/number=1

gene 82176..83030

/gene="orf284"

/note="copy 2"

CDS 82176..83030

/gene="orf284"

/codon_start=1

/transl_table=4

/product="hypothetical protein"

/translation="MSVFKSKTAAIGWTVEPCFIINLHIKDIELLKKIQIFFGVGSVS

ISGDKVARYRVRSRLDLQIIISHFEKYPLQTTKVINFKSFCKILELLNNKLHTNVEGF

LKILSLINKLNNPLSEALLEKLAPLGKIPVVDLEIYSPEYLDIKAKLNPWWITGFATG

EGSFTFFTRKRVNASGNTVKDYTLAFEIAQRSDSLYVLSLIVNTLGCGKVYSEARGIS

KFRLVPRDLILEDLVPFFEKYPLEGNKALQYSTWIKIVEVLHKNPRSDFRENKVEALI

KELSSLNK"

exon 83050..83095

/gene="cob"

/number=2

intron 83096..84662

/gene="cob"

/note="Group IB"

/number=2

gene 83357..84625

/gene="orf422"

CDS 83357..84625

/gene="orf422"

/codon_start=1

/transl_table=4

/product="hypothetical protein"

/translation="MGLYRYSTISIDNSSDLYNLNNINSEFLSWFSGFTDGEGNFLIT

LDRKYIKLRFKINLHIDDIKVLEVIQSKLNIGRVRFDDKNNNCSYIVEDVSGLGLLCE

IFKNYPLHTSKKLDFENFYEAFLIRKKAKLSDASLNRILYLKDNMNSKREIFSYNTSK

SQIIIDPNWFIGFIEGEGTFGIKTGSSLYFQVAQKNTSQECLNGIINFLHNLSNDTII

PENSKILPLHVTNTINSKTNVVSLVVSNTDSLYYYILPLLDNSKMYSRKAIDFKLWRM

ALLLKIHGYYYSIEGKILFLDISNILNKRYSTKTTVDIDKAVNEVIERFQDILKKDPI

FDVKLYESHVENVRKYSIINKSDKPKTVYIYTEEGLVEGSPFSSFSSAHKTLGLNPSS

NTCNRYIDTNRWYKNKYMFTSKPIDRASRD"

exon 84663..84854

/gene="cob"

/number=3

intron 84855..86035

/gene="cob"

/note="Group ID"

/number=3

gene 84855..85712

/gene="orf285"

CDS 84855..85712

/gene="orf285"

/codon_start=1

/transl_except=(pos:84855..84857,aa:Tyr)

/transl_table=4

/product="hypothetical protein"

/translation="YFTIAQDDYKNLYNSKTIKTFNNKRYYSTSHNSVSNFLASKNIK

PVFVYDNLSEDSVRRDIAKETKDLSGIYMILNKESLNYYIGSASTGRFNSRFTNHLIY

LNGNKVLKNSVKKFGLHSFVFIVLELFPEIVNQENNKKLLDLEDFYLKSLLPDYNILT

EAGSSFGYKHSEVTRIKMKANYSEERRKEIGELNRGKTFSNETIEAMRQSALNRKKIN

STEEAILNMKKRSKPILVKELNNTVYGEFNSIVETAEALNCSTKTIQRTLKSSSKLLK

GRWIINYIK"

exon 86036..86132

/gene="cob"

/number=4

intron 86133..87308

/gene="cob"

/note="Group IA(5')"

/number=4

gene 86135..87022

/gene="orf295"

CDS 86135..87022

/gene="orf295"

/codon_start=1

/transl_except=(pos:86135..86137,aa:Thr)

/transl_table=4

/product="hypothetical protein"

/translation="TKYITVLSISLFILCSVLPTIGTVHKNALKVFNKTLDKNHYLSI

PSPFLSFLVGLIDGDGYIQIGKTTKGFITIKLVISLHLEDISTLEYIKSTLKLGNINV

YKDLKSPTCRLVINRTELQEIFFPLLIHNNIFFLTKTRVNQFNLAMHILKNDIKLFDA

IPSEEKIENVFQLPSSPYDYTLLHFFKNWIVGFTSAEGSFFIKSNNDGCFQLKQRTHT

NLFEAFKLVFNTKRKIDATNTYNQFGVSSKTDIQTVINFFSFEGLHPLTGLKYIQYLN

WLRDLQKSARYNKLNYPKL"

exon 87309..87641

/gene="cob"

/number=5

intron 87642..89523

/gene="cob"

/note="Group IB"

/number=5

gene 87968..88861

/gene="orf297"

CDS 87968..88861

/gene="orf297"

/codon_start=1

/transl_table=4

/product="hypothetical protein"

/translation="MCSPVLFAARSTKGITRLSPAERAEIKLPDEVKEVLIGILLDDG

LFVVVKRWSFLAADILFGVGYLYFRYLLSFLLCFINSKVVYLNPQDSKAIILKENKGK

AGIYLWTNNINGKRYVGSSIDLSARLRNYFNLSYLASSKDIMVIYRALLAYGFKNFTL

EILEFCDPSILLEREQYYIDTLNPEYNILKIAGSRLGVKHTLDTIEKIRTGALNRSKE

ALDKNLEHIKNLNSSLEHKEHLIKLNTSLEHIAKTAHPIEVFDTLNKESQYFRSITQV

AKFFEVHPENVRRHILKAFFR"

exon 89524..89870

/gene="cob"

/number=6

gene 90094..90164

/gene="trnC(gca)"

tRNA 90094..90164

/gene="trnC(gca)"

/product="tRNA-Cys"

/anticodon=(pos:90126..90128,aa:Cys,seq:gca)

gene 90180..90250

/gene="trnR(tct)"

tRNA 90180..90250

/gene="trnR(tct)"

/product="tRNA-Arg"

/anticodon=(pos:90212..90214,aa:Arg,seq:tct)

ORIGIN

1 atgtcaatag aaagatgatt tttatctact aatgctaaag atatcggtgt tctttaccta

61 atgtttgcat tattctcagg attattaggt acagcatttt ctgtattaat aagaatggaa

121 cttagtggtc caggtgttca gtacatatcg gataatcaat tatacaatag tataattact

181 gcccatgcta tattaatgat tttcttcatg gtcgataaat gaagactatt taatctaaaa

241 tttaaatata actctgtgta ataaccaaga caataacatt tctattacag aaaataacaa

301 taattctaat aataacaata acggagtacc taagtacatt aaagtatacg tagaaaaccc

361 ttttaataac agggacctaa agtagcgaaa aaccaaaaag gtgtacgtta cttatcaacc

421 tctagtgtaa tacctgctga agaatatatg gatccttatt gagttacggg atttgttgac

481 gcggaaggtt gttttggatt tagaattcgt aagaatccta atttaaaagt aggttgagag

541 gttattcctt atttttttat aaatttacat tctaaagatc ttactatttt atccggatta

601 gcaaactttt ttggtgtagg taatattgtt gttgtaagta aaaacgattc agtacaatat

661 caagtaaatt caacaggtga tatatttaaa gcaattatac cccattttga taattatcct

721 ttaataacaa aaaaaacgag gagattattt aaacaagctt tagagcttat taaaaataaa

781 gaacatttaa ctttggatgg gattaaaaag ctagtggcta ttaaagcttc tttaaatact

841 aaaactttaa ctgaagattt aaaatctact ttcaaggata ttgtacctga aaatatagta

901 aaacctttat tacctgaatt actaaatccc tattgatttg cgggttttac aagcggagat

961 ggtagctttt cagtagaaat ttaaaaatct tcagcacata aaataggttt tcaagtgcta

1021 ctgaaattca taattactca acataatgta gacttaaact tattaaattc ttttatacaa

1081 tttgtaggcg gaggttttgt aaaagaacgt gctaacgctt ctgaatatag agtggtaaaa

1141 ttatcactga ttacagatag acttatgcct ctttttaatc tttacccgat aattggaata

1201 aaaaagagag attttgaaga ttgatgtaaa attgccgaat taatgaacaa caaggcacac

1261 tttacattag agggattaga taaaatacgt aatattaaag ttggtatgaa taagaataga

1321 aaatttacta atacctcttc agatttttta gaggaaaata caccctcaac aattataccg

1381 caaagaggaa atattgatac tgagattgaa actgagactg aaattgtaac taaaacgggt

1441 aaaagtaaca ctttaatttt tattagtaat aatcaggata atggttatat acctaatgaa

1501 atttatatga ataatataaa ttttactaag gttatagtag aaaatccata taataataga

1561 aattatatac aaaaattaac taaagaaaaa aggggttgct atgtttgaga aagtaataac

1621 catgtatatg tagggcattc tattaattta tataatagaa taagttctta ttttatgcct

1681 tctattctta aaactaaagc acgtagagtt ttacgctatt ttaataaaca cgggtttcaa

1741 gacgcaaatc taactattta tataatgaat gaaaattcta ccttggatga agttgtaaga

1801 ctggaacagc attttattga tacattaaat ccaagtttaa atgtagattt aatagcgagt

1861 agttcaggtt atcacgaacc tatgagccaa gaaataagag aaagactacg taaacaaaga

1921 ggtactcctg tatatgttta cgatgcaaag gatttcactt tatatttgaa tcaaaacaac

1981 atacatatga ttcaattaat attcatcata aaactttaaa taattgttta aatggagata

2041 caatatattt agatactttc tttttatctt tagataaaat agaagaatct gataatataa

2101 atttattatc tttagaataa cttaaagaat taataaataa tgcccgtgag gtatacgtag

2161 ttaaacatcc agcttcttgc tctatattag cagaatttaa agatgattct agtaaaaatt

2221 tattattctt atctttaaat agcttagcta atcatttaaa aggagatcgt aagacaatta

2281 gacaatattt aatgggagaa aagtctggtt attacagagg aaaatgaaaa ttttcatata

2341 aagattaaat agaacggcat ggccgggtaa agtagaaata ttttactttc ttttcactat

2401 ttgctggaat ccccttagag cctttaaaac tagtaccgca gactaaaagt tagcagtgga

2461 atacagtaac atattaaagg attgggcaat cagcaggaaa ccaaagataa ttttattatt

2521 gagtaggatc ctcagagact acacgtgaaa tatcttaata aatactatat ttaaagataa

2581 agatatagtc cgttcttatg cgaaagtata ggagtaaaac gtatgccagc cttaataggt

2641 ggatttggta agaatagaat acaaacttta acaacactag tatcatcaaa tgacgaaaaa

2701 tctggtttaa atttattacg tagtaaatta gggccttatt tagcgggatt aattgaagca

2761 gatggttcat ttgctgtcca tgataagaac tctagagcta aaaaatatgc acctaaaata

2821 attattgtgt ttagtttaaa tgatagccct ttagctgaaa aattagcatc tattactcaa

2881 gtaggtaaaa tatataaaag agaaaatcaa ggttgtattt tgtgaagtat tcaaaatagt

2941 ggagatgtaa taaaaatagt tcatattata aatggctata tgcgtactcc taaaattgaa

3001 gcattacatc gtactatcac ttgatacaat gacaatataa atacagatat tcaaccttta

3061 ggtttagatt tatcacctat tgatagtaat agctgattag cagggtttac agatggagat

3121 ggaaatttta gtataaattt aacagataga aagaaaaaag gtgtggttac ttcaaaaaga

3181 gtgcaagctt ttttccgtat agaattaaga caaaattatc atagagaagt ttcagtagtt

3241 caaggaggtg taagttattt tgaaatacta gataaaatag ctagatactt aaaagttaat

3301 ttatactcca gatctagaga acaaaaagat aaaattttct atgcttacat ggtaatatct

3361 catagtttag aaagccattt aaaaactatg gagtattttg atcgttatcc tctatattct

3421 tcaaaacatt tagcttataa agattgaaaa tctgtagtta aacaaattca acttcgtaat

3481 ggtaaacctt taacagctga aaatataaag gaaatccaaa gaattaaaga tcaatttaac

3541 aataaccgcg tccaatttga tttttcacat ttagatacta taatgaatta aagtttaaat

3601 taagaaattg ccgtgggaga tagtaatatc tctcttatta tgtaactata tgcgggaagt

3661 tcctaaagtt ttattatagg agtcgtgaaa atttttactt gatttaaacg tacatgaccc

3721 ttaaaactaa taaaaataac aatggataat ccgcaggaaa caaagataaa atagaatttt

3781 ttttttattt aggatcctca gagactacac gttatactcc attaatataa tggatgaaga

3841 tatagtccaa atgtaattta acaattacaa ttaaatgaat ttcttattac ctttagtaat

3901 aggtgggcct gacatggcaa ataaaaaagc ccaccttggg attaatatta gaaattatag

3961 tagtaattta aaaaccgata attttaaaag ttatctggct ggtttatttg aaggagatgg

4021 tcatatttga gtacaaaagc ccagtgaaaa aaagaaacaa aaccctagat tttgtataac

4081 atttgggatg aaaaatgaac cattagctaa aagactgtta gagttaatag gttcaggttt

4141 cataagatat aaactacaag ataatgcctg tgttttagtt gtttcacctg taattggttt

4201 aaaaaaaata gttaatttaa ttaatggtga attaagaaca cctaaaatac atcaacttca

4261 taatttaatt gattgactaa ataaaaacca tagtactaat ataaccaagt tacctttaaa

4321 aactagtttt ttatctgaag atggttgact aagtggattc atagattcag atggtagttt

4381 ttcaatcgta cacaccaaat tagaaaatgg tgctaaaaaa agaaaaattg cttgtagact

4441 aagaatagaa caaagaatgt tagatcctat tactaatgaa agttatgaac aagttttatc

4501 tagtatagct aactttctta attgttcatt attaactaaa actcagatat ccacaggaaa

4561 ttcgtattat actttagctg cttcaagtaa aatatcttta aagataataa tagactactt

4621 agagaaatat cctttatttt caagtaaata cttggattat aaagattgaa aaaaagtagt

4681 tttattaatt cttgattgta aacatttaac tgaagagagt ttagtaataa taagtacagc

4741 taaaaatagt atgaatagac agagaacttt tttcaattga gatcatttaa attaattaaa

4801 tctataatta agagcttacg cagctcttaa gtattaatcc cttaacaagg gaatgccgtt

4861 aaggagtgta aattccttaa ttattacttc gctatatgca tagaatctct cgattttggt

4921 actattacca gtcaacagat acatagacac ttaattgaga ttatatatat tattttatat

4981 attaatctat aaagttgtaa ttttcaactg aattaatcgt aacacttgtg tatacatggg

5041 aagaatgtgc aggaaaccaa agtaatttta ttacaagtag gatcctcaga gactacacgc

5101 gaagcccctg ttagtatata aaacatacta ttaacagggt gaagacatag tccgttatag

5161 aaggaaactt ctatagtgta tatattatat acaataccga ttccctagat taaataatat

5221 aagtttttga ttactacctc ctagtttaat actattagta ttttcagctt gtatagaagg

5281 tggggcaggt acaggttgaa ctatttatcc tccattatct ggaatacaaa gtcatagtgg

5341 accaagtgtt gatttagcta tctttgcact tcatttatca ggaataagta gtttattagg

5401 tgctatgaat tttataacaa ctgttattaa catgagaaca ccaggtataa gattacataa

5461 attatcttta tttggatgag ctgttgttat tacagctgtt ttattattat tatcactacc

5521 tgtattagct ggtggaattc tgccagcgtt atatttggcc aattgctgga aagagataga

5581 tattttatct ttatcagcag gatgctttat aattttattt ttatgaagtt tcttcagaga

5641 ctatatgcca aaatttatat gttataataa attatattct acaatctcaa cttttaataa

5701 taattttgat tcaaattttg cttcttattt agcaggttta atagaaggag atggtacaat

5761 tgtaacccca aaagtagaaa gatcacctaa gggtaaacta tattacccta ctatccaaat

5821 agtttttgat ataagagatt tccctttagc ccaaattata caatctaagt taaaacatgg

5881 ttctttagct cggaaaaagg gtactaatgc ttatatatta actataaata gttttgaagg

5941 tatgatttta atagttaata ttattaacgg ttatatgaga acacctaaaa ttatttcttt

6001 atataaatta attgattttt taaaccataa atttgattta aaaattgaga aaaaagataa

6061 ggatagtagt tctattggtt ctaactcttg attagcaggt tttattgatg cagatggtca

6121 tttttcagtt agaaccacct tagaaggtaa atatcctaaa atagaatgta aatttgaaat

6181 ttcgattttt tgtccttata atgatttata tattgaaatt tgtaaaaatt tgatgaaggg

6241 tattgcaact tttttagaaa taaaagatga tagttttaaa caagtaaaaa catctaaagg

6301 ttttgttttt tctgttagga cacaaagtat taaaaataat gaaattctac tcaattattt

6361 atcttcattt cctttatttt ctagtaaata cttagattgt caagatttta gtttaatctt

6421 tgagatacta aagaaatatt ataaaacaag acttgagtta gataatgtag atttagaact

6481 tattaataca cataaaaaga gaatatacca aagaacagaa tttatttgag atcatcttgg

6541 aaatctttat aacggtttag atactacaca atttagaatt tcaggtttaa gaatcaaaga

6601 ggaatttttt acaataaata ctttaaagag ttcaggtttc actagagggt tttcttcttg

6661 ttcctgttta aatacacgtt caattaatga taataattta aataaatctt gtttatatgt

6721 ctcatctgtt atatatactg acatgttatt agaaaaaaaa ttattgttag atgataataa

6781 agaaaaatca ggtatatatt tattaacaca tttagaatct aaaaaaatgt atgttggttc

6841 tagtgcagat ctatatagac gtttaaaata ttatttttct aaggttaatt taactcgtaa

6901 tacaaatagt agaatataca atgccttact tcattatgga cattctgctt tttctttaac

6961 tatcttggaa tatgtaaata ccagtggatt aactaaaagt gaggctaaaa atattataat

7021 agggaaagaa caattttata ttgatctttt aaagcccgag tataatattc tgaatacagc

7081 tggatcttta ttagggttta aacactctta tgatacaatt cttaaattta aggaagctaa

7141 aaataaagaa aataatccta tgtttagtaa acaacataca gaagaagcta tattaaagat

7201 gagattgcgt agattaggta aattattaac agaagaaact agactaaaaa ttggttctac

7261 attaagtaga aaagtcttta tatttaaata cgattctatt tcagataaaa aagtctttat

7321 agcaaaattt aacaactata gtgaggctgc acaatacttt aattgcagta ttcgtacttt

7381 atctagatat atagataagg atatattata taaaaaaaat ttttatattt acactaaaaa

7441 tattagtaaa tagattattt tcacttctcg aggatattgg gtgagcagag aaataagata

7501 atatacatgg atatgttaag ggtagggagg cggaccttgt tatctcaaag acaaaatgat

7561 cataataatg aaaataactt tgaattttta aatttaattg cagacttttt aggtgctaat

7621 gttaaagaga ttagaaggta taaacctaac cctgagtata gagttagaac tactaactta

7681 aaaagtaatt tggtgcttgt gggctactta gataaatttc cgttattttc aagtaaatac

7741 ttgaattata aagattgaaa aaccatatta aactactttg aagctaaact acatactaat

7801 cccgatacta tacaaaaaat agtggatata aaagcagaaa tgaataataa tagaactaat

7861 tttaactgag atcatttaga atatttttat aacttatata aataagagat agtcccaaca

7921 atatggtgac atattgagta tttaccttaa gcgacgtggc ttatgagatt taataattaa

7981 ctttaattaa atcatgagac ctggtctagt aaatcaagaa ttcagggaat tactatggtt

8041 ttaacagata gaaatttcaa cacatctttc tttgaaacag ctggaggtgg agatcctatt

8101 ttattccaac atcttttctt aacaaaacaa acttacatta ttatatttat tttattaagc

8161 agtcttactt tatacactgt gtataacagt acccttagta cttttaattt taaatctttt

8221 tatctagaat attctaattt atatcctaac tgtaaactac ccgactctaa atttttagaa

8281 tgattcatag gatttactga aggggaaggt tcttttacag tagctaaaag aggagactta

8341 tcatttgtta taactcaatc tactactgac atagaatgtt taaacttcat taaagataac

8401 ttagggtttg gaaaagttat taagcaatct ataaaaagta atacacatag atttgtagtt

8461 caagatatta aaaatttaat tcttatttgt aaattattta atggtaatat ggtatttcca

8521 actagaaatg ccaaattcct catttttctt tctgctatta atgaaaaatt attaaaaaaa

8581 aatatgaaca ctatatctat aatagataat acagtattac cttctttaaa tgatggttga

8641 ttgtctggta ttacagatgg tgaagggtca tttacttgta gtattttatc tgaacctaat

8701 tccgggtata gatttagata tatattaact caaaagtgag atagtaataa gcctatcttg

8761 gaatttataa atagattatt tggagataaa gtcggagcaa tatattccca tcacgaatct

8821 aaaaataatt ttaatatatg agaacttaga ataaacggag taaaaaactg tgaaagttta

8881 tttgaatact ttgatacatt tactcttaat agtaaaaagc gtcttagtta tattaaatga

8941 aaagaagttc atagtaagtt aaaaaacggt gaacatttga tttcagataa aagattagaa

9001 ataaaagact tatgtaaaaa aataaataaa taatagtgta tttttcagga aaaaagggga

9061 ttggtttagc caaagctatt aaattaataa agcagatgtt aaacaaatta agactttaat

9121 taatgaatac gaaagtatac cacaacccta gtttttaata ataagattat tgtgtaattt

9181 ttaggaaaaa acaataaata ttaaaagcaa cactagtggt acggtcaaaa ttagccataa

9241 caagaccgtc gtttacctaa atgaacgcta cagactgcct cactggtggg tggctgaaat

9301 gctgcttaat gtacagtcgg attcctcttt taatttgtcc aaaaaggctt aaatcttgta

9361 aatatgggat ctaaggttat atagtttacg gttgaaataa aattatataa taaggaattg

9421 gattctttgg acacccagag gttaaatttg taagcctctt aatgttgctg tatgctggaa

9481 acacttcgct atatagtttt aaatactccc tcttaattga cacagtaaaa aagttaaaac

9541 aatgaagtca atcagcaggt aacgttttta attttaaaaa tggaacctca gagactatac

9601 gcaacaatgc tgaagctata aaaaatattt ctattcatgt tcctgaaagc cgttaaatga

9661 taaagaattt gacactactt agctggactg attgatggag atggacattt tagttctgca

9721 caacaattag taatagtttt tagttttcca gaagttagcc tactatatta aagaaacaat

9781 aggttttggt aatgtaagaa aagttaaaga taaaaatgct tatttatata ttatatctaa

9841 taaagaagga ctattgaaag ctataaattt aattaatggt aaattaagga gtattaataa

9901 atttaatcaa gtaaatgcta tattatctag tactaaatat ttaaatgaaa gtttagtatt

9961 taaaattaat gattccaatg attttaataa tcattgaata gcaggatttt ctgatgcaga

10021 tgctagtttt caattaaaaa tagttaatag agataatagg attaaaccag aaattagatt

10081 aaactttcaa attgaccaaa aaatatgcta ttattaataa aaaatatatt tggaggtaac

10141 ataggttata gagaagctca aaatacttat tattatggat caactagctt tggttctgct

10201 aaaaaagtaa taaattattt tgatgtattt catctacaat ctagtaaaca tataaattat

10261 ttgaaatgaa gaaaagcata tttaatagta caaaacaaag atcatttaac agaagctggg

10321 atagaaaaaa taaaaaaatt aaagtattca atgaatagtt attcagcata agatagagtc

10381 ctaacaatga cggaagttat tgagtattaa ttttaatgga tcatattaga ctattatatt

10441 aatcaaagta tttgttacat attaattatc cctgcttttg gaataattag tacaactatt

10501 tctgctaatt ctaataaatc tgtttttggt tacataggta tggtttatgc tatgatgtct

10561 ataggtatat taggattcat agtttgaagt tgagtaacga tggcttcacc ctatagtgat

10621 atagggatta caattaattt cgctatatgc tggaacagtt tagtgctaat tagtcccttg

10681 tacggtaaaa atctaattag ttatactcaa tcagcagaca atctatccct gtattcctta

10741 aaggataaca aacagagcgt cccagagact acacgcgaaa catctttaaa attttctaca

10801 tttcatgctt attataatac attatttaaa aatagaaact ctatttctga tgattgatta

10861 acttgattta ttgggtttgc agaaggagct attcaaacct atgataacgg taagagagtt

10921 cgttttgttc ttactcaaag tgatatactt aataaaatac aatttaaatt taacattggt

10981 gttgttaaac attttcctca aggtaaaagc ggtaaaaata atgattttca tagatgaatg

11041 gttgataacc cttcagatat tttactttta gctcatttat ttaacggaaa tttagctcaa

11101 agtcacagaa ttgaacaatt agctttatga gctaatgctt taaataatcg ttttggttct

11161 gatactataa agttaaataa tactcctgct acgattacac tacaggatgc ctgattatca

11221 gggtttacag atgctgaggg gtgttttaat gtatctatta cagaaaactc tagatataca

11281 ttagggcacg ttataaaaat gcgttactta ttagatcaaa aagatagtat tatactaaat

11341 aaagtatatg aattatttgg atttggtaaa gtgacattaa gatctggtac tgataatgtt

11401 tatcgttata ccgctactgg gtttaaagca ttaaatgata taatagcata ctttaaatta

11461 tttccattaa aaactaaaaa agctatttct tttgaaaaat ggttaactat tcataaccaa

11521 gtacagaata aattacattt aactgaagaa ggattatcac aaataagaac tatgcaaaaa

11581 aaaattaatt taaataatag catgacaaat aaaactggaa aggcttaaag atgaagatat

11641 agtccgatac ttcttgtgaa agaagcatac ttaattgtat gggtaaatat aagatattta

11701 ttaacatttg catcacatgt atacagtagg tttagatgtg gatacaagag cttatttcac

11761 agccgctaca ttgataattg cagtcccaac aggtataaaa atcttctcat gattggctac

11821 ttgttatgga ggttctatta aattaactcc atctatgcta tttgcattag ggtttgtatt

11881 tatgtttact attggtggat taagtggagt tttattagca aatgctgcgc ttgatactgc

11941 cttccacgat acatattacg tagtagctga agtgggcctc tataatctac ccctagatta

12001 ttttgcaact gactatatgc tgggaacctt gggttgatgg tattatttac tcattattaa

12061 aacttttagt aatagaaata aattttcttt taatagtaaa aaaagtaatg cgagctcgaa

12121 ggtagtaatt taccctaact tatcagcaga aaactgtgag ggattctcag agactatacg

12181 tcagtcgcta aatagagtta atttttgaaa tagatttgca agtgtgttag acactaaagg

12241 ctattttgac tttattagtt ttaataaaaa aaaaattctt aaacaaatta taattaaatt

12301 agattataat gatattagaa tattaaaata tttacaaaat tatacacatg taggtagaat

12361 tataagtatt aaaaataaat ctatgtatat aattgaagtg gaagacgatt taagatatat

12421 tttaaataat cttaatggct ttattagaat taaagtccct gaatttaaag aagcttgttc

12481 ttttttcggg attgaattta aagaagctaa ttacacaata aaaaagaatg ataaaactta

12541 tttttcaggc ttaattgatg caaatggaaa aatagtattt aatcaagatt catgtattat

12601 tgaatgtaat ttaatattaa aaaataatga atatataaat aaattgaatt taaatcatgc

12661 tatacctaga tctaaaccta ctaggattga aaatgccttt ctatttcaaa aatttcgtga

12721 tatgtcatct atctataagt attttatgaa aagtaaacta tattcaaata taaaattcta

12781 tagagtaaat aaaattaact attatttaaa cattttcctg aataaaagta accttaaata

12841 taaaaaaaag tataatgatt cttattattt tttaaaaaat tgaattaaat ataataacat

12901 ttattgatat aaaaatactt atgtaaaaga tatatacatc taaacggtgt atgacagact

12961 ctatttagtg aagatatagt ccaattgtta ttctaagatc cactatttta atctaaaagt

13021 gataaatatt tgagtggtga atacctaatt atcccttttt aatggtattg agtgttaatt

13081 cttcttttta aattttgatt ttttctcttt ttacacttat tagaataaca cctagggtcg

13141 tatacccatt atgatgtttg gtataacagg gatccaatat catataaata tattttttgc

13201 atactctacg gacaaatgta tccgaactcg ggatgctact ttttgacgtt gtatatttaa

13261 aggttataaa tgataagtta gatttaaagg ggagtcaata ttttgaaaac aaatgagatt

13321 ctaaattaaa tttattgtat aatacaatat acaattatac aaaattaata aatccttttt

13381 tcttactttt atctgtttat tttattaatt ttttctttgg tgatatcaat agtatatatt

13441 tctatacttt aaagctattg ttacaattta gttttttgat taatttaatt tatctaaata

13501 aaatttgaat tgatcaaaaa attattaaga atgattttcc tgtttttcat atgttaatta

13561 aattaatatt aggtggttgt cttatataca atgttgctgt tataatatat actttgtatg

13621 taatatgtgt attaatatac aattatgtat taaaattcaa cttttgaaat aagataaagg

13681 gttgaagatt atcttttgag tataataagt ttaaagggcc caatgaccca aaagactcta

13741 ctctccttta ttctgaagac aaaaagaaga ataaaaagac agcttcagag ttaaaagatc

13801 ttatattgaa agcccaaact aagaaagcga aatctaattt aaatccagac ttaaaattag

13861 aaagttcaaa tttcaataga aattgggaaa aaaaaatcaa tatagataaa ctaccagact

13921 tctctattga ggatcaaata agaaacctcg aaaaagaata tgttgcatat gacttacaag

13981 aaaaaaagtt caaggatatt gtggttaaca ttggaaaaaa aaagaagaat tttatccttc

14041 cgatgcatca aatttattta aagaatatgt ggatctagta catattctta aagttaacct

14101 aaaaaatagc cttaactcat tgaagaagta tcaaaaaaaa aataaataaa ttaaaacctg

14161 tcaaaaagca ttaagcattt ccattatgtt ctaagtatgg gggcagtttt cgctttattt

14221 agcggatgat acttttgaat acctaaaata ttaggattaa gttataatat tttactatcc

14281 aaagttcatt tctgggtatt atttatcggg gttaatttaa cattcttccc ttgaaaagtt

14341 gggggcctac aatagaaata atgtagcgta aaaaaaataa tttttttgaa aaacttggtt

14401 aattgctgga aagatataaa aaattaagca ttattattac ttatgtccaa tcagcaggta

14461 agcttccttt atgtaaagtc aagcgacttc aacgaccact tgccaagtac ctaaatttat

14521 atgaattaat tatataagta tggtagtgat atggtctaat attagtgact aataagcata

14581 gatagtttat ccaaactaga atcacatcgc tccgtgataa aaaataatat ttattctagt

14641 ttaaagatcc gtagcttgag tcctaatatt aaatatacag atatatctaa gcgtttttat

14701 tctactcatt taaatactca actaaccttc gcggtttcac cgtctgaaat tgatgactta

14761 attaataatg aacctattaa atttgattca cttgatgagg catgtgagct aactaagttt

14821 aaatatttag gagtttcagg ggtttatatg ttaataaata aaaaagactc aaaccgtttt

14881 tatatcggga gttctactaa tttagctaga agaatggaag aatataataa attaactaaa

14941 ggtttacgta gtcctcgttc tagttcagaa ttagagatat ctaaaacttc agctttagat

15001 tgaaaactag agtttatata tattactact cctcaaactt ctttagtttt cgagcaattt

15061 gccataatta aaaataaacc tactattaat gggtttatcc aagttattcc aagatttaac

15121 ccccagtgag ggaaattaga cggtgctatt gttaccatta aaaatttatt atcgttattc

15181 cctaaatgtt cagaaggtta taatagatta gctgcgtttg attcggttta tagtgcagct

15241 aataacataa aatatacagc tgaagatcta gacaataaat attattgttt tttaatatat

15301 gcttatgact taaatttacc tgataaaaat cctgttattt attcatcaat aaatagagct

15361 ttaaaaggat tacagattag tcatagtact ttattaaatc atatttataa taaatatatt

15421 tttgttacgg aatcaaacac gctattatct tttgaacctt tatctaagga ggaatttttt

15481 gaataccaag aaaaacctgc aggagataat caattacgta aacacatcga agtctttaat

15541 caagataatg aattagttgt aagttttaaa tcgggtagag aaatggctag atttttcaaa

15601 attgatggta aagtagcaag agctgctatt actaatggtg aatatcagga ctttttactg

15661 atatctaaag aagtatctaa tcgtaaaact atttatgtat ttgatagtag ttctcacgaa

15721 ttactagaag aaattaaagg tgtatctaaa gctttgaaat atgctaaagt taatttttat

15781 acattaaaaa gtttaatcga aacaggtaaa tctcagaacg gtaaaatata tagctacgaa

15841 gataaattat aaccaattta ctatgcgtac gcactaatga ataatgcaac atttccttgg

15901 tcaagtaggc cctttatcat aaaaatatga tatttgcaca acactatatg ctagaaactc

15961 tattatatta aatataataa atagacaatt agcaggaaat cttaataata tgaaaataaa

16021 aattatatta tatgtagaat cctcagagac tatacgtgtt gcaacttaaa agttgaagaa

16081 atagtccaaa taatatagaa atatattaca atatatctta ctgcgcataa ttttatatta

16141 ttgtaacaaa attaaaccca tttagcttat tttacgcata tcgataatta attgttaaga

16201 ctttttaagt aataagtttg attgaaaaat ttaaacttat tataaaaata atttaatata

16261 cgatatataa taatacctta caattacggc tcttatagaa aattaatacc tatatgtata

16321 aaaggaatga aaatattaaa aaataaaaat aataattttg tagaacaaga ttgagataac

16381 gacgacttag tagaaaaaac cattaataat aataatttaa ttactatcaa acctgaacat

16441 aagtttagtc aagaatgtaa attaaattta gttaatttaa gttctactcc tgattctcct

16501 ttatacaatt ttttattcga taaagaaatt attttaaagg aatataaaaa taaaagcggt

16561 atttatctat tacacaacaa tgttaatgga aaagaatatg tagggagtgc taaggattta

16621 agtaaaagac tagctactta ttactttcct tctcgtttaa tagacagtcg ttttatttct

16681 aattctattt taaaatatgg acatgataac ttttctcttg ttattctaca tgttttaggt

16741 gatagtaact catcctctaa atcggatatt ataagtaaag aacaagaata tattgattta

16801 tataaaccta ttcttaattt aaaccctgtg gcgggatcta gtatgggatt taaacactca

16861 gaagaatcta aaagacttat atctgaattt cataaaggta aacctttatc agaagatact

16921 aaaaaaaaac ttagtcttca ggtgaattaa acccattttg atctaaaact cactcacctg

16981 agacactaga aaaaatgagt aactctaagg tgggtaagtt aaaccctatg tttaataaag

17041 aaaagtctaa ataatttatt gaacaaatgt atagagataa aagtggagct aataatccta

17101 tgtttggtaa accaaaatct gaagaaactt tagcaaaact aagaaaaaag gtttatgttt

17161 atgattacaa caaacaattt attaaatgtt acgatagtat agggtttgca gtaaaagact

17221 tacatattgc agcagaaact attaagaagt atttaaatac tgataaaaag tataaagata

17281 aatactttta ctctagtttg caagaataag cgagatatat attgctacaa ggtatgccta

17341 gaagaattag tgattaccct gacgcattta caggatgaaa tctagttagt agtttcggat

17401 ctatagtgag tgttatagct acagtattat ttttatacat tgtttacaaa caattaatag

17461 ataataaccc tgtaggtaga ttcccttgat taatgcctca atattttact gacgcattac

17521 aagcactatt aaatagaaat tatcctagtt tagagtgagc ccttacaagc ccacctaaac

17581 cacacgcatt tgtaagctta cctttacaat cttaaacttt taggctttga agtcttctaa

17641 cttttttagt aaaattttac taaaaaaaaa atctaaccta ttttattatt gatataatat

17701 actataatat tattatcttt ttcataaaat aaaaaaaaat catatatata tatgttacgt

17761 tttcccactc ttgtatctat aatagaggtt gtattggtat tagtgccagc tctacttgca

17821 gtagcgtatg ttacagttgc agaaagaaaa actatggcaa gtatgcaaag aagactcgga

17881 cccaacgccg taggattaat aggcgttaat ttatcatctt tttctttcaa aattaattca

17941 tctttattta atagcaatgt acgttgtttt cattcttcta gtcttaaata taaaactaaa

18001 gacctggtat caaaaaactt agatacaata caagcaaata ctattaaaag cttaactgaa

18061 ggaagagttg caccaatcct tccatttaat tatccagttt taataacttg ttccaatatt

18121 ttaaaccaaa aggataaatt agagtttttt tagtaaactt tctgacctaa ttatagcagg

18181 aaaaagtaat agtttaggtg ttatttatat atttcaatat aaatttgatc ccaatttatt

18241 ttatattggt agaactaata acttaaaaac tcgtttaatt aatcatgttt caaaatataa

18301 atttgataaa ttccatctta gcgctaaaat attggggtga gaaaactttt ctttaagtgt

18361 aattgaaata aaagaatatt cagatttaat tcaaagtgaa aattattacc ttgaactata

18421 taaaccttta ttaaattcca tattccgttc tttttattct aaagatattt ataaaaaagt

18481 tagtcttatt gaagagctta tacctaaaaa tttaagtgac actaacctta atgcagatga

18541 tttaattaat aacccttacg aaattgatgg tgacgatatc cttttagagt ctgttaacta

18601 caactcttca gaaaaaggta aacttaggtt tccagtatga gttatcgaat gattagataa

18661 tgagctatta gaagatagta ttaaaaatta tcctaacaga caaatagtaa gtgaaataac

18721 aggtgtggct gtacaaacta taaatcgtta tttaaacact aaattaggta gcgacggaaa

18781 atattattta ttcttttctt tagaaaatat agttaaagat ttaacttcta ttgatgttca

18841 agaaaatatt atcttagcga aatctcttaa actaagattc gattctaatt tagatagaaa

18901 agtttgagca tatagtgtag atgaagccgg attaatttat cttttagcag ataagccttt

18961 ttaggtgaaa caggagcttt ctttttaact tcttctgcca caattttata ttatttagat

19021 aaatttaaaa tttacaaaga ctactattta tttagtaaac ctttaattga agaagataaa

19081 aatatattaa ttgaaagaca tatgcttagt aagtcggata tttataaatc tatgggtatt

19141 aaaacaaaaa tttgagttta tgatccgaat actctaagtt tattaaataa tacttacttt

19201 agttctatcc aatcggctat ggattactta aaaactagcc ggagatctgt tttaaaatat

19261 ttagattcag gcttaatctt aaaatcaaat aattccctat tttatttatt tagtcaagaa

19321 actaatagag aaaaattaga aatgttaaat aacaatcctt tgagtaaatc tttagtaaaa

19381 tctgatgtat gggtatatag taattcttct gggactttag ttttattaaa cgagaaccta

19441 ccgtttagct ctataaatcg tacggcggtt gcgttaggtt tatctagcca tacaataaaa

19501 aaatatttag ataaaaatat ttcatttaaa aattattact tttatagtaa taaacaagat

19561 aacatataat aaaaataagt agatttatta taagaggatg gaaggtggga tatatgttaa

19621 tctaaccaga ctagataatt aaacaatagt ataagtagct aactttaaaa taaataagtt

19681 aaacgacggc tacaattaaa ttatactaaa cgtacgttgt atattataat ttttaagtat

19741 tacagaagtt aattttaaga aagaggcgtg cattaagttt ttagtcccct ttataattat

19801 tgttataaag aaaacagggg atgaatttca agaaaaactt ttaagttaac ttgaagcgaa

19861 gcttattgtt aattatcagt tgtataaata aaacgatgaa tagcaatttt tataagaacg

19921 ttcaacgact agaaataatt tatttcatag cgtcccccat cttacagatt ttaatgcaat

19981 ttaatatcct aattgtgtta tacggtagga tgaagacata gtctgaacca ttttgtgaaa

20041 ggtggaaaag gtgcacatac tttgtatgct ttgcccttat gttaacataa ttgattatgg

20101 gctattacaa gcatttgccg atgctttaaa acttatttta aaagaaaata ttgcacctaa

20161 tcaagctaat cttactcttt tctttctagg tcctgttata actttagtgt tttcattatt

20221 aggttacggt gttatacctt atgttcatag ggtgatatag tcaaactccg gggaactcct

20281 aaagcttata atactaaacc aggttcgaaa gttcttgagt ggctgaagta attactcagg

20341 tatagtaaca agttataaga tgatcgtaaa gagaaatgga caatcgcgga tctaagtcaa

20401 attcaaggga agcatgttac ttttattcta cgagttaggg taagttaagc tatatgagct

20461 ttcactgcat ttgtaaaaga gcaacgagta gacggctata ggtatttact gcgctgctaa

20521 cttttagtca gcgctgcgca agcaagctac agagtttgat attcagccgt aagtatttaa

20581 ggtgtattct aaggggctta gaaatgagtt ctcataaaaa ttgtatgcta attattataa

20641 cgattcacca ttaattggtt ttaattcttt aaatattaat tctcgccgga gttattccac

20701 atttaataac aataaatata aacaaggcaa gtcacaaata gacccatgat ttataaccgg

20761 gtttacagat gctgaaggtt gttttacatg tagcatatta aaaagttcta gttacaaatt

20821 aggctgagaa attcaaccag cttatcaaat taaactccat gttaaggatt atccactttt

20881 attagatatt caacatagtc taggtaatat tggaactgta actagtagcg gatctacatg

20941 tacttttaga gtcagaaaat tgaaagagct tttagaatta attaaatttt ttgataatta

21001 tcccttggtt tctagaaaaa aaggggatta tctaatgttt aaagaaattg cttctattat

21061 gcaattgaaa gaacatttga ctactgaagg tatacaaaaa attgtcaact taaaagcaac

21121 tttaaacttt ggtttatcaa aagaattaca attaatgttt cctgaaacta ttccggttgc

21181 ccgtccttta agagaacctt gtgtgatccc acattcacaa tgaatagctg gatttacagc

21241 aggtgaaggt aatttctctg tttctttaga taagggtgta ttcaagtctc ttttatttaa

21301 aattactcag cataaaagag atgaggtctt attaatggca attaaagatt attttaattg

21361 tggaaattgt tatttaagaa aaatagaata cacaatggat ttaaaagtta ccaaattttc

21421 ggatgttact gatataatag tacctttctt tattaaaaat tcgatattag gtgttaaatc

21481 tttagatttc aaagattgat gcttagtttc agaaatagta aaaaaaagag aacataaatc

21541 agaagaaggt atcaaaagaa tccaagaaat tcaaaagggt atgaatagag gaagaagttt

21601 ttagtattac tttctttttt ttttgtatgg accaggttta gctattagtg atttaagttt

21661 aggtatattc tacctattag cagtatcttc tttatctaca tatggtatat tattagcagg

21721 atgaagtgct aatagtaaat atgctttctt aggttcttta agaagtacag ctcaattaat

21781 tagttacgag ttagttttaa gttcagctat attattagta gtatttataa caggaagttt

21841 aaatttaact gtaaatatgg aagcacaaag agctgtttga tttatattac cattattacc

21901 tatatttatt atattcttta tagcttctgt tgcagaaact aatcgtgcac cattcgacct

21961 agctgaggct atccatacaa gctgtacaac tatagctaat cattatatta caaccgtatt

22021 tattccaatt tatatattca tagttattga ttttattctt aaaattaata tgagtatttc

22081 caatattttt agtttatttc gttcattttt cgaatcatac tacactgcca ctcccctttc

22141 gatgccgccg aagggggggg gggggggggg gggggagaga gataaacttt caaattttca

22201 gaagatattt tagttcctca attatcaaag ataatcccct acaccctcaa tgaataactg

22261 gtttcacaga cggggaaggt tcctttggta ttaagatatc taaagctaaa gaatacaaac

22321 ttggatgaaa attacaaccc tttttccaaa ttaaattaaa tgtacgagat gtagatatat

22381 tacaccgtat ccaggaatat tttgaaggga caggaactat aacttttgag aaaaattttg

22441 ctaagtttac tattagaaaa cttggcgata ttgtagacat tctgatacca cattttgaag

22501 cttatcctct attaactaaa aaatttgcag atttcgaact atttagacaa attattttaa

22561 ttctaaaaaa tgaatctcct ttaagtgaac aaggatttat agaaattctt aaccttagat

22621 ataatctgaa taaaggaata tccgaggaat tgaaagaatt atattctaac ttagtaccaa

22681 aggcaagacc tgaagtccca gaatgagaaa tccagccgga atgattagta gggttcgtag

22741 acggagaagg tagttttaat ataattacag tagagaaaaa ttcttcaggt gtaataccaa

22801 tgtctgacac tagttacaaa gtatgattat attttcaaat aacacaacat aatcgtgata

22861 ttgctcttat ggaaagaata gttacattct ttggctgtgg aagtgttaaa aaaagaaaca

22921 ctgatgccgt cgactttaag ttaaataaat tcgagttgtt agaaaatata attataccct

22981 tttttcaaaa gtatccgctt caaagcgcta aaaatttaga cttctctggt tttgttgaag

23041 cagcggatgc aattaaaagt aaaacagctc ggcaatgaac aacagaacaa ttcgataaaa

23101 ttaaaaatat tcaaaataat atgaataaat acatcaaata aatttcaaaa gaaaaaggtt

23161 acaaaaaata ataatctttt aaactatcgc ttaattacta ataataataa ttaagcttta

23221 tctatctttt gttaattttt gattcatgtt aagataatat aaaaacgatt tagctatgag

23281 gatttggcct cattaaagat cacaaattgc tggaaatctt aattaagaca atcagcagga

23341 aagcttcttt gcgcatataa gctcaaagtt gaatcctcag agactagagg tgatcgttta

23401 atattcaata ttaaatatgg tatagtccaa acatctacgt aagtagttga ttaatactat

23461 ttaaagttat acacttattt taaatgtgtt tcatccccta aatttcccct tataaagtaa

23521 ctaaaagatt ttattcttct agtcggcttc caaatattaa tccagtacct gttttagttt

23581 tatataaatt agcagataaa tcttatatta attcatttag aaattcatta aaagataaag

23641 gaggtattta ctcatttata aatacagtta atggtaaaca atatatagga agtgcaaaag

23701 atttatatct tagattaaat gagcatttaa ataataaaaa atccaattta cttttgcaag

23761 cagcttttaa taaatacggg ttagataaat ttaattgaat tgtttatgag tattttacat

23821 atgaaaataa aattataagt aataaatctc ttactgactt agagacaagt tacataagaa

23881 gttttgattt ttctactctc tataatttta aagttgaagc tactagtatg ttaggatata

23941 aacatacaga tgaagctaaa caaaaaatgg tagaaagatt taaagatatt actaatcatc

24001 ccatgtacgg taaaaagcat agcgaggata tattaaaatt aataagtaaa ccaggtaagt

24061 taaaccctat gtacggtaaa acccatagtg atgaaactaa aaaagccatt tctataaaaa

24121 tgagcaaata tcctaacggt gtaggtattt ttgatttaaa taataattta attaaaaaat

24181 ttaacaataa tactgagata gccaattact taggtatatc taaagttact gttgctaaat

24241 atttaaataa tgatttaata tatcaagata tgtataaatt taaacccata ttataataaa

24301 tgtataactt taaaattttt ttggaatcag agcttgttag tgggtttatg acagaacatg

24361 ctgctgttgt atttgtattt tttttcttgg cagaatacgg tagtattgta ttaatgtgta

24421 tattaacaag tatcttattt ataggaggat atttaattat aggtagttct ataatagata

24481 taagcttata tatctatgat tctaatttta tagtaaatta tattataaat agtccaatta

24541 tagaaggttt attatacggt ataagtttag gtattaaaag ttctataatg atatttgtat

24601 ttatttgaac aagagcttca ttccctcgta taagatttga tcaattaatg tcgttctgtt

24661 gaactgtact actacctgtt ctttttgcaa taataatatt aatacctgct atattatata

24721 attttatatt attccctatt aatattagtt tactataata cagtatgtat taattaatac

24781 cataagtatt cttatatatg gtgtatattt tcagaataaa ataaaatatt ttaggttttt

24841 atactaatag caaatataaa agagaatgtt attatctttt ttattactag tacctattgt

24901 aggtattttt ttaatcgcag gggctatatc ttatcaagat aatgttataa aaactacata

24961 ctacaaaaat atagctttaa ttacatctat agttaattta attatttctt tagtagtcta

25021 tatgttattt gactctaata caaatcaatt tcaatttgtt caagagcatt ataatctaag

25081 ttattttgat atttatctag gagtagatgg gatttcaata tattttgtat tattaactac

25141 tataataatg ccaatagcta tattagctaa ttgaaactct ataactgata atataaaatc

25201 atatttaata ataatgttat tattagaaac actattatta gcaatattct tagttttaga

25261 tatattatta ttctatattt tttttgaaag tacactacct cccttatttt tattagtagg

25321 tttatttgga tcaaataata aagttagagc aagttattac ttgtttttat atacatttaa

25381 ccttaagtgt aaaagagcca aacaccgggg aagccctaaa gctctagtaa ccaaagcttt

25441 aaaagaaatt ttaaagctgg cctgattaat gcctcagggt atggtaaaat cactagtttt

25501 ctatttagaa aagttatttt atataacaga tatgtgggtg atcgcggttc taagtcatct

25561 tttttttaaa ggtgtaaaag agcaacgagt agatggttct tcagtatcta gtaatttaga

25621 tattgtaagg tgtactctag tcgctgggaa accagtttta agccgaaaaa tttatacaga

25681 ttataataaa agtataatta taaataaaag attttcatct tctaagtcaa atttaaatcc

25741 ttgatttgta acaggtatca cagacgctga aggatgcttt atgttaggta tttttaaaag

25801 taataattac aaaatgggat atcaagtaca aggtatattt aaaattactt tacataaaaa

25861 agactatgat ttattatctc aaatcaaaga ttattttaag gtaggaacaa taacaaacca

25921 tggagacact actttacagt atactgtaaa atctataaaa gacttagata aaataatata

25981 tcattttgat aaatttacat tgctaagtca aaaatcagta gattatcttt tatttaaaaa

26041 tggtattatg ttaattaaaa ataaagagca tttaagttat gagggtttaa aaaaaatcct

26101 atccttaaga gctgccatga acttaggttt acctgaggaa ttgaaattta attttcccga

26161 tgttattcct ttttctagac cttctacgtt aagattaaac agtatagatt ataactatat

26221 atcaggttta acaagtggag acgggtgttt ttatgtttcg atacgtaatg cacctagtac

26281 aaaaacaggt aaatctgtaa tattaaaatt tcagattgtt caacacagta gagatattga

26341 attaatgaga aagttaatct ctactttaaa ttgtggaaga atagaattaa acttaaaaca

26401 atctgctgtg tattttgtag taacaaattt tcaagatata gttaatcaaa taataccctt

26461 atttgataaa tatcctatta aaggagttaa atctttagat tatgcagatt ttaaatcagt

26521 agttaactta atgcaaacca aagaacattt gactcaagaa ggtttatcta taattcaatc

26581 tttaaaattg aatatgaatt tatttagaaa attataaaaa ttattagact tgtcggttag

26641 ccgatataaa ttgtagtaag cttaaattaa agaaaacatt aatcaaaatt agtgaacgta

26701 tgaggttctt tattcttatt aataggaata ttagctatat cttctataat ggggactact

26761 gatttcgatg ctttatttaa aactaatttt gattatatta ctcaattagt tctatttagc

26821 ggaattttct tatcttttgc agtaaaaaca cctactattt ttttaaatag ttgattatta

26881 aaagctcacg ttgaatcacc tttaggtgga agtatagtac tagctgcaat agttttaaaa

26941 actagtttgt acggtatatt tagattaatt ttacctatat tacctaaagc atctattaat

27001 tttacttata tagtatatgt tataggtgtt attacaataa tatacgctag ttttagtaca

27061 ttaagaacta cagacattaa agaattaatt gcttatagtt ctgtatctca tgcagcagta

27121 tacttaatgg gtgtatttag taatacaata caaggtattg aaggaggtat acttctaggt

27181 ttaggacatg gatttgtttc aagtggttta tttatatgtg ctggaggtat attatatgac

27241 agatctggaa ctagatctat ttttttctat aaaggtatag ctcaaataat gccattattt

27301 gctatattat ttttaatatt atcactgggt aattgtggag ttcctttaac attaaatttt

27361 gtaggtgaat ttatgtctct ttatggaata ttcgaaagat tacctttatt aggtgtattt

27421 gctagttctt ctattatatt atctgcagct tacactatgt acatgtttaa cagaatagga

27481 tttggaggta catttagtaa atattttaaa gaaaatatag ctgatttaac aaaaagagaa

27541 ttctttatct tattgacatt ggtaatattt accgttgttt taggtgtata ccctagtata

27601 gtattagacg gtcttcatta tagtgtttca ggtttaattt ataaccaata aaaaaaaata

27661 gtttaatatt attaaaccta taacctcctc taacggataa tttatatccg tatttagggt

27721 taaattctct gttgattcat taatataatt aggaatatct aaacctaggt taatctatat

27781 aaaataactt aaactaacag aaaatacctt attatgtatt taaataaata attattttta

27841 atacttctgt atttaatatt ttaaaagaaa aattttatgc ctcaactagt acctttttat

27901 tttttaaatg aagtaatatt tacttttagt attattacta taatcgtata tttattttct

27961 aaatatatat tacctagatt cgtacgttta tttttatcac gtacatttat atcaaaacta

28021 tttgataata aataataatt ataataaatt ataattttgt attatacaag gatttttcaa

28081 gatatattat atagcttaaa gtacgatgaa tactttaagt ataaatacag ttaacttaga

28141 aacacttagc cctttaagtc aatttgaaat aaaagactta ctaagtatag atgcgccttt

28201 attaggaaat ttacatattt ctttaacaaa cataggattt tatttatcaa taggggcttt

28261 tataatatta actttaagtt tattaagtac aaattataat aaattaatca gtaataactg

28321 atctataagt caagaaacat tatacgcaac agtacatagt atagtaacaa accaaataaa

28381 cccaagaaat ggtcaaatgt acttcccttt tatttataca ttatttattt tcatattaat

28441 taataattta ataggaatgg ttaacaggag cctttgtaat ttatcattat ttatctcaaa

28501 catatttgaa gtattaatgt tacataataa cttagtattt caaacacaaa attattcttc

28561 ttttcattca agtacacaag taaataaatc taatcacgac cctagatata gttttaataa

28621 taaaaatact ttttatctta accctaatta tataacagga tttgtagatg gagaaggttg

28681 tttttctatt tcaatatata aacaagataa aaatttaaca ggttgacaag ttaaacctgt

28741 atttagcata tcattacata acaaggatat tggattatta gaagctatac aaagaacttt

28801 taaaacagga aaaatatata aacacggaat tgattctgta caatatcggg ttagttcttt

28861 aaaaaatctc caaataatca caaatcattt tgatgcttat cctttaataa ctcaaaaaag

28921 agcagattat attttattta aacaagctat atctatgata aaaaataaag aacatttatg

28981 tacagaaggt ttattaaagc tagtaggtat taaagctaca ttgaattgag gtttatctga

29041 caaatttaaa gaaagttttc cttcagtaaa acctgctatt agaccttcag ttatgtacaa

29101 caatttaaaa attggcgact ttaattgaat tagagggttt atagaagctg aaggaagttt

29161 tcaagttatt attcaaaata gtaaaaacgt tagtttaaga ttttcaataa ctcaacacgc

29221 taaagatgaa gaattattaa aagatatcgt tagttattta aactgtggta gatattataa

29281 atcacctacg cgtgatgaag gtcaatactt ggttacagtt tttacagata tagataataa

29341 aattatacct ttttttaatg aatactcgtt attaggtatt aaaaaatatg actgtagaga

29401 ttttttacta atatttgaac taataaaatc taaagcccat ttaacagacg aaggattaga

29461 aaaaataaaa catattaaag ataatatgaa taaaaataga ataatagaag aataattaat

29521 aaatgtttag ataattttaa tatcatagat aaataatgat aaatacaaaa aatttcacta

29581 tatgctggaa acttctaaag cctttaggta cctacaggta aaaatcttaa aggatgaaac

29641 aatgaacaat cagcaggaaa ccaaaatagc aagccatgtt atgagtagga tcctcagaga

29701 ctacacgtga aaaattattt aattatttta aaatgataat taaatgatta agatatagtc

29761 caccatagta tgtctataag acatattatg ctgtccatat agctttgcat caactagcca

29821 ctttgtttta actttttctc ttagttttac tatagtttta ggtgcaacaa tattaggatt

29881 tcaaaaacac ggtttagaat ttttttcttt actagtgcca gctggttgtc ctttagcttt

29941 attaccttta ttagtattaa tagaactcat atagtagatc aggctactct gtgaataaga

30001 gccaaactcc ggggaagtcc taaagcttat aatactaaac aatatacaaa aatatattag

30061 tggctgaagt aattactcag gtatagtaac aagttataag atgaacgaaa gtgaaatgga

30121 tcatcgcgga tctaaaacac ctcttaacaa tgtgtaaaag agcaacgagt agacggttct

30181 tcagtgttat gattaatact gtaaggtgta ctctagtcgc catgaaagtg gttttgagta

30241 gaattaagta actcaaatta aagtttctgt atagtcttgg tcagtttatt tctcaaaggg

30301 aaatgtgtag cctatttatt atatctttag ttcttaaatt aaggttgata ctttgtaatg

30361 attctctgga tacttatgta gagcctacgc ggaaatacaa cagaacgcaa tttaaactgt

30421 attatagccc agttagttgt atatatactt gtaaaagagg tatatctaca tcttctaacg

30481 tagaacctgt taaagtttat ctttatcctg ataaggaaaa agtagaaata atcagggaaa

30541 ataaagatcg ggttggtata tatcgttgag taaatttaca ttcaggtaaa agttacatag

30601 gttcatcctc aaatttaagt ataagattta aacaatattt taattataat catataacat

30661 atcctaagcg aaatttaaga atttataagg cattgttaaa atatggatat tcagggttta

30721 gattagaaat attagagtat tgctatcctg agattttact tcaaagagag caattttact

30781 ttgatacatg taaccctgag tataatattt taaaagtagc tgggtctcct ttaggataca

30841 gacatagcga agccgctaaa aaattaatta gtctagcatc taaggataga aatgtttcag

30901 aagaaacccg tgaaattaaa agaggaatct tattaggtaa atctttagat agagaacatg

30961 tagaaaacat gcgtttaggt aatactttaa gaaaacctgt tttagttact gatattgaaa

31021 caggagatac taaagaattt atttctctta cagaagctgg agagtactta ggtatatcta

31081 gagtaactgt aggaaaatat ttattaaatg gtacttatta taaaaattat aagatatcta

31141 gttctagctc tagtgcgggg cgcaccaatg aaactagtgt atctaataat acactatctt

31201 attctcaaca ggctattttt tttaataata ttgaaacagg agacgttaaa gaatttgttt

31261 ctatgtcaga tgctgccaaa tatttagata tctctagggc gcgtttatga tattttttta

31321 ataaaacaga tagctctatg aatgatacta taaaagggta tagtgtatct aagttagaca

31381 gcacagatgt taaagcaact agaaagacta aggaattaga agttaccgat cttgaaacca

31441 acaaagtcac tatttaccct tcattcacct tagcagctaa agatttaaat atctcaacat

31501 caagtatatc gggttattta gcaaagcaac gtactaatcc ctttagaaaa agatatatat

31561 ttaaattagt ttaacaacaa aaataaggga gcttcgcata gcctatgtct caaattttag

31621 actattttct tagttatcct gctctactgt tcatcaagag gcgctggatt ttatatgttt

31681 ataattaaaa taaaaactga tcaagagtac agccgatcat atttagctcg taatatttct

31741 ttaggtttaa gattagcagc taacataatg tgagtatagg tctctcactt attgtgttta

31801 aagagccaaa ttccggggaa gccctaaagc tctaataact aaacttttaa aggaaacttt

31861 ttaagcggcc cagttaatca ctgcgggtat agtaatatca ttagagattc tagcaatagg

31921 aatgggtaat cgcggatcta aatcaaataa agacaattct ttatttgtaa aagagcaacg

31981 agtagacggt tcttcgataa taattgttat tattgtaagg tgtactctag tcgccgggaa

32041 agccggttct tgtaagaaac taagtaaatc aagattaaag ataacacaat agcctatcct

32101 taattatgta ttctatgcaa taaatctctt tttttccttt aaagaaagaa taacgtttaa

32161 atgttatatc tttaaagaaa ggataaagtt gtggacgatt tatattatca gatacaaacg

32221 tactaattag taaacataaa tatataaaat ataataaatt atattgtaat aatgcaagac

32281 aacaaggttt aattaaattg acttcatata attgaagaac aataaatact agatcatttt

32341 caacatcaaa tataaattta tcttcaaatt caattgtttt ttctaacgca gatgaaaata

32401 aacctgatat acttaaaact attaaaggta aatctggaat ttacatgtga acaaaccaac

32461 taaacggaaa aaaatacgta ggtagttctg tatttttatg tttgcttaat taagtatata

32521 atgataatta gttcacaaat taaggtagac attgtgatta cgaaatagta atccaactat

32581 acatcctttc actaaaaggt atgctttctt taaaggatac aagatgcata cgtaaaatca

32641 acgtagatat ttctcaagtg ggcgtgataa ctttacccct acccttgtat accctgacgc

32701 tttttctaat aaatctatga tactaaaaga caataaaaat aaagtaggtg tttaccgttg

32761 agttaacaaa ctcaatgggg attcctatat tggtagtagt gtagatttaa cacgtagatt

32821 tagagtatac tatgattttt cttatctttc agttagaata caaaaaagca aaagtataat

32881 ttatagtgca attttaaagc atgggtactc caattttcaa ttagaaatat tagagtattg

32941 ttgtaaagaa accgttataa gtagagaaca atattatatt gatttattta aacctagata

33001 taatattaat cctacggcaa gttcaagact aggtactact cattcagaat caacgaaaga

33061 aaagatgagt aacttggtta aagggcgaaa attaactgaa cacactaaaa atttacttag

33121 cttagcgaat aagggtatca ataatgcaaa ttttggtaaa agacatagtg cagaaactaa

33181 agccttaatt agtttagcta gaataggaaa atcttttctg tctgaatcta ttaaagataa

33241 aatgagtgca gaaaaagggg taagtataaa agttttagat ttaaagacta gtacagtttc

33301 agtgtatact tctataacta gagcagctaa agaaatgagg gttacacagc cttcgctttc

33361 aaagagactt aaagaaacac aaggtcccat aaaaaacgtt tccaagttga aaaagtaata

33421 gaaaattcta aaagttaaat ttaaactgaa tattataatg tgaaaagatt gttaaatgag

33481 actagtatgc ctatatgtag tgccttatta aaatacgggt ataataattt tagtttaact

33541 atattagaat tttgtaacat tgatagttta atgtcaagag aaaaacacta cttcgaagtg

33601 tattctcctg agtataatgt acttaaaaca cctggtagtc cagatagagg ttcaggttga

33661 aaacattctg aagctacagt agaaattatg cgtcttgcag ctcaaaaaag aatagagtcg

33721 ccagaatata taaataaaat atccgaagct caacttaata gaattgaaat tgaagtaaca

33781 gatttaaaaa ctaatacttc tactaaatat catgctataa gagcagctgc tcgtgcttta

33841 aatatagata aaagatatat tgaaaactat gtttatttaa accaaaataa acctgtttta

33901 ggtagatata cttttaaatt gttatctaaa gaagaaacaa attatatacc taaagttcaa

33961 agtacttcta taaaagtaga agtaactaat atagaaacta aggaaatcac aatatatcct

34021 tctataggtg cagcagctaa agagctaggc tatcatcaag ctagtatatc tttatatcta

34081 aaagaaaata gaactaaacc ttttaaaggt ttacatttat ttaaattagt taaagataac

34141 taaatattaa gggttaattt gtgtacgaat gagtggtcat atgttacttc atattttagc

34201 tggttttact tacaatataa tgtcatcagg tataatattc tttttcttag gtttattacc

34261 tttagcattt attatagctt tctctggatt agagataggt atagcattta tacaagctca

34321 agtgtttgta gttttaactt cttcttacat aaaagatggg ttagacttac actaataaag

34381 tttaaataat tttagggata aacagtgcaa agtcaaaaat ttagtcatag gtttagaaaa

34441 aattctaata gttttaaata gaaaatatta atataagtat atatttagat aatttagtat

34501 caaataataa aattatattg cggacataca atcttatatg atgctttttg ttttgttttt

34561 tttttttagt tagataaaac tcgataacgc ttagatttat cctaaaataa aaaatattat

34621 aaacctttaa gtgatgtaat tgattattta attacatcta aaaatagatg agtttggtga

34681 tggctctgat tgaacactgt ccaaaggctt gacacatgct aatcgtacgt ttaatttaat

34741 taaaaatttt attaaaagtg gtgaacaggt gagtatataa ttattaacct accttgaggt

34801 aaggtacaat aaatacctta tataataaag gaaaaataaa ttccgcctta agacgaatcg

34861 ataatttaca gggataggta gtagtaaagg taacggcttc actagcctaa tattctctta

34921 gtcgaaactg agatggttga tcgaccacat tgggaatgag aaaagcccaa ggcaaggaag

34981 tacagcagtg gggaattttg gtcaatggcc taacggctga actggcaatt tggaggaatg

35041 acctatctta tttagtaata ataagaatta gattgaaatg gtacaattaa ttattaaatg

35101 aaattctaaa tatataacat tataatgact aaatatattt atgtcttgac taattacgtg

35161 ccagcagtcg cggtaatacg taagagacta gtgttattca tctttagtag gtttaaaggg

35221 tacttaaacg gaagaaatta cctaaacaag gtataatttc tatctagagt ttaatatgaa

35281 aaggtagaac ttgaggagta gagattcaat gcgttaatac ctaataaagg acaagttaag

35341 gctaatgcga ccttttatgt aaaaactgac gttgaaggac gaaggcttag agagcgaata

35401 ggattagaga ccctagtagt ctaagcagaa aatgatgaat gccataggtt aggattactt

35461 agtctataaa tgtaagtgta agcatttcac ctcaagagta acatggcaac atgggaactg

35521 taatcaccag gccgtttctg acaacagtag tgaagtatgt tacttaattc gatgacccac

35581 gaaaatctta ccacgatcag tataatatat taataatata atacgttata atatattacg

35641 agtgttgcac ggctgttttc ggttaatgtt gtgaaactgt ggttgaggcc atgcaattaa

35701 cgtaaacctt tgctttattt atatatatat tttttataaa gctgttttta tctggaaaac

35761 gataaaataa tagggccaaa gacaagtcat catgaccttt aaatcgtggg ctgtagacgt

35821 gccacatttt cctaaacaaa gagaagcaaa aatgtaaatt taagctaatc tcaaaaaata

35881 ggataaaaaa agatatggat tgtagtctga aactcgacta cataaataag taattactag

35941 taatcgtgaa ccaccaggtc acggtgaatt acatctcgga ttggtactaa tcactcgtcg

36001 cacgctgaaa ggagtatgtg caaaaagttt gctttcctgc tataattaaa taaataatag

36061 ggtttaaaca ttataacagg attcttcgta tgtatgactc taattagtgt taagtcgaaa

36121 tatggttcgt gtagtggaag ttgcacgggg atttaatatt tttatgtaaa taatgtataa

36181 tttttatatt atacatataa ggagagttcc tttattggta agaagggttg agctgtaaac

36241 tcaatagcta tattctgctt taagagttcg aatctcttgt ctcctactat aaaagtgcaa

36301 aatattttgt tggtatgttt aaattaatat acattaaaat aatgttttga taagtggtta

36361 tcgtatagtt agattggtag ctagctataa ttaaattcgg tttcaacata aaaatacaat

36421 aaaattttgt aataaatccc tgtcaattta ggtaataaaa gtaatattaa aatatgatga

36481 cattctactt tttttttagc tgttaataac tttaaaggtt tttctatttg tcccgctata

36541 catttaatcc ttttaatatt ttccttggtt ttacagttac aaacggaagg cataagcctt

36601 tactcaaaca agatatacaa gtgtaacagt aatattaacc tacgtaacag taattatgca

36661 ccgataagta tgactataaa ttctaaggat agtgtacttt ataataattc ttttaaacct

36721 aagagtcttt ttagtcaaga atttgcctta aagggttgtt gtgctctatt aacggtatga

36781 aagataaaca aagaaatttc tcttttactt cgtccctttt tagtgattct acctccagtt

36841 tgtctccatt agtaccagta aaagtatatt ataatgcaga gatttgtaaa ttagatatct

36901 taaaagaaaa taaaggcaga gcaggtattt atcggttcac gaataaatta aatgggaaaa

36961 cctatgtcgg gagtagtgtg gagttacgta acaggttttt acattattat agattgtcat

37021 atttagccaa taaacgacgg gtcaatctat gatctgtaaa gctcttttga agaatggtta

37081 taataacttt acattagaaa ttttagagta ttgcgaacca tcagaagcaa ttttaagaga

37141 gcaatattac attgacttat taaaacccgg ttacaacata ttaaaatttg cagggtctac

37201 tcttggatat aagcactctg aagatacaaa ggcaaaactt tcttcagtgt taaaagcagt

37261 ctgaaagtca gaaatatctg atacattaaa agccaagttg tctgcatcgt gaacaaagga

37321 aagaagatta aaaatgattc ttttaaagga agggaaaccc ctttcagatg agacaagagc

37381 caaaatatct gctgcattaa aagaagtgtg acagtcagaa gaaaaaagag ctaaatactt

37441 agcttcaaag aaaaagaggg gtgtaatagt aacagaagaa accaaagcca aaatgtctgc

37501 tgcaaataaa ggcgtgaaaa aatcagaaga aaccaaagcc aaaatgtctg ctgcacatat

37561 ggggaacaaa catggaccgg gttatcatgg acagatagaa atcttagata cagtaactga

37621 aataaaaaca gtatattcct caatgagtgt gctatcaaag gcactatcta tacctaagcc

37681 tagtatctca gcgtattttt cccgtaacac tgggaagcca tataaaggaa gatatataat

37741 gacaaaagta aactagctaa tcgtacatta catgacctaa ggattcgaat ctcttgtctc

37801 ctaataataa ccttctatag ctcaacggta gagcatgata ctgttaatat caggataaat

37861 gttcaattca ttttagaagg gcttataata tgtattattt aggactatta taaatttttt

37921 aaataaaagg tttaactaaa aaaaaaagtt ttttaagtgg atattaaaca gagaagccta

37981 ttccaggacc atccgtttca tttagtgtct ccatctcctt gaccacttta cactagttta

38041 tctttacttg ctttaacttc aaatgcagcg ttatttgtgc ataattttga atatgcttct

38101 tacctttcat ttgtatcatt attatgttta atcacatcta tgtttttctg atttaaagat

38161 attacaaccg agggtacata tttaggtgat cacactttag ttgtacaaca aggaataaat

38221 ataggtataa tgttatttat aatttctgaa gtttttttct tcttagctat attttgagct

38281 ttttttcata gtgctttaag tcctactgtt gaactaggtg ctcaatgacc cccctttggt

38341 atacaacctg ttaatccttt tgaattacct ttacttaata cagtaatatt attatctagc

38401 ggagctactg ttacatatgc ccatcactct ttaatatctg gagttagatc aactgctgta

38461 tactatactt taattagtat ttgattagct attatattta ctttttttca aggtgtagag

38521 tatgaagtat catcatttac tattagtgat ggtgtttttg gagcatgttt cttttttggt

38581 acaggtttcc acggcttaac tacgctagtt cttctacttt cagaatatag ttttattaat

38641 tttacatatc cctttaattt ttattttaag actgttaaaa atttttatta tattatgata

38701 aataaaagat ttttctctac agacgagctt ttgtcgtacc agcctaaaaa taaaggatta

38761 gaacaacttt gcccttattg agttacaggg ttttgtgatg ctgaatctag ttttagttta

38821 aaagtctcta aaaaaagtac ttcaaaatca ggatgaggtg ttattccgga ttttcgaata

38881 gagttacata gtagagatac tttattacta agaaagatac aatctttttt cggtgtaggt

38941 ataattagtg aacgtcttga cagaaacact atagtctata gtgttcaatc ttttaaagat

39001 atttttaata taataattcc gcattttgat aaataccctt taataacaaa aaaacaagct

39061 gattatattt tatttaaaga agctatttta ttactttgta ctaaggcgca gcatagtatt

39121 gaaggtattc ataaaattct tagtataaaa gctgccatga atactggatt atctgatagt

39181 ttaaaaaatc aatttcctac tgtattttct gtcgctcgtc cttatattac acttaaggac

39241 attcctagcc ctagctgagt cgcaggattt acagacggag agggatgttt ttatgtcaat

39301 actaaaaagg ctaagacttt aactggttat cagattatta tgtctttttc tatatctcaa

39361 cacgtgagag atgaagtttt attaactaaa ttcattgatt attttgggtg tggtaatatt

39421 gaaaaagttt ctacaagacc tagtactgtt acatttgttg tttataaata ttcagatatc

39481 aaagataaaa taataccggt ttttgagaaa tattctttac aaggtataaa aaatttagat

39541 tacctagatt tttgtaaaat agctaatgtt atggagaaca aatcacattt aacagcggac

39601 ggtgtaaaaa aaataaaatc tttaaaatct ggaatgaata gcggtagaac gttataaaaa

39661 tgcgtgtagg ccctgttaaa ttttaattat tactctaagt gataataatt aaaattagca

39721 gaaaataagg taaattgtat ggaactctaa ataaaaatat ttagatgata tgcagccaac

39781 catatattaa acattacata taattttaat atttggaggt tcaacgacta aattacggta

39841 ttatactgta atatatattt tactgggcaa tatattgtat attgtacaga tgacatagtc

39901 tgatcaatta tgtaaataat tgaatataag gtaaaactta tctaacaaca tattagcaat

39961 aataaatatt attgtttagc catgtctctt aattaggcgt actctcattt atctgtatac

40021 gaaggattag ggttaataaa tgtagccctg tttataataa tttttctatt tttaacttta

40081 ttaactataa actattataa ctgtatacac aaatctgtgt cttactttag tgccataaat

40141 ttttcttcta atgtaaaagc taaagcaaac tcagataata acaataattt attaatttct

40201 aactctaagg taggtaattc ttttttagat agatcttttg tggaatggtt tgtaggattt

40261 acagatgctg agggtaattt taatattaaa ttaacagatt taaataataa tacctttaaa

40321 tttgtccaat ttacattcca aattggtcta cataaagatg acattaaagt tttagagttt

40381 atccgtgata atttaaattg cggtcatatt tctaaatctg ggaataaagt taattttttt

40441 gtaaatgatc aaaattcttt attaaatgtt ataattccta tatttgatca agttaatctt

40501 aatagctcta aatatcacca ttttgtttta tttaaaaaag cagtaatgtt aactaaaaat

40561 aaagaacatc ttacagacaa aggtaaatta gctataatag attataaaaa agaaatgcaa

40621 agtatgtctg ggaaatgagt acctagtttt atctataata aaattaatat aactaaacct

40681 tgattagctg gatttataga tggcgaagga tcattctcaa ctaataaata tgtgcctaga

40741 tttaaattag agaatcatat taaagaaacg gaattatatc ataaaattaa agaatttatg

40801 ggtacaccta aacttatata cacttcaact ggatctagag aagatagtaa tcctactatt

40861 gtattagaaa ttaataaaat taaatatatt aaagaaacat taataccatt aatgtatgat

40921 agtaataata aacttttatt aaaaacattg aaatccaata attttttact ttgagtaaat

40981 ttagtggaca tgtattattt aggctatcat acaacattag aaggtaaaca tttatttgac

41041 ttaataaaaa ataatattaa taaatatagt ttaactacca atagtcatct caaaagagaa

41101 tttaaaactc tatctgaaat taatttatta ttatctgagc tttatacatt aaatagccca

41161 tatgaaataa tagatggact tagatactat agaggtacta ataaattagt aagtgaatca

41221 actcaaattg tatctataga tgagaacaac tgtaaagttg aatttaatag catgagtgaa

41281 gcagctaatg cattaaatat atctagaaaa tacataaaag aatgtttagc atcaggtaaa

41341 agttataaag gttatacctt tgttttaaaa tagaaaaaac aaaattatta tagattaaca

41401 atagagtaaa tagcaaaaac atccactaaa tctaatattc ctcatacgcg gtgtatgcgg

41461 tataggtaca agattttgta gacaatttgc tgccaagtat gtatttttag agcgaagtgc

41521 ttaaatatat aaaggttcaa cgactagcca ataaatatta taatacttaa atttaagtaa

41581 gagaatggca aaacactcta cttattaact attgttctta atttaagact ttacgtctaa

41641 gatatgtagg cactaataag aagacatagt cttaacaata attaaaatta ttgaataaat

41701 acgaagtaca cgttataatt ggaacacttt ttttgctagt tgctcttgca ttgatagtat

41761 cttaccatgc taccgacagt catcaccaag gttatgaatc agcgatattg tattgacact

41821 ttgttgatgt cgtgtgacta tttctatata tagcgatgta ctattgagga tcatagataa

41881 gttatcaagt aaaatagatt taagttatgt attacccttt gtattatcaa ttagtttagt

41941 actaatttgt atttataacc cggataaatt ttaacgtcgg ctgtttattt ttgtatatat

42001 tcatattttg catttaatac aggtattata ggtagaataa atatttatat atatatattc

42061 ataaaaaaaa ctttttattt attacttata ttttctttta tttttattct attcttaccc

42121 ttatgaaggt ttagtattta ttaatcagtg gagcggttcg aattttaatt ctaataataa

42181 tttaccaaat tttaataata atggacctaa tccccctcaa aataatgata tgatggtagc

42241 cagtgggtcg catagtgacg aacgtaatcg gaggaatact aatatttgaa ataataaccc

42301 tccttttatt ggtcgtcctg ttactcctgt gtcaccttta actcgcgctg gtcttattca

42361 tgttgatttt agtcctactt aaactttggg tgttcattca aattttgtat ttaaaaatga

42421 gagtagattt atatttaatg atatgactga ctatttaaca agatcccata atggtacacc

42481 attatacaga tgaccatggt tatagatgta gtacttttag tactaataaa gacatccatg

42541 gcccctatca agaagttcct ccttatccag ctaatactta tttatggaat ctggagtata

42601 aaattttata tcgtgtcaaa actcaatttt atcatgataa catgattgaa tatgtcaaga

42661 atatacctgt agacggggcc cctcagaacc ctagattacc aacaaattat cctttttatt

42721 ataaaggttt taatagacca atgaatcaaa tggaaataaa tcaagcacta catacattag

42781 ttacagtgca tcaccataat tcccaaagaa tatgcttcta tcaaagtccg ttagttacgg

42841 atggcacata tagcgataga gattataaca atttgatgac tgcaagcttg gttaatctta

42901 gatacagaaa taaatatgca agtatagatc attatctttt actcaataaa ttaaaaagaa

42961 ctccttgata attagctcga tagctttatt tattatcagt attaatattg ataatacaat

43021 aagggatctt cgtataacgg taatacatgt gacttttaat cattaaattg tcagttcgaa

43081 tctggcagat cctaagaata taaaagaatt agtaacttaa ttggtaaagg acttccttgt

43141 cacggaagta gatatcggtt caaatccgat ttaattcgca aaaaaggaaa aattgctatt

43201 ggtagggtaa agtatttgct atatactgtg tttttcactt gaaagttcga ttctttcttt

43261 ttccgtacaa gagtatagtt taatggtaaa acgaagagct tcaacctctg aattctctgt

43321 tcaaatcaga gtactcttga ataaaaattt acaatgttac ctgtctattt aggtattata

43381 ttatagtaat taatatgaat aacttgttta ttataaatga aaattttaca aacggatata

43441 gaagtgaagt attagatata ctttgtttat taattatatt atcaggtata tttgtaatag

43501 taagtaaaaa tcctattata tctctattat ttttaatagg gttatttgcg ggtgtttctt

43561 gttatttact tataataggt ttaaattttc tagggttatc ttatttagtt gtttatgttg

43621 gtgcagtgtc tatattattt ttatttattt tgatgttaat taacattaga atgagtgaat

43681 tacaaagtaa tactagtaat agtatacctt tagctattag tattgcaatt ttatttaatt

43741 atcctttatt ccaattatta ccttataatg tagctatatt aaataataat aatattataa

43801 ataatatatt atataatata tcatttaata aaatagataa tggattaaaa actaatttag

43861 atataaataa taatgataca ttatttgtaa caagtaaaat atgagatggg aatttagctg

43921 aagtgggcca tatatcaagt ataggtaata tattttatac taattataat atatgattaa

43981 taatagttag ttttatctta cttttagcca tggtaggagc aataacaatt aatataaaac

44041 aaaaaaatta aaaaaaaaag gaattaactc aatggtagag tgaccgtcct acacacggca

44101 agttatcagt tcaagtctga tattccttaa attccttaac ttaatggtaa agtgtacttt

44161 tgataaaagt attatcagtg ttcaattcac tgaggaatta acgtaagaag attggctgag

44221 tggtttaaag cggctgtttt gaatgcagtt aaggttaaaa acttcaaggg ttcgaatctc

44281 ttatcttctg atcaatacaa atcctagttc aattagtaaa acgtattctt tgggagaata

44341 tattctgagt gcgagtctca gggatttgat atatatatat atctatataa atattaaata

44401 tttttaagaa atataaatga tacgaaataa aaatttatac atataattat gtaagtatat

44461 aaagagacta ttaggtaata ctagctatat attaaagaga aacatgagtt tatcaaaaaa

44521 aaaatttgtg ataattgact ctaagataaa tcttaataaa aaaaaacgaa gtgaattgaa

44581 ttatcttatt agcttcagga aaaaaaatca aaagagattc tatgagtagt gtgaacaaaa

44641 atagaaaagt ctaaataata agtaaaattg ccataaagct gtttgaatgt tatggggaac

44701 cttcctcaaa gactaaatat aatatataag cgatagcata aagtaccgtg agggaaagta

44761 aataattagt agttttataa gcagctcgag caaaagcaag agcgtacctt ttgcataatg

44821 ggtcaccaag ttaattttag atgcgagcta atgcgtagtt aaaccgatca tataaacaat

44881 gaatagtatc taaaattaga cccgaagtac agtgatctta ccatggtcag gactataaag

44941 gtccgaacgg gttattgttg caaaaatatc cgaggaattg tggtaagtat agtgaaagac

45001 aattctgact gtattagctg gttttctgcg aaacctatac tagtaggcaa tttaaataac

45061 atcttagcag gtacagaact taatctcaga caagatgtat taaacatttt ctttttgtac

45121 aaatcgggga atcatgagga ttttaccggt gagtttgtgg actcggaatg gcaaagatga

45181 attttaaatt atcagacata taatgataag gttgaaatac agcctatttg tagaagtgaa

45241 ccttctgcta taaaccatca aattgcggga acgccctaaa gcaaccataa ccaagtaaga

45301 gtagtaatac atcttatggc acaggtaatg actcgtggta tggtaaaatc atggttgata

45361 aacaaaagtt aaatgggtta tccgcagcca agcaccgatt ctatacgaag gtgtgcagtt

45421 catcgactaa atgttggttg gcgaaagctt aagatatagt caagcctcat ctgaaaggat

45481 gcaatggcaa taaagtaacg attttgtttt ataataaata attattatta atacttaagt

45541 atttaatgta aaaagaaaaa aatataatgt cacaattaaa caataaaaat atagtacctg

45601 taaaaagcta tgataatacg tttttaaata aaaagaccat tttggttgaa aataaaggta

45661 aatctggtat ttatcgttga attaataaat taaataataa tacttatata ggtagtgggt

45721 tagatttatc aaaaagatta agtgaatact atagtcaaag tgaattaaaa agaaacccta

45781 gacctattca tgctgcttta cttaaatatg gatacgaaaa tttcatactg gaaatactag

45841 aatattgcga agtagatgag cttattgaaa gagaacaata ttatttagat ttattagatc

45901 ctgaatataa tatattaaaa tatgcttatt cattattagg gtataaacat actcctgaaa

45961 atatagctaa atttaagtta agaaaagttt cacaagaact taaagatata ttatcttcag

46021 ctcattcagg taaagaagtt agccaagaaa ctagagataa attatcttta gctataacta

46081 attataaaaa aaataatcct ctttcacctg aagctttagc taacattaga gctaaaacaa

46141 ctgaacgtga aggagtaact gtaatacttt taaatactga aactaatgaa gaattagagt

46201 ttcctacttt aactaaggct ggagaatact taggtataaa aagacaagct atacgaagtg

46261 caataaaagt cttgttaaag gactatatcg tatttcagaa aaaaaataaa cagtagtata

46321 atacaggtac aatatataaa ttactttaat ctcaataatt gaggtcagat taataaataa

46381 tctgttgtct aaatggataa ttcataataa ttatattatg gactatttag ggagaaaatt

46441 taaacttaag tttaaatttc tgtgtatgtc aaaagggaaa cagcccagaa caagagttaa

46501 ggttccaaaa ttattattaa gtgaaattaa gaaggtctat atgtaaaccg acaaggagat

46561 tggcttagaa gcagccataa tttaaagacc tcgtaccaga gcactagtct acgtaaaaag

46621 cgttaaaaat ttaacggatc taaataatat accgatacct tgtctataaa tgataataat

46681 aattattatt atttatgggg tagcagaacg ttgagtaaat tttagtgttt ttttttataa

46741 aaaaaattat attataactc aagtgataat ggtgacatga gtaacgaaaa agaaaactcg

46801 cctaaagctt atggttagga ttaagtatcg gcctctaagt ttatacccta aggggtaaag

46861 cgatgagaaa atcttattac tattaaagac accatttttt aagtgaaaaa tgagcttgaa

46921 aaaaatagta aaaaaaaata accgtaccta ggatctacaa caagtaagct agtagagaat

46981 acgaaggcgc atgagataac aatcttaaag gaactcggca aaatgactcc gtaacttcgg

47041 gataaggagg gctcatttat tctgattaat atcagatata gaggaagaat cataaaatag

47101 tgttgtacga ctgtttaatt aaaacacagc actttgcaga gatgttaaat ctaagtatca

47161 agtgtgaaat ctgcccaatt ccggctaata actaacttag ttaaatttat ataaatttag

47221 ttaataagaa acaaccggca aatggcggcc ttaacctgag ggtcctaagg tagcgaaatg

47281 ccttggccgt taaatgcggt cttgcacgaa tgatgtaacg atacaacagc tgtctctaag

47341 attgactcag tgaaattgga ataactgtgc agatacagtt tacctctaga tagacgagaa

47401 gaccctatgc agctttactg ttactagtta ttgaatataa ttaagtttat tattagttta

47461 aaaggtttat tgataaaata aaagtgaaag acctttattt acttattata ttaatacttt

47521 tgttaatctg gacagtgact agtagacagt ttatgtgggg cacagacccc ttaaagagta

47581 aaagggtgta tctaaaatat ataacttaaa gtttatttta attttttttg tttgttatta

47641 ttttaacttt gttatattta atcttttctt ttgtgtatgt aaatgtatat tatatgtaca

47701 tagatataga gttaggtagg attttaatta tggggcaaat taattgtata taaaagatac

47761 atatgtatat gttatcttaa tagttattat aatatttaat tattaataat tatgtttaat

47821 acttataaag ttgaatggct taatcttgct ttactgtttg attaacaaca aatcttacag

47881 tcgcgtaagc ggggcattag atcacaagat acaataagga aaggtcttgg attattggaa

47941 aagctacgct agggatgttt gtccttcaat attttttaat aattgataca tattgggtta

48001 atttcaaaaa ttacttactt taattgtaag tagatttgaa gcgaagttta tttatgtata

48061 taaataaaat tgttcaacga ctaaaagtaa taatacttta ttaataccca acattttttt

48121 ttagtaatta attttaatat tttaaacaaa aattgcaaaa aaacactaat ttttaccaaa

48181 ataatatatt tattaaaaat ataaataata aatataaatt aatacctatg aatataaaaa

48241 ataattttat aggtgagaat aattattttc cttctgatta taaagaatga agtaataata

48301 tatattatta taataataat aatattaaaa atatacctgc ctatgattta gtaataaata

48361 aattattaaa aggttatttt aattcatatt taaacccgaa tattaatttt ttatattcaa

48421 ataaaagata tacatcttta aataaaatat ttattagtaa agctaatata agacatacta

48481 attctaaagc tataataact atttatgttt atgatagaga aaaaataagt ttatctaaat

48541 tttttataaa atatagccat attgtattag ctgctatcaa agaactaaaa gaatttgtat

48601 tagaaaataa taattttatt caaaataaat taaaatcatt tcagggtgat aatgaattat

48661 taaaattatt aaattttttt aaaagtagaa aattaaaggc ttgattaaac gaattaagat

48721 ataaagatat ttttatttac aagttaagta aattaattag taaattttat agaaaaaaaa

48781 tagaattcaa tataataaga ttaacttcta ttgcatataa ttcagatatt ttagtagatg

48841 taatgagaaa aagctataaa aaaactgggc atgtaagaaa ttcaatgaaa tttatattaa

48901 atcaaggtct tattgaaaaa atagataata ctaatgatag agttagagct actaaaaatg

48961 tagattttaa ccttttagct aataaatatc aaaatttaaa tattaattat atagcttgcg

49021 tgcgcagcgc agtgaaagac gtaaatataa atgaaactat aaaaaattta tataacacta

49081 aaaataaaaa taatgaagaa attatattta attccattaa gtataaaata ttaggtggta

49141 taagaataga tataaaaggt agattaacta gacggtttag agcagataga tctgtttata

49201 acacaatctt gaaaggaagt tttagaaaca tagattcttc atataaagga ttaacgattc

49261 taggttatag aggatgtgct agctctagtg tagattactc tatgtctata tctaagcgtc

49321 atgtaggagc atttgctata aaaggttgaa tatcgggtag atcttactct acttcaacta

49381 gaaataatag taacttagat actataaatt taatagacta tttaggtaat attgatcctt

49441 gaactataac aggttttact gatgctgaag gaagttttat gttaagaatt aaaaagaacg

49501 atcaaagttc tattaaatac tcagtataat tagtttttca aataggttta cataaaaaag

49561 atattgctat tttagaacaa attaaatcaa attgaaatgt aggaaatata acaaataatt

49621 ctaataattc agtaactttt agagtaaccc gttttgagga tttaagagta ataataaacc

49681 attttgataa atatccttta attactcaaa aactaggaga ttataacctt tttaagttag

49741 catttaatat tatagataac aaagaacact taacccttga aggtatagaa aaattagcgg

49801 gtattaaagc taaattaaac tgaggtttaa ctgaataatt agttaaagct ttccctaata

49861 taagttctgt cgatagaccg ctaattataa atcaggaagt accatcttcc aaatgattag

49921 caggaaggtg aaggttgttt ttttgttaat ctaattaaat ctagcactaa actaggtata

49981 caagtacaat tagtattttc tatagttcag catgatcgtg atagagcttt aatgaatagt

50041 ttaatatctt tttttaattg tggctatatt aaagaaaaaa aaaatcagaa tttagatgat

50101 tagagtttgt tgttactaaa ttttcatata ttaatgaaaa aattataccc ttttttcagt

50161 taaataaaat aataggtgta aaatccaaag attttaatga ttgatgtata attgcgaaat

50221 taattgaaaa taaaaagcat ttaactgaag aaggtttaaa tgaaatacgt aaaataaaag

50281 ccagtatgaa tactggaaga attagtggaa gataatataa aatattaagg aaaaaaaaag

50341 aaatgaagaa atagtctgaa ccattttgtg aaaaatggaa ataaaatttt atgataacaa

50401 gttgaacagg ctaatttgcg caagagtgaa cgcaatgagt gcgcggtttg gcacctcgat

50461 gtcggcttaa cttatcctcg tggatgcaga aactatgaag ggtacgactg ttcgtcgatt

50521 aaaaagttac atgagctggg ttaaatttag ccctataatt atgcatttaa ttattaattt

50581 gcatatatta cgaatatact ggaaaaattg caagaaaaat actttatact aagtattaat

50641 ttgcagcgga tttatattta caatcataaa tataatgcgt tcaacgacta atggtttaaa

50701 ccaaacataa tccagaatta tctaaactaa aaaattttaa taaaataaat aaaaaaaaaa

50761 aaatgaccaa tacaacaatg ataagattta ttaattctct taataaatta gaaacgtaca

50821 aaaaagaatt acgaataaaa actaagctat cgttatattt aaatgatagt cttattggtt

50881 tacttttaag tgacggatac attgaaagaa catcgccaac tagtggagct cggcttacta

50941 ttagctttgg tgctaaatat gaaggttatt ttaaccattt gtataagctt tttgagcctt

51001 atattaacac agagccttct ttaattagcg tttacaataa gaaaacagat gatacgtata

51061 aagtatgaag atttaaaact gcaagtttac ctcaattaat ttattatcat gatttatttt

51121 ataaacctaa ttaaacgggt aaattagtta aggaaatacc ttctaatatt gaagaactct

51181 taacgccagt ttcattagct catttaataa tgggtcgcgt cgcgcgcgcg atggtaattt

51241 aaaaactcct gataatatta tacgtattta tacaaatagt tttacacagg aaagaattaa

51301 ggtactagct gacgcaatta ctaataaatt taatatatta actagagttg tacatgatag

51361 gaataatcaa tatattatag taattagtaa aagtcaatta ccattagttc aaactcttat

51421 aaaatctcat atgcacccat ctatgtacta taaattaggt ttaaatggag aaggtctaga

51481 atttgattat aatatacctg cattaaaaga atttagtaag gattgtgtag attattctta

51541 aagtaggtgt tacatttgta ggtttaatgt aattagttta gataataaga catagtctga

51601 acaatactga aaagtattga aactcaaatt tatttgtaag ataaaggcga gtgataacaa

51661 gttgtaaata cgtcgtgaga cagtatggtt tctatcttct aggggaaatt agaatataat

51721 aaggattaac cttgtacgaa aggaacgtgg gcaacgcctt atttgtaaaa agaataatgg

51781 ttttgttaac ctatggttta tctgttgttt atgtgcccag atattaattt aggtataaaa

51841 tttatacagg gatgttactt tcacttaagt aaatatataa cataggcata gcagaaaagc

51901 taagttagcc agagataagt attgaaagca tatatatacg aggctcgcct taagatattt

51961 cttaaatata cgtaaaagaa tattacgaac ctcataggct tagtttgtaa ggatttagat

52021 atctttagat actaagtact aattttataa ataactgaat atttaccgta tcttatattt

52081 agctcagtta gcataaaagt aatgcaattg ttttgtaatc aatggacaca agtgcgatac

52141 ttgtactggg cttaggacta gtagacaagt ggctatgtca tagctttttc aatgctaata

52201 ccgcgggttc gaatcctgcc tagtctaaga ggctgtaact taattggtag agtgtactgc

52261 tcatgacagt aattgtaaaa acctttaagt gataagattt aattgaaaga atatttatat

52321 taccagagta taagaatatt taggggcgct gcgctagcaa gctagcaagc tagcaagctc

52381 ttaaatttaa ggtttaattc cttaatgctc ttatgaaaaa tgtatttaaa ttaagaaata

52441 aagtagttta tctttcaact ttaaaagata agtccatagt tgccaattta gaatgcttcg

52501 ccttaaaggg atacttagta caccgcccat ttacccataa aagatattca tctacgactc

52561 aacctctggt ctttaattta ccctcttttg agtttttgga gtgatttagg ggatttacag

52621 acgctgaagg gtgtttttta atagctaaat cagctaacag ttttgcattt aggtttatta

52681 ttaaattaca taaagatgac ttaaatatat taaattttat tagagcttct ttgaataaca

52741 ttggtaatgt ttcagtttat gaagattcag ctagttttaa agtaacatct ctttcagaga

52801 ttaaatttat tatagagatg ttttctactt atcctttaaa ctccgacaaa cacttaaact

52861 ttttagattt taagaaggct tacgaacttt atactttacc cgtagatgga ccacaagata

52921 taaacaatga aataattaaa cttaaaatta atatgaactc taagagagct aaacatgata

52981 tagacgtaaa ctctgaatat ttaattaata gtgataacca tgaaataatt ataacagata

53041 gttgactatt aggttttatt gaaggagacg ggagtttctc tgtagctaag gaaaataaca

53101 tgttactttt ttctattagc cagaaaggta atttagtttt aatggaagca ataaaaattt

53161 atttattaga attagcaaaa aaaatgggac atggtaccct aaatacggtt tatttaacta

53221 aatctaaaaa tagtacagga aatattgctt atgttttaat cattaaatcc aaagaattta

53281 ttagtcacgt acttattcct tattttgata ctttaacttt ttactctaaa aagaaattag

53341 attattttga ttgaaaatct atagataaat tgaaaagttt aggtttacat tatttacctg

53401 agggtcaaaa gttaataaac ttaataacag gtcaaatgaa taataataga ttatctacat

53461 ttaagagctc ggatttgaga cctgtagata gagatttaat tgattctgaa ctaactagat

53521 tattaaatgg accttcaaat tacgagttaa tagaaggaaa aatttttata aaatcattaa

53581 ataaattttt acctaataga ttaaaaacac aagttaaact tcaagacaat aagggattag

53641 tttttaaaga gtttgaatct aaaaataaat gtgctgagtt tttagggata agtacgcata

53701 ctgtatctaa gagaataata acaaataaac cggtaaatta caaaaataaa gagtatttaa

53761 ttaaatatgc ttaaataccg acctcggtat atactcataa aggctatagc ttaattggta

53821 aagcatactg ctcatgacag tacttttgag tgttcaagtc actctagcct taggtaagtg

53881 ttcaactcac ttcagcctta tttagcggat tagtgtaata gaaacatttt tgactcatga

53941 tcagaagata ggggtgcgat tcctttatcc gcgtattatc cgagtggtgg aattggtaga

54001 cacaagagat ttaaaccctc ttggtgaata accgtgtacg ttcaaatcgt acctcggata

54061 tagttaagca actgtagttt aatggtaaaa tgatcgtctt ccaaacgaat gatatcggtt

54121 caattccgat tagttgtata aaggggatat agtataattg gtattacagc aattttgcat

54181 attgtttatt taggttcaag tcctaatatc tccactaaaa gcccgggaag ctcaattggt

54241 tgagcgagac actgaagctg tctaggttgt aagttcgaat cttaccttgg gcataatttg

54301 atgaagcggg tgtgctggaa ttggtagaca ggctctgttt aggccagagt gggtaaaacc

54361 gtgtaagttc aaatcttatt gtccgtaata ataaaattag tttaaagtaa attatatttt

54421 ttattttaat ttggagtaga caaatgggta tgtcatagat ttttggtgtc taagattgag

54481 tgttcgaatc actcctccaa aatttgtgat agtcttcact gcgctgcgca cgcaagctat

54541 ataatattaa tatttaatta aaaaaagtaa gaataggata atatagctca attggtggtg

54601 tttattaggt tagggtggga taggtacaag cgtttatacc tacttttaag aatctttggt

54661 agttttgaga tttaatcctc aaaatatttt gttcgaatca aaatggttct aaattaatac

54721 tatgtggtaa ttcctgtcaa tttaggttaa ttttttagtt tatttttcta tgaatataaa

54781 agtaggtcaa ttaaatcctt gagttataac agggtttgct agcttgcaac gcagagggtt

54841 gctttacagt aggtttcact tctagtagta cacataaaag tggttatcaa attagaccta

54901 tgtttactat taatttaaat aaaaaagatt taactctttt agagcaaata aagaactatt

54961 ttggaaatgg tagtatatca cctagcggta acaattgttt gcaattaaaa atagcagatt

55021 taaatgtatt aattaataat gttttacctc attttgacaa gtatccttta gttacccaaa

55081 aaagagcaga ttacttatta gtaaaaaaag ttattttttt aatgaaagat aaaaaacact

55141 taggaccaaa ctatcaagaa ggacttaatg agattatatc tattaaagcg tccattaact

55201 taggtttacc tgatagatta aaaaccctat tccctaatat tactccaatt aatagacctt

55261 tagttgaatt ttcaaatata cctgacccta attgattaac agggtttgtc tcaggagacg

55321 ggtcatttga aattaatata tctaaaagtt caagtagcat attaaataaa agggtaatat

55381 taagatttag agtgactcag catattagag atatagattt aatgaaaagt ttaattttat

55441 actttaattg tggtagcgtt tatgaaacaa aagatgcagt atggttccaa ataacaaaaa

55501 tatcagatat tgatgaaaaa ataataccct ttaatacccg atcgtagggg caaaatatgg

55561 tgactttgtt ttatttaaag aggtagctga gttagttaaa ttaaaagctc atttaactaa

55621 agaaggttta gaaaaaatag agcaaattaa atctaattta attgttaaca cctaaagtat

55681 acctcctaca ttggtttata gtttcttttt tattttttgt tttcttatat tctttaaaag

55741 ataaataaaa aaaaaaatag gtgattatag ttcaatcggt agaacagcta gttgtgatct

55801 agcatgttcc ctgttcgagt cagggttttc acccttgtga tacagtaggt tcgattcaga

55861 gtattttccc ttaggtgtat atatatatat acatatagct tgagtagttt aaccggttaa

55921 aaccttattt tcatacgata atgatgggta ttcgacttac ccctcaggct aatcattttg

55981 atacatataa ctgaaaaaga ataaaattaa ttgactatta taatagccgg ggtagtttaa

56041 gaggtaaaac tttaattcca catgttaaaa atgagaattc gattttctct cttggcttac

56101 ttgtatatct atatatatat ataagtatat taaaaaaaaa tgcttttaat ttcaatatta

56161 tctttattac tttctaatgc cgttacaata cgacgagata tatcaatact ttttaataga

56221 gttgctataa ttgctttaat ttattctata ttacatagtg taacaacttt atttatttta

56281 ggtaaaggta taggtttaca cggaggttta cttaatgtta ctagtattac acaagttttt

56341 aatattttta tattcctagt aagtatatta atattacaat taactagttt ttttcctaga

56401 aaagtatgaa taccagaaca ttcttcacta atgcaattat tgtttaataa tttagtatta

56461 tatagaacaa aaattataaa taaaatggga gaacacttaa gaattataga atatccttta

56521 atactattat ttataattag tggagcagta tttttaatat ctactagtga tttaatatct

56581 gtttttcttg ctatagagtt acagagttac ggtttatatc tactaagtac tatatataga

56641 aattctgaac tatctacaac cggagggtta atgtatttct tattaggtgg tttaagttca

56701 tgttttatcg tgcgcctctg cgctttatgt tattatgtat tcaaagaata tagatacttt

56761 tccaatgtat ctccggcaac agagttaggc aaaggggaaa tcctaatgtt gaacatagca

56821 tctctgctta aaggtaggaa cggacgaaac cagattagtc taagaaccta tcgagccgaa

56881 tcacccatga gaagagctat actgcccgaa ctccattcac taggggcctt aacatgaggg

56941 ctaaatcact tctgggatag gatttatgat ctgtgcataa ctttgagatg aagagtattg

57001 caaggaaaca gattcaacct gggtacaata agtactctca acgtgaagag tggagtctct

57061 aaaggtgata taaaaataac attgagaact gggggattgc ctacagcctt aaagggccat

57121 ggtaacagag gagtcgtagt actgcttgca ggaagggctc cagcttctgg tgtctgtatg

57181 atgtcgactt ccgctgatgg tatcagtaca gtttcaactg acggattaaa caaaatacag

57241 aagattaacg aattatgtag cgaaaataaa agttttattg ttacggacaa actatacaat

57301 ataatgtaca gtaaggacgt attttcagca gcatatcaca agttgaaaag caaacctggg

57361 aatatgactc ccggtattgt accaacaact ttagatggta tgtcagatga agaaatcacg

57421 agaatcatct ccacactaag agatggaagt ttcaagttta accctggtag aagagtgtat

57481 ataccaaaga gcaatggggg cgaacgtcct ttaacaatcg cacctccaag agataaatta

57541 gtacaagaag taatgagaat gatcttggag gctatttatg aacctgcatt ccagaaatgt

57601 agtcacggat tcagacccaa caaaagctgt cacacggcat tgagagatgt tagacagaag

57661 tttggaatgg cgaaatgatt catcgaagga gatatatcca aatgttttga ttctgtggac

57721 cataacattt taatgggaat tattagttca agaattaaag atcaaaggtt tctagacttg

57781 atccgtaaag ccctaaaagc aggatatatg gaagccagag aatactcaca ttcacttgca

57841 ggaaccccac aagggtcaat aattagccca atactggcta acatctacct agataaacta

57901 gacagctttg taatggaact gaaaactggt ttcgacagag gagtaaaagc ctctatcaat

57961 ccagaataca aaagattatc tagtagaaag gacagagcta aagatacact aatgaagcgt

58021 atgatcaatc tcataatgct gaaaacagca tccaaattac acattgaccc caacttcaaa

58081 aagttggaat acgtgagata tgcggatgat tgaatcatag gtgtcagagg atccagaaaa

58141 gactgtgaag tcttgattat ggagataagg gatttcttag ataaaaacct caacctaaga

58201 ttgtcggagg agaaaactaa aatcactaac gcaagtaagg atttagctac cttcctatcg

58261 gtgggtatcc aacgtaaaac acacagaact ttacgtagga tggtacaagg tcataatcgt

58321 agaaatgtga acaacttacg tcttctagct cctataaaca aagtaacatc caaactgacg

58381 gagaacggat tcatgaaaaa tggaaccccc tatccaaaat ttagatgaat gagtaacgag

58441 aaggatacca ttatcctact atataactcg gtttatagag gaattatgaa ctactacaga

58501 tttgtagata attacaacaa cctatcctct aaagttcact atatactgaa aaactcttgt

58561 gctagactat tagcagccaa atataagtca acccaagcta aaatatataa ggagtatggt

58621 aagaacatga aaggtgacaa caaacacgga ttcatagaaa ttatactagg aataaaacta

58681 gccgcgttta gcccaaaaac taacgacgta ctctttagat tcaacgcgga aggtatctct

58741 aaaacctctt tggaagacct aagctgctca gtctgtcaat ccgaataccg tgtcgaaatg

58801 caccatgtca gaatgatgaa agacctggat ccaaagaaaa gtctagtgga tagacttatg

58861 atcaagagaa gaagaaaaca aattccccta tgtagaagct gtcatatgga actgcataga

58921 aaggataacc gtgataacaa taaaaacttc aaaaataagc gaaaataatt agaaaatcta

58981 atgccgtaag tggagagccg tatgatggga aactatcacg tacggttcgg gaaaaggggt

59041 gtgtcatggt aaaagtgacc catctctagt tcatttatta ggtacaagtt tattatacgc

59101 taattctgga actactaatt tagatgctat atatgctata actagtctta gtgatagctc

59161 agatatatga tataaacctt attacattaa ctttgctttc cttatattta gtataggatt

59221 tttatttaaa gtaagtgctg cccctttcca tttttgatct cctagaaaag gacttgggaa

59281 atccttaaaa acaaacgttg ggagtaaact accaaattcc ggggaacccc taaaacttct

59341 ggtactaaac catatatgaa aatatatgag tggctgaatt aattactcag gtatagtaat

59401 aagccagaag gtgagtgaaa acgaaatggg caatcgcgga tctaagtcag taatacaaac

59461 gaggaaagtc gagtattatt gtaaaagagc aacgagtaaa tggtagttga tgtggtatta

59521 atttatcaca tttaagatgt actctactgg gcttcgaaag aagttatcat gttaaaatcc

59581 tttctaacca aattattcaa aaacaatttt attctactgt atctaaccta aaatttccta

59641 tagatccttg atttatatcg ggattctctg atgcagaagg atgctttcta gttataatac

59701 gtaaaacaca aaaaaaccag ttaggttgac aattagaggc taattttaca attaaccttc

59761 attctagaga cctagacctt ttaaaactta ttaaaactta ttttggagta ggaagaatag

59821 gtaaagaaag aaatggttgt tgtgatttta caataggttc tttagatcaa ataataacaa

59881 aagtaatacc tcattttgat aaataccctc taaaaactaa taaatattct gactatttat

59941 tatttaaaga agttgtaatg ataatgcaac gtaaggaaca tttaacagtt gaaggtttac

60001 aaaaaatagt taatattaga gcttctctaa atagaggatt aacgccttta ttatcagaag

60061 ctttccctaa tagtattcct tttacaagac catcattacc tgtacttagt gataagttag

60121 accctcaatg ggtagctggt ttcacaagtg gtgatggttg ttttaaaatt agtattagag

60181 agtctaaatt acataaagca ggaagtcgtg taacattact ttttatagta actcaacata

60241 ttagagacga attactatta aaaagtttag tagatttctt tgggtgtggt caaacttatt

60301 cttataaaga ttatattgag tttagatgtc aatcattcaa agataactat gaaaatattt

60361 tacctttttt caacaaatat cctattcttg gtgtgaaatc tttagatttt gaagattgag

60421 ctaaagtagc caaaatgata caaacaaaag ttcatttaac taacgaaggt tttgatcaga

60481 ttcgcctaat aagaaaagga gttaataaag gtagatatat aaaataaaat tgttttttct

60541 ttaatttgaa cgataaattt aatctatgtc atattggacg tttatgacgc tatacctaca

60601 atagttacta cttttgtagc aattgtagct aaaatatcta tatttatttt cttattagaa

60661 attgtttatt atactaaaaa ttattttaca gattttaatt gaacatatgg cctactaata

60721 agttctttct tatcattgat aataggaaca gttgtaggtt taactcaatt tagaataaaa

60781 agattatttg catatagtac tattagtcat gtagggttta ttttattagc tttaagtata

60841 tctagcgtag aatctactca agcatttata ttctatttaa tgcaatattc tattagtaat

60901 ctaaatgctt tcttaatatt agttgccata gggttctctt tatattgtta tattaataat

60961 aataaagagt ataaagatct tttagataaa aataattccc caatacaatt gattagtcaa

61021 ttaaaaggtt atttttatat aaatcctaca ttagccttaa gcttaacaat tactcttttt

61081 tcttttgcag gtatacctcc tcttgcaggg tttttcgcta aacagatggt attaagtgca

61141 gctatagata atggatatat atttattact ttagttgcta tatctactag tgttatagga

61201 gctgtttatt atttaaatat aataaaagaa atttttttct tttcacctga atataaaata

61261 aatcctctat tagaaaattt aaattttaat ggaaatatat ataacaataa aaatgtatta

61321 attaaatcaa ttaactttaa atataataat atagcaatat ctagttctat atctattaca

61381 atatctatta ttacattaat aatattatta tttatatttg taaataaaga atgattaagt

61441 atgagtacca tatattgcct attcttctta tgcgctgagg tacattttgt acgtaattta

61501 ttgctgttat taaataaaaa agaaccttta agtagtaatt attcaaaaaa tacttacttt

61561 ccgcaaggtt tatataatac ttgtgtttca aatttgccta ggtctttata ttataaagtt

61621 tcccttcgta gttattcaag ctacgcttcg aaaaaacaaa tttaccctgt ttcaccttga

61681 tttatatcag gatttacaga tgccgaaggt tgttttaacg taggtattca aaaaaactct

61741 aatggtaaat attatgttaa accttctttt caaataaaag ttcactcaag agataattta

61801 ttattaatga gtataaaaca ttatttagga aatataggta atatttacat atcaaatact

61861 gattcaaatt ttacggtaaa atctttagct gatcttttaa aaattatttc gcattttaat

61921 aattatccct taatgactaa aaaaaaagca gattttttac tttttgaaga aatagtgtct

61981 aagatagttg aaggagaaca tttgtctgcc aaaggtttac aagaaattgt aaatattcgt

62041 gcttcaataa atttaggttt atcccctgct ttaaaaacta attttcctaa cactatacct

62101 gtagctagac ctgatataga aaagatagat acaattcatc ctgaatgaat gtctgggttt

62161 gtaacaggtg aaggttgttt tttagttaat ttatctaaat atggtaaaga tagattagat

62221 ggggtttcac taagcttcaa ggtatctcaa cactcgagag acgagttgtt gttaagatct

62281 tttatttctt tttttggatg tggtttattt aattatcatg ataaaaataa gaaagctggg

62341 atatttattg ttagaaaatt ctcagatata tatgataaaa tattaccctt ctttgaaaaa

62401 catcaaatat tgggtattaa aaaagaagat tttgaagatt ggtctaaagt agctaaatta

62461 attaactcta aggatcattt aactgaagaa ggtttagaaa aaattcgtaa aataaaatca

62521 ggtatgaata cactaagata atattatctt ttttattgta acactcaacc taaatgttat

62581 aggtcgctcg gtaaatgccc tttttaaggt catttataaa aaacattgga ttaatagcaa

62641 gaatatcaac accctcggtt agtttgatga tttgctgcga aatttattta tatttttatt

62701 ataaatttta taatgatata gtataaatta aaaacgttca acgactatta gggaagttat

62761 aataacaacc ctatataaat ccaatactta ttaatttaaa ttgttaaaaa taaaaattag

62821 taaggaaaga catagtctga acaatatagc gatatattga tttataaaat tagttagtat

62881 atatattagc taatttttta acacctttga ccatattggt acaatattta tttaattatt

62941 aaatgagtag tatgagtata ttttttatat ttgtaattat aatagcaata ctttttttag

63001 ctttaaattt aatatttgca cctagtaatc cctaccaaga aaagtattca gctttcgaat

63061 gtgggtttca ttctttttca caatctagat cacaatttaa cataactttc tttatatatg

63121 ctttagtttt cttactttta gacttagaaa tattattatt atacccttac gccgttagtt

63181 cttattctaa tgatatttat ggtctaataa tagtattaat atttacaact atagttacta

63241 taggttttgt atttgaatta ggtaaaggag ccttaaaaat aggtagccgt caagatgtgg

63301 aatctatcag taaaaaaaat actattatta tatctttaat aggaacaaaa taataaattt

63361 atgtaaacga ataactataa tattatgtta atatatatat atatgtatac atactttata

63421 agtatacact atttacttta tgatattatt caaatatatt tagtatgata caagttgcta

63481 aaataatagg tacagggtta gctactacag ggttaattgg agctggtgta ggtattggtg

63541 tagtttttgg tgctttaata ttaggtgtag ctagaaaccc ttcaatgaga ggtcaactat

63601 ttgcttacgc tattttaggt tttgcatttt ctgaagccac tggatcaaat ggttcagtat

63661 aaatcgagtt aattgcaaga agtatctaat tgattaactt gcagcgaaaa tagttatata

63721 actattacgt acaacgacta ataattcggt agttttatac taaagacata gtctgatcaa

63781 taaagtaatt tattgaacta acaatactaa taataaactt caatgtttat gtacttttaa

63841 aggtagtttt attacttgtg ttatcgataa aaaaaaaaat ctaacctatt ttattattga

63901 tatagtatat tatagtatta ttatcttttt cataaaataa caaaaaaaat gaaatatatc

63961 cgtatttata acaattttat attaacacgt gcatttagct ctaatactaa taatatttac

64021 gactatgcca taaaaatata taaagatccg ttacatcaac gtaaattagt acgagatgaa

64081 aataatggta aggtaggtgt atattgctga ataaataata taaacggcaa ctactacata

64141 ggtagcggcg atcctttata tttaagaata agtgattact ttcaagattg gtatatttta

64201 tctagaacta gtttatacat agttagagct ttatctaagt atggtatggc taatttttct

64261 ttagttatat tagaatactc gaatacagat gatgttatta aatgtgtgct ttgcacaaaa

64321 atgaatagat tttctaaaac cttcttataa ccttagccct acagcaggaa acacaaaagg

64381 ttataagcat agcgctgaaa gtattgaaaa gatgagatac aaggctttag gtagaaaaca

64441 cacagaagaa gtaaagcaat ctatgagcga atctcgtaaa ggagaaaata atccttttta

64501 tggtaaaaca catagcgagg atacaataga gttattaaaa acaactgcag ctaatagact

64561 taaatctcct gtacctggta tagaagtgga aataacagat attgaaacta aattaactca

64621 tacttgcgag tctatacgta aagcagctag ctttatgggt tctgatatta aaactatatt

64681 acgtcgggaa gaaacactaa aggtattaat actccctata aaaaaaagta cataattaac

64741 ataaaaagaa gttaatattt agtaaatgta tttgctttaa tgatggcctt tttattatta

64801 tacgtagctt aataagatat aaatcttaat gttatcactt aattctatat aataaaaata

64861 tattagtgga taatatatta tatatagaaa aacaaaaaaa attaaaaaaa aatatatgta

64921 ttactttttt atcaatttaa tgattaaatt agacgcacct agcgcttgag gaatatattt

64981 ccaagatagt gcaactcctc aaatggaagg actagtagaa ttacatgaat ttttgtgtat

65041 aagagccaaa ctccggggac accttaaagt tctaataact aagtttataa gggaaacttt

65101 ttaaatggcc cagctaatca ctgtgggtat agtaatatca ttagaaatag aagaatattt

65161 ttctgaaaat aggctatcgc ggatctaaat caaatatata tttatgtatt tgtaaaagag

65221 caacgagtag acggttcttc gatgttaaac ataatatcgt aaggtgtact ctagtcgctg

65281 ggaaaccagt ttttgagaga aaatttaact cttattattt tttttttatt attataattt

65341 atggacataa taatagctta atagcataaa aacgcggagc tctcaattag attaacaacc

65401 tattgatctt gatttaatat tattcagttt aaatataata tttgtataaa ttaataatat

65461 taaataaata tatggaaaat tataaaaata aatattcagg gttaaaccca cattatgtta

65521 caggattctc agacggtgaa gcttgttttc acctagccat aggtaaaaat tctaagtata

65581 aaataggtta ttatgtaaat ccagggtttt cgatagtttt acataaaaaa gatgaacagt

65641 tgttaagaaa tattcagagt ttttttggtg gaataggtaa tttaaaagta aaatctaata

65701 tagtacaata tagaattttt tctttaaaag aattagatat tctactagaa cattttgata

65761 actacccttt aataacaaaa aaatttgtag attatgaatt atttaaggaa gctttaataa

65821 taatgaaaaa caaagaacat ctaacaatag agggttttaa taagattata gctttaagag

65881 cttctatgaa tttaggttta ccggaaactt taaaagaagc atttccagat gttaagataa

65941 gagagataat acccaaggat ctaccatctt tattaaaccc ttattgagtt gcaggattta

66001 cagatgctga aggttgtttt tgaataaaaa ctttaaaaga ttctactaaa aacaaagtaa

66061 gtacagtttt caaataacac agcacagcag agacttgctt ttattgaaaa aattaattga

66121 attttgtggt agattagaac aggtaggttc agcttataat ttggttgtaa ctaaattatc

66181 tttaataaat gaaattataa ttcctttttt taaaacgtat cctattttag ggattaaatc

66241 taaagactat aatgattgat gtaaagcagt tgaattaaaa aacaataagg cacatttaac

66301 acctgaaggg cttaaagaaa tattaaaaat aaaatctaac attaatgctt ctagagatta

66361 aattaccggg atacatacga atttttactt gaattggctt atctggacct ttcataataa

66421 tagtaactca aaattaaagt tactatattt taattaaact atgagaatag tattaacaat

66481 atagacggac aatataatgt attatttagt tataatatta tttggtgtag gatgagtttt

66541 attatctatc actaaaaatt ttattattag tgcttctccc atttcacata aatatttaaa

66601 ccatggtaga tatgtgccta ctcgtaagta ctttaaattt aatatattaa ataaaatgcg

66661 tttatatact acaatacccc cttctggtat aaagttttat gaaaatgctt atttaatgag

66721 aagattaata ataaacgaaa ataaaaataa gtctggaatt tataaatgaa caaataaatt

66781 aacaaatgat atttatattg gacaatctac aaatttatct agaagattta taagatattt

66841 taattatagt tatttaaaaa tttgggatac tcctgcaata agtagagttt tagttacaca

66901 tggatatatt aacttttcat tggaaatttt agaatattgt aagaaatttg acttgagaga

66961 aagagaacaa tactatattg ataagctaaa ccctaagtat aatactctaa atatagcagg

67021 tagctcttca tattataatc acattgaaga aactaaaggt tcccatgttt taattattgt

67081 aaaaaatggt ttagaaagac ctaaattaat aggtagtttt ttatcaataa gaaaagctgc

67141 cagattctta ggtattagta gtaacacaat tagattctat ataaactcag gtaaatgaat

67201 tagaggtaga tacaaattaa tttcaaatta aattaaagaa actgcgagtt aaatttcact

67261 atatgctgga aacccctaaa gcctttaagt actatatatc attaaatatt atcagtgaaa

67321 atcttaaagg atgtaacaat ggattatcag caggaaactg gtagattcct atatctaggc

67381 aggaccctca gagactacac gtgaaaacta tatatataca tatatagtaa gatatagtcc

67441 gattaggtat gaaagtactt aagttttcga cattaatcga gttaatatga actattactc

67501 ctgctgtgat attaatatta atagctttcc cttcatttaa attattatac ttaatggatg

67561 aagttagtga tccatctatg tctatattag cggaaggttt catcatttgt ggccttaaat

67621 tgtttatact atatgttttt tataaaataa aagatgccag caatataata aatatatctg

67681 gagaaaaaaa taatacttat atatataaaa gactagctca agtcaaatat attggttata

67741 attcgtacgc taaaaatgca aggttcaccc tcgaattaaa aagagcattt catactaaaa

67801 ttagagcttc tagtagaata ggtccccatg ataaagatgt gttatctgtt ataataggtt

67861 ctttattggg agatgcttat gcaaatgcta ggacaataga gggtactaga atatcatata

67921 gacaaagtaa tgtacataaa gagtatttat tttgattata tgactttttt tttcaaagag

67981 gttattgttc aaatttaaaa cctagaaaat acacaagaac attaaaaggt aaagagtttt

68041 atggttatga atttaacact tttactttta gaagttttaa ttgaattcat aaattatttt

68101 ataaaaaagg aattaaatat ataaatccta atttagaatc atatctaact cctttagctt

68161 tggctatttg aataatggat gatggtactt gagcaggtaa tggagttaga attgctacta

68221 actcatttaa atttgaagaa gtaaaaatat taggaaatat gttagtaaaa ctttatggtt

68281 taaattatac aattcaaact atagaaggta gacattcaat atatataaca aaagagtcta

68341 tacctaaatt aataaattta acattacctc atataatacc tagtatgaaa tataaattag

68401 gtataaaaga taaccaataa gtataagttc ttttaataaa catataatat atccaatagt

68461 cgtgatctta gtagaaagaa gttgctaagt aatagagcca tatttagtta aacatataat

68521 tcaatctatt aatttctaag aacccgacag ggatcttaaa gagaaatact tttctctttg

68581 gcgatgcgag tgaaaacagt taacatttta ttattttagt atcgccttta gtcctaggtt

68641 taataataaa acaagactgt cagtatataa tttgtttttt tatatatatt gcaacagact

68701 gggtcacttg tgggtggcta attttagctg cttaatgtac agtcgagaaa ttataaaaat

68761 aatttagcac ctagggtact tttttggatt tacggtttta acttatatta cgtatataaa

68821 cttaaaatta tttgacccat taaaacataa agagcatgtt tatgcgtatt ctatacctac

68881 gagtgcctta agtattcata ctaaatcatt taaaaataaa ctacaaaacc aatttgtacg

68941 tagttattct acaaatatta ataaattaga cccttgattt attacagggt ttacagacgc

69001 ggaaggatct tttatgctta atttttctaa atcatctgaa tctaagcatg gatttagaat

69061 acgtggtatt tttcaaatag aattacatga aaaagatttt gaacttttaa aaaccattca

69121 atcatttttc ggtggtatag gatatattac tgctgcaact aaaaattgtg tggcatttag

69181 agcacgttct atagaagatc ttcaaattat aattgctcat tttgataaat atcctcttaa

69241 aactaaaaaa cgagctgatt ttgatttatt tagaatcgct gttaataaac tttctttaaa

69301 agagcactta aaactagaag ggtttaaaga aattgtaggt atcagagctt ctatgaataa

69361 tgggttaact gataccttaa aatttgcttt ccccgatgta accccagctc ttagaccaac

69421 tctgtttgat gaattaggga taaactctag ttgactcgcc ggatttgttt caggagaagg

69481 ttgttttttt attaatttag cgacttctgc atcaaataaa ataggttata gaatttcttt

69541 attatttact atctctcagc atactagaga tagagagtta atgaatagtg ttgttaattt

69601 tttagagtgt ggggatatta aagaaagttc tagtagagtt aatattttaa catataaagt

69661 tactgatttt tctaagatag ttgaaaaaat tattccgttt tttagagata atagagtttt

69721 aggtgttaaa tctgatgatt tttcatcttg atgtcaagct atagagctaa tgaaagataa

69781 aagacactta acagaagaag gtttaaataa aattagatta ataaaatcta atatgaataa

69841 aggaagagtt ttagatccag aagcagaagt atagttatta ttttttttag gtttacatga

69901 atattttttt ttacataatc ttaagttaaa taatattttt atttttaaag taatttaata

69961 tagatgaagt aaataaatat gtttataatt tcgcaccaat gatactgaag ttatgcgtat

70021 ccagatttct tagattctag tgaagaattt attgaatttg attcttacat tgttccagaa

70081 tctgacttag aggaaggtgg tttaagaatg ctagaggtag acactagagt tataatacct

70141 gaattaacac acattagatt tatcattaca tcaggagacg ttatacattc ctaaaaaatt

70201 taaaagagtg tatttaaaga atcaaactcc ggggaaaccc taaagcttta gtgaccaaag

70261 taataaggaa acctattaac tggcctagct aatcactcag ggtatggtaa tactactaaa

70321 gatgaaggac aattttgttc ctaaatgggt aatcgcggat ctaagtcagt aatatttata

70381 agtattattg taaaagagca acgagtagac ggttcttcga taataattag tattattgta

70441 aggtgtactc taatcgccgg gaaagccggt tcttggaaga aattaagtaa atcaagatta

70501 aagaaattaa attttatatt aaaattcgat tttgtttttt gaatttttat ttatttctat

70561 tttagtttat aaatttactt atcataattt ttatgtttat agaattaaaa gttatactgt

70621 caattttaac aacaaaaaat ctttacctaa taatacaaac tacgatagat ttaataatgg

70681 atctcgtgta gcaagttcat ttttacaaaa aaggttttat tcgtcatctt ctaataaaac

70741 tctttataaa gactatgaat ctataactag tgatgtatta aactctttac tagaaaaaca

70801 acaaatatct attagtaaag aagaattaaa taaactaata aagattagtg gagttgtatt

70861 tgatttacct ttagatgaaa taacttatcc gtctttcgaa agtttagtgg gtagacctaa

70921 tactagaaac cctaagaagg gtgtttatat attttctcat aaaaaatcag ggtctaagta

70981 tgtaggttca tctaatagtt tatctagaag aatggatcag tattttactt ttaagcattt

71041 taatcaagag aattcaggtt tactactgcc tttattaaag aaagatggat ttgagtcatt

71101 taatttatca atctttgtta aaatttaagt gtatctccta taaattctga ttacttttat

71161 ttatttcttt aacagtatta tttattgcat gaagaattta accttaacac tcaaagaata

71221 gttaatttta gagttaacca aggcaaatct atttatctat atgatcttga aggaaagatt

71281 ttatattata gtagtaattc tttaaaaaaa tttatggata ttttacgtac tcaccatgaa

71341 acatgtacta attgtataaa aacaggtgaa agttatttta aaataacgga tactcctatt

71401 ttatagcttg ctagcttgct tgcgcagcgc tgactaaaag ttagcagcgc agtgcaatcc

71461 agtccaattt aagcctacct caggtattag atttggtagc tgataaaaga aaagatggtt

71521 taaagaaaac cactagtata aaatttagta aagcaactca ggtaagaaag gtaaacgagg

71581 aagatgtttt agtagtataa cagaaacagt taaatatttc aacgacttaa atttaaaact

71641 agatagaaat aaaattagca aagtattaga tacggatgaa ccttataaag gttatatctt

71701 ttcaaaatca actgcttaaa taaatttaat atcatgtctt ttgcttgtcc atctttaggt

71761 attaaaactg atgcttatcc tggtagatta aatcaagttt ctgtttttgt taatagagaa

71821 ggtgtattct atggtcttaa ttaattatgg ccatgactgt agtgaaccta tggttataaa

71881 tcatcaaatt gcgggaacac cttaaagaaa tcttaaccaa gtaagtatgg taacataact

71941 tatggcacag gtaatgactc gtggtacggt aaaatcaaga tttattatac aataggttat

72001 ccgcagccaa gcatcaacta ctgttatcag caaggatgtg cagttcatcg actaaatggt

72061 gattagtgtt atagtttaag gcacaagctc ttaaatataa tgcttaagat atagtcaggc

72121 cctacctgaa aaggtacagg aatattaatt tatattctgt ttattagatg tatatttata

72181 gacgcaatca taaatatatg tttaggggaa aatctttata atagtttgct aaacttatta

72241 ttttaatgta aaactaaata atgtattctt aacattaagg tgaaactagc ctagattctt

72301 gtaatatttt aatgcataat atgattgata aaaatatttt taataatcgc tttttacaac

72361 tctgtcataa gagttctggt ttagggttaa tagctactcg cagatgtttt agtacaagta

72421 ctgtaaaagc aaataaattt aaccctgaat ctttagcctt agatcatatc aatagtaata

72481 aacctacaac ttcttcagtt atcaataaaa tattgttaaa tcaaaatata tctttaactg

72541 aatccaaatt agaagaatta ttaaaagtaa atggcgtaga attagattta cctattagta

72601 catctgaaga taaaaattta ttatctgaat taactggaaa atctaattat aaaggatttt

72661 ttggggtata tatgtttata cataaaaaca caggggataa atatgtaggt tcatctaatt

72721 tacttagacg tagattggat tattatttta aatctttaga gtcacaagct gcagatttat

72781 cttatacagg taaattctta ccctttttaa aaaaagaggg attaagtgca tttaaattaa

72841 taatctttaa attggataat aaaaaattta atgttaaaga tgctttgatt ttagaacaat

72901 atcatttatt aaataaagaa tttaacctta atactttaag agtggttaat gctggatcct

72961 caaaaggtga aagtatatat gtttatgatt tatcttgtaa aattctttat taccaatcta

73021 gttccaaaat tggattaaaa agggtattaa aaatacatcc tgaaactgtc gcaaaatatt

73081 tagattctaa aataccgtat cttaagaaat ttttattatt aagtttccct atacccggtg

73141 tgtcttttag tgatatgtct gttacaaagt tgttagaaat gatgcaagaa gaaagaaaaa

73201 atttatatat attaggtaca cgtagaagta tttctgtttt attagaaata aaagaaggaa

73261 atacaaaagt agctgataat tgttgaggtc aaactttaaa ttttgaatct ttaacttctt

73321 gtattgagta tttaaaaaat atagggttat atataaaaag agatacatta tctaaatata

73381 taaagactgg gaaagagttt cataaatttt tatgtagata ttcagataaa aatttaccta

73441 gtaattttga agaaataggg ttaattctag atgagtacaa acaaactctt gcttactttg

73501 atgtatattt agctaaagaa aataaaaaaa ataaaccagt gttagtaaaa agtgaaaatt

73561 ataataatga atttaatagt ataacagata ctattaaata ttttgactcc attaatgtta

73621 aattagatag aaagacttta tatttacgct taaaagatgg taagccttat aaaggttatt

73681 actttggtca caagctctaa atatttttcg atctatagct tcgctagcct tacattaaaa

73741 attataaaga aaattcgcaa tgttcagaaa tatgtggtat attacatagc tcaatgccta

73801 ttgttattga atcagtatct ttagaaaaat tcttaacatg acttgaagaa caataatata

73861 aatttatata tacatataaa tttatataaa ggtgtgttag cttaattggt taagcgtagt

73921 actacggatg ctataaatat gagttcgagt ctcgtacaca tctctcttaa gcatacttaa

73981 gaaaatatgt aaatatatat acatatatat atatatatat atatatttat taaataatta

74041 atttaaaaat atatttatta tgaatataac cttaatactt tttttaatag gaattttagg

74101 atttgtttta aatagaaaaa atattatatt aatgcttatt tctatagaaa taatgctttt

74161 atctataaca ttcctaatat tgataagttc acttaatatt gatgatataa taggccaaac

74221 ctatgcaatt tatataatag ttgtagcagg agctgagtca gcaattggtc taggtatttt

74281 agttgccttt tatagattaa atcgttcttt gttttcttac tatctatcct acaattacac

74341 tccaatgtct ttttataaaa aaatccctca acaaataaga tattactcta ctaatgccta

74401 tgacagaaat ataagcaatc attatataga cccttgattt attaccggta tttttgatgc

74461 ggaaagttct ttcgtagtaa ctgtattaaa aaaccctaga tataaaacag ggtgaaatgt

74521 tcaagctaga gttcaaataa aaatgcatga gattgacaga gttctaatag aaagcattag

74581 aaactacttt gggaatatag gttatatatc taaacctaat aataaaaatt taacagttga

74641 atttagaatt agtacattaa aggatttaat tgatataatt atacctcatt ttgataaata

74701 ccctctaaaa acaaaaaaat atatagattt cttattattt aaagatattg ttctattaat

74761 gagtaagaaa gaacataata ccatagaggg tataaaaaaa ataatttata taaaaacatc

74821 attaaacact ggtttaactg aaaatttaaa agaagcattt cctaatgcat tccctttaaa

74881 taatttagat tataacaata gaccggtagg gcctatacac cctaaatgaa tggcagggtt

74941 ttgtacagga gaatcaaatt tctttataac agttcaaaaa tctaacacaa aaacaggttt

75001 agctatatcc ttgaggtttt caattagtca acactcaaga gatttattat tactagaaag

75061 ttttgttaaa ttttttaatt gcggatatgt gtctaattat aaaacccgtt tagtaactga

75121 atttattgtt actaaaattg gtgatatata taaacatatt attccttttt tcgaggaaaa

75181 tttaatatta ggatctaaat attcaaatta tttagatttc aatcaagcag ccaaaataat

75241 aataaataag gaacatttta ataaagaggg attagaaaag attttatttt taaaaaataa

75301 aatgacaaca ttttacaagg aaaaattgta aataatcata gataagacag ggcccaaagg

75361 acatatgatc aaggtgaagt aaaccttcat ctagttttcc tagtgaaaca aactatttcg

75421 ttatagttag caaatctagg taagcaaaac cgtcaaattg cgggaagttc ttaaagacaa

75481 atctaccaaa ttattatagt aatataataa tggaacaggt aatgactcgt tgtatggtaa

75541 aaacgatttg tatgaagaaa tgaaatagat aatccgcagc caagcgccta ttacttaaaa

75601 caagtaaggt gtgcagttca tcgactaaat ggtggttggt gttattatgg gtaaaataat

75661 aataatgctt aagatatagt caggcataat ttaatagtta tggaattacc ctagccatcc

75721 taaggaaatt gtatttctat ggaaaagggg gaaaacgaag aggaagtatt gcaatagaat

75781 ataaataatg tatttaagta taataatttt acctttatta ggatctatag tttctggctt

75841 ttttggtaga aaagttggag ttagcggtgc acaaattata acatgttcaa gcataacaat

75901 tactactatt ttagctatta tagctttttt tgaagtaggt attaataata ttgtgttgtc

75961 tattaattta tttagatgaa tagacagtga atgatttaat ataatttgag gttttcaatt

76021 tgatagttta acagtgaaag atatgggcct tttagaaggt acaaatgctg tgttggtttt

76081 aatccaactg tgtgagatgt ctattattaa tttttattta catatgttta tgtatccacc

76141 ttttagttca attactggtt ttagaagcta tattttaggg gcggagccca ctaaatgcta

76201 taaatctaat atatctgaac tgaaaatata cccccgtgtt aataataaac taaatatcgg

76261 tttatgtagc ccccgtagac aatattcaac tttaaaaaat agcaatatag tcgcaggagg

76321 ctctgaggaa aactttcaat ctagcataaa attgccttct gattctactt tcttacaatg

76381 atttgtagga tttaccgatg cagaagggaa ttttattatt aatcccctaa aaaatactaa

76441 acttgatatt tctagatttt catttatgtt taaaatagct ttacataagg acgatagtga

76501 agttttaatt tatattaaag ataagttagg agtaggagga gttcgttact ataaagacga

76561 atgtatcttt aatgttacaa ataaagaagg aattgcttta ttaatctcta tatttgataa

76621 atataattta aataccacaa aacatttaga ttttttagat tttaaagaag ctttttatct

76681 ttattgaaat agaaaagaaa attcagacgt ttgtttaggc ggagcctcag ccccttatcc

76741 tttggatcag aagcaagctg tatacagcgc agaaaaagaa aaaatattag atttaaaaaa

76801 taaaatgaat actaatcgtg ttgattttga tagacctaat aattcacccg ttcttattac

76861 gagatcttga ttattaggat ttattgaagg agacggttct ttttttatca gaagagatac

76921 tttaactcct acttttgcta ttgaagtttc aggagtacaa tttcctgtat tagttgaaat

76981 taagcaattt ttagagagtt ctttaggatt tgataaatat tctttattta aattaaaaaa

77041 ttcttctatt atatctgtaa atatgaataa acctagaaat aacagtaaaa gtagtacatc

77101 tattattatt aataatatta gtattttaaa taattatttc ataccctttt ttaatgaaac

77161 agagttttta acaaaaaaag gtaaagattt taaagatttt aaattaatat ctaaaataat

77221 ttatatcggt ggacatagaa aagaagatat aagatcttta atattaaaat taacaaatac

77281 tatgaataat tttagattat caactaataa agaaaaagtt caaagtttaa ctagtgaaga

77341 aatggattta cttttaaaat cttctcctac agttgaaaga ctttttgatg gtagagtaat

77401 agatagtagt actaaaaaaa tattacctaa attaaatagt tgtgtgtatg aaattattgg

77461 cgaaagaggt gatcaatatt tggctaattc tttaagcgaa gctgcttcaa ttataaatat

77521 atatcccgac actttaagta aatatttaga cattgaggct tcggcctcaa atttagaagt

77581 atttgtagat gttaaaacat acaaagtaag aagagttcgg gttttaggtt gctaaaggtc

77641 cttgctgcct cccgccctac taggtttcac cagaagaggt tgcgcggggt ggtttcattt

77701 ttaaggcgct gcgcggcgct gcgctagcaa gctagcaagc tagcaagctc ttaaatttaa

77761 aaaaataaaa attaaaaaat caaccgctaa agctctaaaa tgatgggcta ttaaatagtt

77821 tataattatt tactgtaaaa taatattagt atactggtat tgtaaggtga ggaggcggga

77881 ttattattta acacatacta catatatata aattaaaaaa aaaaaattat taatagataa

77941 cctttataag ctgatttgcg tagacggagc ctcagcaagt tcataagagc tcagggtaaa

78001 ccagaagtgc acttaaatcc ttataggtac atatttagga actacgccta ataatattac

78061 ggccgtaatg ggttctaatt tatttaacag ggctagtcct aaatatataa agaagaattg

78121 agcaagatta taatcaaaca attctatact cacataaaaa aggtgaaaac ggttaaaggg

78181 catactccct aagaccgtcg gcagttataa atgtcgctac agactggttc accagggtta

78241 ggctgaaatg tctgtccaaa tgtacagtcg gatcctagaa tgagtgcgat atcataggga

78301 ggatgtattt atcataacaa acactacgca tggtggatag ccttaccata tgggtaataa

78361 actcctgagt attaatttac ttctaaagtc aatactcaaa tatttgagag actttaattt

78421 tttgttaact aggattgatc tatgttaata cctgtattaa taattagtac attagttcat

78481 ttctattcta taggttatat gagtggagac cctcgagggt gtgttaaggg aaaacatacc

78541 tatgggggta aattgtcaaa ctccggggaa cccctaaagc ttctgatacc aaaccatgta

78601 tgaaaatata tgagtggctg aattaattac tcaggtacgg taacaagtca ggagatttgt

78661 gaaaacaaaa tgggcaatcg cggatctaag tcaaataaag gtaacacttt atttgtaaaa

78721 gagcaacgag tagacggcaa ttggtttatt aagtttaatt taataaactt aaggtgtact

78781 ctggtaggtt tcgaaagaaa tagtaagata tcttacggat taaactcttt taagtgttta

78841 aatccctgtg tcaaaatccc atctaaccaa ttcggtttaa caaaaaaata ttctacttat

78901 agttattctc ctgttaaccc tggcgtctga tcaggattaa tagatggtga aggttcattt

78961 agtataatta tagataaagt acaagcccgt aaattaggtt gacgggttca attaaaattt

79021 caactaggtt tacatgcaaa agattataat ttattatgtt tattaaaaca agatttgggt

79081 aacataggtt ctattcattt agctaaaaat agaaatatag ctaattattc aattgattca

79141 attgaagatt taaacaagct tatgacccat tttaaaaatt atcctttatt aactcagaaa

79201 gctgcagatt taatgttatt taaacaagct atagtacttg taaataataa agcccatctt

79261 actgtagaag gtttaaataa aataataaat attaaagctt ctatgaattt aggtttatca

79321 gataaattaa aatcagaatt tcctaatttt attccagtag aaagacctat aattaatctt

79381 gataatttaa atttagatcc ttattgaatt tcaggttttg ttagtgctga aggtaatttt

79441 gatgttcgtg ttcccgccac taatagtaaa ttgggacatc gtgtacaatt aagatttaga

79501 gttacacaac ataatagaga tattgaatta atgaacaaaa tagttgaata ttttaatgct

79561 ggaaaaatct ataaatataa tggaggatcc gctgttaata taacaatagt ggattttact

79621 aatattactg aaacgatagt tcctttttta aaaaaatacc ctataatagg tgtaaaattt

79681 tatgattata ttgattgatg taaaatccac gaattaatgg tgaataaagc gcatcttact

79741 atagatggta ttaattccat taaattaatt aaatcaggaa tgaatacagg tagagagtct

79801 taagttattt aaccaatgtt aataagaata tactataaac caattgtatg attaatttga

79861 caacaaatgc ataatcaaag attctttagt tatttaagtt tattcacatt tatgatgata

79921 atactagtaa cagctaataa ttatctatta atgtttgtag gatgagaagg ggtaggagtt

79981 tgttcttatc ttttagtgag tttttgattt actagaatag cagcaaatca aagttctatg

80041 tctgccttta taactaatag agttggtgat tgttttttaa caataggaat gtttgcaata

80101 ttatgatctt taggaaattt agattatagt acagtatttt cactagcacc ttatgttaat

80161 gaaaatatta taataataat aggagtttgt ttattaatag gtgctatggc taaaagttct

80221 caagttggac ttcatgtttg attaccaatg gctatggaag gtcctactcc tgtttctgct

80281 ttaatacacg cagctactat ggttacagcg ggtgtatatt tattaatgcg ttcttctcct

80341 ttaattgaat atagttctac tatattatta ctttgtttat gattaggggc tataactact

80401 gtatttagtt cacttgtagg gtttttccaa caggacataa aaaaagttat agcttattca

80461 acaatgtctc aattaggaat gatggttatt gctgtaggtt tatcttcata taatgtagct

80521 ttattccatt tagttaacca cgctttctat aaaggattgt tattcttagg cgcaggggca

80581 gttatacatg ctatggctga taatcaagat tttagaaaat atggaggttt aatatctttt

80641 ttacctttaa gctattctgt tattttaatt gctagtttaa gtttagttgc tttcccattt

80701 atgactggat tttatagtaa agattttata ttagaatctg cttacggtca atactatttt

80761 agtagtataa ctgtttacat tatagccgta ataggtgcta tatttactac tttatattca

80821 gttaaagttc tttatttaac ttttttaaca aaccctaatg gacctttaat aaattataaa

80881 catgcccacg aaagtgatat atttatgagt ttacctttag taatattagc tatattctct

80941 atatttttcg ggtttttaac taaagatata tttataggtt taggatctgg attctttata

81001 gataatagta tatttattca ccctaactct gaaataatga ttgatactga atttggggta

81061 tctacatatt gaaaattatt accttttgtt tttactgttt catttagtac aatagctatt

81121 atattatctg aatttttatc tgaaaatata gttaatttca aactatctac aacaggtaaa

81181 actatcttcg ggttctttaa ccaaagattt ttagtagaat ttttctataa taaatatatt

81241 actaatttaa tatttaaatt aggaggtcaa actgttaaag ttttagataa aggtagtata

81301 gaattattag gaccttatgg tttagaaaaa aaattgatta attcaagtaa aaatattagt

81361 agtttaaata aaggtattgt aacaaactat gctttattta ttctagtagg attcatactt

81421 tatatgtttt ctataacttt aggttttact aataatttat tcttaataat attactttta

81481 atttttatta ttagttcaat ttattaataa aaatttaaac ataaaaataa tttagtacat

81541 gatacataat aatacctata tataacggcc cttatagtta attataaaat aagatatata

81601 tatataaatg agagtcttaa agaataatag tatattaaaa ttagccaatt cgtatcttat

81661 agacgcttct caacctagta atataagtta tttatgaaat tttggttcat tattggcaat

81721 ttgtttagga atacaaatta taacaggtgt tacactagct atgataaatg tgtagctata

81781 aaatcttaca aattgctgga acacccttac atatatatag atgaaggtta atcagcaggg

81841 aagcttcttt gcgcatataa gctcaaagtt gaaccttcag agactagagg taagacatgt

81901 agaagttatg acgaaagttc ggtttaaact acgcatgatg gtatagtcca ccctttcgaa

81961 agattaagga attatggctt aagatttatt atttgttggc tttattaatt attgcttatt

82021 tagtaataca aaataaacaa ataagcaaaa taatttccaa tcatataaat ataaaaaata

82081 aattctctta ttctacttta tactccgcag ataaagataa accgttaaac ccttggtttg

82141 tgacaggatt ttctgatgca gaatcatctt ttgctatgtc tgtgtttaaa tctaaaacag

82201 ctgcaatagg ttgaactgtt gaaccttgtt ttattattaa tttacacata aaagatatag

82261 aattattaaa aaaaattcaa atattttttg gcgtgggctc agtctcaatt tccggagaca

82321 aggttgcacg ttatagagtc agatcaagac ttgatcttca aattattata tcacactttg

82381 aaaaatatcc tttacaaacc actaaagtta taaactttaa aagtttttgt aaaatattag

82441 aacttctaaa taataaactt cacactaatg tagaagggtt tttaaaaata ttatctttga

82501 taaataaact taataatcca ttatctgaag ccttacttga aaaattagca cctctaggta

82561 aaatccctgt cgtggattta gagatatatt cccctgaata tttagacata aaagctaaat

82621 taaatccttg atgaataaca ggatttgcga caggtgaggg ttcttttaca ttctttacta

82681 gaaaacgggt aaatgcttca ggtaatactg ttaaggatta cactcttgca tttgaaatag

82741 cacagagaag tgatagttta tatgttttaa gtttaatagt taatacttta ggttgcggga

82801 aagtatactc tgaagccaga ggaataagta aatttagatt ggttcctaga gacttaattt

82861 tagaagattt agtgcctttt tttgaaaaat atcccttaga gggaaataaa gctttacaat

82921 attctacatg aattaaaatt gtagaggtct tacataaaaa tcctagatct gactttagag

82981 aaaataaagt agaagcttta ataaaagagc tgtcaagttt aaataaataa caaattgcaa

83041 agccttttgg cattatagtc ccagcatatt agaagctttt aactctgtag agcatatctc

83101 gtaatgtgtt ctttaaatca aataaattgc tggaacccct tataatgata ttataagaaa

83161 atcagcagga aaattctagt aataaaatta ctggtagatc ttcagagact aaacatttga

83221 catttatatt aataataaat gatgatatag tccaaccttt actgagaagt aaagttaatg

83281 taagattata tatctatacc taattttcgg tataatttta ttatcattta ttgctgtgtc

83341 gggtattaaa aataaaatgg ggttatatag atattctaca ataagtatag ataattcatc

83401 tgatctttat aacttaaata atataaattc agagttttta tcttgatttt ctggttttac

83461 cgacggtgaa ggtaatttct taataacttt agatagaaaa tatattaaat taagatttaa

83521 aattaattta catatagacg atattaaagt tcttgaagtt attcaatcta aattaaatat

83581 aggtagagtc agatttgatg ataaaaataa caattgttct tatattgttg aagatgtatc

83641 agggttaggc ttattatgtg aaatttttaa aaactatcct ttacatacaa gtaaaaaatt

83701 agattttgaa aatttttacg aagcttttct tataagaaaa aaagcaaaat tatctgatgc

83761 cagcttaaat agaatactat atttaaaaga taatatgaac tctaaaagag aaatattttc

83821 atataataca tctaaatctc aaattataat agaccctaat tgatttatcg gtttcattga

83881 aggtgaaggt acttttggta ttaaaacagg atcttcattg tatttccaag ttgctcaaaa

83941 aaatacaagt caagagtgtt taaatggaat aataaatttt ttacataatt tatcaaatga

84001 tacaataatt cctgaaaata gtaaaatatt acctttacat gtaaccaata cgattaattc

84061 aaaaacaaat gttgtttcat tagtagtatc taacacagat tctttatatt actacatatt

84121 acctttacta gataattcta agatgtattc acgtaaagct atagacttta aattatgaag

84181 aatggcttta ttattaaaaa tacatggcta ttattattca attgaaggaa aaattttatt

84241 tttagatata tcaaatatcc ttaataaaag atatagtact aaaacaactg tggatataga

84301 caaagctgtt aatgaagtta tagaaagatt tcaagatatt ttgaaaaaag atcctatttt

84361 tgatgtaaaa ctttatgaat cacatgttga aaacgtgcga aaatatagca tcataaataa

84421 atcagataaa cctaaaactg tgtatatcta cacagaagag ggattagtag aagggtctcc

84481 tttctcatca tttagttcag cacataaaac actaggttta aatcctagta gtaatacatg

84541 taatcgctat atagatacaa atagatgata taaaaataaa tatatgttta catctaaacc

84601 catagataga gcgtctaggg attagattat agtagatata ttattctcta cttaatattt

84661 tgattatgag agacgtaaat aacgggtgat tagttcgtta tttacatagt aatactgctt

84721 cagctttctt ctttttagta tacttacata taggaagagg tatatattat gggtcataca

84781 gatcaccaag agtattagtt tgaacaatag gtgtagtaat ccttatactt atgataggta

84841 ttggtttcct gggttacttt acaatagccc aagatgacta taaaaattta tataatagta

84901 aaacaataaa aacattcaac aataaaagat attattccac atcccataat tctgtaagta

84961 attttttagc ctctaaaaac attaaacctg tttttgtata cgataatcta tctgaagatt

85021 cagttcgtag agatatagct aaggaaacta aagaccttag tggtatttat atgatattaa

85081 ataaagaatc tttaaattat tatataggat ctgcttctac agggagattt aattctagat

85141 ttacaaatca tttaatctat ttaaatggta ataaagttct taaaaattct gtaaagaagt

85201 tcggcttaca tagttttgta tttatagtat tagagttatt tcctgaaata gttaatcaag

85261 aaaataataa gaaattgtta gacttagaag acttttattt aaaatcactt ttacctgatt

85321 ataatatatt aactgaggct gggtctagct ttggttataa acattcagaa gtaacaagaa

85381 taaaaatgaa agctaactat agtgaagaac gtagaaaaga aataggggaa ttaaacagag

85441 gaaagacttt ttctaatgaa actatagagg ctatgagaca atcagcttta aatagaaaaa

85501 agataaattc cacagaagaa gcgattttaa atatgaaaaa gcgttctaaa cctattttag

85561 taaaagagct taataataca gtttatggtg aatttaatag tatagttgaa acagcagaag

85621 ctttaaactg ttcaactaaa actatacaaa gaactttaaa aagttctagt aaattattaa

85681 aaggacgttg aattattaat tacattaaat aatattatag ttatagtcct gcgagtataa

85741 agttttatgc aatttttgga aaaaaataaa acttaaagca gaatagcctg acagggttat

85801 tgaagaaata tttatgtttc tttggcgata ctagtgaaaa cgattaaaat atattaagaa

85861 attaatatac aagatcgtcg gttctccata gagtcgcgac agactgggtc actagtgggt

85921 ggctgaaatg ctgcttaatg cacagtcgga actatttatc catcgataaa tgtgatattc

85981 gaacaagaaa gatttcacag cttttaaaga cttcacatct ttaaaagtaa gtttgtatgt

86041 tttaccatat ggtcaaatgt cattatgagg tgcaacagtt attactaatc ttattagtgc

86101 tataccttga attggacaag acatagttga gtcgacaaaa tatattacag tattaagtat

86161 tagtttattt attttatgtt cagttttacc tactattggt actgttcata aaaacgcgtt

86221 aaaggtattc aataaaacct tagataaaaa tcattattta tcaattccta gtcctttttt

86281 aagtttttta gtagggttaa tagatggaga tggttatatt cagataggta aaacaactaa

86341 aggttttata acaataaaat tagtaatttc tttacactta gaagatatct ctactttaga

86401 atatattaaa tctactttaa aattaggaaa tattaatgta tataaggatc taaaaagtcc

86461 aacttgtaga ctggtaataa atagaactga attacaagaa attttctttc ccttattaat

86521 acataataac atctttttct taactaaaac tagagtaaat caatttaatt tagctatgca

86581 tattcttaaa aatgatatta aattatttga tgcaatacca tctgaagaaa aaattgaaaa

86641 tgtatttcaa ttacctagta gtccttatga ttatacatta ttacattttt ttaaaaactg

86701 gattgtagga tttacatccg cagaaggttc ttttttcatt aaaagtaata atgatggttg

86761 ctttcaatta aaacaaagaa cccatacaaa tttatttgaa gcttttaagt tagtatttaa

86821 tactaaacga aaaatagatg ctacaaatac ctataatcaa tttggtgtta gttctaaaac

86881 tgatatacaa actgttataa attttttttc ttttgaaggt ttgcatcctt taacaggatt

86941 aaaatatatt caatatttaa attgattaag agatttacaa aaatctgcgc gttataataa

87001 actaaattac ccaaaactgt aaaatgggct ccttagaaat ataatttatt ttctagaggc

87061 aaatggggaa aattacaagc acatttaatt aacaatatac ctccgtgtta ttaaattact

87121 tgtagcggag tttaattcgg cttaatatag aattaaaaac gttcaaagac taatgagtga

87181 gttaagccta caataagctc gacacgataa ccccaaaatt ttataatatt aaataaaatt

87241 taagacatag tctaattata tttgaaaaaa tatattatat atattaaaaa ttatataaaa

87301 atatattgtc atttgaggag gttttagcgt tagtaatgca actttaaata gattctttgc

87361 attacatttt gtattacctt ttgtattagc tgctttagtt ttaatgcatt taatagcact

87421 tcatgataca gcagggtcag gtaatccttt gggagtggct ggtacttatg atagaatacc

87481 atttgctcct tattaccttt ttaaagattt aattactatt tttatattta tttttgtttt

87541 aagtttcttt gttttcttta tgcctaatgt tttaggtgac tcagataatt atataatggc

87601 taatcctatg cagacacctg cagctattgt acctgaatga taaagtaaaa taaatgtcat

87661 tctaaaatct cgcgaattgc tggaaaccca ttttaatttt aatttaaata tgacaatcag

87721 cagggaaacc ttttattaag ctaatatagt atagcagaag gaagttttca actctgaaac

87781 atatttttcg tgatgtaaat aatggatgat taattcgtta ttgacatata ctaagctaat

87841 gaaggaatct tcagagacta tacacgagac aattataata tatataattg aagagatagt

87901 ccgatcatag tagaaatatt atgagtttaa cacaccatca attggtttat cctctatact

87961 attctttatg tgtagtccag tattatttgc tgcacgttca actaaaggaa taactcgttt

88021 atctcctgcg gaaagagctg aaataaaatt accggatgaa gtaaaagaag tattaatagg

88081 gattttactt gatgatggct tgttcgtagt ggttaagagg tgatcctttt tagccgccga

88141 tattttattc ggcgttggat atttatattt tagatattta ttaagctttt tactatgttt

88201 tattaactct aaggttgttt atcttaatcc tcaagattca aaagctataa ttttaaaaga

88261 aaataaaggt aaagcaggta tttatttatg aacaaataat attaatggaa aaagatatgt

88321 tggaagttct atagatttat ctgctagact tagaaattat tttaatcttt cttatctagc

88381 atcttctaaa gatattatgg taatttatag agctttatta gcttatggat ttaaaaattt

88441 tacattagag attttggaat tttgtgatcc ttctatttta ttagaaagag aacaatatta

88501 tatagatact ttaaatcctg aatataatat tttaaaaata gcaggatcca gattaggggt

88561 taaacacact ttagatacaa tagagaaaat tagaactggg gctttaaatc gttcaaaaga

88621 ggctttagat aaaaacttag agcatatcaa aaatttaaat tcgagtctag agcataaaga

88681 gcatttaatt aaacttaata cttcccttga acatatagct aaaactgctc atcctattga

88741 agtttttgat acactaaata aagagagtca gtattttaga tctattactc aagtggcaaa

88801 gttttttgaa gtacaccctg aaaatgtacg tagacatatc ttaaaagctt tttttagata

88861 gatatcttat taaaaaacgt ccacaaatat agtcttcttt aatgatctat atctaacata

88921 gtaagaagat caccaactgc taactctaga ttagtttatt ctcaaactgc tgtaaaacat

88981 aaagaatatt tcgattatgt atttagtttc tttttacctt tttgtgctaa tgactataaa

89041 cctcaatcta gactagtagt ggataataga actaaaaaaa acatacagtg ctatatcttt

89101 caccactatg caactccctt cttttaatga atatagagag ttattttatg atttaaatag

89161 aaaaaaaatc attcccgaaa acataagaga attgttaaca ccccgtggtt tagctttttg

89221 aattatggat gacggaagta gacaaggtag cggtttacat ctaagtgttt atgtattctc

89281 tcacgtcgca cacagatgta gataaattaa tgtttacgcg gcttcgccga ctacaagata

89341 aattcaatct aaaatgctct atacattata acagggataa caaaccccgt atttatatat

89401 ttaaagaatc tatggataat ttaataactt tagtaaaacc ttattttatt aaagaaatgc

89461 tatataaatt agggttataa agctgtcatt ttaaagtttc ccttggatac aaattgacaa

89521 tcgatttatt acctttctat gcaatattaa gatctatacc taataaatta ttaggtgtta

89581 ttgcaatgtt tggggcttta gtaattttac ttactttacc ttatgctgat ttaggtagat

89641 ctagaggttt ccaatttaga cctttaagta aattagcttt ctttttattc gttgtaattt

89701 tcttaacttt aggattttta ggagctaaac atgtagagtc tccatatatt attatgggac

89761 aaatatatgc aatattatat ttttcttatt tcttaataat attacctaca gttagtgtat

89821 tagaaaatag tcttatggat gtagcctatt taaagaatat taataaataa taagtataaa

89881 tttgtattgg taaataaaaa tattattacc attgtaatta tatatcttat ttatttaata

89941 tattcaaaag taaaatcata cgcttagttt taactttatt taaagaagtt aaagattata

90001 tacctaaaat ataatattta ttattttagt ttaagtactt ttaagtaagt actattaaaa

90061 atatatgcat atatatataa atatatgtat ataagattgc gaggtaaatg gtttacactt

90121 tgattgcaga tcttaagtat gaggttcgat tcctccgtaa tcttatagta tacaataaat

90181 tcttattagc ttaagggtaa agcaagatac ttctaatatc aagattctag ttcgaatcta

90241 gaataagaat tttttataat ttttttttcc aaaatttcat actataaagt tatttaattt

90301 tatttactct attaacagtt ttaacaatat tttcaatcta agtagtaaaa gttatgtatt

90361 aaaatcatct atatct

//

LOCUS Ceratocystiopsis_minuta39800 bp DNA linear 17-JAN-2019

DEFINITION .

ACCESSION

VERSION

KEYWORDS .

SOURCE Unknown.

ORGANISM Unknown.

Unclassified.

REFERENCE 1 (bases 1 to 39800)

AUTHORS Wai, Zubaer, Hausner

TITLE Direct Submission

JOURNAL Submitted (17-JAN-2019) Department of Microbiology, University of

Manitoba, 66 Chancellors Circle, Winnipeg R3T 2N2, Canada

FEATURES Location/Qualifiers

source 1..39800

/organism="Ceratocystiopsis minuta"

/mol_type="genomic DNA"

gene 1..9704

/gene="cox1"

CDS join(1..212,1450..1968,3001..3136,4269..4526,6818..6954,7956..7989,9414..9704)

/gene="cox1"

/EC_number="1.9.3.1"

/codon_start=1

/transl_table=4

/product="cytochrome c oxidase subunit 1"

/translation="MSIERWFLSTNAKDIGVLYLMFALFSGLLGTAFSVLIRMELSGP

GVQYISDNQLYNSIITAHAILMIFFMVMPALIGGFGNFLLPLVIGGPDMAFPRLNNIS

FWLLPPSLILLVFSACIEGGAGTGWTIYPPLSGIQSHSGPSVDLAIFALHLSGISSLL

GAMNFITTVINMRTPGIRLHKLSLFGWAVVITAVLLLLSLPVLAGGITMVLTDRNFNT

SFFETAGGGDPILFQHLFWFFGHPEVYILIIPAFGIISTTISANSNKSVFGYIGMVYA

MMSIGILGFIVWSHHMYTVGLDVDTRAYFTAATLIIAVPTGIKIFSWLATCYGGSIKL

TPSMLFALGFVFMFTIGGLSGVLLANAALDTAFHDTYYVVAHFHYVLSMGAVFALFSG

WYFWIPKILGLSYNILLSKVHFWVLFIGVNLTFFPQHFLGLQGMPRRISDYPDAFTGW

NLVSSFGSIVSVIATVLFLYIVYKQLIDNNPVGRFPWLMPQYFTDALQALLNRNYPSL

EWALTSPPKPHAFVSLPLQS*TKGKLF"

exon 1..212

/gene="cox1"

/number=1

intron 213..1449

/gene="cox1"

/note="Group IB"

/number=1

gene 214..1176

/gene="orf320"

CDS 214..1176

/gene="orf320"

/codon_start=1

/transl_except=(pos:214..216,aa:Asn)

/transl_table=4

/product="hypothetical protein"

/translation="NEWRLFNLKFQSNSVLCNNQGNNMPISENNNNSNNNNNNEIPKY

IKVYVENPFNNRDLILKVTKNQKGIYVWESNNHVYVGHSINLYNRISSYFMPSILKTK

ARRVLRYFNKHGFQDTNLTIYIMGENSTLDEVVRLEQHFIDTLNPSLNVDLVASSSGY

HEPMSQEIREKLRKQRGTPIYVYKAEDFTLLYIFESKQHTYDSINIHHKTLNNCLNGD

TIYLDTFFLSLDQIEESDNINLLPLEELKKLINNAREVYVVKHPASSFILAEFKDDSS

KNLLFLSLTSLANHLKGDRKTIRQYLKGEKSGYYRGKWKFSYID"

exon 1450..1968

/gene="cox1"

/number=2

intron 1969..3000

/gene="cox1"

/note="Group IB"

/number=2

gene 1970..2917

/gene="orf315"

CDS 1970..2917

/gene="orf315"

/codon_start=1

/transl_except=(pos:1970..1972,aa:Lys)

/transl_table=4

/product="hypothetical protein"

/translation="KFVSLLMLLYAGNTSLYSFKYSLLIDIVKKLKQWSQSAGNVFNF

KYGTSETIRNNTETIKNISIHVPEHLKPLNDKQFGHYLAGLIDGDGHFSSAQQLVIVF

SSPDVNLAYYIKKTIGFGSVKKVKDKNAYVYIISNKEGLLKAINLINGKLRSINKFNQ

VNTILSNTKYLNENLVFKINDSNDFNNHWIAGFSDADASFQLKIVNRDNRIKPEIRLN

FQIDQKDRNMLLLIKNIFGGNIGYREAQNTYYYGSTSFGSAKKVINYFDTFHLQSSKH

TNYLKWRKAYLIVQNKDHLTETGIEKIKKLKYSMNSYSV"

exon 3001..3136

/gene="cox1"

/number=3

gene 3137..4186

/gene="orf349"

CDS 3137..4186

/gene="orf349"

/codon_start=1

/transl_except=(pos:3137..3139,aa:Trp)

/transl_table=4

/product="hypothetical protein"

/translation="WVTMASPYSDIGITINFAICWNSLVLISTLYGKNLISYTQSADN

LSLYLLKDNKQSVSETTRETSLKFSTFHAYYYTLFKNRNSISDDWLTWFIGFAEGDGA

IQTYDNGKRVRFVLTQKESDILHKIQFKFNIGVVKHFPQGKSGKSNDFHRWMVDNPSH

ILLLAHLFNGNLAQNHRIEQLALWANALNNRFGYDTIKLNNIPATVTLQDAWLSGFTD

AEGCFNVSITTNSRYTLGHVIKMRYLLDQKDSIILNKVYELFGFGKVTLRSGTDNVYR

YTATGFKALNDIIAYFKLFPLQTKKALSFEKWLTIHNKVQAKSHLTEEGLSQIRTLQK

KINLNNSMTNKTGKA"

intron 3137..4268

/gene="cox1"

/note="Group IB"

/number=3

exon 4269..4526

/gene="cox1"

/number=4

gene 4527..5531

/gene="orf334"

CDS 4527..5531

/gene="orf334"

/codon_start=1

/transl_except=(pos:4527..4529,aa:Glu)

/transl_table=4

/product="hypothetical protein"

/translation="EVGLNNLPVDYFATDYMLGTIGRGYYLLIINTFYVFSRKNFFNS

KKNNTSLEDKICHLLPAENCEGFSETIRQSLDRRNFWNRFAGVLDTKGYFDIKIYNNK

KILKQIIIKLNNNDIRILKYIQNYLHIGKIISNKNKSMYIINIENHIIYILKNINGLI

RIKVPEFKEACYLLNIEFKEAKYRINPKNSYFAGLFDTKGNIVFNEITNTIECNLILK

TNKYIKKLDLSHAIFKSKPMRVERRSKVNDKLYTAFLFQKYGNMLYIYKYFLKNKLYS

NIKFYKISQIYSFLKIREYKFYPKYTLEYNIYSTFLKNWFKHGNLCWYKSTFIKNIYI

"

intron 4527..6817

/gene="cox1"

/note="Group IB(3')"

/number=4

gene 5874..6791

/gene="orf305"

CDS 5874..6791

/gene="orf305"

/codon_start=1

/transl_table=4

/product="hypothetical protein"

/translation="MEVIITRTGALKDKLGLKGSQDFENKWDSIINFLYKTIYNYTKS

INPFFLFLSVYFINFFFDDNSSISFYALRLLFQFSFLISLIYLNKIWIDQKTLKIDFP

FFHMLIKFILGCCLIYNVAVIIKTLYVICILIYNYVLKFNFWSKIKDLKLSFDYNKFK

GPNNPKDNNTIFYSENKKRNKKTASELKDLILKAQAKKAKSNLSPDLNLESSNFNRNW

DKKINIEKLPDFSIEDQIRNIEKEYIAYDLQERKFKDIVVNIGKKKEEFFPDESKSLF

KDYVDLVNLLKVNLKNNLKSLKKFQRKNK"

exon 6818..6954

/gene="cox1"

/number=5

intron 6955..7955

/gene="cox1"

/note="Group IB"

/number=5

gene 6956..7750

/gene="orf264"

CDS 6956..7750

/gene="orf264"

/codon_start=1

/transl_except=(pos:6956..6958,aa:Thr)

/transl_table=4

/product="hypothetical protein"

/translation="TLKGSTFVVSGKRGFSNKNSPDPNEFVLFFENVNKEKRNIYKEL

RKKAGVYLFKNKITNDFYIGSSTNLTSRMVSYYYYTNSQNPSRMVIIRAMKKYGLDNF

SLGILEFCEKDVCLILEQKWLDQYKPKYNVLSTAGNSLGYKHSIETINKLKEKLSKEL

HPKFGTVTSPETREAISESIKEFYLNNSHPSKGLKGKLSPQYGIGGQFVFCYNKNGKE

LIFPSINGARQHFKVRWTLIKSNIDKNKWITLNGEDWLIQSSPRQI"

exon 7956..7989

/gene="cox1"

/number=6

intron 7990..9413

/gene="cox1"

/note="Group IB"

/number=6

gene 8416..9399

/gene="orf327"

CDS 8416..9399

/gene="orf327"

/codon_start=1

/transl_table=4

/product="hypothetical protein"

/translation="MKTLKNNKFIVQDWDNDALVEKAIDNNNLITIKPEHKFSQECKL

DLANLSSTSDSPLYNFLYDKEIILKEYKNKSGIYLLHNNVNGKEYVGSGKDLSKRLAT

YYFPSRLTDSRFISNSILKYGHDNFSLVILCVLGDSYSSSKPDIISKEQEYIDLYKPI

LNLNPVAGSSMGFKHSEESKRLISEFHKGKPLSEDTKKKLSILFSGKLNPFWSKTHSS

ETLDKMSKSKTGTLNPMFNKEKSKEFIEQMYRDKSGANNPMFGKPKSEETLAKLRKKV

YVYDYNKQFIKCYDSIGFAVKDLHIAAETIKKYINTDKKYKDKYFYSSLQE"

exon 9414..9704

/gene="cox1"

/number=7

gene 9846..11983

/gene="nad1"

CDS join(9846..10481,11549..11983)

/gene="nad1"

/EC_number="1.6.5.3"

/codon_start=1

/transl_table=4

/product="NADH dehydrogenase subunit 1"

/translation="MLRFPTLVSIIEVVLVLVPALLAVAYVTVAERKTMASMQRRLGP

NAVGYYGLLQAFADALKLILKENIAPNQANLTLFFLGPVITLVFSLLGYGVIPYGPGL

AISDLNLGIFYLLAVSSLSTYGILLAGWSANSKYAFLGSLRSTAQLISYELVLSSAIL

LVVFITGSLNLTVNMEAQRAVWFILPLLPIFIIFFIASVAETNRAPFDLAEAESELVS

GFMTEHAAVVFVFFFLAEYGSIVLMCILTSILFIGGYLIIGSSIIDISLYIYDSNFII

NYIINSPIIEGLLYGISLGIKSSIMIFVFIWTRASFPRIRFDQLMSFCWTVLLPVLFA

IIILIPAILYNFILFPINISLL"

exon 9846..10481

/gene="nad1"

/number=1

intron 10482..11548

/gene="nad1"

/note="Group IB"

/number=1

gene 11154..11525

/gene="orf123"

CDS 11154..11525

/gene="orf123"

/codon_start=1

/transl_table=4

/product="hypothetical protein"

/translation="MLGYKHTDEAKQKMVERFKDITNHPMYGKNHSEEILKLISKPGK

LNPMYGKKHSDETKKAISLKMSKYPNGVGIFDLNNNLINKFSNNTEIADYLGISKVTV

AKYLNNDLIYKGMYKFKPILS"

exon 11549..11983

/gene="nad1"

/number=2

gene 12092..13555

/gene="nad4"

CDS 12092..13555

/gene="nad4"

/EC_number="1.6.5.3"

/codon_start=1

/transl_table=4

/product="NADH dehydrogenase subunit 4"

/translation="MLLSFLLLVPIIGIFLIAGTISYQDNVIKTTYYKNIALITSIIN

LIISLVVYMLFDSNTNQFQFVQEHYNLSYFDIYLGVDGISIYFVLLTTIIMPIAILAN

WNSITDNIKSYLIIMLLLETLLLAIFLVLDILLFYIFFESTLPPLFLLVGLFGSNNKV

RASFYLFLYTLWGSLFLLIGILAISSIMGTTDFDALFKTNFDYITQLVLFSGIFLSFA

VKTPTIFLNSWLLKAHVESPLGGSIVLAAIVLKTSLYGIFRLILPILPKASINFTYIV

YVIGVITIIYASFSTLRTTDIKELIAYSSVSHAAVYLMGVFSNTIQGIEGGILLGLGH

GFVSSGLFICAGGILYDRSGTRSIFFYKGIAQIMPLFAILFLILSLGNCGVPLTFNFV

GEFMSLYGAFERLPLLGVFASSSIILSAAYTMYMFNRIGFGGTFSKYFKENIVDLTKR

EFFILLTLVIFTVILGIYPSIVLDGLHYSVSGLIYNQ"

gene 13800..13958

/gene="atp8"

CDS 13800..13958

/gene="atp8"

/EC_number="3.6.3.14"

/codon_start=1

/transl_table=4

/product="ATP synthase F0 subunit 8"

/translation="MPQLVPFYFLNEVIFTFSIITIIVYLFSKYILPRFVRLFLSRTF

ISKLFDNK"

gene 14029..16288

/gene="atp6"

CDS join(14029..14612,16081..16288)

/gene="atp6"

/EC_number="3.6.3.14"

/codon_start=1

/transl_table=4

/product="ATP synthase F0 subunit a"

/translation="MNTLSINTVNLETLSPLSQFEIKDLLSIDAPLLGNLHISLTNIG

FYLSIGAFIILTLSLLSTNYNKLISNNWSISQETLYATVHSIVTNQINPRNGQMYFPF

IYTLFIFILINNLIGMVPYSFASTSHFVLTFSLSFTIVLGATILGFQKHGLEFFSLLV

PAGCPLALLPLLVLIELISYLARNISLGLRLAANIMSGHMLLHILAGFTYNIMSSGII

FFFLGLLPLAFIIAFSGLEIGIAFIQAQVFVVLTSSYIKDGLDLH"

exon 14029..14612

/gene="atp6"

/number=1

intron 14613..16080

/gene="atp6"

/note="Group IC2"

/number=1

gene 15285..16049

/gene="orf254"

CDS 15285..16049

/gene="orf254"

/codon_start=1

/transl_table=4

/product="hypothetical protein"

/translation="MWTNQLNGKKYVGSSVDLRRRLLEYYNVKRLLNETSMPICTALL

KYGYKNFSLTILEFCDIEKLMLREKHYFEVYSPEYNILKTPGSPDRGSGWKHSEATIE

IMRVAAQKRIESSKYINKLSEAQLNGIEIEVTDLKTNTSTKYHAIRAAARALNIDKRY

IENYVYLNQDKPVLDRYTFKLLSEKETKYIPKVQKTSIKLEVTNIETKEITIYPSIGA

AAKELGFHQASVSLYLKENRIKPFKGLYLFKIVKDN"

exon 16081..16288

/gene="atp6"

/number=2

gene 16565..18064

/gene="rns"

rRNA 16565..18064

/gene="rns"

/product="small subunit ribosomal RNA"

gene 18102..18187

/gene="trnY(gta)"

tRNA 18102..18187

/gene="trnY(gta)"

/product="tRNA-Tyr"

/anticodon=(pos:18137..18139,aa:Tyr,seq:gta)

gene 18195..18265

/gene="trnN(gtt)"

tRNA 18195..18265

/gene="trnN(gtt)"

/product="tRNA-Asn"

/anticodon=(pos:18227..18229,aa:Asn,seq:gtt)

gene 18343..19152

/gene="cox3"

CDS 18343..19152

/gene="cox3"

/EC_number="1.9.3.1"

/codon_start=1

/transl_table=4

/product="cytochrome c oxidase subunit 3"

/translation="MDIKRRSIFQDHPFHLVSPSPWPFFTSFSLLGLTSNMALYMHNF

DYASYLSFVSLFCLIMSMFFWFKDVTTEGTYLGDHTLAVQQGLNVGIMLFIVSEAFFF

LAIFWAFFHSALSPTVELGAQWPPFGIQPVNPFELPLLNTVLLLSSGATITYAHHSLL

SGDRPDAVYWSIVSIVLAIIFTVFQGVEYAVSSFTISDSVYGACFFFGTGFHGIHVMI

GSLFLLIALMLIVTYHATDSHHQGYESAILYWHFVDVVWLFLYIAMYYWGS"

gene 18951..20753

/gene="orf600"

CDS 18951..20753

/gene="orf600"

/codon_start=1

/transl_table=4

/product="hypothetical protein"

/translation="MFFFRNWVSWDTCDDWFAFFIDSFDVDCYLSCDWQSSSGLWICD

IVLTLCRCSVTFPLYSNVLLRFINPLWTKISLYSNVLLKFINTLWTKLISLCKNVLLR

FINPLWTKLISLCRNILLRFINPLWTKLISLWRNVLLRFINPLWTKLIPLRSKLDRSE

VLASILSTSLVLICVLNPDLTEVVFHTTIFCTYAYVAYNTGIISKIYTIFKTHKIKFC

LLVIFIFIGIIFIFIGIILWNIIDSLVFITGGQGSNFNNNFPNFNNNNNNGPNPPQNN

DMVVASGSHNDEHNRRNTRIWNNNPPFINRPATPLFNAPSDASASLMVNAPNSVSATP

AVNTTTVANATPVVNAVVNATPVVNAPSDASASSAVSNRPLIVYSSDTVPGPDSVILR

HSDFAPVYRSHDEAMAYVASRTSRFIFEDNRDYLTGIPEGRPNFWFNEQGRLMSSLST

NIYVHGPYEDLHYVRHIPLWHVRYNLHDIPFLTQGPPVYYVDDMSSYVNTIPTSGPHH

LPNTYTNYPFYMKHYNNPMSRLEIEAAIDNLIKLHFNNSIQIARSENDILSPGTPHTG

KNILIEKVVNLRDRNMYISIDHYLLLKKWHWNTF"

gene 20810..20881

/gene="trnK(ttt)"

tRNA 20810..20881

/gene="trnK(ttt)"

/product="tRNA-Lys"

/anticodon=(pos:20842..20844,aa:Lys,seq:ttt)

gene 20893..20965

/gene="trnD(gtc)"

tRNA 20893..20965

/gene="trnD(gtc)"

/product="tRNA-Asp"

/anticodon=(pos:20926..20928,aa:Asp,seq:gtc)

gene 21138..21217

/gene="trnS(gct)"

tRNA 21138..21217

/gene="trnS(gct)"

/product="tRNA-Ser"

/anticodon=(pos:21170..21172,aa:Ser,seq:gct)

gene 21221..21291

/gene="trnW(tca)"

tRNA 21221..21291

/gene="trnW(tca)"

/product="tRNA-Trp"

/anticodon=(pos:21253..21255,aa:Trp,seq:tca)

gene 21347..22006

/gene="nad6"

CDS 21347..22006

/gene="nad6"

/EC_number="1.6.5.3"

/codon_start=1

/transl_table=4

/product="NADH dehydrogenase subunit 6"

/translation="MNNLFIINENFTNGYRSEVLDILCLLIILSGIFVIVSKNPIISL

LFLIGLFAGVSCYLLIIGLNFLGLSYLVVYIGAVSILFLFILMLINIRMSELQSNTSN

SIPLAISIAILFNYPLFQLLPYNVAILNNNNNIINNILYNVSFNKIDNGLKTNLDINN

NDTLFVTSKIWDANLAEVGHISSIGNIFYTNYNIWLIIVGFILLLAMVGSITIVMKQK

N"

gene 22023..22094

/gene="trnV(tac)"

tRNA 22023..22094

/gene="trnV(tac)"

/product="tRNA-Val"

/anticodon=(pos:22055..22057,aa:Val,seq:tac)

gene 22095..22165

/gene="trnI(gat)"

tRNA 22095..22165

/gene="trnI(gat)"

/product="tRNA-Ile"

/anticodon=(pos:22127..22129,aa:Ile,seq:gat)

gene 22171..22255

/gene="trnS(tga)"

tRNA 22171..22255

/gene="trnS(tga)"

/product="tRNA-Ser"

/anticodon=(pos:22205..22207,aa:Ser,seq:tga)

gene 22263..22334

/gene="trnP(tgg)"

tRNA 22263..22334

/gene="trnP(tgg)"

/product="tRNA-Pro"

/anticodon=(pos:22296..22298,aa:Pro,seq:tgg)

gene 24949..26067

/gene="rps3"

CDS 24949..26067

/gene="rps3"

/codon_start=1

/transl_table=4

/product="ribosomal protein S3"

/translation="MNIKNNFIGENNYFPSDYKEWNNNIYYFNNNNIKNIPAYDLIIN

KLLKGYFNSYLNPNINFLYSNKRYTSLNKIYISKANIKHTNSKAIITIYVYDREKISI

SKFFIKYSYIILAAIKELKGFVFENNKFFQNRLNRFQGYNNLLKLLNFFKSRKLKAWL

NELRYKDILIYKLSKLISKFYKKKIEFNIIRLTSIAYNSDILVDVMRKSYKKTGHVRN

SMKFILNQGLIEKIDNTNDRVRATKNVDFNLLANKYQNLNINSIVKNVNINETIKNLY

KTNNKNNEEIIFNSIKYKILGGIRIDIKGRLTRRFRADRSVSNTTLKGSFRNIDSSYK

GLTILGYRGCASSSIDYSMSISKRHVGAFAIKGWVSGR"

gene 26713..26783

/gene="trnT(tgt)"

tRNA 26713..26783

/gene="trnT(tgt)"

/product="tRNA-Thr"

/anticodon=(pos:26745..26747,aa:Thr,seq:tgt)

gene 26786..26858

/gene="trnE(ttc)"

tRNA 26786..26858

/gene="trnE(ttc)"

/product="tRNA-Glu"

/anticodon=(pos:26819..26821,aa:Glu,seq:ttc)

gene 26860..26932

/gene="trnM(cat)"

/note="copy 1"

tRNA 26860..26932

/gene="trnM(cat)"

/product="tRNA-Met"

/anticodon=(pos:26893..26895,aa:Met,seq:cat)

gene 26936..27006

/gene="trnM(cat)"

/note="copy 2"

tRNA 26936..27006

/gene="trnM(cat)"

/product="tRNA-Met"

/anticodon=(pos:26968..26970,aa:Met,seq:cat)

gene 27010..27092

/gene="trnL(taa)"

tRNA 27010..27092

/gene="trnL(taa)"

/product="tRNA-Leu"

/anticodon=(pos:27044..27046,aa:Leu,seq:taa)

gene 27100..27170

/gene="trnG(tcc)"

tRNA 27100..27170

/gene="trnG(tcc)"

/product="tRNA-Gly"

/anticodon=(pos:27132..27134,aa:Gly,seq:tcc)

gene 27175..27246

/gene="trnA(tgc)"

tRNA 27175..27246

/gene="trnA(tgc)"

/product="tRNA-Ala"

/anticodon=(pos:27208..27210,aa:Ala,seq:tgc)

gene 27253..27325

/gene="trnF(gaa)"

tRNA 27253..27325

/gene="trnF(gaa)"

/product="tRNA-Phe"

/anticodon=(pos:27286..27288,aa:Phe,seq:gaa)

gene 27351..27432

/gene="trnL(tag)"

tRNA 27351..27432

/gene="trnL(tag)"

/product="tRNA-Leu"

/anticodon=(pos:27385..27387,aa:Leu,seq:tag)

gene 27475..27547

/gene="trnQ(ttg)"

tRNA 27475..27547

/gene="trnQ(ttg)"

/product="tRNA-Gln"

/anticodon=(pos:27508..27510,aa:Gln,seq:ttg)

gene 27592..27665

/gene="trnH(gtg)"

tRNA 27592..27665

/gene="trnH(gtg)"

/product="tRNA-His"

/anticodon=(pos:27626..27628,aa:His,seq:gtg)

gene 27692..27764

/gene="trnM(cat)"

/note="copy 3"

tRNA 27692..27764

/gene="trnM(cat)"

/product="tRNA-Met"

/anticodon=(pos:27726..27728,aa:Met,seq:cat)

gene 27821..27892

/gene="trnM(cat)"

/note="copy 4"

tRNA 27821..27892

/gene="trnM(cat)"

/product="tRNA-Met"

/anticodon=(pos:27854..27856,aa:Met,seq:cat)

gene 27936..29597

/gene="nad2"

CDS 27936..29597

/gene="nad2"

/EC_number="1.6.5.3"

/codon_start=1

/transl_table=4

/product="NADH dehydrogenase subunit 2"

/translation="MLLISILSLLLSNAVTIRRDISILFNRVAIIALIYSILHSVTTL

FIIGKGIGLHGGLLNVTSITQVFNIFIFLVSILILQLTSFFPRKVWIPEHSSLIQLLF

NNLVFYRTKVINKMGEHLRIIEYPLILLFIISGAVFLISTSDLISVFLAIELQSYGLY

LLSTIYRNSELSTTGGLMYFLLGGLSSCFILLGTSLLYANSGTTNLDAIYAITSLSDS

ADIWYKPYYINFAFLIFSIGFLFKVSAAPFHFWSPDVYDAIPTIVTTFVAIIAKISIF

IFLLEIVYYTKNYFTDFNWTYGLLISSFLSMIIGTVVGLTQFRIKRLFAYSTISHVGF

ILLALSISSVESTQAFIFYLMQYSISNLNAFLILVAIGFSLYCYINNNKEYKDLLDKN

NSPIQLISQLKGYFYINPTLALSLTVTLFSFAGIPPLAGFFAKQMVLSAAIDNGYIFI

TLVAISTSVIGAVYYINIIKEIFFYSPEYKINPLLENLNFNGNIYNNKNVLIKSINFK

YNNIVISSSISITISIITLIILLFIFVNKEWLSMSTILVQYLFNY"

gene 29598..30011

/gene="nad3"

CDS 29598..30011

/gene="nad3"

/EC_number="1.6.5.3"

/codon_start=1

/transl_table=4

/product="NADH dehydrogenase subunit 3"

/translation="MSSMSIFFIFVIVVAILFLALNLIFAPSNPYQEKYSAFECGFHS

FSQSRSQFNITFFIYALVFLLLDLEILLLYPYAVSSYSNDIYGLIIVLIFTTIVTIGF

VFELGKGALKIGSRQDVESISKKNNIIISLIGTKK"

gene complement(30076..30414)

/gene="orf112"

CDS complement(30076..30414)

/gene="orf112"

/codon_start=1

/transl_table=4

/product="hypothetical protein"

/translation="MNKTKMLKKLFIPRLQISAFFRHFHAILNPSVASSSNYLFNEEL

LMGINSILSDNNLTKLEQQIKLEEFLLNQYKELFDTNKVHLPANINTNVLNPDFVNYY

FFSEMIFISI"

gene 30611..30802

/gene="atp9"

CDS 30611..30802

/gene="atp9"

/EC_number="3.6.3.14"

/codon_start=1

/transl_table=4

/product="ATP synthase F0 subunit c"

/translation="MIQVAKIIGTGLATTGLIGAGVGIGVVFGALILGVARNPSMRGQ

LFSYAILGFAFSEATGLSE"

gene 31084..31899

/gene="orf271"

CDS 31084..31899

/gene="orf271"

/codon_start=1

/transl_table=4

/product="hypothetical protein"

/translation="MKINYKIYIKNNFTLARQFSNHTNDIYSYSIKTYKDPLNQRKLI

RDENNGKTGVYCWINNINGKYYIGSGDPLYLRISDYFQDWYILSRTNLYIVRAISKYG

IASFSLIILEYSNSDNVIKSEQKWIDLLKPDYNLSPTAGNTKGYRHRIESIEKMRDKA

LGRKHTEEVKQSMSESRKGENNPFYGKTHSQDTLVLLKTAAANRTKPPVPGIEVDIID

IETKLTHTFESIRKAASFMGSDIKTVLRREELQNTKGINTPYKKKYIIIIKRS"

gene 32046..32798

/gene="cox2"

CDS 32046..32798

/gene="cox2"

/EC_number="1.9.3.1"

/codon_start=1

/transl_table=4

/product="cytochrome c oxidase subunit 2"

/translation="MYYFFINLMIKLDAPSPWGIYFQDSATPQMEGLVELHDNIMYYL

VIILFGVGWILLSITKNFIISASPISHKYLNHGTLIELIWTITPAVILILIAFPSFKL

LYLMDEVSDPSMSILAEGHQWYWSYQYPDFLDSSEEFIEFDSYIVPESDLEEGGLRML

EVDTRVIVPELTHIRFIITSGDVIHSFACPSLGIKTDAYPGRLNQVSVFINREGVFYG

QCSEICGILHSSMPIVIESVSLEKFLTWLEEQ"

gene 32829..32901

/gene="trnR(acg)"

tRNA 32829..32901

/gene="trnR(acg)"

/product="tRNA-Arg"

/anticodon=(pos:32862..32864,aa:Arg,seq:acg)

gene 32980..33318

/gene="nad4L"

CDS 32980..33318

/gene="nad4L"

/EC_number="1.6.5.3"

/codon_start=1

/transl_table=4

/product="NADH dehydrogenase subunit 4L"

/translation="MNITLILFLIGILGFVLNRKNIILMLISIEIMLLSITFLILISS

LNIDDIIGQTYAIYIIVVAGAESAIGLGILVAFYRLNRSLFSYYLFYNYTPMYLYKKI

PQQIILGSKY"

gene 33882..36851

/gene="nad5"

CDS join(33882..34598,35607..36851)

/gene="nad5"

/EC_number="1.6.5.3"

/codon_start=1

/transl_table=4

/product="NADH dehydrogenase subunit 5"

/translation="MYLSIIILPLLGSIVSGFFGRKVGVSGAQIITCSSITITTILAI

IAFFEVGINNIVLSINLFRWIDSEWFNIIWGFQFDSLTVSMLIPVLIISTLVHFYSIG

YMSGDPHNQRFFSYLSLFTFMMIILVTANNYLLMFVGWEGVGVCSYLLVSFWFTRIAA

NQSSMSAFITNRVGDCFLVIGMFAILWSLGNLDYSTVFSLAPYVNENIIIIIGVCLLI

GAMAKSSQVGLHVWLPMAMEGPTPVSALIHAATMVTAGVYLLMRSSPLIEYSSTILLL

CLWLGAITTVFSSLVGFFQQDIKKVIAYSTMSQLGMMVIAVGLSSYNVALFHLVNHAF

YKGLLFLGAGAVIHAMADNQDFRKYGGLISFLPLSYSVILIASLSLVAFPFMTGFYSK

DFILESAYGQYYFSSITVYIIAVIGAIFTTLYSVKVLYLTFLTNPNGPLINYKHAHES

DIFMSLPLVILAIFSIFFGFLTKDIFIGLGSGFFIDNSIFIHPNSEIMIDTEFGVSTY

WKLLPFVFTVSFSTIAIILSEFLSENIVNFKLSTIGKTIFGFFNQRFLVEFFYNKYII

NLIFKLGGQTVKVLDKGSIELLGPYGLEKKLINSSKNISSLNKGIVTNYALFILVGFI

LYMFSITLGFTNNLFLIILILIFIISSIY"

exon 33882..34598

/gene="nad5"

/number=1

gene 34599..35501

/gene="orf300"

CDS 34599..35501

/gene="orf300"

/codon_start=1

/transl_except=(pos:34599..34601,aa:Leu)

/transl_table=4

/product="hypothetical protein"

/translation="LVTRALLKLHYMREHPVLTLWSTVYSISFGKIYNEGQSAGNQNK

GSSETTCEADTLDNKFKWWFIGFTEGDGSFIINNTGYLEFKITQSSLDAQVLFYIKKT

LGFGSVSVQSKINNTHQFRVRDKKTILNLINIFNGNIITKYKNQQFNLWLEAFNKIYN

TNIEYIAPKYKVNLDNAWLSGFTDAEGCFTSSAYLAKTGKHIVTVRYVISQKNDIEFS

NDLANLINGYVTHIKSYNGYNTVVNLGKLNIILKYFITYQLKTKKLISYKRWLKVYNL

VKNKEHLTEEGIEKIKSLIKLINK"

intron 34599..35606

/gene="nad5"

/note="Group IB"

/number=1

exon 35607..36851

/gene="nad5"

/number=2

gene 36952..39302

/gene="cob"

CDS join(36952..37344,38526..39302)

/gene="cob"

/EC_number="1.10.2.2"

/codon_start=1

/transl_table=4

/product="apocytochrome b"

/translation="MRVLKNNSILKLANSYLIDASQPSNISYLWNFGSLLAVCLGIQI

ITGVTLAMHYSPSILEAFNSVEHIMRDVNNGWLVRYLHSNTASAFFFLVYLHIGRGIY

YGSYRSPRTLVWTIGVVILILMIGIGFLGYVLPYGQMSLWGATVITNLISAIPWIGQD

IVEFIWGGFSVSNATLNRFFALHFVLPFVLAALVLMHLIALHDTAGSGNPLGVAGTYD

RIPFAPYYLFKDLITIFIFIFVLSFFVFFMPNVLGDSDNYIMANPMQTPAAIVPEWYL

LPFYAILRSIPNKLLGVITMFGALVILLTLPYVDLGRSRGFQFRPLSKLAFFLFVVVF

LTLGFLGAKHVESPYIIMGQIYAILYFSYFLVILPTFSVLENSLMDVAYLKNNNK"

exon 36952..37344

/gene="cob"

/number=1

gene 37345..38202

/gene="orf285"

CDS 37345..38202

/gene="orf285"

/codon_start=1

/transl_except=(pos:37345..37347,aa:Tyr)

/transl_table=4

/product="hypothetical protein"

/translation="YFTIAQNDYKNLYNSKTIKTFNNKRYYSTSHNSVSNFLASKNIK

PVFVYDNLSEDSVRRNIAKETKDLSGIYMILNKESLNYYIGSASTGRFNSRFTNHLIY

LNGSKVLKNSVKKYGLDNFVFIILELFPEIVNQENNKKLLDLEDFYLKSLLPDYNILT

EAGSSFGYKHSEVTRIKMKANYSEERRKEIGELNRGKTFSNETIEAMRQSALNRKKLN

YTEQGILNMKKSSKPILVKELNNTVYGEFNSIVETAEALNCSTKTIQRTLKSSSKLLK

GRWIVNYIK"

intron 37345..38525

/gene="cob"

/note="Group ID"

/number=1

exon 38526..39302

/gene="cob"

/number=2

gene 39517..39587

/gene="trnC(gca)"

tRNA 39517..39587

/gene="trnC(gca)"

/product="tRNA-Cys"

/anticodon=(pos:39549..39551,aa:Cys,seq:gca)

gene 39603..39673

/gene="trnR(tct)"

tRNA 39603..39673

/gene="trnR(tct)"

/product="tRNA-Arg"

/anticodon=(pos:39635..39637,aa:Arg,seq:tct)

BASE COUNT 14725 a 4713 c 5903 g 14459 t

ORIGIN

1 atgtcaatag aaagatgatt tttatctact aatgctaaag atatcggtgt tctttaccta

61 atgtttgcat tattctcagg attattaggt acagctttct ctgtactaat aagaatggaa

121 cttagtggtc caggtgttca gtatatatcg gataatcaat tatacaatag tataattact

181 gctcatgcta tattaatgat tttcttcatg gtcaacgaat gaagactatt taatcttaaa

241 tttcaatcta attctgtatt gtgcaataac caaggcaata atatgcctat ttcagaaaat

301 aacaataact ctaataataa taacaataat gaaataccta agtatattaa agtttacgta

361 gaaaaccctt ttaataatag ggaccttatt ttaaaagtaa cgaaaaatca aaaaggtatt

421 tatgtttgag aaagtaataa ccatgtttat gtagggcatt ctattaattt atataataga

481 ataagttctt attttatgcc ttctattctt aaaactaaag cacgtagagt tttacgttat

541 tttaataaac acgggttcca agatacaaat ctaactattt atataatggg tgaaaattct

601 accttggatg aagttgtaag attggaacaa cattttattg atacattaaa tccaagttta

661 aatgtagatt tagtggcaag tagttcaggt tatcatgaac ctatgagtca agaaataaga

721 gaaaaactac gtaaacaaag aggtactcct atatatgttt acaaagcaga agattttact

781 ttattatata tatttgaatc aaaacaacat acatatgatt caattaatat tcatcataaa

841 actttaaata attgtttaaa tggagataca atatatttag atactttctt tttatcttta

901 gatcaaatag aagaatctga taatataaat ttattacctt tagaagaact taaaaaatta

961 ataaataatg cccgtgaggt atatgtagtt aaacatccag cttcttcctt tatattagca

1021 gaatttaaag atgattctag taagaattta ttatttttat ctttaactag cttagctaat

1081 catttaaaag gagatcgtaa gacaattaga caatatttaa agggagaaaa atctggttat

1141 tacagaggaa aatgaaaatt ttcatatata gattaaatag aagggcatgg ccgggtaaag

1201 tagaaatatt ttactttctt ttcactattt gctggaatcc ccttagagcc tttaaaacta

1261 gtaccgcaga ctaaaagtta gcagtggaat acagtgacat cttaaaggat tgggcaatca

1321 gcaggaaacc aaagataatt ttattgtcaa gtaggatcct cagagactac acgtgaaata

1381 tcttaataaa tactatattt aaagataaag atatagtccg ttcttatgtg aaagcatagg

1441 agtaaaacgt atgcctgctt taataggtgg gtttggtaat ttcttattac ctttagtaat

1501 aggagggcct gatatggcat tccctagatt aaataatata agtttttgat tactacctcc

1561 tagtttaata ttactagtat tttcagcatg tatagaaggt ggagcaggta caggttgaac

1621 tatttatcct ccattatcag gaatacaaag tcatagtgga ccaagtgttg atttagctat

1681 ctttgcactt catttatcag ggataagtag tttattaggt gctatgaatt ttataacaac

1741 tgtaattaat atgagaacac caggtataag attacataaa ttatctttat ttggatgagc

1801 tgttgttatt acagctgttt tattattatt atcactaccc gtattagctg gtggaattac

1861 tatggtttta acagatagaa atttcaatac atctttcttt gaaacagcag gaggtggaga

1921 tcctatttta ttccaacatc ttttctgatt ctttggacac ccagaggtta agtttgtaag

1981 cctcttaatg ttgctgtatg ctggaaacac ttcgctatat agttttaaat actccctctt

2041 aattgatata gtaaaaaagt taaaacaatg aagtcaatca gcaggtaacg tttttaattt

2101 taaatatgga acctcagaga ctatacgcaa caatactgaa actataaaaa atatttctat

2161 tcatgttcct gaacatttaa agccgttaaa tgataaacaa tttggacact acttagcagg

2221 gttgattgat ggagatggac attttagttc tgcacaacaa ctagtaatag tttttagttc

2281 tcctgatgtt aatttagcct actatataaa aaaaacaata gggtttggta gtgtaaaaaa

2341 agttaaagat aaaaatgctt atgtatatat tatatctaat aaagaaggtc tattgaaagc

2401 tataaatttg attaatggta aattaagaag tattaataag tttaatcaag taaatactat

2461 attatctaat actaaatatt taaatgaaaa tttagtattt aaaattaatg attccaatga

2521 ttttaataat cactgaatag ccggattttc tgatgcagat gctagttttc aattaaaaat

2581 agttaataga gataatagga ttaaaccaga aattagatta aactttcaaa ttgaccaaaa

2641 agatagaaat atgctattat taataaaaaa tatatttgga ggtaacatag gttatagaga

2701 agctcaaaat acttattatt atggatcaac aagctttggt tccgctaaaa aagtaataaa

2761 ttattttgat acatttcatc tccaatctag taaacataca aattatttga aatgaagaaa

2821 agcatattta attgtacaaa ataaagatca tttaacagaa acaggaatag aaaaaataaa

2881 aaaattaaaa tattcaatga atagttattc agtataagat agagtcctaa caatgacgga

2941 agttattgag tattaatttt aatagatcat attagactat tatattaatc aaagtatttg

3001 ttacatatta attatccctg cttttggaat aattagtaca actatttctg ctaattctaa

3061 taaatctgtt tttggttaca taggtatggt ttatgctatg atgtctatag gtatattagg

3121 attcatagtt tgaagttgag taacgatggc ttcaccctat agtgatatag ggattacaat

3181 taatttcgct atatgctgga acagtttagt gctaattagt accttgtacg gtaaaaatct

3241 aattagttat actcaatcag cagacaatct gtccctgtat ttattaaagg ataacaaaca

3301 gagcgtctca gagactacac gcgaaacatc tttaaagttt tctacatttc atgcttatta

3361 ttatacatta tttaaaaata gaaactctat ttctgatgat tgattaactt gattcattgg

3421 gtttgcagaa ggagacgggg ctattcaaac ctatgataac ggtaagagag ttcgttttgt

3481 tcttactcaa aaagaaagtg atatacttca taaaatacaa tttaaattta atattggtgt

3541 tgttaaacat tttcctcaag gaaagagcgg taaaagtaat gattttcata gatgaatggt

3601 tgataaccct tcgcatattt tacttttagc tcatttattt aatgggaatt tagctcaaaa

3661 ccacagaatt gaacaattag ctttatgagc taatgcttta aataatcgtt ttggttatga

3721 tactataaag ttaaataata tccctgctac ggttacactg caggatgcct gattatctgg

3781 gtttacagat gctgaagggt gttttaatgt atctattaca acaaattcta gatatacatt

3841 agggcatgtt ataaaaatgc gttacttatt agatcaaaaa gatagtatta tattaaataa

3901 agtatatgaa ttatttggat ttggtaaagt gacattaaga tctggtactg ataatgttta

3961 tcgttatact gctactgggt ttaaagcatt gaacgatata atagcatact ttaaattatt

4021 tccattacaa actaaaaaag ctctttcttt tgaaaaatgg ttaactattc ataataaagt

4081 acaagctaaa tcacatttaa ctgaagaagg attatcacag ataagaactc tgcaaaaaaa

4141 aattaattta aataatagca tgacaaataa aacaggaaag gcttaaagat gaagatatag

4201 tccgatactt cttgtgaaag aagcatactt aattgtatgg gtaaatataa aacatttatt

4261 aacatttgca tcacatgtat acagtaggtt tagatgtaga tacaagagct tatttcacag

4321 ccgctacatt gataattgca gtcccaacag gtataaaaat cttctcatga ttggctactt

4381 gttatggagg ttctattaaa ttaactccat ctatgttatt tgcattaggg tttgtattta

4441 tgtttactat tggagggtta agtggagttt tattagctaa cgctgcattg gacacggcct

4501 tccacgatac atactacgta gttgctgaag tgggcctcaa taatctacct gttgattatt

4561 ttgcaactga ctatatgctt ggaaccattg gaagagggta ttatttacta attattaata

4621 ctttttatgt atttagtaga aaaaattttt ttaatagtaa aaaaaataat acgagcctag

4681 aggataaaat ctgccatttg ctaccagcag aaaactgtga gggattctca gagactatac

4741 gtcagtcgct agatcgaaga aatttttgaa atagatttgc aggtgtctta gacactaaag

4801 gttattttga tattaaaatt tataataata aaaaaattct taaacaaatt ataattaaat

4861 taaataataa tgatattaga atattaaaat atatacaaaa ttatttacat ataggtaaaa

4921 ttataagtaa taaaaataaa tctatgtata taattaatat agaaaatcat ataatatata

4981 ttttaaagaa tattaatggt ttaattagaa ttaaagtccc tgaatttaaa gaagcctgtt

5041 atttattaaa tattgagttt aaagaagcta aatacagaat aaatccgaag aattcttatt

5101 ttgcaggatt atttgataca aaaggaaata tagtgtttaa tgaaataaca aacactattg

5161 aatgtaattt aatattaaaa accaataagt atataaaaaa attggattta agccatgcta

5221 tttttaagtc taagcctatg agagttgagc gtaggtcgaa ggttaatgat aaattatata

5281 cggcatttct atttcaaaaa tatggtaata tgctctatat atataaatat tttttaaaaa

5341 ataaactata ttcaaatata aagttttata aaataagtca aatttattct tttttaaaaa

5401 ttagagaata taaattttat cctaaatata ccttagaata taatatttat tccacttttt

5461 taaaaaattg atttaaacat ggtaaccttt gttgatataa aagtactttt attaaaaata

5521 tatatatata aaaacatcta cctaacatat ctagtaaaga tatagtccgt gtaatataaa

5581 aaaaaaatat agtatgtaat aattatacta cagttacata ctctacaaaa tttaatccag

5641 ttacataatg gtattaattt aatgtaattg gagattggtc ttagtttaat atagttatta

5701 atattaaagc ttatagacta tataccgtga agtcctttaa atttaaattt aaatgtattt

5761 tttatatttt tttttgatat ataaatttgt ttaggcttat attgcgatcg gttcgccgaa

5821 tgttcaaccc aatataaaaa gaagtattag agcacttcct taacgtagta gccatggaag

5881 tgataataac ccgaacaggt gctctaaaag ataaattagg cttaaaggga agtcaagatt

5941 ttgaaaacaa atgagattct atcataaatt ttttgtataa aacaatttac aattatacaa

6001 aatcaataaa tccttttttc ttatttttat ctgtttattt tattaatttt ttctttgatg

6061 ataatagtag tatatctttc tatgctttaa gattattgtt ccaatttagt tttttgatta

6121 gtttgattta tttaaataaa atttgaattg accaaaaaac tttaaaaatt gattttcctt

6181 tttttcatat gttaattaaa tttatattag gttgttgtct tatatataat gtagctgtta

6241 taataaaaac tttgtatgta atatgtatat taatatataa ttatgtatta aaattcaact

6301 tttgaagtaa aataaaagat ttaaaattat cttttgacta taacaagttt aaagggccta

6361 ataaccccaa agataataat actatctttt attcagagaa caaaaagagg aataaaaaaa

6421 cagcttcaga gttaaaagat cttatattga aagcccaagc taagaaagcg aaatctaatt

6481 taagtccaga cttaaattta gaaagttcaa atttcaatag aaattgggat aaaaaaatca

6541 atatagaaaa attacctgat ttttctattg aggatcaaat aaggaacatc gaaaaagagt

6601 atattgcata tgatttacaa gaaaggaagt tcaaggatat tgtggttaac ataggaaaaa

6661 aaaaggaaga gtttttcccc gacgagtcta agagtttatt taaagattat gtggatctag

6721 taaacttact taaagttaac ctaaaaaata atcttaaatc attgaaaaaa tttcaaagaa

6781 agaataaata aagtaaacac caccttaagg tttaatgcat ttccattatg tattaagtat

6841 gggagcagtc ttcgctttat ttagcgggtg atatttttga atacctaaaa tattaggttt

6901 aagttataat attttactat caaaagttca cttctgggta ttatttatag gggttacact

6961 aaagggaagt acatttgtcg taagtggtaa aagaggattt tctaataaaa attcgccgga

7021 tcctaatgag tttgttttat tttttgaaaa tgttaataaa gaaaaaagaa atatatataa

7081 agagttaaga aaaaaagcag gtgtttattt gtttaaaaat aaaataacta atgatttcta

7141 tataggtagt agcactaatt taacaagtag aatggtgagt tattattatt atactaattc

7201 acaaaatccc tctagaatgg ttataattag agctatgaaa aaatatggct tagataattt

7261 ttctttaggt attttagagt tttgtgaaaa agatgtttgt ttaatattag aacaaaaatg

7321 attagatcaa tataaaccta aatataatgt tttaagtaca gctggtaatt ctttaggtta

7381 caagcatagt attgaaacaa ttaataaatt aaaggaaaag ctaagcaaag agcttcaccc

7441 taaatttggt accgttacat ccccagaaac aagagaagct attagtgaaa gcataaaaga

7501 attttatctc aataatagcc atcctagtaa aggattaaaa ggtaaattat ctcctcaata

7561 tggtatagga ggtcaattcg tattttgtta taataaaaat ggtaaagaat tgatttttcc

7621 ttctataaac ggagcaagac aacatttcaa agttagatga actcttatta aaagtaatat

7681 tgataaaaat aaatgaataa ctcttaacgg cgaagattga ttaatacaat cctcacctag

7741 acaaatttag tttgattagc ccctcccaat ccttctatat gctggaaact ctcataaggc

7801 ttaagtactt attttaataa gtgaaaatct taagtatatc tagataatca gcaggaaacc

7861 aacaggtatt aataccttag taggatcctc agagactaca cgaaggagat aattttagta

7921 aattaaaatt atcaagatat agtccacaca atgtgtaatt taacattctt ccctcaacat

7981 ttccttggtc aagtaggccc tttatcataa aaatatgata tttgcacaac actatatgct

8041 agaaactcta ttatattaac tacaataaat agacaattag caggaaatct taataatatg

8101 gaaattaaat ttgtattatt cgtagaaccc tcagagacta tacgtgttgc aactgaaaag

8161 ttgaagaaat agtccaaata atatagtaat atattataat atatcttatt gcacatacct

8221 ttatattatt gtaacaaatt taaacacatt tagcttattt tatgcatatt agtagttaat

8281 tgtctagacc tttaagtaat aagtttgatt gaaaaattta aacttattag taaattattt

8341 aatacacgat atgtaataat accttacaat aacggttctt atagaaaatt aaaatttata

8401 tgtaaaaaaa aataaatgaa aacattaaaa aataataaat ttatagtaca agattgagat

8461 aacgatgcct tagtagaaaa agccattgat aacaataact taattactat aaaacctgaa

8521 cataaattta gtcaagaatg taaattagat ttagctaatt taagttctac ttctgattct

8581 cctttataca attttttata tgataaagaa attattttaa aagaatataa aaataaaagc

8641 ggtatttatc tattacacaa taatgttaat ggaaaagaat atgtaggtag tggtaaagat

8701 ttaagtaaaa gactagctac ttactacttt ccttctcgtt tgacggatag tcgttttatt

8761 tctaattcta ttttaaagta tggacatgat aatttttctc tagttattct atgtgtttta

8821 ggtgatagct attcatcttc aaaaccggat attataagta aagaacaaga atatattgat

8881 ttatataaac ctattcttaa tttaaatcct gtggcggggt ctagcatggg atttaagcac

8941 tcagaagaat ctaaaagact tatatctgaa ttccataaag gaaaaccttt atcagaagat

9001 actaaaaaaa agcttagtat tttattttca gggaaattaa accctttttg atctaaaact

9061 cactcatctg aaacactaga taaaatgagt aaatctaaga cgggtacgtt aaaccctatg

9121 tttaataaag aaaagtctaa agaatttatt gaacaaatgt atagagataa aagtggtgct

9181 aataatccta tgtttggtaa acctaaatct gaagaaactt tagctaaatt aagaaaaaaa

9241 gtttatgttt atgattacaa caaacaattt attaaatgtt acgatagtat agggtttgca

9301 gtaaaagact tacacattgc agcagaaact attaaaaagt atataaatac tgataaaaag

9361 tataaagata aatactttta ctctagtttg caagaataag caagatatat ttgctacaag

9421 gaatgccaag aagaattagt gactaccctg atgcatttac aggatgaaat ttagttagta

9481 gtttcggatc tatagtaagt gttatagcta cagtattatt cttatacatt gtttacaaac

9541 aattaataga taataatcct gtaggtagat tcccttgatt aatgcctcaa tattttactg

9601 acgcattaca agcactatta aatagaaatt atcctagttt agagtgagca ttaactagcc

9661 cacctaaacc acacgccttc gtaagcttac ctttacaatc ttaaactaag ggaaaactct

9721 tctaattttt ttagtagact tctactaaaa aaaaaatact taatatattt tattcttgat

9781 ataatatact atattattat tattatcatg ctcataaaat aaaaaaaaaa cccatatata

9841 tatatatgtt acgttttcct actcttgtgt ctataataga ggttgtttta gtattagttc

9901 cagctctact tgcggtagcg tatgttacag ttgcagaaag aaaaactatg gctagtatgc

9961 aaagaagatt agggcctaac gctgtaggtt attatggact attgcaagcc tttgcagatg

10021 ctttaaaact tattttaaaa gaaaatattg cacctaatca agctaatctt actcttttct

10081 ttttaggacc tgttataact ttagtgtttt cattactagg ttacggtgtt ataccttacg

10141 gaccaggttt agctattagt gatttaaatt taggtatatt ctatttatta gcagtatctt

10201 ctttatctac atatggtata ctattagcgg gatgaagtgc taacagtaaa tatgctttct

10261 taggttcttt aagaagtaca gctcagttaa ttagttacga attagtttta agttcagcta

10321 tattattagt agtatttata acaggaagtt taaatttaac tgtaaatatg gaagcacaaa

10381 gagctgtttg atttatatta cctttattac ctatatttat tatatttttt atagcctctg

10441 tagcagaaac taatagagct ccttttgatt tagctgaggc tagtctagat ttggcctcat

10501 taaagatcac aaattgctgg aaatctttat taagataatc agcaggaaag cttctttgta

10561 cacgcaagtt caaagttgaa tcctcagaga ctagaggtga tcatttaata ttgaatatta

10621 aatatggtat agtccaaaca tctacgtaag tagttgatta atgctattta aagttataca

10681 ctcttgataa atgcgtttca acctgtaatt tgtcaattac cccctgtaaa gtaactaaaa

10741 gattttattc ttctaaatct tcttctagtg gactttcaga tattaatcct gtacctgttt

10801 tagttttata taaattagca gataaatctt atattaattc atttagaaat tcattaaaag

10861 ataaaggagg tatttactct tttataaata cagttaatgg taaacaatat ataggaagtg

10921 caaaagattt atatattaga ttaaatgagc atttaagtaa caaaaaatct aatttacttt

10981 tgcaagccgc ttttaataaa tatggattag ataaatttaa ttgaattgtt tatgagtatt

11041 ttacctatga aagtaaaatt ataagtaata aatctctcac tgatttagag acaagttaca

11101 taagaagttt tgatttttct actctttata attttaaagt tgaagctact agtatgttag

11161 gatataaaca tacagatgaa gctaaacaaa aaatggtaga aagatttaaa gatattacta

11221 accatcccat gtacggtaaa aaccatagcg aggaaatatt aaaattaata agtaaaccag

11281 ggaagttaaa tcctatgtat ggtaaaaaac atagtgatga aactaaaaaa gccatttctc

11341 taaaaatgag caaataccct aacggtgtag gtatttttga tttaaataat aatttaatta

11401 ataagtttag caataatact gagatagccg attacttagg tatatccaaa gttactgttg

11461 ctaaatattt aaataatgat ttaatatata aaggtatgta caaatttaaa cccatattat

11521 cataagcgta taactttaaa attttttgga atcagaactt gttagcgggt ttatgacaga

11581 acacgctgct gttgtatttg tatttttttt cttggcagag tacggtagta ttgtattaat

11641 gtgtatatta acaagtatac tattcatagg aggatattta atcataggta gttctataat

11701 agatataagc ttatatatct atgattctaa ttttataata aattatatta taaatagtcc

11761 tattatagaa ggtttattat acggtataag tttaggtatt aaaagttcta taatgatatt

11821 tgtatttatt tgaacaagag cttcattccc tcggataaga tttgatcaat taatgtcatt

11881 ctgttgaact gtattactac ctgttctttt tgcaataata atattaatac ctgctatatt

11941 atataatttt atattattcc ctattaatat tagtttacta tagtatagta tgtatttatt

12001 aaaaaccata agtattctta tatatggtgt atattttcat agtaaaataa aatattttag

12061 gcttttatac taatagcaaa tataaaagag aatgttatta tcttttttat tattagtacc

12121 tataataggt atttttttaa tcgcaggaac tatatcttat caagataatg taataaaaac

12181 tacatactac aaaaatatag ctttaattac atctataata aatttaatta tttctttagt

12241 ggtgtatatg ttattcgact ctaatacaaa tcaatttcaa tttgttcaag agcattataa

12301 cctaagttac ttcgatattt atttaggggt agatggtatt tcaatatatt ttgtactatt

12361 aactactata ataatgccaa tagctatatt agctaattga aactctataa ctgataatat

12421 aaaatcatat ctaataataa tgttattatt agaaacacta ttattagcaa tattcttagt

12481 tttagatata ttattattct atattttttt tgaaagtaca ctacctccct tatttttatt

12541 agtaggttta tttgggtcaa ataataaagt tagagctagt ttctacttgt ttttatacac

12601 actatgaggt tctttatttc tattaatagg gatattagct atatcttcta taatgggaac

12661 tacagatttc gatgctttat ttaaaactaa ttttgattat attactcaat tagtcctatt

12721 tagtggaatt ttcttatctt ttgctgtaaa aacaccaact atttttttaa atagttgatt

12781 attaaaagct cacgttgaat cacctttagg tggaagtata gtattagctg ctatagtatt

12841 aaaaactagt ttatatggta tatttagatt aattttacct atactaccta aagcatctat

12901 taattttact tatatagtat acgttatagg tgttattaca ataatatatg ctagttttag

12961 tacattaaga actacagata ttaaagaatt aattgcttat agttctgtat ctcatgcggc

13021 agtatacttg atgggtgtat ttagtaatac aatacaaggt attgaaggag gtatacttct

13081 aggtttaggt catggatttg tttcaagtgg tttatttata tgtgctggag gtatattata

13141 cgatagatct ggaactagat ctatattttt ttataaaggt atagctcaaa taatgccatt

13201 gtttgcgata ttattcttaa tactatcatt aggtaattgt ggagttcctt taacattcaa

13261 ttttgtaggt gaatttatgt ctctatatgg ggcattcgaa agattacctt tattaggtgt

13321 atttgctagt tcttctatta tattatcagc ggcttatact atgtatatgt ttaatagaat

13381 agggtttgga ggtacattca gtaaatattt taaagaaaat atagttgatt taacaaaaag

13441 agaattcttt atcttattaa cattagtaat atttactgtt attttaggta tataccctag

13501 tatagtgtta gatggtcttc attatagtgt ttcaggttta atttataatc aataaataaa

13561 aatagtctaa tatatatata tatatatata atctttctta gcataccata aatgaatgga

13621 attgaacaag aagaattact tggatgctga tgagactcat tttgctaatt aggtgcttta

13681 atgtttgata taatactatt attagttata taaaataact taaacaaata tcttatcatg

13741 tatttaaata aataattatt tttaatactt ctgtatttaa tattttaaaa gaaaaattta

13801 tgccacaact agtacctttt tattttttaa atgaagtaat atttactttt agtattatta

13861 ctataatcgt atatttattt tctaaatata tattacctag attcgtacgt ttatttttat

13921 cacgtacatt tatatcaaaa ctatttgata ataaataata attataatat attataattt

13981 tgtattatac aaggattttt caagacatac tatatagctt aaagtacgat gaatacttta

14041 agtataaata cagttaactt agaaacactt agccctttaa gtcaatttga aataaaagac

14101 ttactaagta tagatgcacc tttattaggt aatttacata tttctttaac aaacatagga

14161 ttttatttat caattggagc ttttataata ttaactttaa gtttattaag tacaaattat

14221 aataaattaa taagtaataa ctgatctata agtcaggaaa cattatacgc aactgtacat

14281 agtatagtaa caaatcaaat aaacccaaga aatggtcaaa tgtacttccc ttttatttat

14341 acattattta ttttcatatt aattaataat ttaataggaa tggtaccata tagctttgct

14401 tcaactagcc attttgtttt aactttttct cttagtttta ctatagtttt aggtgctaca

14461 atattaggat ttcaaaaaca tgggttagaa ttcttttctt tactagtgcc agctggttgt

14521 cctttagcct tattaccttt attagtatta atagaattaa tatcatattt agctcgtaat

14581 atttctttag gtttaagatt agctgctaac ataatgtgag tataggtctc tcacttattg

14641 tgtttaaaga gccaaattcc ggggaagccc taaagctcta ataactaaac ttttaaagga

14701 aactttttaa gcggcccagt taatcactgt gggtatagta atatcattag agattctagc

14761 aataggaatg ggtaatcgcg gatctaaatc aaataaagat aagactttat ttgtaaaaga

14821 gcaacgagta gacggttctt cgataataat aatagttatt attgtaaggt gtactctggt

14881 cgccgggaaa tccggttctt ggaagaaact aagtaaatca agattaaaga taacacaata

14941 gcctatcctt aattatgtat tctatacaat aaatctattt tttcctttaa agaaagaata

15001 acttttaaat tttaaatgtt atatttttaa agaaaggata aagttgtgaa cgatttatat

15061 tatcagatac aaatgtacta attagtaaac ataaatataa taaattatat tataataata

15121 caagacaaca aggtttaatt aaattgactt catgaagaac aataaatact agatcttttt

15181 caacatcaaa tataaattca tcttcaaact ctattatttt ttctaacgca gatgaaaata

15241 aaccagatat acttaaaact attaaaggta aatctggtat ttacatgtga acaaaccaac

15301 taaatggaaa aaaatatgta ggaagttctg tagatttaag acgaagatta ttagaatatt

15361 ataatgtaaa gagattgtta aatgagacta gtatgccaat atgtactgca ttattaaaat

15421 acggatataa aaattttagt ttaactatat tagaattttg tgacattgag aaattaatgt

15481 taagagaaaa acactacttt gaagtgtatt ctcctgagta taatatactt aaaacaccag

15541 gtagtccaga tagaggttca ggttgaaaac attctgaggc tacaatagaa attatgcgtg

15601 tcgcagctca aaaaagaata gaatcatcaa aatatataaa taaattatcc gaagctcaac

15661 ttaatggaat tgaaattgaa gtaactgatt taaaaactaa tacttctact aaatatcatg

15721 ctataagagc tgctgctcgt gctttaaata tagataaaag atatattgaa aattatgttt

15781 atttaaatca agataagcct gttttagata gatatacttt taaattgtta tctgaaaaag

15841 aaacaaagta tatacctaaa gttcaaaaaa cttctataaa attagaggta actaatatag

15901 aaactaagga aattacaata tatccttcta taggtgcagc agctaaagag ttagggtttc

15961 atcaagctag tgtatcttta tatctaaaag aaaatagaat taaacctttt aaaggtttat

16021 atttatttaa aatagttaaa gataactaaa taatttattg taaggattaa tttgtgtacg

16081 aatgagtggt catatgttac ttcatatttt agctggtttc acatacaata taatgtcatc

16141 aggtataata ttctttttct taggtttatt acctttagca tttattatag ctttctctgg

16201 attagagata ggtatagcat ttatacaagc tcaagtattt gtagttttaa cttcttctta

16261 tataaaagat gggttagatt tacattaata aaattaaaac atttttaggg ataaacagtg

16321 taaagtcaaa tttagggtgc atattattag aaaaaatttt aatagtttta aatagaaaat

16381 attaatataa gtatatattt agataattta gtatcaaata ataaaattat attgcggaca

16441 tacaatctta tatgctactt ttttaattta gatagattaa actcgattac gcttatattt

16501 atcctaaaaa taaaaaatat tataaacctt taagtgatgt aattgattat ttaattacat

16561 ctgaaaatag atgagtttgg tgatggctct gattgaacac tgtccaaagg cttgacacat

16621 gctaatcgta cgtttaattt aattaaaatt ttattaaaag tggtgaacag gtgagtatat

16681 aattattaac ctaccttgag gtaaggtaca ataaatacct tatataataa aggaaaataa

16741 attccgcctt aagacgaatc gataatttac agggataggt agtagtaaag gtaacggctt

16801 cactagccta atattctctt agtcgaaact gagatggttg atcgaccaca ttgggaatga

16861 gaaaagccca aggcaaggaa gtacagcagt ggggaatttt ggtcaatggc ctaacggctg

16921 aactggcaat ttggaggaat gacctatctt atttagtaaa aaataagaat tagagtgaaa

16981 tggttcaatt aattattaaa tgaaattcta aatatataac attataatga ctaaatatat

17041 ttatgtcttg actaattacg tgccagcagt cgcggtaata cgtaagagac tagtgttatt

17101 catctttagt aggtttaaag ggtacttaaa cggaagaaat tacctaaaca aggtataatt

17161 tctatctaga gtttaatatg aaaaggtaga acttgaggag tagagattca attcgctaat

17221 acctaataaa ggacaagtga aggctaatgc gaccttttat gtaaaaactg acgttgaagg

17281 acgaaggctt agagagcgaa taggattaga gaccctagta gtctaagcag aaaatgatga

17341 atgccatagg ttagggttac ttagtctata aatgaaagtg taagcatttc acctcaagag

17401 taacatggca acatgggaac tgtaatcacc aggccgtttc tgacaacagt agtgaagtat

17461 gttacttaat tcgatgaccc acgaaaatct taccacgatc agtataatat attcataaca

17521 taatacgata taatatatta cgagtgttgc acggctgttt tcggttaatg ttgtgaaact

17581 gtggctgagg ccatgcaatt aacgtaaacc tttgctttat ttatatattt tttttttata

17641 aagctgtttt tatctggaaa acgataaaat aatagggcca aagacaagtc atcatgacct

17701 ttaaatcgtg ggctgtagac gtgccacatt ttcctagaca aagagaagca aaaatgtaaa

17761 tttaagctaa tctcaaaaaa taggataaaa aaagatatgg attgtagtct gaaactcgac

17821 tacataaata agtaattact agtaatcgtg aaccaccagg tcacggtgaa ttacatctcg

17881 gattggtact aattactcgt cgcacgctga aaggagtatg tgcaaaaagt ttgctttcct

17941 actataatta aataaataat agggtttaaa cattataata ggattcttcg tatgtatgac

18001 tctaattagt gttaagtcga aatatggttc gtgtagtgga agttgcacgg ggatttaatt

18061 tttttatgta aataatgtat aatttttata ttatacatat aaggagagtt cctttattgg

18121 taagaagggt tgagctgtaa actcaatagc tatatcagct ttaagagttc gaatctcttg

18181 tctcctaata ataaccttct atagctcaac ggtagagcat gatactgtta atatcaggat

18241 aaatgttcaa ttcattttag aagggcttat aatatgtatt atttaggact attataaata

18301 ttttaaataa aaggtttaac taaaaaaaaa taaagttttt aaatggatat aaaacgtaga

18361 agtatatttc aagatcatcc gtttcattta gtgtctccat ctccttgacc atttttcact

18421 agtttcagtt tacttggttt aacttcaaat atggcgttat acatgcataa ttttgattat

18481 gcttcttacc tttcatttgt atcactattc tgtctaataa tgtctatgtt tttctgattc

18541 aaagatgtta caactgaggg tacatattta ggtgatcaca ctttagctgt acaacaagga

18601 ctaaacgtag gtataatgtt atttatagtt tctgaagctt ttttcttcct tgctatattt

18661 tgagcctttt ttcatagtgc tttaagccct acagttgaac taggtgctca atgacctcct

18721 tttggtatac aacctgttaa tccttttgaa ttacctttac ttaatacagt gctattatta

18781 tctagcggag ctactattac atatgctcat cattctttat tatctggaga tagaccagat

18841 gctgtatatt gaagtatagt tagtattgtt ttagctatta tatttactgt ttttcaaggt

18901 gtagaatatg cagtatcatc ttttactatt agtgatagtg tttatggtgc atgttttttt

18961 ttcggaactg ggtttcatgg gatacatgtg atgattggtt cgcttttttt attgatagct

19021 ttgatgttga ttgttactta tcatgcgact gacagtcatc atcagggtta tgaatctgcg

19081 atattgtatt gacactttgt agatgtagtg tgacttttcc tttatatagc aatgtattat

19141 tgaggttcat aaatccacta tggacaaaaa tttcattata tagcaatgta ttattgaagt

19201 tcataaatac actatgaaca aaattaattt cattatgtaa aaatgtatta ttgagattca

19261 taaatccact atggacaaaa ttaatttcat tatgtagaaa tatcttattg agattcataa

19321 atccactatg gacaaaatta atatcactat gaagaaatgt attattgaga ttcataaatc

19381 cactatggac aaaattaata ccattaagaa gtaaattaga tagaagtgaa gtattagcct

19441 ctatattatc aactagttta gtattaattt gtgtactaaa ccctgatcta actgaggtag

19501 tattccatac tactattttt tgtacatatg catatgttgc atataataca ggtattataa

19561 gtaaaatata tactattttt aaaactcata aaataaaatt ttgtttatta gttatattta

19621 tttttattgg tataatattt atttttattg gtataatatt atgaaatatt attgatagtt

19681 tagtattcat tactggagga cagggttcaa actttaataa taattttcca aattttaata

19741 ataataataa taatggacct aatccccctc aaaataatga tatggtggta gcaagtgggt

19801 cacataacga tgaacataat cgtcgtaata ctagaatttg aaataataat cctccattta

19861 ttaatcgtcc cgcaacacct ctatttaatg cacctagtga tgcatctgct tctcttatgg

19921 ttaatgcacc taatagtgta tctgctactc ctgcagttaa tactactact gtagctaatg

19981 ctactcctgt agttaatgct gtagttaatg ctactcctgt agttaatgcg cctagtgatg

20041 catctgcttc ttctgcagtc tctaatcgcc ctcttatcgt atatagtagt gatactgttc

20101 ctggccctga ctctgttatt ttacgtcata gtgattttgc tcctgtctat agaagtcacg

20161 atgaagctat ggcttacgta gcttcacgta caagtagatt tatatttgaa gataatcgtg

20221 actatctaac tggaatacct gaaggtcgtc caaatttctg atttaatgaa cagggtagac

20281 ttatgagttc tcttagtact aatatatacg ttcatggtcc ctatgaagat cttcactatg

20341 ttagacatat tcccttatga catgtgagat ataaccttca tgatatacct tttttaactc

20401 aaggacctcc tgtgtattat gttgatgata tgtctagtta tgttaatact attcctacat

20461 caggccctca tcacctacct aatacttaca caaattatcc attttatatg aaacattata

20521 ataatccaat gagtagatta gagatagaag cagcaataga taatctaatt aaattacatt

20581 ttaataattc tatacaaata gcccggtctg aaaacgatat attaagccca ggtacacctc

20641 atactggtaa aaatatactt atagagaaag tggttaatct tagagacaga aatatgtata

20701 taagtattga tcattatctt ttattaaaga aatgacattg aaacaccttt taatcgctta

20761 ggttttattt attattaata ttaagatata tatattaata atataataag ggatcttcgt

20821 ataatggtaa tacatgtgac ttttaatcat taaattgtca gttcgaatct ggcagatcct

20881 aagtatataa aagaattagt aacttaattg gtaaaggact tccttgtcac ggaagtagat

20941 atgggttcga atcccacctg attcgcgata tgacagtatt aaatgattat tttatgtagt

21001 tttcagctga tttttaggat agtgtggttg aagctaaaga tttaggttgt aaattcgata

21061 taatgaagaa aaaggtttta ttggttataa ataaaatcaa agagtaatcg gttcaaatcc

21121 gatttaattc gcaaaaagga aaaattgcta ttggtagggt aaagtatttg ctatatactg

21181 tgtttttcac ttgaaagttc gattctttct ttttccgtcc aagagtatag tttaatggta

21241 aaacgaagag cttcaacctc tgaattctct gttcaaatca gagtactctt gaataaaaat

21301 ttacaatgtt ccctgtctat ttaggtatta tattatagta attattatga ataacttatt

21361 tattataaat gaaaatttta caaacggcta tagaagtgaa gtattagata tactttgctt

21421 attaattatt ttatctggaa tatttgtaat agtaagtaaa aatcctatta tatctttatt

21481 gtttttaata ggactatttg cgggtgtttc ttgttattta cttataatag gtttaaattt

21541 tttaggacta tcttatttag ttgtttatat tggtgcagtg tctatattat ttttatttat

21601 tttgatgtta attaatatca gaatgagtga attacagagt aatactagca atagtatacc

21661 tttagctata agcattgcaa ttttatttaa ttaccctctg ttccaattat tgccttataa

21721 tgttgctata ctaaataata ataataatat tataaataat atattatata atgtatcatt

21781 taacaaaata gataatggat taaaaactaa tttagatata aataataatg atacattatt

21841 tgtaacaagt aaaatatgag atgctaattt agctgaagtg ggacatatat caagtatagg

21901 taatatattt tatactaatt ataatatatg attaataata gttggtttta ttttactgtt

21961 agctatggtt ggatcaataa caatcgttat gaaacaaaaa aattaaatac caataaaaaa

22021 aaaaggaatt aactcaatgg tagagtgacc gtcctacaca cggcaagtta tcagttcaaa

22081 tctgatattc cttaaattcc ttaacttaat ggtaaagtgt acttttgata aaagtattat

22141 cagtgttcaa ttcactgagg aattagtaga agaaggttgg ctgagtgggt taaagcgact

22201 gttttgaatg cagttaaggt taaaactttc aagagttcga atctcttacc ttctgataga

22261 cacaaatcct agttcaatta gtaaaacgta ttctttggga gaatatattc tgagtgcgag

22321 tctcaggggt ttgatttata tatatatata tttatatata tattaaatat tttatagaaa

22381 tataaatgat acgaaataaa aatttataca tataattatg taagtatata aagagactat

22441 taggtaatac tagctatata ttaaagagaa acatgaattt atcaaaaaaa aaaagaattg

22501 tgataataaa ctctaagata aatcttaata aaaaaacgaa gtgaattgaa ttatcttatt

22561 agcttcagga aaaaaaaatc aaaagagatt ctatgagtag tgtgaacaaa aatagaaaag

22621 tctaaataat aagtaaaatt gccataaagc tgtttgaata ttatggggaa ccttcctcaa

22681 agactaaata taatatataa gcgatagcat atagtaccgt gagggaaagt aaataactag

22741 tagttttata agcagctcga gcgaaagcaa agagcgtacc ttttgcataa tgggtcacca

22801 agttaatttt agatgcgagc taatgcgtag ttaaaccgat catataaata atgaatagta

22861 tctaaagtta gacccgaagt acagtgatct taccataatc aggactataa aggtccgaac

22921 gggttattgt tgcaaaaata tccgaagaat tgtggtaagt atagtgaaag acaattctga

22981 ctgtattagc tggttttctg cgaaacctat actagtaggc aatttaaata acatcttagc

23041 aggtacagaa cttaatctca gacaagatgt aataaacatt ttctttttgt acaaatcggg

23101 gaatcatgag gattttaccg gtgagtttgt ggactcggaa tggcaaagat gaattttaaa

23161 ttatcagaca tataatgata aggttgtatg tcaaaaggga aacagcccag aacaagagtt

23221 aaggttccaa aattattatt aagtgaaatt aagaaggtct atatgtaaac cgacaaggag

23281 attggcttag aagcagccat aatttaaaga cctcgtacca gagcactagt ctacgtaaaa

23341 agcgttaaaa atttaacgga tctaaataat ataccgatac cttgtctata aatgataata

23401 ataattatta ttatttatgg ggtagcagaa cgttgagtaa attttagtgt ttttttttat

23461 aaaaaaaatt atattataac tcaagtgaga atggtgacat gagtaacgaa aaagaaaact

23521 cgcctaaagc ttatggttag gattaagtat cggcctctaa gtttataccc taaggggtaa

23581 agcgatgaga aaaccttatt actattaaag acaccatttt ttaagtgaaa aatgagcttg

23641 aaaaaagtag taaaaaatta accgtaccta ggatctacaa caagtaagct agtagagaat

23701 acgaaggcgc atgagataac aatcttaaag gaactcggca aaatgactcc gtaacttcgg

23761 gataaggagg gctcatttat tctgattaat atcagatata gaggaagaat cataaaatag

23821 tgttgtacga ctgtttaatt aaaacacagc actttgcaga gatgttaaat ctaagtatca

23881 agtgtgaaat ctgcccaatt ccggctaata actaacttag ttaaatttat ataaatttag

23941 ttaataagaa acaaccggca aatggcggcc ttaacctgag ggtcctaagg tagcgaaatg

24001 ccttggccgt taaatgcggt cttgcacgaa tgatgtaacg atacaacagc tgtctctaag

24061 attgactcag tgaaattgga ataactgtgc agatacagtt tacctctaga tagacgagaa

24121 gaccctatgc agctttactg ttactagtta ttgaatataa ttaagtttat tattagttta

24181 aaaggtttat tgataaaata aaagtgaaag acctttattt acttattatg ttaatacttt

24241 tgttaatctg gacagtgact agtagacagt ttatgtgggg cacagacccc ttaaagagta

24301 aaagggtgta tctaaaatat ataacttaaa gtttatttta atttttttgt ttgttattat

24361 tttaactttg ttatatttaa tcttttcttt tgtgtatgga aatgtatatt atatgtacat

24421 agatatagag ttaggtagga ttttaattat ggtgcaaatt aattgtatat aaaagataca

24481 tatctgtata ttatctttaa tagttattat aatatttaat tattaataat tatgtttaat

24541 acttataaag ttgaatggct taatcttgct ttactgtttg attaacaaca aatcttacag

24601 tcgcgtaagc ggggcattag atcacaagat acaataagga aaggtcttgg attattggaa

24661 aagctacgct agggatgttt gtccttcaat atttttttaa taattgatac atattgggtt

24721 aatttcaaaa attacttact ataagtgtaa gtagatttga agcgaagttt atttatgcat

24781 ataaataaaa ttgttcaacg actaaaagta ataatacttt attaataccc aacatttttt

24841 tttagtaatt aattttaata ttttaaacga aaattgaaaa aaaacactaa attttaccaa

24901 aataatatat ttattaaaaa tttaaataat aaatataaat taatacctat gaatataaaa

24961 aataatttta taggtgaaaa taattatttt ccttctgatt ataaagaatg aaataataat

25021 atatattatt ttaataataa taatattaaa aatataccag cctatgattt aataataaat

25081 aaattattaa aaggttattt taattcatat ttaaatccga atattaattt tttatattca

25141 aataaaagat acacatcttt aaataaaata tatattagta aagctaatat aaaacatact

25201 aattctaaag ctataataac tatttatgtt tacgatagag aaaaaataag tatatctaaa

25261 ttttttataa aatatagtta tattatatta gctgctatta aagaactaaa aggatttgta

25321 tttgaaaata ataaattttt tcaaaataga ttaaatagat ttcagggtta taataattta

25381 ttaaaactat taaatttttt taaaagtaga aaattaaaag cttgattaaa cgaattaaga

25441 tataaagata ttttaattta caaattaagt aaattaatca gtaaatttta taaaaaaaaa

25501 atagaattca atataataag attaacttct attgcatata attcagatat tttagtagat

25561 gtaatgagaa aaagctataa aaaaactgga catgtaagaa attcaatgaa atttatatta

25621 aatcaaggtc ttattgaaaa aatagataac actaatgata gagttagagc tactaaaaat

25681 gtagatttta atcttttagc taataaatat caaaatttaa atattaattc tatagtgaaa

25741 aacgtaaata taaatgaaac tataaaaaat ttatataaga ctaataataa aaataatgaa

25801 gaaattattt ttaattctat taaatataaa atattaggtg gtataagaat agatataaaa

25861 ggtagattaa ctagacggtt tagagcagat agatctgttt ctaacacaac cttaaaagga

25921 agttttagaa atatagattc ttcatataaa ggattaacaa ttttaggtta tagagggtgt

25981 gctagctcta gtatagatta ttcaatgtct atatctaaac gtcatgttgg agcatttgct

26041 ataaaaggtt gagttagtgg aagataatat aaaatattaa ggtaaaaaaa aaaaaaatga

26101 agaaatagtc tgaaccattt tgtgaaaaat ggaaataaaa ttttatgata acaagttgaa

26161 caggctaatt tgcgcaagag tgaacgtaat gagtgcgcgg tttggcacct cgatgtcggc

26221 ttaacttatc ctcgtggatg cagaaactat gaagggtacg actgttcgtc gattaaaaag

26281 ttacatgagc tgggttaaat acgtcgtgag acagtatggt ttctatcttc taggggaaat

26341 tagaatataa taaggattaa ccttgtacga aaggaacgtg ggcaacgcct tatttgtaaa

26401 aagaataatg gttttgttaa cctatggttt atctgttgtt tatgtgccca gatattaatt

26461 taggtataaa ttttatacag ggatgttact ttcacttaag taaatatata acataggcat

26521 agcagaaaag ctaagttagc cagagataag tgttgaaagc atatatatac gaggctcgcc

26581 ttaagatatt tcttaaatat acgtaaaaga atattacgaa cttcataggc ttagtttgta

26641 aggatttaga gatctttaga tactaagtac taattttata aataactgaa tatttaccgt

26701 atcttatatt tagctcagtt agcataaaag taatgcaatt gttttgtaat caatggacac

26761 aagtgcgata cttgtactgg gcttaggact agtagacaag tggctatgtc atagcttttt

26821 caatgctaat atcgcgggtt cgaatcctgc ctagtctaag aggctgtaac ttaattggta

26881 gagtgtactg ctcatgacag taattgtaag tgttcaactc acttcagcct tatttagcgg

26941 gttagtgtaa taggaacatt tttgactcat gatcagaaga tataggtgcg attcctgtat

27001 ccgcttatta tccgagtggt ggaattggta gacacaagag atttaaaccc tcttggtgaa

27061 taaccgtgta cgttcaaatc gtacctcgga tatagttaag caactgtagt ttaatggtaa

27121 aatgatcgtc ttccacacga atgatatcgg ttcaattccg attagttgta taaaggggat

27181 atagtataat tggtattaca gcaattttgc atattgttta tttaggttca agtcctaata

27241 tctccattga aagcccgata agctcaattg gttgagcgag acactgaagc tgtctaggtt

27301 gtaagttcga atcttacatc gggcaaaata aatatattat attcaatgaa gcgggtgtgc

27361 tggaattggt agacaggctc tgtttaggcc agagtgggta aaaccgtata agttcaagtc

27421 ttattgtccg tagtaataaa attagtttaa agtattttat atttattatt ttaatttgga

27481 gtagacaaat gggtatgtca tagatttttg gtgtctaaga ttgagtgttc gaatcactcc

27541 tccaaaattt tgtgataaat tagcgtttag tattcaaata aaagaagaat aggataatat

27601 agctcaattg gtggagcgac tgtatgtggc acagtaggtt ctctgttcga ttcagagtat

27661 tttcccttat gttaagacat atatttatat tgcttgagta gtttaaccgg ttaaaacctt

27721 attttcatac gataatgatg ggtattcgac ttacccctca ggctaatcat tttgttatat

27781 aattactgaa aaagaataat attaattgac tattataata gccggggtag tttaagaggt

27841 aaaactttaa tttcatatat taaaaatgag aattcgattt tctctcttgg cttacttgta

27901 tatctatata tatataagta tatcaaaaaa aaaatatgct tttaatttca atattatctt

27961 tattactttc taatgccgtt acaatacgac gagacatatc aatacttttt aatagagttg

28021 ctataattgc tttaatttat tctatattac acagtgtaac aactttattt attataggta

28081 aaggtatagg tttacacgga ggtttactta atgtcactag tataacacaa gtttttaata

28141 tttttatatt cttagtaagt atattaatat tacaattaac tagttttttt cctagaaaag

28201 tatgaatacc agaacattct tcattaatac aattattatt taataattta gtattttata

28261 gaacaaaagt tataaataaa atgggagaac acttaagaat tatagaatat cctttaatac

28321 tattatttat aattagcggt gcagtatttt taatatctac tagtgattta atctctgttt

28381 ttcttgctat agaattacag agttacggac tatatctact aagtactata tatagaaatt

28441 ctgaactatc tacaacagga gggttaatgt atttcttatt aggtggttta agttcatgtt

28501 tcattttatt aggtacaagt ttattatacg ctaattctgg aactactaat ttagacgcta

28561 tatatgctat aactagtctt agtgatagtg cagatatatg atataaacca tattacatta

28621 actttgcttt ccttatattc agtataggat ttttatttaa agtaagtgct gcccctttcc

28681 atttttgatc tcctgatgtc tatgacgcta tacctacaat agttactact tttgtagcta

28741 ttatagctaa aatatctata tttattttct tattagaaat agtttattac actaaaaatt

28801 attttacaga ttttaattga acatacggtc tattaataag ttctttctta tcaatgataa

28861 taggaacagt tgtaggttta actcaattta gaataaaaag attatttgcg tatagtacta

28921 ttagtcatgt agggtttatt ttattagctt taagtatatc tagtgtagaa tctactcaag

28981 catttatatt ttatctaatg caatattcta ttagtaattt aaatgcattc ttaatattag

29041 tagctatagg tttttcttta tattgttaca ttaataataa taaagaatat aaagacctct

29101 tagataaaaa taattcccca atacaactga ttagtcaatt aaaaggttat ttttatataa

29161 accctacatt agctttaagt ttaactgtta ctcttttttc ttttgcaggt ataccacctc

29221 ttgccggatt tttcgctaaa cagatggtat taagtgcagc tatagataat ggatatatat

29281 ttattacttt agttgctata tctactagtg ttataggagc tgtttattat ataaatataa

29341 taaaagaaat ttttttttat tcaccagaat ataaaataaa tcctctttta gaaaatttaa

29401 attttaatgg taatatatat aacaataaaa atgtattaat taaatcaatt aactttaaat

29461 ataataatat tgtaatatct agttctatat ctattacaat atctattatt acattaataa

29521 tattattatt tatatttgta aataaagaat gattaagtat gagtaccata ttggtacaat

29581 atttatttaa ttattaaatg agtagtatga gtatattttt tatatttgta attgtagtgg

29641 caatactttt tttagcttta aatttaatat ttgcacctag taatccttac caagaaaagt

29701 attcagcttt cgaatgtggg tttcattctt tttcacaatc tagatcacaa tttaatataa

29761 ctttctttat atatgcttta gtttttttac ttttagattt agaaatatta ttattatacc

29821 cttacgctgt tagttcttat tctaatgata tttatgggct aattatagta ttaatattta

29881 ccactatagt aacgataggt tttgtatttg aattaggtaa aggagcctta aaaataggta

29941 gccgtcaaga cgtggagtct attagtaaaa aaaataatat tattatatct ttaataggta

30001 caaaaaagta aatttatgta aacggtagat tatataaata agtattaact ttaaaattag

30061 ctcttttaat agtatctaaa tacttataaa aatcatctct gaaaagaaat aataatttac

30121 aaaatcagga tttaatacat tcgtattaat atttgctggt aagtgaactt tattagtatc

30181 aaataattct ttatattgat ttagtaaaaa ttcttctaat ttaatttgtt gttctagttt

30241 agtaagatta ttgtcactta atatactatt aatacccatt aataattcct cattaaataa

30301 ataattagat gagctagcta cacttggatt caagatagca tgaaaatgtc taaaaaaagc

30361 cgagatctgc aatctgggta taaaaagttt tttcaacatt ttagttttat tcatggttta

30421 ttagtatgta ttaacctaaa ttattagcaa cgtaaaaatt attaacatca taattatgat

30481 tatgataaaa tatatatctt ctaaactaaa tcttaaagaa agtcgttctg aggaagaaag

30541 aaaaatatat acatacatac tttataagta tacactattt actttatgat attattaaaa

30601 tatatttagt atgatacaag ttgctaaaat aataggtaca ggattagcta ctacaggatt

30661 aattggagca ggtgtaggga ttggtgtagt ttttggggct ttaatattag gtgtagctag

30721 aaatccttca atgagaggtc aactattttc ttatgctatt ttagggtttg cattctctga

30781 agccactgga ttaagtgaat agttcagtat aaatcgcgta aattgcaaga agtatctaat

30841 tgattaactt gcagcgaaaa tagttatata actattacgt tcaacgacta ataattcggt

30901 agtttttact aaagacatag tctgatcaat aaagtaattt attgaattaa cagtactaat

30961 agcatacttc aataatatat atttatggat attttttcga gttagtttta ttatcttatg

31021 ttaccttatg ttattactta attctaaata atactttaga aatctataat ttaaaaataa

31081 taaatgaaaa ttaattataa aatttatata aaaaataatt ttacattagc acgtcaattt

31141 agtaatcata ctaacgatat ttacagttat tctataaaaa catataagga tcctttaaat

31201 caacgtaagt taatacggga tgaaaataat ggtaaaacag gtgtgtattg ttgaataaat

31261 aatataaacg gtaaatatta tataggtagt ggtgatccac tatatttaag aataagtgat

31321 tactttcaag attgatacat tttatctaga acaaatttat atatagttag agcgatatct

31381 aaatatggta tagctagttt ttctttaatt atattagaat attcaaattc agataatgtc

31441 attaaatctg aacaaaaatg aatagattta ctaaaacctg attataatct tagccctaca

31501 gcaggtaaca caaagggtta tagacataga attgaaagta ttgaaaagat gagagataag

31561 gctttaggta gaaaacatac agaagaagta aaacaatcta tgagtgaatc acgtaaagga

31621 gaaaataatc ctttttatgg taaaacccat agtcaagata ctttagtatt attaaaaaca

31681 gcggcagcta atagaactaa accccctgta ccgggtatag aagtggatat aatagatatt

31741 gaaactaaat taactcatac ttttgagtct atacgtaaag cagctagctt tatgggttct

31801 gatattaaaa cagtattacg ccgtgaagaa ttacaaaata ctaaaggtat taatactccc

31861 tacaaaaaga aatacataat tattataaaa agaagttaat tattagaata tgtattcgct

31921 ttaatgatgg cattcttatt attatacgta gcttaatata ataataaaat attaatgtta

31981 ttacttaatt ctatatagta aaaatatatt tatatataat atgtagaaaa ataaataaat

32041 aaattatgta ttactttttt attaatttaa tgattaaatt agacgcacct agcccttgag

32101 gaatatattt ccaagatagt gcaactcccc aaatggaagg attagtagag ttacatgata

32161 atataatgta ttacttagta ataatattat ttggtgtagg atgaatttta ttatctatca

32221 ctaaaaattt tattattagt gcttctccta tatcacacaa atatttaaac cacggtacat

32281 taatagaatt aatatgaact attactcctg ctgtaatatt gatattaata gctttccctt

32341 catttaaatt attatactta atggatgaag ttagtgatcc atctatgtct atattagcgg

32401 aaggtcatca atgatactga agttaccaat atccagattt cttagattct agtgaagaat

32461 ttattgaatt tgattcttac attgtaccag aatctgattt agaagaagga ggtttaagaa

32521 tgttagaggt agacactaga gttatagtac ctgaattaac acacattaga tttattatta

32581 catcaggaga tgttatacat tcttttgctt gtccatcttt aggtattaaa actgatgctt

32641 accctggtag attaaaccaa gtttctgttt ttataaatag agaaggagta ttctacggac

32701 aatgttcaga aatatgtggt atattacaca gttcaatgcc tattgttatt gaatctgtat

32761 ctttagaaaa attcttaaca tgacttgaag aacaataata taaatttata aatataaatt

32821 tatataaagg tgtgttagct taattggtta agcatagtac tacggatgct ataaatatga

32881 gttcgagtct cgtacacatc tctcttaagt ctgcttaaga aaatatataa atgtatatat

32941 atatatatac caaataatta atttataaat atatttatta tgaatataac cttaatactt

33001 tttttaatag gaattttagg atttgtttta aatagaaaaa atattatatt aatgcttatt

33061 tctatagaaa taatgctttt atctataaca ttcctaatat tgataagttc acttaatatt

33121 gatgatataa taggtcaaac ctacgcaatt tacataatag ttgtagcagg agctgagtca

33181 gcaattggcc taggtatttt agttgccttt tatagattaa atcgttcttt gttttcttac

33241 tatctattct acaattacac tccaatgtat ttatataaaa aaatccctca acaaataata

33301 ttaggatcaa aatattaaaa ttatttagat ttcaatcaag cagccgaaat aataataaat

33361 aaggaacatt taaataaaga ggggttagaa aagattttat tcttaaaaaa taaaatgaca

33421 acaaaggaaa aattgtaaat aatcataggt aagacagggc ccaaagaaca tatgatcaag

33481 gtgaagtaaa ccttcatcta gttttcctag tgaaacaaac tatttcgtta tagttagcaa

33541 acctaggtaa gcaaaaccgt caaattgcgg gaagttctta aagacaaatc taccaaatta

33601 ttatagtaat ataataatgg aacaggtaat gactcgttgt atggtaataa cgatttgtat

33661 gaagaaatga aatagataat ccgcagccaa gcacctatta cttaaaacaa gtaaggtgtg

33721 cagttcatcg actaaatggt ggttggtatt attataataa tgcttaagat atagtcaggc

33781 ataacttaat agttatggaa ttaccctagc cttcctaagg aaattatatt tctatggaaa

33841 aggggaaaac gaagaggaag tatagcaata gaatataaat aatgtattta agtataataa

33901 ttttaccttt attaggatct atagtttctg gcttttttgg tagaaaagtt ggagttagcg

33961 gtgcacaaat tataacatgt tcaagtataa caattactac tattttagct attatagctt

34021 tttttgaagt aggtattaat aacattgtat tgtctattaa tttatttaga tgaatagaca

34081 gtgaatgatt taatataatt tgaggttttc aatttgatag tttaacagtt tctatgttaa

34141 tacctgtatt aataattagt acattagttc atttttactc tataggttat atgagtggag

34201 accctcataa tcaaagattc tttagttatt taagtttatt cacatttatg atgataatac

34261 tagtaacagc taataattat ttattaatgt ttgtagggtg agaaggggtt ggagtttgtt

34321 cttatcttct agtgagtttt tgatttacta gaatagcagc aaatcaaagt tctatgtctg

34381 cctttataac taatagggtt ggtgattgtt ttttagttat agggatgttt gcaatattat

34441 gatctctagg taatttagat tatagtacag tattttcatt agcaccttat gttaatgaaa

34501 atataataat aataatagga gtttgtctat taataggtgc tatggctaaa agttctcaag

34561 ttggtctcca cgtttgatta cccatggcta tggagggttt agtaacaagg gctctcctaa

34621 aacttcacta tatgcgggaa caccccgttt taacattatg gtccactgtt tattctataa

34681 gcttcggaaa aatctataat gaggggcaat ccgcaggaaa ccaaaataag ggttcctcag

34741 agactacatg tgaagcagat acattagata ataaatttaa atgatgattt ataggattta

34801 ctgaaggaga cggatcattt attataaata atactggtta tttagagttt aaaataacac

34861 aatctagtct agatgctcaa gtattatttt atataaagaa aactttaggg tttggttctg

34921 tatccgtaca aagtaaaatt aacaatactc accaatttag agttagagat aaaaaaacaa

34981 tattaaattt aattaatata ttcaatggta atattataac taaatataaa aatcaacaat

35041 tcaacttatg attagaagct tttaataaaa tatataatac aaatattgaa tatatagcac

35101 caaagtacaa agtaaattta gataatgcct gattatctgg gtttactgat gctgaaggtt

35161 gttttactag ttctgcttat ttagctaaaa cgggtaaaca tatagttact gttagatatg

35221 ttatttctca aaaaaatgat atagaattta gtaatgactt agctaattta ataaatggat

35281 atgttacaca tattaaaagt tataatggtt ataatactgt tgtaaattta ggtaaattga

35341 atataatttt aaagtacttt ataacttacc agttaaaaac taaaaaactt atatcatata

35401 aaaggtgatt aaaagtttat aatttagtta aaaataaaga acatttaact gaagaaggta

35461 ttgaaaaaat aaaatcatta attaaattaa tcaacaaata gtgtatctga agatatagtc

35521 cgaacaacta tgaaagtagt tgagcatttt taccgtatac gtataaatgt aataatattt

35581 atatatagta aaaatcaaca aatttgccta ctcctgtttc tgctttaata cacgcagcta

35641 ctatggttac agcaggtgta tacttattaa tgcgttcttc tcctttaatt gagtatagtt

35701 ctactatatt attactttgt ttatgattag gggctataac tactgtattt agttcacttg

35761 taggattttt ccaacaggac ataaaaaaag ttatagctta ttcaacaatg tctcaattag

35821 gaatgatggt tattgctgta ggtttatctt catataatgt agctttattc catttagtta

35881 atcacgcttt ttataaaggg ttattattct taggagctgg ggcagttata catgccatgg

35941 ctgataatca agattttaga aaatacgggg gtttaatatc ttttttacct ttaagttatt

36001 ctgttatttt aatcgctagt ttaagtttag tagctttccc ttttatgact ggattttata

36061 gtaaagattt tatattagaa tctgcttacg gtcaatatta ctttagtagt ataactgttt

36121 atattatagc cgtaataggt gctatattta ctacattata ttcagttaaa gttctctatt

36181 taactttctt aactaaccct aatggacctt taataaatta taaacatgcc cacgaaagtg

36241 atatatttat gagtttacct ctagttatat tagctatatt ctctatattt tttgggtttt

36301 taactaaaga catatttata ggtttagggt ctggattctt tatagataat agtatattta

36361 ttcatcctaa ttctgaaata atgattgata ctgaatttgg agtatcaaca tattgaaaat

36421 tattgccttt tgtgtttact gtttcattta gtacaatagc tattatatta tctgaattct

36481 tgtctgaaaa tatagttaat ttcaaactat ctacaatagg taaaactatt ttcgggttct

36541 ttaaccaaag atttttagta gaatttttct ataataaata tattattaac ttaatattta

36601 aattaggtgg tcaaactgtt aaagttttag ataaaggtag tatagaatta ttagggcctt

36661 atggtttaga aaaaaaatta attaattcaa gtaaaaatat tagtagttta aataaaggta

36721 ttgtaacaaa ctatgcttta tttattctag taggattcat actttatatg ttttctataa

36781 ctttaggatt tactaataat ttattcttaa taatattaat tttaattttt attattagtt

36841 caatttatta ataattaaaa ttaatcatta aaataattta gtacatgata cataataata

36901 cctatatata acggtcctta tagtaaatta taaaataata tatatatata aatgagagtt

36961 ttaaagaata atagtatatt aaaattagcc aattcgtatc ttatagatgc ttctcaacct

37021 agtaatataa gttatttatg aaattttggt tcattattgg cagtttgttt aggaatacaa

37081 attataacag gtgtaacatt agctatgcat tatagcccaa gtattttaga agcttttaac

37141 tctgtagaac atattatgag agacgtaaat aatggatgat tagttcgtta tttacacagt

37201 aatactgctt cagctttctt ctttttagtt tacttacata taggaagagg tatatattat

37261 gggtcatata gatcaccgag aacattagtt tgaacaatag gtgtagtaat tcttatactt

37321 atgataggta taggtttcct gggttacttt acaatagccc aaaatgacta taaaaattta

37381 tataatagta aaacaataaa aacattcaac aataaaagat attattccac atcccataat

37441 tctgtaagta attttttagc ctctaaaaac attaaacctg tttttgtata cgataattta

37501 tccgaagatt cagtacgtag aaatatagct aaggaaacta aagaccttag tggtatttat

37561 atgatattaa acaaagaatc tttaaattat tatataggat ctgcttctac agggagattt

37621 aattctaggt ttacaaatca tttaatttat ttaaatggta gtaaagttct taaaaattct

37681 gtaaagaagt acggcttaga taattttgta tttataatat tagagttatt ccctgaaata

37741 gttaatcaag aaaataataa gaaattgtta gacttagaag acttttattt aaaatcactt

37801 ttacctgatt ataatatatt aactgaggct ggctctagtt ttggttataa acattcagaa

37861 gtaacaagaa taaaaatgaa agctaactat agtgaagaac gtagaaaaga aataggggaa

37921 ttaaacagag gaaagacttt ttctaatgaa actatagagg ctatgagaca atcagcttta

37981 aatagaaaaa agctaaatta tacagaacaa gggattttaa atatgaaaaa gtcttctaaa

38041 cctattttag ttaaagagct taataataca gtttatggtg aatttaacag tatagttgaa

38101 acagcagaag ctttaaactg ttcaactaaa actatacaaa gaacattaaa aagttctagt

38161 aaattattaa aaggacgttg aattgttaat tacattaaat aatattatag ttatagtcct

38221 gtgagtataa agttttatgt aatttatgga aaaaaataaa acttaaagca gaataacctg

38281 acagggttat tgaagaaata tttatgtttc tttggcgata ctagtgaaaa cgattaaaat

38341 atattaagaa attaatatac aagatcgtcg gttctccata gaatcgcgac agactgggtc

38401 actagtgggt ggctgaaatg ctgcttaatg cacagtcgga actatttatc catcgataaa

38461 tgtgatattc gaacaagaaa gatttcacag cttttaaaga cttcacatct ttaagagtaa

38521 gtttgtatgt actaccttac ggtcaaatgt cattatgagg tgcaacagtt attactaatc

38581 ttattagtgc tataccttga ataggacaag acatagttga gtttatttga ggagggtttt

38641 cagttagtaa tgcaacttta aatagattct ttgcattaca ttttgtatta ccttttgtat

38701 tagctgcttt agttttaatg catttaatag cacttcacga tacagctggg tcaggtaatc

38761 ctttaggggt agctggtact tacgatagaa taccttttgc tccttattac ctttttaaag

38821 atttaattac tatttttata tttatttttg ttttaagttt ctttgttttc tttatgccta

38881 atgttttagg tgactcagat aattatataa tggccaatcc tatgcagaca cctgcagcta

38941 ttgtacctga atgatattta ttacctttct atgcaatatt aagatctata cctaataaat

39001 tattaggtgt tattacaatg tttggagctt tagtaatttt acttacttta ccttatgttg

39061 atttaggtag atctagaggt ttccaattta gacctttaag taaattagct ttctttttat

39121 ttgttgtagt tttcttaact ttaggatttt taggggctaa acatgtagag tctccatata

39181 ttatcatggg gcaaatatat gcaatattat atttttctta tttcttagta atattaccta

39241 catttagtgt attagaaaat agtcttatgg atgtagccta tttaaaaaat aataataaat

39301 aataagtatc gatttatatt ggtaataata aaatattatt ataatcataa ttatatactt

39361 tattcattta atatattaaa aaaataaaca ctaaagcata tcatagagta aaaccactaa

39421 aacatagtat ttactatatt aatttaagta cttttaagta aatattaata atattttata

39481 tatgtatata taaatattta tatatatatg tatataagat tgcgaggtaa atggtttaca

39541 ctttgattgc agatcttaag tatgaggttc gattcctccg taatcttata gtacaccata

39601 aattcttatt agcttaaggg taaagcaaga tacttctaat atcaagattc tagttcgaat

39661 ctagaataag aattttttct aaattttttt tttcaaaatt ttatgctata aagttattta

39721 aatttattta ttctattaat agttttaaaa atattctcaa tctaaaaagt aaaagttatg

39781 tattaaattc atctatatct

//

LOCUS Fragosphaeria 57056 bp DNA linear 17-JAN-2019

DEFINITION .

ACCESSION

VERSION

KEYWORDS .

SOURCE Unknown.

ORGANISM Unknown.

Unclassified.

REFERENCE 1 (bases 1 to 57056)

AUTHORS Wai, Zubaer, Hausner

TITLE Direct Submission

JOURNAL Submitted (17-JAN-2019) Department of Microbiology, University of

Manitoba, 66 Chancellors Circle, Winnipeg R3T 2N2, Canada

FEATURES Location/Qualifiers

source 1..57056

/organism="Fragosphaeria purpurea"

/mol_type="genomic DNA"

gene 1..5189

/gene="cox1"

/note="copy 1"

CDS join(1..281,1532..1981,3017..3342,4639..5189)

/gene="cox1"

/EC_number="1.9.3.1"

/codon_start=1

/transl_table=4

/product="cytochrome c oxidase subunit 1"

/translation="MPIERWFLSTNAKDIGTLYLIFALFSGLLGTAFSVLIRMELSGP

GVQYIADNQLYNSIITAHAILMIFFMVMPALIGGFGNFLLPLLVGGPDMAFPRLNNIS

FWLLPPSLLLLIFSACIEGGAGTGWTIYPPLSGIQSHSGPSVDLAIFAIHLTGLSSLL

GAINFITTVVNMRTPGIRLHKLSLFGWAVVITAVLLLCSLPVLAGGITMVLTDRNFNT

SFFETAGGGDPILFQHLFWFFGHPEVYILVIPGFGIISTTISANSNKSVFGYIGMVYA

MMSIGILGFIVWSHHMYTVGLDVDTRAYFTAATLIIAVPTGIKIFSWLATCYGGSIKL

TPSMLFALGFVFMFTIGGLSGVLLANAALDTAFHDTYYVVAHFHYVLSMGAVFALFSG

WYFWVPKILGLSYNMLLSKVHFWILFIGVNLTFFPQHFLGLQGMPRRISDYPDAFAGW

NLVSSFGSIVSVVASGLFLYIVYKQLLDNNPVGRFPWLIPQYFTDTLQALLTRNYPSL

EWALTSPPKPHAFVSLPLQSNIINLYL"

exon 1..281

/gene="cox1"

/number=1

intron 282..1531

/gene="cox1"

/note="Group IB"

/number=1

gene 283..1176

/gene="orf297_1"

/note="copy 1"

CDS 283..1176

/gene="orf297_1"

/codon_start=1

/transl_except=(pos:283..285,aa:Lys)

/transl_table=4

/product="hypothetical protein"

/translation="KLKVLLGFNKRYFSKNLNNNNSNFNSYLAGLFEGDGHIWIQKTI

GSKTHNPRFCITFNMKNEPLAKKLLDILGSGFIRYKLQDNACVLVISPVTGLKRVVDL

LNGELRTPKIHQLHSLIDWLNKNHNTNYSKLDIKDSPLSNDAWLSGFVDSDGSFSVLY

SKTENGAKKRKITCRLRIEQRILDPVSKCSYEQILSKITNFLICSLLTKTQKSTGNKY

YTLTASSKKSLIVIINYFERYPLFSSKYLDYKDWKEIVQLILENKHYTEEGLMKTDLV

KKSMNRQRTYFNWDHITNLNI"

exon 1532..1981

/gene="cox1"

/number=2

intron 1982..3016

/gene="cox1"

/note="Group IB"

/number=2

gene 1983..2933

/gene="orf316_1"

/note="copy 1"

CDS 1983..2933

/gene="orf316_1"

/codon_start=1

/transl_except=(pos:1983..1985,aa:Asn)

/transl_table=4

/product="hypothetical protein"

/translation="NFISLLMSLYAGKASLFSFKYSLFIDIVKKLKQWSQSAGNIFKF

KNGTSETIRDNTEKIKNISIHVPEHLKPLNDQQLGHYLAGLIDGDGHFSSKQQLVIVF

SSPDIKLAYYIKEIIGFGHVNKVKDKNAYLYIISNKEGIIKTINLINGKLRTINKFNQ

VINNILSNPKYSEEKLEFKINDSNDFNNHWIAGFSDADASFQIKIINRDNKLKSEIRL

NFQIDQKDNNLLLSIKDVFGGNIGYRKNQDTYYYGSTSFGSAKKVINYFDRFNLQSSK

HINYLKWRKTYIIVQNKDHLTETGIEKIKNLKSSMNRYSV"

exon 3017..3342

/gene="cox1"

/number=3

intron 3343..4638

/gene="cox1"

/note="Group IB"

/number=3

gene 3345..4625

/gene="orf426_1"

/note="copy 1"

CDS 3345..4625

/gene="orf426_1"

/codon_start=1

/transl_except=(pos:3345..3347,aa:Leu)

/transl_table=4

/product="hypothetical protein"

/translation="LNHLALPLKITICWELLIIILLEIFLFSVTMYNFEQSAGNQRIN

LNILVGTSETTRDPHKSFMRRYSPWNKNSLINLYNYNYNLKYNKNNVRLYSSFIPKNI

KEDNIKNLEYIKRYENFEEDRSRILKEEKVESGVYCLINNTNGHSYVGSSNNLASRMK

NYLNNSFLKSRQNINMPIVKALLKYGQSNFTLLILEYIETESLTIRETFYITHIMPHY

NVLKQGYSSLGYKHTEETKNLLSELATNRIHSESTKSLISRALIGENNPFYNKNHSTE

TKVRMIEANSNYPVYVYDSLKNLLVIFPSVFTLSKLIHSDHPTLVKVIKEQTIFRGEW

YLSNIPYNLKDTPIISSWSSEESKRLVSNINDQVHIKKAIFVYDEHKNLLSKYEGVTK

AQAALGINHVTIKKHAKTQAIYNGYIFSYERLTN"

exon 4639..5189

/gene="cox1"

/number=4

gene 5373..6443

/gene="nad1"

/note="copy 1"

CDS 5373..6443

/gene="nad1"

/EC_number="1.6.5.3"

/codon_start=1

/transl_table=4

/product="NADH dehydrogenase subunit 1"

/translation="MLYFPTLMSIIEVILVLVPALLAVAFVTLAERKTMASMQRRLGP

NAVGYYGLLQAFADALKLLLKEYVAPTQANIILFFLGPVITLVFSLLGYGVIPYGPGL

AISDFSLGILYLLAVSSLATYGILLAGWSANSKYAFLGSLRSTAQLISYELVLSSAIL

LVIFLTGSLNLTVNMESQRAIWFILPLLPIFIIFFIGSVAETNRAPFDLAEAESELVS

GFMTEHAAVVFVFFFLAEYASIVLMCILTSILFIGGYLTPSNFLIDMLLHVYGVDVLI

DTIRNSPILEGLIYGLNLGIKSSIFIFVFIWVRASFPRIRFDQLMAFCWTVLLPIIFA

VIILIPAILYNFTLFPINISLL"

gene 6554..8017

/gene="nad4"

/note="copy 1"

CDS 6554..8017

/gene="nad4"

/EC_number="1.6.5.3"

/codon_start=1

/transl_table=4

/product="NADH dehydrogenase subunit 4"

/translation="MLLSFLLLVPLIGIFFIAGTISYEDNAIKITYYKNIALITSIIN

LIISLFVYILFDSNTNQFQFVQEHYNLSFFDIYLGVDGISIYFVLLTTIIMPIAILSN

WNSITENIKSYLILMLLLETLLLAIFLVLDILLFYIFFESTLPPLFLLIGLFGSNNKV

RASFYIFLYTLWGSLFLLLCILTMSSIMGTTDFDALFKTNFDYITQLFLFGGVFLSFA

VKTPTIFLNNWLLKAHVESPLGGSIVLAAIVLKTSLYGICRLILPILPKASINFTYIV

YVIGVITIIYASFSTLRTTDIKELIAYSSVSHAAVYLIGVFSNTIQGIEGSILLGLAH

GFVSSGLFICAGGILYDRSGTRSIYFYKGIAQIMPLFSVLFFILSLGNCGVPLTLNFV

GEFMSLYGVFERLPLLGVFASSSIILSAAYTIYMFNRIGFGGIFSKFFLENIADLTKR

EFFLLFILVLFTIIFGIYPSFILDGLHYSVTGLIYYS"

gene 8083..8241

/gene="atp8"

/note="copy 1"

CDS 8083..8241

/gene="atp8"

/EC_number="3.6.3.14"

/codon_start=1

/transl_table=4

/product="ATP synthase F0 subunit 8"

/translation="MPQLVPFYFLNEVIFTFAILTIVVYISSKYILPRFIRLFLSRTF

ISKLFDNK"

gene 8316..9116

/gene="atp6"

/note="copy 1"

CDS 8316..9116

/gene="atp6"

/EC_number="3.6.3.14"

/codon_start=1

/transl_table=4

/product="ATP synthase F0 subunit a"

/translation="MITLSVNNLDTLNLEILSPLTQFEIRDLLSIDAPLLGNLHLSIT

NIGFYLTIGAFIILTLSLLSTNYNKLISNNWSISQESLYATIHSIVTNQINPKNGQIY

FPFIYTLFIFILINNLIGMIPYSFASTSHFVLTFSLSFTIVLGATFLGFQKHGLEFFS

LLVPAGCPLPLLPLLVLIELISYLARNISLGLRLAANIMSGHMLLHILAGFTYNIMSS

GIIFFLLGLLPLSFIIAFAGLEIGIAFIQAQVFVVLTSSYIKDGLDLH"

gene 9146..9318

/gene="rnpB"

/note="copy 1"

gene 10925..11009

/gene="trnY(gta)"

/note="copy 1"

tRNA 10925..11009

/gene="trnY(gta)"

/product="tRNA-Tyr"

/anticodon=(pos:10959..10961,aa:Tyr,seq:gta)

gene 11934..12004

/gene="trnT(tgt)"

/note="copy 1"

tRNA 11934..12004

/gene="trnT(tgt)"

/product="tRNA-Thr"

/anticodon=(pos:11966..11968,aa:Thr,seq:tgt)

gene 12007..12079

/gene="trnE(ttc)"

/note="copy 1"

tRNA 12007..12079

/gene="trnE(ttc)"

/product="tRNA-Glu"

/anticodon=(pos:12040..12042,aa:Glu,seq:ttc)

gene 12114..12186

/gene="trnM(cat)"

/note="copy 1"

tRNA 12114..12186

/gene="trnM(cat)"

/product="tRNA-Met"

/anticodon=(pos:12147..12149,aa:Met,seq:cat)

gene 12190..12260

/gene="trnM(cat)"

/note="copy 2"

tRNA 12190..12260

/gene="trnM(cat)"

/product="tRNA-Met"

/anticodon=(pos:12222..12224,aa:Met,seq:cat)

gene 12278..12359

/gene="trnL(taa)"

/note="copy 1"

tRNA 12278..12359

/gene="trnL(taa)"

/product="tRNA-Leu"

/anticodon=(pos:12312..12314,aa:Leu,seq:taa)

gene 12837..12907

/gene="trnG(tcc)"

/note="copy 1"

tRNA 12837..12907

/gene="trnG(tcc)"

/product="tRNA-Gly"

/anticodon=(pos:12869..12871,aa:Gly,seq:tcc)

gene 12913..12983

/gene="trnA(tgc)"

/note="copy 1"

tRNA 12913..12983

/gene="trnA(tgc)"

/product="tRNA-Ala"

/anticodon=(pos:12945..12947,aa:Ala,seq:tgc)

gene 13169..13241

/gene="trnF(gaa)"

/note="copy 1"

tRNA 13169..13241

/gene="trnF(gaa)"

/product="tRNA-Phe"

/anticodon=(pos:13202..13204,aa:Phe,seq:gaa)

gene 13276..13357

/gene="trnL(tag)"

/note="copy 1"

tRNA 13276..13357

/gene="trnL(tag)"

/product="tRNA-Leu"

/anticodon=(pos:13310..13312,aa:Leu,seq:tag)

gene 13627..13699

/gene="trnQ(ttg)"

/note="copy 1"

tRNA 13627..13699

/gene="trnQ(ttg)"

/product="tRNA-Gln"

/anticodon=(pos:13660..13662,aa:Gln,seq:ttg)

gene 13787..13860

/gene="trnH(gtg)"

/note="copy 1"

tRNA 13787..13860

/gene="trnH(gtg)"

/product="tRNA-His"

/anticodon=(pos:13821..13823,aa:His,seq:gtg)

gene 13868..13938

/gene="trnM(cat)"

/note="copy 3"

tRNA 13868..13938

/gene="trnM(cat)"

/product="tRNA-Met"

/anticodon=(pos:13900..13902,aa:Met,seq:cat)

gene 14115..14187

/gene="trnI(tat)"

/note="copy 1"

tRNA 14115..14187

/gene="trnI(tat)"

/product="tRNA-Ile"

/anticodon=(pos:14149..14151,aa:Ile,seq:tat)

gene 14244..15686

/gene="nad2"

/note="copy 1"

CDS 14244..15686

/gene="nad2"

/EC_number="1.6.5.3"

/codon_start=1

/transl_table=4

/product="NADH dehydrogenase subunit 2"

/translation="MLLISILSLLLSNAVTIRRDISILFNRVAIIALVYSILHSTISL

FIIGKGIGLHGGLLNITSLTQIFDIFIFLISILILQLTSFFPRKVWIPEHSSLTQLLL

NNFIFYRTKIINKMGEHLRIIEYPLILLFIICGAVFLISTGDLISVFLSIELQSYGLY

LLSTIYRNSELSTTGGLMYFLLGGLSSCFILLGTSLLYANSGTTNLDGLYAITSISDS

TDLWYKPYYINLSLLVFSIGFLFKVSAAPFHFWSPDVYDAIPTIVTTFVAIIAKISIL

VFFLEIVYYTKNYFIDFNWTYGLLISSLFSLIIGTVAGLTQFRIKKLFAYSTISHIGF

ILLALSISSVESTQAFIFYLMQYSISNLNAFLILIAIGFSLYYYISDNKEYKELLDKD

NSPIQLISQLKGYFYINPTLALSLVITIFSFAGIPPLVGFFAKQMVLSAALDNGYIFL

SLIAILTSVIGAVYYLNIIKEIFFIHHNIK"

gene 15905..16306

/gene="nad3"

/note="copy 1"

CDS 15905..16306

/gene="nad3"

/EC_number="1.6.5.3"

/codon_start=1

/transl_table=4

/product="NADH dehydrogenase subunit 3"

/translation="MSSMSIFFIFVLVIAVLFLVINLVFAPHNPYQEKYSIFECGFHS

FLGQNRSQFTIKFFIYALIFLLLDLEILLIFPFGVSGYTNDLYGLIIVLGFITIVTIG

FVYELGKNALKIDSRQSIIKNKSNNIKIFTL"

gene 16422..16646

/gene="atp9"

/note="copy 1"

CDS 16422..16646

/gene="atp9"

/EC_number="3.6.3.14"

/codon_start=1

/transl_table=4

/product="ATP synthase F0 subunit c"

/translation="MVLVAKIIGTGLATTGLIGAGVGIGIVFGALILGVARNPSLRGQ

LFSYAILGFAFSEATGLFALMMAFLLLYVV"

gene 16672..17472

/gene="orf266"

/note="copy 1"

CDS 16672..17472

/gene="orf266"

/codon_start=1

/transl_table=4

/product="hypothetical protein"

/translation="MILTKLNSNQINYTNRSNFSTSTRLYKIDATGLTFSQKSILASN

ISTGPDIPFRLNDLYSLGRLLGQEVPITQNDFVGTPDLRSTLTISATSNEAINMTSRV

THLNNYELLAIITALAIVGFGVIAITARLRRLTRIVENAVTEETPAWDIRNQTNGLER

DILDEFSLLTDRMDEAALSLARYIQVQEHSNLDMIPRSTIATINELQPQWNEMLRALG

ENLASNNTITFLGRENQLYLYQNWMQILTESMQEFLVQYEIFLDFLFF"

gene 17612..18364

/gene="cox2"

/note="copy 1"

CDS 17612..18364

/gene="cox2"

/EC_number="1.9.3.1"

/codon_start=1

/transl_table=4

/product="cytochrome c oxidase subunit 2"

/translation="MNLILNNLIMKLDAPEAWGIYFQDSATPQMEGLVELHDNIMYYL

VIILFAVGWILLSVIRNFVMSTSPISHKYLNHGTLIELIWTITPAVILILIAFPSFKL

LYLMDEVSDPSMSILAEGHQWYWSYQYPDFLDSSEEFIEFDSYIVPDSDLEEGGLRML

EVDNRVIIPELTHIRFIITSGDVIHSFSCNSLGIKCDAYPGRLNQVSVFVNREGVFYG

QCSEICGILHSSMPIVIESVSLEKFLTWLEEQ"

gene 18417..18489

/gene="trnR(acg)"

/note="copy 1"

tRNA 18417..18489

/gene="trnR(acg)"

/product="tRNA-Arg"

/anticodon=(pos:18450..18452,aa:Arg,seq:acg)

CDS 18550..18819

/gene="nad4L"

gene 18819..20810

/gene="nad5"

/note="copy 1"

CDS 18819..20810

/gene="nad5"

/EC_number="1.6.5.3"

/codon_start=1

/transl_table=4

/product="NADH dehydrogenase subunit 5"

/translation="MYLSIIILPLLGSIVSGFFGRKVGVSGAQIITCSSIIVTTILAI

ITFFEVGFNNIVLSINLFRWIDSEWFNIIWGFQFDSLTVSMLIPVLIISSLVHVYSIG

YMNGDPHNQRFFSYLSLFTFMMIILVTANNYLLMFVGWEGVGVCSYLLVSFWFTRIAA

NQSSMSAFLTNRVGDCFLTIGMFAILWSLGNLDYSTVFSLAPYINENIIIIIGVCLLI

GAMAKSSQVGLHVWLPMAMEGPTPVSALIHAATMVTAGVYLLMRSSPLIEYSSTVLLL

CLWLGAITTVFSSLVGFFQQDIKKVIAYSTMSQLGMMVIAVGLSSYNVALFHLVNHAF

YKGLLFLGAGAVIHAMADNQDFRKYGGLISFLPLSYSVILIASLSLVAFPFMTGFYSK

DFILESAYGQYCFSSITVYIVAVIGAIFTTLYSVKVLYLTFLTNPNGPLINYKHAHES

DIFMSLPLVILAIFSIFFGFITKDIFIGLGSGFFIDNSIFIHPNSEIMIDTEFGVSTY

WKLLPFIFTVLFSIIAIILSEFFSDLIVNFKLSTLGRTIFGFFNQRFLIEFFYNKYIT

NLVLNLGSQTVKVLDKGSIELLGPYGLEKNLITSSKNISSLNKGIVTNYALFILVGFI

LYMFSISLGFTNNLSLIILILMFSISLISKKKSKFIINK"

gene 20913..23354

/gene="cob"

/note="copy 1"

CDS join(20913..21305,22554..23354)

/gene="cob"

/EC_number="1.10.2.2"

/codon_start=1

/transl_table=4

/product="apocytochrome b"

/translation="MRIIKNNTILKLVNAYLIDASQPSNISYLWNFGSLLAICLGIQI

ITGVTLAMHYSPSILEAFNSVEHIMRDVNNGWLIRYLHSNTASAFFFLVYLHIARGMY

YGSYRSPRTLVWTIGVIILILMIAIGFLGYVLPYGQMSLWGATVITNLISAIPWIGQD

IVEFIWGGFSVNNATLNRFFALHFVLPFVLAALVLMHLIALHDTAGSGNPLGVAGTYD

RVPFAPYFLFKDLITIFIFIFVLSFFVFFMPNILGDSENYIMANPMQTPPAIVPEWYL

LPFYAILRSIPNKLLGVIAMFGALLILLILPITDLGRSRGFQFRPLSKFVLYLFGVDF

LLLARLGAVHVEDPFIQIGQICACIYFVFFIFSLPVASVIENSFMDIAYVTEENKISI

KNKLK"

exon 20913..21305

/gene="cob"

/number=1

gene 21306..22196

/gene="orf296"

/note="copy 1"

CDS 21306..22196

/gene="orf296"

/codon_start=1

/transl_except=(pos:21306..21308,aa:Tyr)

/transl_table=4

/product="hypothetical protein"

/translation="YELSPKWFNKELKYHNNNNNNKLISFNKRYYSTTSNINNSTNSK

RLDEILEELKLDYIYSFEDLNLETTKKEILDKTKGLSGIYMIINKITKDYYIGSASTN

RFYARFSNHLIYFRGSKVVKAAVNKYGLENFTFIILELYPNIINKENNKELLDLEDKY

LKLLLPNYNILTEAGSSFGYKHTEIDRQKMKDMYTDERRELIRNLNKGKKLSPETIEK

IREKALKIPIRSIDTRSNYITHTRPITLYNLDGTIYGSYPTIIEAAKSINCGEKTLIR

ALKTEKGLVKRQWIIKDTLK"

intron 21306..22553

/gene="cob"

/note="Group ID"

/number=1

exon 22554..23354

/gene="cob"

/number=2

gene 24062..24132

/gene="trnC(gca)"

/note="copy 1"

tRNA 24062..24132

/gene="trnC(gca)"

/product="tRNA-Cys"

/anticodon=(pos:24094..24096,aa:Cys,seq:gca)

gene 24168..24238

/gene="trnR(tct)"

/note="copy 1"

tRNA 24168..24238

/gene="trnR(tct)"

/product="tRNA-Arg"

/anticodon=(pos:24200..24202,aa:Arg,seq:tct)

gene 24373..29561

/gene="cox1"

/note="copy 2"

CDS join(24373..24653,25904..26353,27389..27714,29011..29561)

/gene="cox1"

/EC_number="1.9.3.1"

/codon_start=1

/transl_table=4

/product="cytochrome c oxidase subunit 1"

/translation="MPIERWFLSTNAKDIGTLYLIFALFSGLLGTAFSVLIRMELSGP

GVQYIADNQLYNSIITAHAILMIFFMVMPALIGGFGNFLLPLLVGGPDMAFPRLNNIS

FWLLPPSLLLLIFSACIEGGAGTGWTIYPPLSGIQSHSGPSVDLAIFAIHLTGLSSLL

GAINFITTVVNMRTPGIRLHKLSLFGWAVVITAVLLLCSLPVLAGGITMVLTDRNFNT

SFFETAGGGDPILFQHLFWFFGHPEVYILVIPGFGIISTTISANSNKSVFGYIGMVYA

MMSIGILGFIVWSHHMYTVGLDVDTRAYFTAATLIIAVPTGIKIFSWLATCYGGSIKL

TPSMLFALGFVFMFTIGGLSGVLLANAALDTAFHDTYYVVAHFHYVLSMGAVFALFSG

WYFWVPKILGLSYNMLLSKVHFWILFIGVNLTFFPQHFLGLQGMPRRISDYPDAFAGW

NLVSSFGSIVSVVASGLFLYIVYKQLLDNNPVGRFPWLIPQYFTDTLQALLTRNYPSL

EWALTSPPKPHAFVSLPLQSNIINLYL"

exon 24373..24653

/gene="cox1"

/number=1

intron 24654..25903

/gene="cox1"

/note="Group IB"

/number=1

gene 24655..25548

/gene="orf297_2"

/note="copy 2"

CDS 24655..25548

/gene="orf297_2"

/codon_start=1

/transl_except=(pos:24655..24657,aa:Lys)

/transl_table=4

/product="hypothetical protein"

/translation="KLKVLLGFNKRYFSKNLNNNNSNFNSYLAGLFEGDGHIWIQKTI

GSKTHNPRFCITFNMKNEPLAKKLLDILGSGFIRYKLQDNACVLVISPVTGLKRVVDL

LNGELRTPKIHQLHSLIDWLNKNHNTNYSKLDIKDSPLSNDAWLSGFVDSDGSFSVLY

SKTENGAKKRKITCRLRIEQRILDPVSKCSYEQILSKITNFLICSLLTKTQKSTGNKY

YTLTASSKKSLIVIINYFERYPLFSSKYLDYKDWKEIVQLILENKHYTEEGLMKTDLV

KKSMNRQRTYFNWDHITNLNI"

exon 25904..26353

/gene="cox1"

/number=2

intron 26354..27388

/gene="cox1"

/note="Group IB"

/number=2

gene 26355..27305

/gene="orf316_2"

/note="copy 2"

CDS 26355..27305

/gene="orf316_2"

/codon_start=1

/transl_except=(pos:26355..26357,aa:Asn)

/transl_table=4

/product="hypothetical protein"

/translation="NFISLLMSLYAGKASLFSFKYSLFIDIVKKLKQWSQSAGNIFKF

KNGTSETIRDNTEKIKNISIHVPEHLKPLNDQQLGHYLAGLIDGDGHFSSKQQLVIVF

SSPDIKLAYYIKEIIGFGHVNKVKDKNAYLYIISNKEGIIKTINLINGKLRTINKFNQ

VINNILSNPKYSEEKLEFKINDSNDFNNHWIAGFSDADASFQIKIINRDNKLKSEIRL

NFQIDQKDNNLLLSIKDVFGGNIGYRKNQDTYYYGSTSFGSAKKVINYFDRFNLQSSK

HINYLKWRKTYIIVQNKDHLTETGIEKIKNLKSSMNRYSV"

exon 27389..27714

/gene="cox1"

/number=3

intron 27715..29010

/gene="cox1"

/note="Group IB"

/number=3

gene 27717..28997

/gene="orf426_2"

/note="copy 2"

CDS 27717..28997

/gene="orf426_2"

/codon_start=1

/transl_except=(pos:27717..27719,aa:Leu)

/transl_table=4

/product="hypothetical protein"

/translation="LNHLALPLKITICWELLIIILLEIFLFSVTMYNFEQSAGNQRIN

LNILVGTSETTRDPHKSFMRRYSPWNKNSLINLYNYNYNLKYNKNNVRLYSSFIPKNI

KEDNIKNLEYIKRYENFEEDRSRILKEEKVESGVYCLINNTNGHSYVGSSNNLASRMK

NYLNNSFLKSRQNINMPIVKALLKYGQSNFTLLILEYIETESLTIRETFYITHIMPHY

NVLKQGYSSLGYKHTEETKNLLSELATNRIHSESTKSLISRALIGENNPFYNKNHSTE

TKVRMIEANSNYPVYVYDSLKNLLVIFPSVFTLSKLIHSDHPTLVKVIKEQTIFRGEW

YLSNIPYNLKDTPIISSWSSEESKRLVSNINDQVHIKKAIFVYDEHKNLLSKYEGVTK

AQAALGINHVTIKKHAKTQAIYNGYIFSYERLTN"

exon 29011..29561

/gene="cox1"

/number=4

gene 29745..30815

/gene="nad1"

/note="copy 2"

CDS 29745..30815

/gene="nad1"

/EC_number="1.6.5.3"

/codon_start=1

/transl_table=4

/product="NADH dehydrogenase subunit 1"

/translation="MLYFPTLMSIIEVILVLVPALLAVAFVTLAERKTMASMQRRLGP

NAVGYYGLLQAFADALKLLLKEYVAPTQANIILFFLGPVITLVFSLLGYGVIPYGPGL

AISDFSLGILYLLAVSSLATYGILLAGWSANSKYAFLGSLRSTAQLISYELVLSSAIL

LVIFLTGSLNLTVNMESQRAIWFILPLLPIFIIFFIGSVAETNRAPFDLAEAESELVS

GFMTEHAAVVFVFFFLAEYASIVLMCILTSILFIGGYLTPSNFLIDMLLHVYGVDVLI

DTIRNSPILEGLIYGLNLGIKSSIFIFVFIWVRASFPRIRFDQLMAFCWTVLLPIIFA

VIILIPAILYNFTLFPINISLL"

gene 30926..32389

/gene="nad4"

/note="copy 2"

CDS 30926..32389

/gene="nad4"

/EC_number="1.6.5.3"

/codon_start=1

/transl_table=4

/product="NADH dehydrogenase subunit 4"

/translation="MLLSFLLLVPLIGIFFIAGTISYEDNAIKITYYKNIALITSIIN

LIISLFVYILFDSNTNQFQFVQEHYNLSFFDIYLGVDGISIYFVLLTTIIMPIAILSN

WNSITENIKSYLILMLLLETLLLAIFLVLDILLFYIFFESTLPPLFLLIGLFGSNNKV

RASFYIFLYTLWGSLFLLLCILTMSSIMGTTDFDALFKTNFDYITQLFLFGGVFLSFA

VKTPTIFLNNWLLKAHVESPLGGSIVLAAIVLKTSLYGICRLILPILPKASINFTYIV

YVIGVITIIYASFSTLRTTDIKELIAYSSVSHAAVYLIGVFSNTIQGIEGSILLGLAH

GFVSSGLFICAGGILYDRSGTRSIYFYKGIAQIMPLFSVLFFILSLGNCGVPLTLNFV

GEFMSLYGVFERLPLLGVFASSSIILSAAYTIYMFNRIGFGGIFSKFFLENIADLTKR

EFFLLFILVLFTIIFGIYPSFILDGLHYSVTGLIYYS"

gene 32455..32613

/gene="atp8"

/note="copy 2"

CDS 32455..32613

/gene="atp8"

/EC_number="3.6.3.14"

/codon_start=1

/transl_table=4

/product="ATP synthase F0 subunit 8"

/translation="MPQLVPFYFLNEVIFTFAILTIVVYISSKYILPRFIRLFLSRTF

ISKLFDNK"

gene 32688..33488

/gene="atp6"

/note="copy 2"

CDS 32688..33488

/gene="atp6"

/EC_number="3.6.3.14"

/codon_start=1

/transl_table=4

/product="ATP synthase F0 subunit a"

/translation="MITLSVNNLDTLNLEILSPLTQFEIRDLLSIDAPLLGNLHLSIT

NIGFYLTIGAFIILTLSLLSTNYNKLISNNWSISQESLYATIHSIVTNQINPKNGQIY

FPFIYTLFIFILINNLIGMIPYSFASTSHFVLTFSLSFTIVLGATFLGFQKHGLEFFS

LLVPAGCPLPLLPLLVLIELISYLARNISLGLRLAANIMSGHMLLHILAGFTYNIMSS

GIIFFLLGLLPLSFIIAFAGLEIGIAFIQAQVFVVLTSSYIKDGLDLH"

gene 33518..33690

/gene="rnpB"

/note="copy 2"

gene 33761..35258

/gene="rns"

rRNA 33761..35258

/gene="rns"

/product="small subunit ribosomal RNA"

gene 35296..35381

/gene="trnY(gta)"

/note="copy 2"

tRNA 35296..35381

/gene="trnY(gta)"

/product="tRNA-Tyr"

/anticodon=(pos:35331..35333,aa:Tyr,seq:gta)

gene 35414..35484

/gene="trnN(gtt)"

tRNA 35414..35484

/gene="trnN(gtt)"

/product="tRNA-Asn"

/anticodon=(pos:35446..35448,aa:Asn,seq:gtt)

gene 35557..36366

/gene="cox3"

CDS 35557..36366

/gene="cox3"

/EC_number="1.9.3.1"

/codon_start=1

/transl_table=4

/product="cytochrome c oxidase subunit 3"

/translation="MINSVRSNFQDHPFHLVSPSPWPLYTSLSLYSLTTSTALSMHNF

NNAYFFVYLSVFLLMSSMFFWFRDIISESTFLGDHSLAVQKGLNLGVILFIASEALFF

LAVFWAFFHSALTPTVELGAQWPPLGIEPVNPFELPLLNTVILLSSGATVTFGHHSLI

QGNRSGAVYGTMITGLLAIIFTVFQGIEYSVSSFTISDGAFGTCFFFGTGFHGLHVMI

GTIFLLVALWRIYAYHLTDNHHLGFESGILYWHFVDLVWLFLYLFVYYWGS"

gene 36324..37388

/gene="orf354"

CDS 36324..37388

/gene="orf354"

/codon_start=1

/transl_table=4

/product="hypothetical protein"

/translation="MAFFVSFCVLLGILDIFISISNTSIQTIQTFIIIERFPLLWSIF

IPIIFILYFYFFSEQIWKNRKIMLIKIFKRIAFFYSLVFFIAFSVFLLYNIILNYTDF

YLFNSLTLLDFISYTRTSLMIFYIFKYIKTIIYQGFNKDNFLIFIFGLSIINLITILI

KIFITTFIPEIYIVLSLDGTPNTGNSSGPQGSQPPQWPQGPQGPQPPQGPEPPQGPNH

AQPSHMSEVTEEQQEELEEIKDFEEEYEESGNTAAQLTAESDNAAEDVIRITENLYSK

EAELQEKQARGENTEQTLNEIRHLNESLNEANSKEETLFVEAERASRYHERVGTLYKN

KYDAYCNKYGNNPNNNNNNN"

gene 37431..37502

/gene="trnK(ttt)"

tRNA 37431..37502

/gene="trnK(ttt)"

/product="tRNA-Lys"

/anticodon=(pos:37463..37465,aa:Lys,seq:ttt)

gene 38086..38158

/gene="trnD(gtc)"

tRNA 38086..38158

/gene="trnD(gtc)"

/product="tRNA-Asp"

/anticodon=(pos:38119..38121,aa:Asp,seq:gtc)

gene 38165..38245

/gene="trnS(gct)"

tRNA 38165..38245

/gene="trnS(gct)"

/product="tRNA-Ser"

/anticodon=(pos:38197..38199,aa:Ser,seq:gct)

gene 38277..38347

/gene="trnW(tca)"

tRNA 38277..38347

/gene="trnW(tca)"

/product="tRNA-Trp"

/anticodon=(pos:38309..38311,aa:Trp,seq:tca)

gene 38430..39086

/gene="nad6"

CDS 38430..39086

/gene="nad6"

/EC_number="1.6.5.3"

/codon_start=1

/transl_table=4

/product="NADH dehydrogenase subunit 6"

/translation="MNNLFIINESFTNGYKNEVLDIISILVILSGIFVIISKNPIISL

LFLIGLFAGISSYLLIIGLSFLGLSYLVVYIGAVSILFLFILMLINIRISELQSDTNN

SIPLAISIAILFNYSLFQILPYDIAILNNNNYLNNILYNVSFNKLDNGLETSLNINNN

DTLFVTSKMWDGNLAEVGHITSIGNIMYTNYNLWLIITSFILLLAMVGSIVITMKSSK

"

gene 39155..39227

/gene="trnV(tac)"

tRNA 39155..39227

/gene="trnV(tac)"

/product="tRNA-Val"

/anticodon=(pos:39188..39190,aa:Val,seq:tac)

gene 39289..39359

/gene="trnI(gat)"

tRNA 39289..39359

/gene="trnI(gat)"

/product="tRNA-Ile"

/anticodon=(pos:39321..39323,aa:Ile,seq:gat)

gene 39360..39443

/gene="trnS(aga)"

tRNA 39360..39443

/gene="trnS(aga)"

/product="tRNA-Ser"

/anticodon=(pos:39393..39395,aa:Ser,seq:aga)

gene 39451..39536

/gene="trnS(tga)"

tRNA 39451..39536

/gene="trnS(tga)"

/product="tRNA-Ser"

/anticodon=(pos:39484..39486,aa:Ser,seq:tga)

gene 39652..39724

/gene="trnP(tgg)"

tRNA 39652..39724

/gene="trnP(tgg)"

/product="tRNA-Pro"

/anticodon=(pos:39685..39687,aa:Pro,seq:tgg)

gene 43238..43963

/gene="orf241"

CDS 43238..43963

/gene="orf241"

/codon_start=1

/transl_table=4

/product="hypothetical protein"

/translation="MDLYDKYFKNIFYKELISIKRSKLKLDINEFKFKDIFLYKLSKL

IGKFYNKKVEFNIINLKSIAYNSNIFTEILKRKISDRKDLIFRTMDIILSKGIIFEEK

NINKEKKLVKNINFNLIENKYKNLNINYIVKNINLNKTIKDLYDIEDYDNKDIIFDSI

KYKNLGGMKLEIKGRLTRRNRADRAIFKLKLKGRLKNTDSSFKGLSSVYLRGNTSSHL

EYSMGVNKRKIGAFAVKGWITGK"

gene 44614..44684

/gene="trnT(tgt)"

/note="copy 2"

tRNA 44614..44684

/gene="trnT(tgt)"

/product="tRNA-Thr"

/anticodon=(pos:44646..44648,aa:Thr,seq:tgt)

gene 44687..44759

/gene="trnE(ttc)"

/note="copy 2"

tRNA 44687..44759

/gene="trnE(ttc)"

/product="tRNA-Glu"

/anticodon=(pos:44720..44722,aa:Glu,seq:ttc)

gene 44794..44866

/gene="trnM(cat)"

/note="copy 4"

tRNA 44794..44866

/gene="trnM(cat)"

/product="tRNA-Met"

/anticodon=(pos:44827..44829,aa:Met,seq:cat)

gene 44870..44940

/gene="trnM(cat)"

/note="copy 5"

tRNA 44870..44940

/gene="trnM(cat)"

/product="tRNA-Met"

/anticodon=(pos:44902..44904,aa:Met,seq:cat)

gene 44958..45039

/gene="trnL(taa)"

/note="copy 2"

tRNA 44958..45039

/gene="trnL(taa)"

/product="tRNA-Leu"

/anticodon=(pos:44992..44994,aa:Leu,seq:taa)

gene 45519..45589

/gene="trnG(tcc)"

/note="copy 2"

tRNA 45519..45589

/gene="trnG(tcc)"

/product="tRNA-Gly"

/anticodon=(pos:45551..45553,aa:Gly,seq:tcc)

gene 45595..45665

/gene="trnA(tgc)"

/note="copy 2"

tRNA 45595..45665

/gene="trnA(tgc)"

/product="tRNA-Ala"

/anticodon=(pos:45627..45629,aa:Ala,seq:tgc)

gene 45851..45923

/gene="trnF(gaa)"

/note="copy 2"

tRNA 45851..45923

/gene="trnF(gaa)"

/product="tRNA-Phe"

/anticodon=(pos:45884..45886,aa:Phe,seq:gaa)

gene 45958..46039

/gene="trnL(tag)"

/note="copy 2"

tRNA 45958..46039

/gene="trnL(tag)"

/product="tRNA-Leu"

/anticodon=(pos:45992..45994,aa:Leu,seq:tag)

gene 46310..46382

/gene="trnQ(ttg)"

/note="copy 2"

tRNA 46310..46382

/gene="trnQ(ttg)"

/product="tRNA-Gln"

/anticodon=(pos:46343..46345,aa:Gln,seq:ttg)

gene 46470..46543

/gene="trnH(gtg)"

/note="copy 2"

tRNA 46470..46543

/gene="trnH(gtg)"

/product="tRNA-His"

/anticodon=(pos:46504..46506,aa:His,seq:gtg)

gene 46551..46621

/gene="trnM(cat)"

/note="copy 6"

tRNA 46551..46621

/gene="trnM(cat)"

/product="tRNA-Met"

/anticodon=(pos:46583..46585,aa:Met,seq:cat)

gene 46798..46870

/gene="trnI(tat)"

/note="copy 2"

tRNA 46798..46870

/gene="trnI(tat)"

/product="tRNA-Ile"

/anticodon=(pos:46832..46834,aa:Ile,seq:tat)

gene 46927..48588

/gene="nad2"

/note="copy 2"

CDS 46927..48588

/gene="nad2"

/EC_number="1.6.5.3"

/codon_start=1

/transl_table=4

/product="NADH dehydrogenase subunit 2"

/translation="MLLISILSLLLSNAVTIRRDISILFNRVAIIALVYSILHSTISL

FIIGKGIGLHGGLLNITSLTQIFDIFIFLISILILQLTSFFPRKVWIPEHSSLTQLLL

NNFIFYRTKIINKMGEHLRIIEYPLILLFIICGAVFLISTGDLISVFLSIELQSYGLY

LLSTIYRNSELSTTGGLMYFLLGGLSSCFILLGTSLLYANSGTTNLDGLYAITSISDS

TDLWYKPYYINLSLLVFSIGFLFKVSAAPFHFWSPDVYDAIPTIVTTFVAIIAKISIL

VFFLEIVYYTKNYFIDFNWTYGLLISSLFSLIIGTVAGLTQFRIKKLFAYSTISHIGF

ILLALSISSVESTQAFIFYLMQYSISNLNAFLILIAIGFSLYYYISDNKEYKELLDKD

NSPIQLISQLKGYFYINPTLALSLVITIFSFAGIPPLVGFFAKQMVLSAALDNGYIFL

SLIAILTSVIGAVYYLNIIKEIFFYSPQYKINPLLEKLNFNGRIYNKQNILVKSITFK

YNNIAISSFLSITISIITLIILLFIFVNKEWLSMSTILVQLLFNY"

gene 48589..48990

/gene="nad3"

/note="copy 2"

CDS 48589..48990

/gene="nad3"

/EC_number="1.6.5.3"

/codon_start=1

/transl_table=4

/product="NADH dehydrogenase subunit 3"

/translation="MSSMSIFFIFVLVIAVLFLVINLVFAPHNPYQEKYSIFECGFHS

FLGQNRSQFTIKFFIYALIFLLLDLEILLIFPFGVSGYTNDLYGLIIVLGFITIVTIG

FVYELGKNALKIDSRQSIIKNKSNNIKIFTL"

gene 49106..49330

/gene="atp9"

/note="copy 2"

CDS 49106..49330

/gene="atp9"

/EC_number="3.6.3.14"

/codon_start=1

/transl_table=4

/product="ATP synthase F0 subunit c"

/translation="MVLVAKIIGTGLATTGLIGAGVGIGIVFGALILGVARNPSLRGQ

LFSYAILGFAFSEATGLFALMMAFLLLYVV"

gene 49356..50156

/gene="orf266"

/note="copy 2"

CDS 49356..50156

/gene="orf266"

/codon_start=1

/transl_table=4

/product="hypothetical protein"

/translation="MILTKLNSNQINYTNRSNFSTSTRLYKIDATGLTFSQKSILASN

ISTGPDIPFRLNDLYSLGRLLGQEVPITQNDFVGTPDLRSTLTISATSNEAINMTSRV

THLNNYELLAIITALAIVGFGVIAITARLRRLTRIVENAVTEETPAWDIRNQTNGLER

DILDEFSLLTDRMDEAALSLARYIQVQEHSNLDMIPRSTIATINELQPQWNEMLRALG

ENLASNNTITFLGRENQLYLYQNWMQILTESMQEFLVQYEIFLDFLFF"

gene 50296..51048

/gene="cox2"

/note="copy 2"

CDS 50296..51048

/gene="cox2"

/EC_number="1.9.3.1"

/codon_start=1

/transl_table=4

/product="cytochrome c oxidase subunit 2"

/translation="MNLILNNLIMKLDAPEAWGIYFQDSATPQMEGLVELHDNIMYYL

VIILFAVGWILLSVIRNFVMSTSPISHKYLNHGTLIELIWTITPAVILILIAFPSFKL

LYLMDEVSDPSMSILAEGHQWYWSYQYPDFLDSSEEFIEFDSYIVPDSDLEEGGLRML

EVDNRVIIPELTHIRFIITSGDVIHSFSCNSLGIKCDAYPGRLNQVSVFVNREGVFYG

QCSEICGILHSSMPIVIESVSLEKFLTWLEEQ"

gene 51101..51173

/gene="trnR(acg)"

/note="copy 2"

tRNA 51101..51173

/gene="trnR(acg)"

/product="tRNA-Arg"

/anticodon=(pos:51134..51136,aa:Arg,seq:acg)

gene 51503..53494

/gene="nad5"

/note="copy 2"

CDS 51503..53494

/gene="nad5"

/EC_number="1.6.5.3"

/codon_start=1

/transl_table=4

/product="NADH dehydrogenase subunit 5"

/translation="MYLSIIILPLLGSIVSGFFGRKVGVSGAQIITCSSIIVTTILAI

ITFFEVGFNNIVLSINLFRWIDSEWFNIIWGFQFDSLTVSMLIPVLIISSLVHVYSIG

YMNGDPHNQRFFSYLSLFTFMMIILVTANNYLLMFVGWEGVGVCSYLLVSFWFTRIAA

NQSSMSAFLTNRVGDCFLTIGMFAILWSLGNLDYSTVFSLAPYINENIIIIIGVCLLI

GAMAKSSQVGLHVWLPMAMEGPTPVSALIHAATMVTAGVYLLMRSSPLIEYSSTVLLL

CLWLGAITTVFSSLVGFFQQDIKKVIAYSTMSQLGMMVIAVGLSSYNVALFHLVNHAF

YKGLLFLGAGAVIHAMADNQDFRKYGGLISFLPLSYSVILIASLSLVAFPFMTGFYSK

DFILESAYGQYCFSSITVYIVAVIGAIFTTLYSVKVLYLTFLTNPNGPLINYKHAHES

DIFMSLPLVILAIFSIFFGFITKDIFIGLGSGFFIDNSIFIHPNSEIMIDTEFGVSTY

WKLLPFIFTVLFSIIAIILSEFFSDLIVNFKLSTLGRTIFGFFNQRFLIEFFYNKYIT

NLVLNLGSQTVKVLDKGSIELLGPYGLEKNLITSSKNISSLNKGIVTNYALFILVGFI

LYMFSISLGFTNNLSLIILILMFSISLISKKKSKFIINK"

gene 53597..56038

/gene="cob"

/note="copy 2"

CDS join(53597..54005,55254..56038)

/gene="cob"

/EC_number="1.10.2.2"

/codon_start=1

/transl_table=4

/product="apocytochrome b"

/translation="MRIIKNNTILKLVNAYLIDASQPSNISYLWNFGSLLAICLGIQI

ITGVTLAMHYSPSILEAFNSVEHIMRDVNNGWLIRYLHSNTASAFFFLVYLHIARGMY

YGSYRSPRTLVWTIGVIILILMIAIGFLGYELSPSQMSLWGATVITNLISAIPWIGQD

IVEFIWGGFSVNNATLNRFFALHFVLPFVLAALVLMHLIALHDTAGSGNPLGVAGTYD

RVPFAPYFLFKDLITIFIFIFVLSFFVFFMPNILGDSENYIMANPMQTPPAIVPEWYL

LPFYAILRSIPNKLLGVIAMFGALLILLILPITDLGRSRGFQFRPLSKFVLYLFGVDF

LLLARLGAVHVEDPFIQIGQICACIYFVFFIFSLPVASVIENSFMDIAYVTEENKISI

KNKLK"

exon 53597..54005

/gene="cob"

/number=1

intron 54006..55253

/gene="cob"

/note="Group ID"

/number=1

gene 54008..54880

/gene="orf290"

/note="copy 2"

CDS 54008..54880

/gene="orf290"

/codon_start=1

/transl_except=(pos:54008..54010,aa:Trp)

/transl_table=4

/product="hypothetical protein"

/translation="WFNKELKYHNNNNNNKLISFNKRYYSTTSNINNSTNSKRLDEIL

EELKLDYIYSFEDLNLETTKKEILDKTKGLSGIYMIINKITKDYYIGSASTNRFYARF

SNHLIYFRGSKVVKAAVNKYGLENFTFIILELYPNIINKENNKELLDLEDKYLKLLLP

NYNILTEAGSSFGYKHTEIDRQKMKDMYTDERRELIRNLNKGKKLSPETIEKIREKAL

KIPIRSIDTRSNYITHTRPITLYNLDGTIYGSYPTIIEAAKSINCGEKTLIRALKTEK

GLVKRQWIIKDTLK"

exon 55254..56038

/gene="cob"

/number=2

gene 56746..56816

/gene="trnC(gca)"

/note="copy 2"

tRNA 56746..56816

/gene="trnC(gca)"

/product="tRNA-Cys"

/anticodon=(pos:56778..56780,aa:Cys,seq:gca)

gene 56852..56922

/gene="trnR(tct)"

/note="copy 2"

tRNA 56852..56922

/gene="trnR(tct)"

/product="tRNA-Arg"

/anticodon=(pos:56884..56886,aa:Arg,seq:tct)

BASE COUNT 21051 a 6138 c 7946 g 21921 t

ORIGIN

1 atgccaatag aaagatgatt tttatctaca aatgctaaag acattggaac tctttatcta

61 atatttgcat tattttcagg actattaggt acagcatttt ctgttttaat aagaatggaa

121 cttagtggac caggtgttca atatatagct gataatcaat tatacaatag tataataaca

181 gctcacgcta ttttaatgat tttctttatg gttatgcctg cattaatagg tggttttggt

241 aatttcttat tacctttatt agtaggaggg ccagatatgg caaaattaaa agttctcctt

301 ggatttaata aaagatattt tagtaaaaat ttaaataata acaatagtaa ttttaatagt

361 tatttagctg gtttatttga aggagatgga catatttgaa ttcaaaaaac cattggatca

421 aaaacacata atcctagatt ctgtataact ttcaatatga aaaatgaacc tttagctaaa

481 aaattattgg atatattagg atctggtttt attagatata aattacaaga taatgcttgt

541 gtattagtta tttcaccagt aacaggttta aaaagagttg ttgatttatt aaatggtgaa

601 ttaagaactc caaaaataca ccaacttcat agtttaatag attgattaaa taaaaatcat

661 aatactaatt atagtaaatt agatataaaa gacagtccac tatctaatga tgcttgatta

721 agtggatttg tagattcaga tggtagtttt tctgttttat atagtaaaac agaaaatgga

781 gcaaaaaaaa gaaaaatcac ttgtagatta agaatcgaac aaagaatatt agatcctgta

841 tctaaatgta gctatgaaca aattttatca aaaataacaa attttctgat ttgttcatta

901 ctaactaaaa ctcaaaaatc tactggtaat aagtactata cattaacagc ttctagtaaa

961 aaatccttga tagtaataat aaattatttt gaaagatatc cgttattttc aagtaaatat

1021 ttagattata aagattgaaa ggaaatagtt caattaatat tagaaaataa acattataca

1081 gaagagggtt taatgaaaac agatcttgta aaaaaaagta tgaatagaca aagaacctat

1141 ttcaattgag atcatattac caatttaaat atttagttta ctaaaataaa cattaaatcc

1201 taaattatgg gaatgccgtt aaagagtgta aattctttaa ttattacttc gctatatgca

1261 tggaatctct cgaatatgga attacttttc cagtcaacag atacacagac acctaatagt

1321 atatttttat aatattcaag gtttattttt aaccgtatta gtcgtaacac ttgtgtatac

1381 atgggaagaa tatgcaggaa accaacagat agttttcttt aaatatctta gtaggatcct

1441 cagagactac acgcgaagcc cctgtaacga gggtgaaaac atagtccgtg atggtaggaa

1501 actactattg aatatatata tatatattac gattcccgag attaaataat ataagttttt

1561 gattattacc gcctagtcta ttgttattaa tattctcagc atgtatagaa ggtggagctg

1621 gaacaggttg aactatatat cctccattat ctggaataca aagtcatagt ggacctagtg

1681 ttgatttagc tatatttgca attcatttaa caggactaag tagtttatta ggagctatta

1741 atttcataac aacagtagtt aatatgagaa ctccgggtat aagattacat aaattatctt

1801 tatttggatg agctgttgtt attacagctg tgttattatt atgttcttta cctgttttag

1861 ctggtggaat tactatggtt ttgacagata gaaattttaa tacatctttc tttgaaacag

1921 ctggaggtgg agatcctatt ttattccaac atcttttctg attcttcgga catccagagg

1981 ttaattttat aagcctctta atgtcgctat atgctgggaa agcttcgcta tttagtttta

2041 aatactccct ctttattgat atagtaaaaa agttaaaaca atgaagtcaa tcagcaggta

2101 acatttttaa atttaaaaat ggaacctcag agactatacg cgacaatact gaaaaaataa

2161 aaaatatatc tattcatgtc cctgaacact taaaaccatt aaatgatcaa caattaggac

2221 actatttagc tggtttaata gacggagatg gacattttag ttctaaacaa caattagtaa

2281 ttgtatttag ttctccagat attaaattag cttattatat taaagaaata ataggttttg

2341 gtcatgtaaa taaagttaaa gataaaaatg cttatttata tattatatct aataaagaag

2401 gtataattaa aacgataaat ctaattaatg gtaaattaag aactattaat aaatttaatc

2461 aagttattaa taatatttta tctaatccta agtattcaga agaaaaatta gaatttaaaa

2521 ttaatgattc taatgatttt aataatcact gaatagcagg attttctgat gcagatgcta

2581 gttttcaaat taaaattatt aatagagata ataaacttaa atcagaaatt agattaaact

2641 ttcaaataga tcaaaaagat aataatttat tattatcaat taaagatgta tttggaggta

2701 acataggtta tcgtaaaaat caagatactt attattatgg atcaactagt tttggttcag

2761 ctaaaaaagt aattaattat tttgatagat ttaatttgca atctagcaaa catattaatt

2821 atttaaaatg aagaaaaaca tatataatag tacaaaataa agatcattta acagaaacag

2881 ggatagaaaa aataaaaaat ttgaaaagtt caatgaatag atattcagta taagatagag

2941 tcctaacaag aatgaaagtt cttgagtatt aattttaata gatcttatta gactatttta

3001 ttaatcaaaa catttgttat attttagtta taccaggttt tggtataatt agtacaacta

3061 tatctgcaaa ttctaataaa tcagtgtttg gttatatagg tatggtttat gctatgatgt

3121 ctattggtat attaggtttt attgtttgaa gccatcatat gtatactgtt ggtttagatg

3181 tagatacaag agcatatttt acagctgcta cattaattat tgctgtacct actggtatta

3241 aaatattctc ttgattagca acttgttatg gaggttctat aaaattaact ccttctatgt

3301 tatttgcttt aggttttgta tttatgttta ctataggagg gttattaaac cacttggctc

3361 tccctttaaa gatcaccata tgctgggaac ttctaataat tatactacta gaaatctttt

3421 tgttttcagt gacaatgtat aattttgaac aatcagcagg taaccaacgg ataaatttaa

3481 atatcctagt aggtacctca gagactacac gtgatcctca taaatctttt atgagaagat

3541 atagtccatg aaataaaaat tcattaatta atttatataa ctacaattat aacttgaaat

3601 ataataaaaa taatgttcgt ctatattcta gttttatacc taaaaatata aaagaagata

3661 atataaaaaa tctagaatat ataaaaagat atgaaaattt tgaagaagat agaagtcgta

3721 ttttaaaaga agaaaaggtt gaatctggtg tttactgttt aattaataat acaaatggtc

3781 atagttatgt aggtagttct aataatttgg cctctagaat gaaaaattat cttaataatt

3841 cttttttaaa aagcagacaa aacattaata tgccaatagt aaaagcttta ttaaaatacg

3901 gtcaatctaa ttttactctt cttatcttag aatatataga aacagaatct ttaactatta

3961 gagagacttt ttatataaca catattatgc ctcattataa cgtattaaag caaggttatt

4021 cttctttagg ttataaacac acagaggaaa ctaagaattt attatctgaa ttagctacta

4081 atagaataca ttctgaatcc actaaaagtt taatatctag agctttaata ggtgaaaata

4141 atccttttta taataaaaat cattccactg aaactaaggt aagaatgata gaagctaatt

4201 caaattatcc tgtttatgtt tatgattctt taaaaaactt attagttatt tttccatcag

4261 tttttactct atctaagtta attcattcag atcatcctac attagttaaa gttattaagg

4321 aacaaactat ttttagaggg gaatgatatt taagtaatat accttataat ttaaaagata

4381 ctcctataat atctagttga tcttcagagg aaagtaaaag attagtaagt aatataaatg

4441 atcaagttca tattaaaaaa gcaatatttg tgtatgatga acataaaaat ttattatcta

4501 aatacgaagg ggtaactaaa gctcaagcag ctttaggtat aaatcatgtt acaataaaaa

4561 aacatgcaaa aacacaagct atttataatg gttatatatt tagttatgaa agattaacaa

4621 attaatgaat tctattcgta agtggagttc ttttagctaa tgctgcactt gatacagcat

4681 tccacgatac atattatgta gttgctcatt ttcattatgt attaagtatg ggagcagttt

4741 ttgctttatt tagtggatga tatttctgag taccaaaaat attaggttta agttataata

4801 tgttattatc aaaagttcat ttctgaatat tatttatagg agttaattta acattctttc

4861 ctcaacattt tttaggtcta caaggaatgc cacgtagaat aagtgattat cctgatgctt

4921 ttgcaggttg aaatttagtt agtagttttg gttctatagt aagtgttgta gcttctggat

4981 tattcttata tattgtttat aaacaattat tagataataa tcctgtaggt agattccctt

5041 gattaattcc acaatatttt actgatactt tacaagcatt attaactaga aattacccta

5101 gtttagaatg agctttaact agtccaccta aacctcatgc atttgtaagt ttacctttac

5161 aatctaatat tattaattta tatttataat ataaaattat atatagtata taaatcatat

5221 atattatata tatattatta ttagcttaat ggtaaagcaa atattttaat ttagttcgat

5281 tctaaaataa taatctaaaa ctattttatt atgattataa atttttatat tattatcatt

5341 tatataaaat aaataaaaat tttattttac aaatgttata ttttcccact ttaatgtcta

5401 ttatagaagt tattttagta cttgttcctg cattgcttgc tgttgcattt gtaacattag

5461 ctgaaagaaa aacaatggca agtatgcaaa gaagattagg tcctaatgct gtaggttact

5521 atggattatt acaagcattt gcggatgctt taaagcttct tttaaaagaa tatgtagctc

5581 caactcaagc taatattata ttattctttt taggccctgt gataacttta gttttttcat

5641 tattaggtta tggtgttata ccttatggac caggattagc tattagtgat tttagtttag

5701 gtattttata tttattagct gtatcatctt tagctacata tggtatacta ttagcaggtt

5761 gaagtgctaa cagtaaatat gctttcttag gttctttaag aagtacagct caattaatta

5821 gttacgaatt agttttaagt tctgctattt tattagtaat ctttttaaca ggtagcttaa

5881 atttaactgt taatatggaa tcacaaagag ctatttgatt tatattacct ttattaccta

5941 tatttattat attttttata ggttctgttg ctgaaacaaa tagagctcct tttgacttag

6001 cagaggcgga atcagaactt gttagtggat ttatgacaga acatgctgct gttgtgtttg

6061 tattcttttt tcttgcggaa tatgccagta tagttttaat gtgtatatta acaagtatat

6121 tatttatagg aggatactta acacctagta actttttaat agatatgtta ttacatgttt

6181 atggtgttga tgttttaatt gatacaatta gaaatagtcc tatactagaa ggtttaattt

6241 atggtttaaa tttaggaata aaaagttcta tatttatttt tgtgtttatt tgagttagag

6301 cttcatttcc tagaatacgt tttgaccagt taatggcatt ctgttgaact gtattattac

6361 ctattatatt tgcagttatt atattaattc ctgctatttt atacaatttt actttatttc

6421 ctatcaatat tagtttactt taatttatag atatacatta atttattatc atgaagttta

6481 catagggtgt atatttttat agttaaattt aataatttag gcatacttat atactataaa

6541 tatataaaaa agaatgttat tatctttttt attattagta cctttaatag gtattttttt

6601 catagctgga accatttctt atgaagataa tgcaataaaa attacatatt ataaaaatat

6661 tgctttaatt acatctatta taaatttaat aatctcttta tttgtatata tattatttga

6721 ttctaatact aatcaatttc aatttgtaca agaacattat aatttaagtt tttttgatat

6781 ttatttagga gtggacggaa tttcaatata ttttgtatta ttaactacaa taataatgcc

6841 tattgctata ttatctaatt gaaattcaat aactgaaaat ataaaatctt atttaatatt

6901 aatgttatta ttagaaacgt tattattagc tatattttta gttttagata tattattatt

6961 ttatattttt tttgaaagta cactacctcc tttattttta ttaataggtt tatttggttc

7021 taataataaa gttagagcaa gtttttatat tttcttgtat acattatgag gatctttatt

7081 tttattatta tgtattttaa ctatgtcttc tataatggga actacagatt ttgatgcttt

7141 atttaaaact aattttgatt atataacaca attgttttta tttggaggtg tatttttatc

7201 atttgctgta aaaactccta ctatcttttt aaacaattga ttattaaaag ctcatgttga

7261 atcaccttta gggggaagta tagtgttagc agctatagtt ttaaaaacaa gtttatatgg

7321 tatatgtaga ttaattttac ctatattacc aaaagcatct attaatttta catatatagt

7381 ttatgtaata ggtgttatta ccataattta tgctagtttt agtacattaa gaactacaga

7441 tattaaagag ttaatagctt atagttctgt atctcatgct gctgtgtatt taataggagt

7501 atttagtaat acaattcaag gaattgaagg aagtatactt ttaggtttag cacatggatt

7561 tgtttcaagt ggtttattta tatgtgcagg aggtatatta tatgatagat ctggaactag

7621 atctatttac ttttataaag gtatagctca aattatgcca ttattttcag tattattttt

7681 tatattatct ttaggtaatt gtggtgttcc tttaacatta aattttgtag gtgaatttat

7741 gtctctttat ggtgtatttg aaagattacc tttattaggt gtatttgcta gttcttctat

7801 tatactatct gctgcttata caatatacat gtttaataga ataggatttg gaggtatatt

7861 tagtaaattt tttttggaaa atatagctga tttaacaaaa agagaattct tcttactttt

7921 tattttagtt ttatttacta ttatttttgg tatatatcct tcttttatat tagacggttt

7981 acattattct gttacaggtc ttatatatta ttcataaaaa ttctttttat aattaaataa

8041 ttattaataa tacttaagta tttaatatca aaaaaaaaaa caatgccaca attagtacct

8101 ttttattttt taaatgaagt aatatttact tttgctattc ttactatagt agtatatata

8161 tcttctaaat acatattacc tagatttata cgtttatttt tatctcgtac atttatatca

8221 aaactttttg ataataaata aatattgtaa tataatataa gattaaataa gttatacaag

8281 gatttttcaa gacatataat gtagcttaaa gtactatgat tactttaagc gttaataact

8341 tagatacact aaatttagaa atacttagtc ctttaactca atttgaaata agagatttat

8401 taagcataga tgcacctcta ttaggaaact tacatctttc tataacaaac ataggatttt

8461 atttaacaat aggtgctttt attatattaa ctttaagttt attaagtaca aactataata

8521 aattaataag taataattga tcaataagtc aagaatcttt atatgcaact atacatagta

8581 tagttacaaa tcaaataaac cctaaaaacg gtcaaatata ttttcctttt atttatactt

8641 tattcatatt tattttaata aataatttaa taggaatgat accttatagt tttgcttcaa

8701 ctagtcattt tgttttaaca ttttctctta gttttactat agttttaggt gcaacatttt

8761 tagggttcca aaaacatgga ttagaattct tttctttatt agttccagct ggttgtccct

8821 taccattatt acctttatta gtattaatag aattaatatc ttatttagct cgtaatatat

8881 ctttaggttt aagacttgcc gctaacataa tgagtggtca tatgttactt catattttag

8941 ctggttttac ttataatata atgtcttcag gtataatttt ctttttattg ggattattac

9001 ctttatcatt tattatagct tttgctggat tagaaatagg tatagcattt atacaagctc

9061 aagtatttgt agttttaact tcttcttata ttaaagatgg attagattta cattaaaaaa

9121 aaaaagaatt tagaggtaaa tttcaaagga aagtctatag ttaatataca tatatataaa

9181 taaaaattta aagttttaaa tagaaaatat caatatgtta ataaataatt taatattaaa

9241 taacacaaat atattgcgga catacaatct tttatataac ttttaataca gaaagataat

9301 taacaagata tagcttatat ttaccctaaa ttaaaaaaaa attagtataa acctttaagt

9361 gatgtaattg attattacaa ttacatcata atagatgagt ttggtgatgg ctctgattga

9421 acactgtcca aatgcttgac acatgctaat cgaacgttta atttaattaa taattttatt

9481 aaaagtggtg aacaggtgaa tattagatta tcatactacc ttaaggtaag ggggaaaatc

9541 ccttatatta ataaagtgaa ttacgcctta agatgacacg ataaatcata gagataggta

9601 gttgttaagg taatggctta actagccaac gactctctta gtcgaaactg aaagggttga

9661 tcgaccacat tggggatgaa aaaatcccaa ggcaaagaag tacagcagtg gggaattttg

9721 gtcaatggcc taacggctga actggcaact tggaggaatg gcctaccttg ttttttttgt

9781 gtaattttgc aaggaattaa agtgtaagtg gttaaatcca tatggaataa aattctaaat

9841 atataattta taatgacaat atatatttat gtcttgacta attacgtgcc agcagtcgcg

9901 gtaatacgta agagactagt gttattcatc tttagtaggt ttaaagggta cccggacgga

9961 aaaaatttgc ctgaaaagga acaattttta tctagagttt aatgtaaaaa ggtagtactc

10021 gtggagtaga gataaaattc gtttatacct ataagaggac tggtaaaggc gaaagcgacc

10081 ttttatgtaa aaactgacgt taaaggacga aggcgtagag cacgaatagg attagatacc

10141 ctagtagtct acgcagaaaa ttatgaatgc cataagtaaa gttttacttt gcctataaat

10201 gaaagtgtaa gcatttcacc tcaagagtaa cgtggcaaca tgggaactga aatcactagg

10261 ccgtttctga cacaagtagt gaagtatgtt atttaattcg atggtccacg aaaaacctta

10321 ccacaatttg aattatattt gtaatattta tattataaat ataactagtg ttgcacggct

10381 gttttcagtt aatgttgtga aactgtggct tagaccatga aattaacagg atcctttact

10441 ttatttataa aaattttttt tataaagttg ttttatcaaa tattgataga agaataaggt

10501 ttaagacaag tcatcatggc ctttgtattg tgggctatag acgtgccaca tatacctaga

10561 caaagagaag cgaaaatgtg aattttagct aatctcaaaa aataggataa aattgaatat

10621 ggattatagt ctgaaactcg actatatgaa taagtaatta ctagtaatcg tgaatcagca

10681 tgtcacggtg aatataatct cggactggta ctaaccactc gtcgcatgct gaaaagagtg

10741 tgtgcaataa gtttgcttta ttattataag taagtaaata atagggttta taacttatag

10801 taatattctt catatgtatg actctaatta gtgttaagtc gaaatacggt tcgtgtagtg

10861 gaagttgcac gggatgaatt gaatttaact taaaattatg tataattttt atattataca

10921 ttaaggaggg ttcctttatt ggtaagaagg gttgagctgt aaactcaata gctactttag

10981 ctttaagagt tcgaatctct gtctcctatt tatattttaa atctaaaata taaaatttaa

11041 ccttctatag ctcaacggta gagcatgata ctgttaatta gattaactcg acgtaataga

11101 gcagatagag caatatttaa attaaaacta aaaggaagat taaaaaatac agattcttct

11161 tttaaaggat tatcttctgt atatttaaga ggaaatacca gttctcattt agaatattca

11221 atgggtgtta ataaacgtaa aataggtgct tttgcagtta aaggttgaat taccggtaaa

11281 taataaggtt taataaaaat aaaaaaaaat gaagaaatag tctgaaccat tttgtgaaaa

11341 atggaaataa aaatttatga taacaagttg aacaggctaa tttgcgcaag agtgtgcaaa

11401 atgagtgcgc ggtttggcac ctcgatgtcg gcttaactta tcctcatgga tgcagaaact

11461 atgtagggta cgactgttcg tcgattaaaa agttacatga gctgggttaa atacgtcgtg

11521 agacagtatg gtttctatcc tctagaggga attagaatat aataaggatt aaccttgtac

11581 gaaaggaacc tgggcaacgc cttatatcca aaaggaataa tggttttgtt aacctctggt

11641 ttatctgttg tttatgtgca aaatattaat gtaatagtga ttattatata ataattacta

11701 ttatagaatg tttttttcac tcaattaaat gtatagcata ggcatggcag aaaagctaag

11761 ttagccagag ataagtgctg aaagcatata agtacgaagc tcaccttaag atatttctta

11821 aatatacgta aaataatatt acgcaatagg cttagtttgt aataatttag agattttcag

11881 atactaagta ctaattttat aaataactga atatttaacg tatcttatat ttagcttggt

11941 tagcataaaa gtaatgcaat tgttttgtaa tcaatagaca caagtgcgat acttgtactg

12001 agcttaggat tagtagacaa gtggctaagt catagctttt tcaatgctaa tatcacgggt

12061 tcgaatcctg tctagtctaa tttcaattta agttttaatt tatacctaaa ttagaggcca

12121 taacttaatt ggtagagtgt actgctcata acagtaatta taagtgttcg actcactttg

12181 gccttatatc gcgggttagt gtaacagtaa catttttgac tcatgatcaa aagaaaaagg

12241 tgcaaatcct ttatccgcat taaaatcatt atttttaatc ctagtgatga aattggtaaa

12301 cataacaagc ttaaaacttg taggctaatg ccttaaaggt tcgagtcctt tttaggatat

12361 aatagtattt ttttttataa caataaaatt aaaatttatt aaaaaaaaat aaataaaatt

12421 ttataaaact aagatattag tgtgatgatc ttaaaaataa agtaaatatt tgtattttaa

12481 ctaataaatt ttaatataaa acatctaact tatactttat aagttgaaat ataaatataa

12541 aggttaaaat atataattct tcaaataaat tataaaaata aatcgtaaat atggagagtg

12601 ctgataatat ataagtattt atagtaatac tttttatata aaataaatat atattatagt

12661 tattatacat aaaaatatat tttattaact acaggttaat aaaattttag aaagtgccaa

12721 agaaacaaat atttattata atctacctaa atagacttta tttgattata ttaaatcaaa

12781 taaattattt aaaaataaat attatttttg ttatttttaa tttaataaat aatatagcaa

12841 ctgtaattta atggtaaaat aatcagtttc cacctgaaat ttaagggttc gattcccttt

12901 agttgtacat aagggaatat agtataatgg tattacagtt aggttgcatc taacttattt

12961 aggttcaaat cctaatattt ccatgtatta atgttataat agaaggggtc taatgcaatg

13021 gtagtattgt aatttagctt attataaata tatatttaaa tcttagtaca cttataaaga

13081 taaaactttt ttttaataaa aatttttact gttaaacatt actttaatat ttatcaaaat

13141 aaatttttaa tttctaatat ataatatagc tcagataact caattggcag agtgaaatat

13201 tgaagctatt tatgttgtaa gttcaaatct tactctgagc atattaaatt tgtatgtaaa

13261 aaattttata gtatagttcg aatgttggaa ttggtagaca aagtaagttt aggtcttatt

13321 gattaaattc atgaaggttc aagtccttct tcgaataata taattaattt attaacaaaa

13381 aaattttttt ttatttatta tctttttttt tttgagtata tttatatata tatatatatg

13441 ttacatttaa atagtcatta atattgtata gataaaatat tattgaagta ttaataaata

13501 aataagtaag taaaattata tttaatataa agaatacaat aaaattaaca attttatata

13561 aaaatattat aaatttaata taaaaggtta tatttaaact aaaaatatat taggtatata

13621 tcttaatttg gagtagacta atgggtaagt cctagatttt tgatatctaa aattgagtgt

13681 tcgaatcact tctccaaaaa aaaggagggt aaaatttatt ttctgtttta attttaatgc

13741 tattaataaa aatagcattt ttagactaaa atatatgatt acaataggat aatatagctc

13801 aattggtgga gcgactgtat gtggcacagt aggttctctg ttcgattcag agtattttcc

13861 ctatatagct tgaatagttt aaaggtaaaa ccttattttc atacgataaa gataggtgtt

13921 caattcacct ttcaagctaa gaataaacat aaattaaagt ttttcaaata aatataaatt

13981 tattatatag tgccagaaaa taaaatagca gtatgttata acgacgtatt taaataaaat

14041 aaaaagttat tatttaataa cacatgttat ataaagcgta taaaatgatt aacagaaaaa

14101 tattatatta taaagtcaag gtagtttaat aggctaaaac tttaatttta tatattaata

14161 atgagaattc gattttctct cttgatttac ttatgttaat atatatttat ctatatatat

14221 acatataagt aaaaaaaaaa ataatgcttt taatttcaat attatcttta ttactttcta

14281 atgccgttac tatacgacga gatatatcaa tactttttaa tagagtggct attattgctt

14341 tagtttattc tatactacat agtacaataa gtttatttat tataggtaaa ggtataggtt

14401 tacacggagg tttactaaat attactagtt taacacaaat atttgatatt tttatattct

14461 taattagtat attaatatta caattaacaa gttttttccc tagaaaagta tggataccag

14521 aacattcttc attaacacaa ttattactta ataattttat attttataga acaaaaatta

14581 ttaataaaat gggagaacat ttaagaataa tagaatatcc tttaatatta ttatttataa

14641 tttgtggtgc agtattttta atatctactg gtgatttaat ttctgttttc ctttctatag

14701 agttacaaag ttatggttta tatttattaa gtactatata tagaaattca gaattatcta

14761 ctacaggggg attaatgtat ttcttattag gaggtttaag ttcatgtttt attttattag

14821 gtacaagttt attgtatgca aattcaggta ctactaattt agatggttta tatgctatca

14881 ctagtattag cgatagtacg gatttatgat ataaacctta ttatataaat ttatctttac

14941 ttgtttttag tataggattt ttatttaaag taagtgctgc tccatttcat ttttgatcac

15001 ctgatgttta tgatgctatt cctacaatag ttacaacatt tgtagcaatt atagctaaaa

15061 tatctatatt agtattcttt ttagaaatag tttattatac taaaaattat tttatagatt

15121 ttaattgaac atatggttta ttaattagtt ctttattttc attaataata gggacagttg

15181 caggtttaac tcaatttaga ataaaaaaac tttttgctta tagtactatt tctcatatag

15241 gtttcatact attagcttta agtatatcta gtgtagaatc tactcaagca tttatattct

15301 atttaatgca atattctatt agtaatttaa atgctttctt aatattaata gctataggat

15361 tttctttata ttattatatt agtgataata aagaatataa agagctatta gataaagata

15421 attctcctat acaattaatt agccaattaa aaggttattt ttatataaat cctacattag

15481 cattaagttt agttattact attttttctt ttgcaggtat acctccttta gttggatttt

15541 ttgctaaaca gatggtatta agtgctgctt tagataacgg ttatatattt ttatctttaa

15601 ttgctatact tactagtgta ataggtgctg tatattattt aaatataata aaagaaattt

15661 tttttattca ccacaatata aaataaaccc attattagaa aaattaaatt ttaatggtag

15721 aatatataac aaacaaaata ttttagttaa atctattact tttaaatata ataacattgc

15781 aatatctagt tttctgtcta taacaatatc tataataaca ttaataatat tattatttat

15841 atttgtaaat aaagaatgat taagtatgag taccatattg gtacaattat tatttaatta

15901 ttaaatgagt agtatgagca tattttttat atttgtactt gttatagcag tattattttt

15961 agttattaat ttagtatttg caccccataa tccatatcaa gaaaaatatt ctatttttga

16021 gtgtggattt catagttttt taggtcaaaa tagaagtcaa tttactatta aattctttat

16081 atatgcactt atatttcttc ttctagattt agaaatatta ttaatatttc cttttggagt

16141 tagtggttat actaacgatt tatacggatt aattatagta ttaggattta taactattgt

16201 tacaataggt tttgtatatg aattaggtaa aaatgcttta aaaatagata gcaggcaatc

16261 tataataaaa aataaaagta ataatataaa aatttttact ttataaaaat aaatatatat

16321 atatatatat ttatttaaaa ttatgtgata ttatatctaa atactaaaat aaatgatatt

16381 caactcatta atttaagata ttttttaata ataaattaaa aatggtatta gttgcaaaaa

16441 taattggaac aggattagct acaacaggtt taataggtgc tggtgttggg attggaatag

16501 tatttggtgc tttaatatta ggtgtagcta gaaatccatc attaagaggt caattatttt

16561 catatgctat attaggattt gctttctctg aagctacagg attatttgca ttaatgatgg

16621 ctttcttatt attatacgtt gtttaattat ataagtaaaa agaaaaaaaa catgatatta

16681 acaaaattaa actcaaatca gataaattac actaatagaa gtaatttcag tactagtaca

16741 cgtttatata aaatagacgc tactggatta acattttctc agaaatccat tttagcctct

16801 aatatttcaa caggtccaga tatacctttt agattaaatg atttatattc tcttggtaga

16861 ttattaggtc aagaggttcc tattacacaa aatgactttg taggaacacc agacttaagg

16921 tcaactctaa cgatatcggc aacaagtaat gaggctatta atatgactag tagggttaca

16981 catctaaata attatgaatt attagcgatt ataacagctt tagctatagt aggttttggt

17041 gttattgcga taactgctcg tttaagacga cttactcgaa tagttgaaaa tgcagttaca

17101 gaagaaacac ctgcttgaga catacgtaat caaacaaatg gtttagaaag agatattctc

17161 gatgaattta gtttattaac ggatagaatg gatgaagctg ctctaagttt agctcgatat

17221 attcaagtac aagaacactc aaacttagat atgataccta gaagtactat agctactata

17281 aatgaattgc aacctcaatg gaatgaaatg ttacgagctt taggtgagaa tttagcatcg

17341 aataatacaa taaccttttt aggacgagag aatcaattat atctttatca aaattgaatg

17401 caaatactta ctgaaagtat gcaagaattt ttagttcaat atgaaatttt tttagatttt

17461 ttattttttt aattattatt tgctttaatt atgatattaa gtttaatatt ataataaact

17521 ttaatgttat tatttaattc tgtataatat gaaataaaat aatttaatta ttttagacta

17581 cagtacagaa aattatactt aaacataaac tatgaattta attttaaata atttaattat

17641 gaaattagat gcacctgaag cttgaggtat atatttccaa gatagtgcta caccccaaat

17701 ggaagggtta gtagaattac atgataatat aatgtattat ttagtaataa ttttatttgc

17761 tgtagggtga atattattat ctgtaataag aaattttgtt atgagtactt ctcctatatc

17821 tcataaatat ttaaatcacg gtacattaat agaattaatt tgaactataa caccagctgt

17881 tatattaata ttaatagctt tcccatcatt taaattatta tatttaatgg atgaagttag

17941 tgatccctca atgtcaattt tagcagaagg tcatcaatga tattgaagtt atcaataccc

18001 agatttttta gattctagtg aagaatttat agaatttgat tcttatattg ttccagattc

18061 tgatttagaa gaaggaggac taagaatgtt agaagtagat aatagagtaa taatccctga

18121 attaacacat attagattta ttataacatc aggtgatgtt atacattctt tttcttgtaa

18181 ttctttaggt ataaaatgtg atgcttatcc tggaagatta aaccaagtat ctgttttcgt

18241 aaatagagaa ggagttttct atggtcaatg ctctgaaata tgtggtatat tacatagttc

18301 tatgcctatt gttattgaat ctgtttcttt agaaaaattt ttaacatgac ttgaagaaca

18361 atagatcaat aaagaaataa ttatattata tatatgtata tgtatatata attaaaagtg

18421 tgttaactta attggttaag tatagtacta cggatactaa aattatgagt tcgaatctca

18481 tacacatttc tcttaacctt gtttaagaaa acctttttca aataatttat ttatctatat

18541 atatttatta tgaatataac tttaatactt tttttaatag gaattttagg atttgtttta

18601 aatagaaaaa atattatatt aatgcttatt tctatagaaa taatgctttt atctataaca

18661 ttcttaatat tgataagttc acttaatatt gacgacataa taggtcaaac atacgctatt

18721 tatataataa ttgtagctgg ggcagaatca gccataggtt taggtattct cgtagccttt

18781 tatagattaa gaggaagtat tgcaatagaa tataaataat gtatttaagt ataataattt

18841 tacctttatt aggatctata gtttccgggt tttttggtag aaaagttgga gttagtggag

18901 cacaaattat aacatgttca agtataatag ttacaactat attggctatt attacttttt

18961 ttgaagtagg ttttaataat attgttttat ctatcaattt atttagatgg atagatagtg

19021 aatgatttaa cattatttga ggttttcaat ttgatagttt aactgtatca atgttaatac

19081 ctgtattaat tataagttct ttagttcatg tttactctat aggttatatg aatggtgatc

19141 cccataatca gagatttttt agttatttaa gtttatttac atttatgatg ataatacttg

19201 taacagctaa taattattta ttaatgttcg taggttgaga aggagttggt gtttgttctt

19261 atcttttagt aagtttctga tttactagaa tagcagcaaa tcaaagttct atgtcagctt

19321 ttttaactaa tagagtagga gattgttttt taactatagg aatgtttgca atattatgat

19381 ctttaggtaa cttagattat agtacagtat tttcattagc accttatatt aatgaaaata

19441 ttattataat tataggagtt tgtttattaa taggtgctat ggctaaaagt tcacaagttg

19501 gtcttcatgt ttgattacct atggctatgg agggtcctac tcctgtttct gctttaattc

19561 atgcagctac aatggttaca gcaggagtat atttattaat gcgttcttcc ccgttaattg

19621 aatatagttc tactgtttta ttattatgtt tatggttagg agcaataact acagtattta

19681 gttcacttgt tggtttcttc caacaagata taaaaaaagt tatagcttat tctacaatgt

19741 ctcaattagg aatgatggtt atagcggtag gtttatcttc atataatgtt gccttgtttc

19801 atttagtaaa tcatgctttt tataaaggat tattattttt aggtgctggt gctgttatac

19861 atgcaatggc tgataatcaa gattttagaa aatatggagg attaatatct ttcttacctc

19921 taagttattc tgttatttta atagcaagtt taagcttagt agccttccct ttcatgactg

19981 gattttatag taaagatttt atattagaat ctgcttatgg acaatattgt tttagcagta

20041 taactgtgta cattgtagct gttatagggg caatatttac tactttatat tcagttaaag

20101 ttctttattt aacttttcta actaacccta atggaccttt aataaactat aaacatgctc

20161 atgaaagtga tatatttatg agtttaccat tagttatatt agctatattc tctatattct

20221 ttggatttat aactaaagat atttttatag gtttaggttc aggattcttt atagataata

20281 gtatatttat tcaccctaac tctgaaataa tgatagatac agaatttggt gtttcaactt

20341 attgaaaact attacctttt atttttactg tgttatttag tattatagct ataatcttat

20401 ctgaattttt ctcagatctt atagttaatt ttaaattatc tacattaggt agaactattt

20461 ttgggttttt taatcaaagg tttttaattg aattttttta caataaatat attacaaatt

20521 tagttttaaa tttaggaagt caaacggtta aagttttaga taaaggtagt atagaattat

20581 taggaccata cggtttagaa aaaaatttaa ttacttcaag taaaaatatt agtagtctaa

20641 ataaaggtat tgtaaccaat tatgctttat ttattttagt tggatttata ttatatatgt

20701 tttctataag tttaggtttt actaataatt taagcttaat aatattaata ttaatgttta

20761 gtattagttt aatctcaaaa aaaaaaagta aatttataat aaataaataa atttataata

20821 aataaatatt ttagtacatg atacacaata atacctttta tattaattaa cggtccttat

20881 gttaaataaa aaaaaaataa aaataaaaaa aaatgagaat aataaaaaat aatactatat

20941 taaaattagt taatgcatat cttatagacg cttcacaacc aagtaatata agttatttat

21001 gaaactttgg ttcattatta gcaatttgct taggaataca aattataaca ggtgtaacat

21061 tagctatgca ttatagtccc agtatattag aagcttttaa ctctgttgaa catatcatga

21121 gagatgttaa taatggatga ttaattcgtt atttacatag taatactgct tcagctttct

21181 tctttttagt atatttacat atagctagag gtatgtatta cggttcatat agatcaccta

21241 gaacattagt ttgaacaata ggtgtaataa tacttatact tatgatagca ataggattcc

21301 tgggttatga acttagccca aaatggttta ataaagaatt aaaataccat aataataata

21361 ataataacaa attaatatcc tttaataaaa gatattactc cacaacatct aatataaata

21421 atagtactaa ttctaagaga ttagatgaga tccttgaaga actaaaatta gattatattt

21481 atagttttga agatttaaat ttagaaacta ctaaaaaaga aattttagat aaaactaaag

21541 gtttaagtgg tatttatatg ataataaata aaataactaa agattattat ataggttctg

21601 cctcaactaa tagattttat gctagattta gtaatcatct tatatatttt agaggaagta

21661 aagtagttaa agcagctgta aataaatatg gtttagaaaa ctttactttt attatattag

21721 aattatatcc taatataatt aacaaagaaa ataataaaga attattagat ttagaagata

21781 aatatttaaa attattatta cctaattata atatattaac agaagcagga tctagttttg

21841 gttataaaca tactgaaatt gaccgtcaaa aaatgaaaga tatgtacact gatgaaagac

21901 gagaattaat acgtaattta aataaaggta aaaagctttc tcctgaaaca atagagaaaa

21961 ttagagaaaa agctttaaaa atacctatta gatctataga tactagaagt aattatatta

22021 cacatactag accgattact ttatataatt tagatggtac tatttatggt agttatccta

22081 ctattataga agcggctaaa tctataaatt gtggtgaaaa aacactaata agagcactaa

22141 aaacagaaaa aggtctagta aaaagacaat gaataataaa agacactcta aaataaaatt

22201 attattatta ttattatagt tattatttta actatttatt aaatcatagt cccgtgagta

22261 ataatatttc tgcaattttt ataaaaaaag aaatattaaa gcggtatatc tcgacggaga

22321 tatagaatag tattaaatac tatttggcga tattagtgaa aacgatcaaa gaggtttata

22381 cttcaagatc gtcggttata taaatgatcg cgacagactg ggtcactaat gggtggctga

22441 aatgctgctt aatgcacagt cggaattttt aattaatgtt tattaattaa taaaatatac

22501 gaattcaaag ttattttaat ttattttgta gatttacaga ataaataaat ttgtacgttc

22561 ttccatacgg tcaaatgtct ttatgaggag caacagttat tactaacctt attagcgcaa

22621 taccttgaat aggacaagat atagtagagt tcatttgagg aggtttctct gttaataatg

22681 ctactttaaa tagatttttt gcattacatt ttgtattacc ttttgtatta gctgctttag

22741 tactaatgca tttaatagca cttcatgata cagcggggtc aggtaatcct ttaggggtag

22801 caggaactta tgatagagta ccttttgctc cttatttttt atttaaagat ttaattacaa

22861 tctttatttt tatatttgtt ttaagcttct ttgttttttt tatgccaaat attttaggag

22921 atagtgaaaa ctatataatg gcaaatccaa tgcaaacacc tcctgctatt gtaccagaat

22981 gatatcttct tcctttttat gctatattaa gatctattcc caataaattg ttaggagtta

23041 ttgcaatgtt tggagcttta ttaatattac ttattttacc tattacagat ttagggagat

23101 ctagaggttt ccaatttaga cctttaagta agtttgtttt atacttattt ggtgtagact

23161 ttttattatt agctcgttta ggtgctgtac atgtagaaga cccttttatt caaattgggc

23221 aaatttgtgc atgtatttat tttgtatttt ttattttttc attacctgta gcaagtgtta

23281 ttgaaaatag ttttatggat attgcttatg taacagaaga aaataaaatt tcaataaaaa

23341 ataaattaaa ataataatta tttttatatg tataaataaa ttttcatgac gcacctgata

23401 tgtctattaa gcagctaaag tatttattag agatctgtta cctggaacat tatcagcaat

23461 tatgttggca tacgcaaatc atattattta agcagcaaat atggctaata tttttatgga

23521 accaactaat gaacatatta tatctacagt acaaggaatg attgattttc atgttagtgt

23581 ggagaatacg tttaggaaaa ttgataatct actaatactt tattttacgg agatttaatt

23641 tttaattatt aactataatt atacttgctc taaatatgct tcagggtact caagttgaga

23701 tagccatttt agaaaattat aatattcaat ttacacttat ttgtagtgaa ttaatataaa

23761 atcttgattt tttaagataa cttacaaccg attttgtaat tctacatcct tataattttg

23821 caatctttta ctcaaagata gagaatttag ataatcttat agttaacctt tatttacaag

23881 taggggatac atatgcgaat agttatttaa aataaatgag gagtttatat gagaataact

23941 atgcagaaat agtaagatca ttttaatgat ttacttcatt tcttttaatt tatttttcat

24001 aattattttt ttaaataaca tttttaaatt aaataattta taatattatt atataatatt

24061 aggattatga agtaaacggt ttacacttaa attgcaaatt taaagtttga ggttcgactc

24121 ctccgtaatc ttatgtacta aacataatat aatagaaatt taatattttc ttattagctt

24181 aatggtaaag caagatactt ctaatatcca gattttagtt cgaatctaaa atgagaattt

24241 tttttttatt tttaatacat aatttaatac tatttagtta tataatttat tattattatt

24301 agtttgagtg ataaacggaa aaatattttt aatttaaaaa gtacaagtaa tatattaaat

24361 tcatctatat ctatgccaat agaaagatga tttttatcta caaatgctaa agacattgga

24421 actctttatc taatatttgc attattttca ggactattag gtacagcatt ttctgtttta

24481 ataagaatgg aacttagtgg accaggtgtt caatatatag ctgataatca attatacaat

24541 agtataataa cagctcacgc tattttaatg attttcttta tggttatgcc tgcattaata

24601 ggtggttttg gtaatttctt attaccttta ttagtaggag ggccagatat ggcaaaatta

24661 aaagttctcc ttggatttaa taaaagatat tttagtaaaa atttaaataa taacaatagt

24721 aattttaata gttatttagc tggtttattt gaaggagatg gacatatttg aattcaaaaa

24781 accattggat caaaaacaca taatcctaga ttctgtataa ctttcaatat gaaaaatgaa

24841 cctttagcta aaaaattatt ggatatatta ggatctggtt ttattagata taaattacaa

24901 gataatgctt gtgtattagt tatttcacca gtaacaggtt taaaaagagt tgttgattta

24961 ttaaatggtg aattaagaac tccaaaaata caccaacttc atagtttaat agattgatta

25021 aataaaaatc ataatactaa ttatagtaaa ttagatataa aagacagtcc actatctaat

25081 gatgcttgat taagtggatt tgtagattca gatggtagtt tttctgtttt atatagtaaa

25141 acagaaaatg gagcaaaaaa aagaaaaatc acttgtagat taagaatcga acaaagaata

25201 ttagatcctg tatctaaatg tagctatgaa caaattttat caaaaataac aaattttctg

25261 atttgttcat tactaactaa aactcaaaaa tctactggta ataagtacta tacattaaca

25321 gcttctagta aaaaatcctt gatagtaata ataaattatt ttgaaagata tccgttattt

25381 tcaagtaaat atttagatta taaagattga aaggaaatag ttcaattaat attagaaaat

25441 aaacattata cagaagaggg tttaatgaaa acagatcttg taaaaaaaag tatgaataga

25501 caaagaacct atttcaattg agatcatatt accaatttaa atatttagtt tactaaaata

25561 aacattaaat cctaaattat gggaatgccg ttaaagagtg taaattcttt aattattact

25621 tcgctatatg catggaatct ctcgaatatg gaattacttt tccagtcaac agatacacag

25681 acacctaata gtatattttt ataatattca aggtttattt ttaaccgtat tagtcgtaac

25741 acttgtgtat acatgggaag aatatgcagg aaaccaacag atagttttct ttaaatatct

25801 tagtaggatc ctcagagact acacgcgaag cccctgtaac gagggtgaaa acatagtccg

25861 tgatggtagg aaactactat tgaatatata tatatatatt acgattcccg agattaaata

25921 atataagttt ttgattatta ccgcctagtc tattgttatt aatattctca gcatgtatag

25981 aaggtggagc tggaacaggt tgaactatat atcctccatt atctggaata caaagtcata

26041 gtggacctag tgttgattta gctatatttg caattcattt aacaggacta agtagtttat

26101 taggagctat taatttcata acaacagtag ttaatatgag aactccgggt ataagattac

26161 ataaattatc tttatttgga tgagctgttg ttattacagc tgtgttatta ttatgttctt

26221 tacctgtttt agctggtgga attactatgg ttttgacaga tagaaatttt aatacatctt

26281 tctttgaaac agctggaggt ggagatccta ttttattcca acatcttttc tgattcttcg

26341 gacatccaga ggttaatttt ataagcctct taatgtcgct atatgctggg aaagcttcgc

26401 tatttagttt taaatactcc ctctttattg atatagtaaa aaagttaaaa caatgaagtc

26461 aatcagcagg taacattttt aaatttaaaa atggaacctc agagactata cgcgacaata

26521 ctgaaaaaat aaaaaatata tctattcatg tccctgaaca cttaaaacca ttaaatgatc

26581 aacaattagg acactattta gctggtttaa tagacggaga tggacatttt agttctaaac

26641 aacaattagt aattgtattt agttctccag atattaaatt agcttattat attaaagaaa

26701 taataggttt tggtcatgta aataaagtta aagataaaaa tgcttattta tatattatat

26761 ctaataaaga aggtataatt aaaacgataa atctaattaa tggtaaatta agaactatta

26821 ataaatttaa tcaagttatt aataatattt tatctaatcc taagtattca gaagaaaaat

26881 tagaatttaa aattaatgat tctaatgatt ttaataatca ctgaatagca ggattttctg

26941 atgcagatgc tagttttcaa attaaaatta ttaatagaga taataaactt aaatcagaaa

27001 ttagattaaa ctttcaaata gatcaaaaag ataataattt attattatca attaaagatg

27061 tatttggagg taacataggt tatcgtaaaa atcaagatac ttattattat ggatcaacta

27121 gttttggttc agctaaaaaa gtaattaatt attttgatag atttaatttg caatctagca

27181 aacatattaa ttatttaaaa tgaagaaaaa catatataat agtacaaaat aaagatcatt

27241 taacagaaac agggatagaa aaaataaaaa atttgaaaag ttcaatgaat agatattcag

27301 tataagatag agtcctaaca agaatgaaag ttcttgagta ttaattttaa tagatcttat

27361 tagactattt tattaatcaa aacatttgtt atattttagt tataccaggt tttggtataa

27421 ttagtacaac tatatctgca aattctaata aatcagtgtt tggttatata ggtatggttt

27481 atgctatgat gtctattggt atattaggtt ttattgtttg aagccatcat atgtatactg

27541 ttggtttaga tgtagataca agagcatatt ttacagctgc tacattaatt attgctgtac

27601 ctactggtat taaaatattc tcttgattag caacttgtta tggaggttct ataaaattaa

27661 ctccttctat gttatttgct ttaggttttg tatttatgtt tactatagga gggttattaa

27721 accacttggc tctcccttta aagatcacca tatgctggga acttctaata attatactac

27781 tagaaatctt tttgttttca gtgacaatgt ataattttga acaatcagca ggtaaccaac

27841 ggataaattt aaatatccta gtaggtacct cagagactac acgtgatcct cataaatctt

27901 ttatgagaag atatagtcca tgaaataaaa attcattaat taatttatat aactacaatt

27961 ataacttgaa atataataaa aataatgttc gtctatattc tagttttata cctaaaaata

28021 taaaagaaga taatataaaa aatctagaat atataaaaag atatgaaaat tttgaagaag

28081 atagaagtcg tattttaaaa gaagaaaagg ttgaatctgg tgtttactgt ttaattaata

28141 atacaaatgg tcatagttat gtaggtagtt ctaataattt ggcctctaga atgaaaaatt

28201 atcttaataa ttctttttta aaaagcagac aaaacattaa tatgccaata gtaaaagctt

28261 tattaaaata cggtcaatct aattttactc ttcttatctt agaatatata gaaacagaat

28321 ctttaactat tagagagact ttttatataa cacatattat gcctcattat aacgtattaa

28381 agcaaggtta ttcttcttta ggttataaac acacagagga aactaagaat ttattatctg

28441 aattagctac taatagaata cattctgaat ccactaaaag tttaatatct agagctttaa

28501 taggtgaaaa taatcctttt tataataaaa atcattccac tgaaactaag gtaagaatga

28561 tagaagctaa ttcaaattat cctgtttatg tttatgattc tttaaaaaac ttattagtta

28621 tttttccatc agtttttact ctatctaagt taattcattc agatcatcct acattagtta

28681 aagttattaa ggaacaaact atttttagag gggaatgata tttaagtaat ataccttata

28741 atttaaaaga tactcctata atatctagtt gatcttcaga ggaaagtaaa agattagtaa

28801 gtaatataaa tgatcaagtt catattaaaa aagcaatatt tgtgtatgat gaacataaaa

28861 atttattatc taaatacgaa ggggtaacta aagctcaagc agctttaggt ataaatcatg

28921 ttacaataaa aaaacatgca aaaacacaag ctatttataa tggttatata tttagttatg

28981 aaagattaac aaattaatga attctattcg taagtggagt tcttttagct aatgctgcac

29041 ttgatacagc attccacgat acatattatg tagttgctca ttttcattat gtattaagta

29101 tgggagcagt ttttgcttta tttagtggat gatatttctg agtaccaaaa atattaggtt

29161 taagttataa tatgttatta tcaaaagttc atttctgaat attatttata ggagttaatt

29221 taacattctt tcctcaacat tttttaggtc tacaaggaat gccacgtaga ataagtgatt

29281 atcctgatgc ttttgcaggt tgaaatttag ttagtagttt tggttctata gtaagtgttg

29341 tagcttctgg attattctta tatattgttt ataaacaatt attagataat aatcctgtag

29401 gtagattccc ttgattaatt ccacaatatt ttactgatac tttacaagca ttattaacta

29461 gaaattaccc tagtttagaa tgagctttaa ctagtccacc taaacctcat gcatttgtaa

29521 gtttaccttt acaatctaat attattaatt tatatttata atataaaatt atatatagta

29581 tataaatcat atatattata tatatattat tattagctta atggtaaagc aaatatttta

29641 atttagttcg attctaaaat aataatctaa aactatttta ttatgattat aaatttttat

29701 attattatca tttatataaa ataaataaaa attttatttt acaaatgtta tattttccca

29761 ctttaatgtc tattatagaa gttattttag tacttgttcc tgcattgctt gctgttgcat

29821 ttgtaacatt agctgaaaga aaaacaatgg caagtatgca aagaagatta ggtcctaatg

29881 ctgtaggtta ctatggatta ttacaagcat ttgcggatgc tttaaagctt cttttaaaag

29941 aatatgtagc tccaactcaa gctaatatta tattattctt tttaggccct gtgataactt

30001 tagttttttc attattaggt tatggtgtta taccttatgg accaggatta gctattagtg

30061 attttagttt aggtatttta tatttattag ctgtatcatc tttagctaca tatggtatac

30121 tattagcagg ttgaagtgct aacagtaaat atgctttctt aggttcttta agaagtacag

30181 ctcaattaat tagttacgaa ttagttttaa gttctgctat tttattagta atctttttaa

30241 caggtagctt aaatttaact gttaatatgg aatcacaaag agctatttga tttatattac

30301 ctttattacc tatatttatt atatttttta taggttctgt tgctgaaaca aatagagctc

30361 cttttgactt agcagaggcg gaatcagaac ttgttagtgg atttatgaca gaacatgctg

30421 ctgttgtgtt tgtattcttt tttcttgcgg aatatgccag tatagtttta atgtgtatat

30481 taacaagtat attatttata ggaggatact taacacctag taacttttta atagatatgt

30541 tattacatgt ttatggtgtt gatgttttaa ttgatacaat tagaaatagt cctatactag

30601 aaggtttaat ttatggttta aatttaggaa taaaaagttc tatatttatt tttgtgttta

30661 tttgagttag agcttcattt cctagaatac gttttgacca gttaatggca ttctgttgaa

30721 ctgtattatt acctattata tttgcagtta ttatattaat tcctgctatt ttatacaatt

30781 ttactttatt tcctatcaat attagtttac tttaatttat agatatacat taatttatta

30841 tcatgaagtt tacatagggt gtatattttt atagttaaat ttaataattt aggcatactt

30901 atatactata aatatataaa aaagaatgtt attatctttt ttattattag tacctttaat

30961 aggtattttt ttcatagctg gaaccatttc ttatgaagat aatgcaataa aaattacata

31021 ttataaaaat attgctttaa ttacatctat tataaattta ataatctctt tatttgtata

31081 tatattattt gattctaata ctaatcaatt tcaatttgta caagaacatt ataatttaag

31141 tttttttgat atttatttag gagtggacgg aatttcaata tattttgtat tattaactac

31201 aataataatg cctattgcta tattatctaa ttgaaattca ataactgaaa atataaaatc

31261 ttatttaata ttaatgttat tattagaaac gttattatta gctatatttt tagttttaga

31321 tatattatta ttttatattt tttttgaaag tacactacct cctttatttt tattaatagg

31381 tttatttggt tctaataata aagttagagc aagtttttat attttcttgt atacattatg

31441 aggatcttta tttttattat tatgtatttt aactatgtct tctataatgg gaactacaga

31501 ttttgatgct ttatttaaaa ctaattttga ttatataaca caattgtttt tatttggagg

31561 tgtattttta tcatttgctg taaaaactcc tactatcttt ttaaacaatt gattattaaa

31621 agctcatgtt gaatcacctt tagggggaag tatagtgtta gcagctatag ttttaaaaac

31681 aagtttatat ggtatatgta gattaatttt acctatatta ccaaaagcat ctattaattt

31741 tacatatata gtttatgtaa taggtgttat taccataatt tatgctagtt ttagtacatt

31801 aagaactaca gatattaaag agttaatagc ttatagttct gtatctcatg ctgctgtgta

31861 tttaatagga gtatttagta atacaattca aggaattgaa ggaagtatac ttttaggttt

31921 agcacatgga tttgtttcaa gtggtttatt tatatgtgca ggaggtatat tatatgatag

31981 atctggaact agatctattt acttttataa aggtatagct caaattatgc cattattttc

32041 agtattattt tttatattat ctttaggtaa ttgtggtgtt cctttaacat taaattttgt

32101 aggtgaattt atgtctcttt atggtgtatt tgaaagatta cctttattag gtgtatttgc

32161 tagttcttct attatactat ctgctgctta tacaatatac atgtttaata gaataggatt

32221 tggaggtata tttagtaaat tttttttgga aaatatagct gatttaacaa aaagagaatt

32281 cttcttactt tttattttag ttttatttac tattattttt ggtatatatc cttcttttat

32341 attagacggt ttacattatt ctgttacagg tcttatatat tattcataaa aattcttttt

32401 ataattaaat aattattaat aatacttaag tatttaatat caaaaaaaaa aacaatgcca

32461 caattagtac ctttttattt tttaaatgaa gtaatattta cttttgctat tcttactata

32521 gtagtatata tatcttctaa atacatatta cctagattta tacgtttatt tttatctcgt

32581 acatttatat caaaactttt tgataataaa taaatattgt aatataatat aagattaaat

32641 aagttataca aggatttttc aagacatata atgtagctta aagtactatg attactttaa

32701 gcgttaataa cttagataca ctaaatttag aaatacttag tcctttaact caatttgaaa

32761 taagagattt attaagcata gatgcacctc tattaggaaa cttacatctt tctataacaa

32821 acataggatt ttatttaaca ataggtgctt ttattatatt aactttaagt ttattaagta

32881 caaactataa taaattaata agtaataatt gatcaataag tcaagaatct ttatatgcaa

32941 ctatacatag tatagttaca aatcaaataa accctaaaaa cggtcaaata tattttcctt

33001 ttatttatac tttattcata tttattttaa taaataattt aataggaatg ataccttata

33061 gttttgcttc aactagtcat tttgttttaa cattttctct tagttttact atagttttag

33121 gtgcaacatt tttagggttc caaaaacatg gattagaatt cttttcttta ttagttccag

33181 ctggttgtcc cttaccatta ttacctttat tagtattaat agaattaata tcttatttag

33241 ctcgtaatat atctttaggt ttaagacttg ccgctaacat aatgagtggt catatgttac

33301 ttcatatttt agctggtttt acttataata taatgtcttc aggtataatt ttctttttat

33361 tgggattatt acctttatca tttattatag cttttgctgg attagaaata ggtatagcat

33421 ttatacaagc tcaagtattt gtagttttaa cttcttctta tattaaagat ggattagatt

33481 tacattaaaa aaaaaaagaa tttagaggta aatttcaaag gaaagtctat agttaatata

33541 catatatata aataaaaatt taaagtttta aatagaaaat atcaatatgt taataaataa

33601 tttaatatta aataacacaa atatattgcg gacatacaat cttttatata acttttaata

33661 cagaaagata attaacaaga tatagcttat atttacccta aattaaaaaa aaattagtat

33721 aaacctttaa gtgatgtaat tgattattac aattacatca taatagatga gtttggtgat

33781 ggctctgatt gaacactgtc caaatgcttg acacatgcta atcgaacgtt taatttaatt

33841 aataatttta ttaaaagtgg tgaacaggtg aatattagat tatcatacta ccttaaggta

33901 agggggaaaa tcccttatat taataaagtg aattacgcct taagatgaca cgataaatca

33961 tagagatagg tagttgttaa ggtaatggct taactagcca acgactctct tagtcgaaac

34021 tgaaagggtt gatcgaccac attggggatg aaaaaatccc aaggcaaaga agtacagcag

34081 tggggaattt tggtcaatgg cctaacggct gaactggcaa cttggaggaa tggcctacct

34141 tgtttttttt gtgtaatttt gcaaggaatt aaagtgtaag tggttaaatc catatggaat

34201 aaaattctaa atatataatt tataatgaca atatatattt atgtcttgac taattacgtg

34261 ccagcagtcg cggtaatacg taagagacta gtgttattca tctttagtag gtttaaaggg

34321 tacccggacg gaaaaaattt gcctgaaaag gaacaatttt tatctagagt ttaatgtaaa

34381 aaggtagtac tcgtggagta gagataaaat tcgtttatac ctataagagg actggtaaag

34441 gcgaaagcga ccttttatgt aaaaactgac gttaaaggac gaaggcgtag agcacgaata

34501 ggattagata ccctagtagt ctacgcagaa aattatgaat gccataagta aagttttact

34561 ttgcctataa atgaaagtgt aagcatttca cctcaagagt aacgtggcaa catgggaact

34621 gaaatcacta ggccgtttct gacacaagta gtgaagtatg ttatttaatt cgatggtcca

34681 cgaaaaacct taccacaatt tgaattatat ttgtaatatt tatattataa atataactag

34741 tgttgcacgg ctgttttcag ttaatgttgt gaaactgtgg cttagaccat gaaattaaca

34801 ggatccttta ctttatttat aaaaattttt tttataaagt tgttttatca aatattgata

34861 gaagaataag gtttaagaca agtcatcatg gcctttgtat tgtgggctat agacgtgcca

34921 catataccta gacaaagaga agcgaaaatg tgaattttag ctaatctcaa aaaataggat

34981 aaaattgaat atggattata gtctgaaact cgactatatg aataagtaat tactagtaat

35041 cgtgaatcag catgtcacgg tgaatataat ctcggactgg tactaaccac tcgtcgcatg

35101 ctgaaaagag tgtgtgcaat aagtttgctt tattattata agtaagtaaa taatagggtt

35161 tataacttat agtaatattc ttcatatgta tgactctaat tagtgttaag tcgaaatacg

35221 gttcgtgtag tggaagttgc acgggatgaa ttgaatttaa cttaaaatta tgtataattt

35281 ttatattata cattaaggag ggttccttta ttggtaagaa gggttgagct gtaaactcaa

35341 tagctacttt agctttaaga gttcgaatct cttgtctcct atttatattt taaatctaaa

35401 atataaaatt taaccttcta tagctcaacg gtagagcatg atactgttaa tatcaggaca

35461 aatgttcgat tcattttaga agggtcatat aatatgtata ttctgactat tataagtatg

35521 aaaaaaataa aataattgat attaacctat aaatttatga ttaattctgt tcggagtaac

35581 tttcaggatc atccttttca tttagtatct ccttcgcctt ggccacttta tactagttta

35641 tctttatatt ctttaactac aagtactgca ttatctatgc ataattttaa taacgcttat

35701 ttttttgtat atttaagtgt atttttacta atgtcttcaa tgtttttttg attccgtgat

35761 attataagtg aaagtacatt tttaggtgat catagtttag ctgtacaaaa aggattaaat

35821 ttaggtgtta tattatttat agcatctgaa gctttattct ttttagctgt tttttgagct

35881 ttctttcata gtgctttaac acctactgtt gaattaggag ctcaatgacc gccattaggt

35941 atagaacctg ttaatccttt tgagttacca ttacttaata cagtaatatt gttatctagt

36001 ggtgctacag ttacatttgg tcatcattca ttaatacaag gtaacagaag tggagctgta

36061 tacggtacaa tgattactgg tttattagct ataattttca ctgtttttca aggtatagaa

36121 tatagtgtat catcttttac tataagtgat ggtgcttttg gtacatgttt tttctttggt

36181 acaggatttc atggattaca tgttatgata ggaacaatat ttttacttgt agctttatga

36241 agaatttatg cttatcattt aacagataat caccatctgg gttttgaaag tggaattttg

36301 tattgacatt ttgtagattt ggtatggctt tttttgtatc tttttgtgta ttactgggga

36361 tcttagatat atttatatct atcagtaata ctagtataca aacgatacaa acttttatta

36421 taatagagcg gttcccttta ttatgatcaa tctttatacc tattatattt atactatatt

36481 tttatttttt ttctgaacaa atttgaaaaa atagaaaaat aatgttaatt aaaattttta

36541 aaagaatagc ctttttttat agtttagttt tttttattgc ttttagtgtc tttttacttt

36601 acaatattat tttaaactat actgattttt atttatttaa ttctttaact ttattagatt

36661 tcatatctta tactagaact tctttaatga ttttttatat attcaaatat ataaaaacta

36721 ttatttacca gggatttaat aaagataatt ttttaatatt tatttttggt ttatctataa

36781 ttaatttaat tactatttta attaaaattt ttattacaac attcatacct gaaatttata

36841 ttgtattatc tttagatggt actcctaata caggtaatag ttctggacct caagggtctc

36901 aaccgcctca atggcctcaa gggcctcaag gacctcaacc acctcaagga cctgaaccac

36961 ctcaaggacc taaccatgct caaccatccc acatgtctga ggttacagaa gaacaacaag

37021 aagagttgga ggaaataaaa gacttcgaag aagaatatga agaatctggt aatacagctg

37081 cacaattgac tgcagaatca gataatgctg cggaagatgt aattagaatt accgagaatt

37141 tatatagtaa agaggctgag cttcaggaaa aacaagctag aggtgaaaat acagaacaaa

37201 cacttaacga gattagacat ttgaatgaaa gtttaaatga agctaattca aaggaagaaa

37261 ctctttttgt agaagctgaa cgagcaagta gatatcatga acgagttggt actttatata

37321 aaaataaata tgatgcttat tgtaataaat atggtaataa tcctaataat aacaataata

37381 ataattaaat atgcttattt ataataaatt ataaatttta taatttatta ggggtctttg

37441 tataatggta atacatatga cttttaatca tcaaattgtt ggttcgaatc caacagatcc

37501 taatgctcag agaattactg atatattttt tgatataaat ttataataat tataacaaaa

37561 tataaaatat ttttattaat ataacaaata ttagtattat tatattatgt ttatcttatt

37621 atattataga tacacctata ttaatggata gtttagataa ctctagttta ttaaatgaga

37681 acttagatac aagttcggaa attaatagta ataagaacaa gaataaatat tttattataa

37741 aacattgaat actctagtgt atttcaggta gagatattta ggaggtgaac aggattacat

37801 ggatttaata ttaagtcaga tataactaca taattaaaag attttactaa aagtcctatt

37861 aattatatga ctaataatca agaatctcgt attttagata atcataaaag actaaataat

37921 tcgaatagta attttagtgt ttacggtaaa aatttgatac atgaataagt aaaatggctt

37981 aattttgcag ttaacagtaa tagacctgta aattgttaat ttgtgcctta tattaataaa

38041 tataaacatt aaaatataaa taatattact ttaatatttt aataagaatt aataacttaa

38101 ttggtaaagg acttccctgt cacggaagta gatatcggtt cgactccgtt ttaattcgta

38161 tacaggaaaa attgctatag gtaaggcgag tcaattgcta tttgatgtct ttttagactt

38221 aaaagttcga atcttttttt ttccgtatat aataataagt ttattataca actaataaga

38281 gtatagttta atggtaaaac aaggagcttc aacctccgaa ttctctgttc aaatcagagt

38341 gctcttgagt aaacatacat aataaaaaaa aatttttttt tattgtgtaa tttcctatat

38401 cattaggtac taaattatta gagttataaa tgaataactt atttattata aatgaaagtt

38461 ttacgaatgg gtataaaaat gaggtattag atataatttc catattagtt atattatcag

38521 gtatatttgt tattataagt aaaaacccta ttatttcttt attattctta atcggattat

38581 ttgcaggtat atcttcttat ttattaataa taggtttaag ttttctgggt ttatcttatt

38641 tagttgttta tattggagct gtttcaattt tattcttatt tattttaatg ttaattaaca

38701 ttagaataag tgaattacaa agtgatacta ataatagtat acctttagct ataagtattg

38761 caattttatt taattattct ttgttccaaa tattacctta tgatattgca attttaaaca

38821 ataataacta cttaaataac atattatata acgtatcttt taataagtta gataatgggt

38881 tagaaactag cttaaatata aataataatg atacattatt tgttacaagt aaaatgtgag

38941 atggtaattt agcagaggtt ggtcatatta caagtatagg taacattatg tatacaaatt

39001 ataatttgtg attaattata actagcttta tattattact tgcgatggta ggaagtatag

39061 taattactat gaaaagttca aaataatttt aaataaaaaa atttttttat aataataata

39121 gtggttaaaa aaaatatata tatatatata tataaaggaa ttaactcaat tggcagagta

39181 accgttttac acacggtatg ttatagattc gaattctata ttccttattt ttagtaaatt

39241 tatttatata ataaatattt tttaatttaa tatattgtta ttattttaaa ttccttaact

39301 taatggtaaa gtgcattttt gataagaatg ttatcagcgt tcaattcgct gaggaattaa

39361 gaaagttggc tgaatggtta aagcggctag ttagaaacta gttatatata tttatactat

39421 gtgttcaaat cacatacttt ctgatatata agaagattga ctgagtggct aaagtggtat

39481 gtttgagcca tatttattat ttattaatta cattggttcg aatccaatat cttctgtatt

39541 aatcaaaatt aatttaaata tataaatgat aattattaga ttaagatata taaaaacaga

39601 taaactaaaa aaaaaataaa caattaaaaa taaaatacat atattataac gcggaatgga

39661 gccaaattgg tgaggtgctg tgcctgggcc acagagatac aaagttcgaa actttgcatt

39721 ccgaaaatat tatatacatt ataaacgata taaaaataaa agtattagga atttaagtaa

39781 tatattttac tatttttatt taaaacatct ttcatatgtt gagacatata aattaaaggt

39841 aatatcggca ctaataacaa tagaaatatt attatatatt tgtaccactt tcttctaatt

39901 tgatatattt cctaattaac ttaactatgt taatatttag taaactttca tctattaatt

39961 ttaattattc actaaaatat catccttttc attttaaaaa atcatataaa attacttaaa

40021 atacaataga aataaataat acaaatatta aaaattaata tagatcctat aaccccaact

40081 aaaaaaaaaa gtaatattaa aaattagtta tttttcataa cgtggttatc aaataaggta

40141 tttttattcc atactatata ttataaataa tataatatta aaaattttta agaaatataa

40201 ttgatacgaa ataaaaattt atacataaat ttatgtaagt atataaagag actactaagg

40261 aatactagct atgtattaaa gagaacgtat tatccaatca attaagataa taaactctaa

40321 gagaaatcag ataataaacg aagtaaattg aattatctta ttagcttcag gaaaataaat

40381 caaaagagat tctatgatta gcgtgagcaa aaatagagaa gtctaaataa taagtaaaat

40441 gactctaaag ttgtttgaat tatatgggga accttcctca aagactaaat ataatacata

40501 agcgacagta aaacagtacc gtgagggaac gtacatagaa atagtagttt tataagcagc

40561 tcgagctaaa gcaagagcgt accttttgca taatgggtca ccaagttaat tttagatgcg

40621 agcaaataaa taatgcgcag ttaaaccgat cataaaaaaa atgaatagta tctaaaatta

40681 gacccgaagt acagtgatct taccatggtc aggattataa aggtccgaac gggttatcgt

40741 tgtatagata tccgaggaac tgtggtaagt atagtgaaag acaattcgtg actgtattag

40801 ctggttttct gcgaaaccta taacagtagg caatttaagt aacatcttaa caggtacaga

40861 acttaatctc agacaaaatg taaaaaatac attttatttt gtatatatcg ggggatcatt

40921 tagattttac cggtgagttt gtggactcga aatggtgaag atgaatctta aattatcaga

40981 catagaatga taaggttgta tgtcaaaagg gaaacagccc agaacaagag ttaaggttcc

41041 aaaattatta ttaagtgaaa ttaagaaagt ttttatataa gtcggcaagg agattggctt

41101 agaagcagcc ataatttaaa gacctcgtac cagagcactt gttaaatgtt aaaagcatcg

41161 aaaatttaac ggatctaaat aatataccga taccttgtct ataaataata taaattatat

41221 taacttatat tatttatggg gtagcagaat attgagttaa tcttagtatt tttttttata

41281 aaaaaaatta tattataact caagtaagaa tggtgacatg agtaacgaaa aagtaaatac

41341 tcgcctaaag cttatggtta aaggtaagta acggcctcta agtttataac cttaaggtta

41401 gaacgatgag aaaaatctta ttactattaa atacaacatt ttaagtggaa agtgtgcagg

41461 aaataattag taatactgta ttaaaaaata caataaccgt tcctaaaaac tacaataagt

41521 aagctagtag agaatacgaa ggcgcatgag ataacaatct taaaggaact cggcaaaata

41581 actaccgtaa cttcgggata aggagggctc attattcttg attaatatca ggtaaaaagg

41641 aagaagcata aaatagtgtt gtacgactgt ttaattaaaa caaagcactt tgcataagat

41701 gataaatcta agtattgagt gtgatgtctg cccaatgtcg gtaagttaac gaaagaaact

41761 aaatattata aatttggttt tttaggaatg tccggtcaat ggcggcctta acgtgagggt

41821 cctaaggtag cgaaatgcct tggccgttaa atgcggtctt gcatgaatga tgtaacgata

41881 caacagctgt ctctaagatt gactcagtga aattggaata actgtgcaga tacagtttac

41941 ctctagctgg acgagaagac cctatgcagc tttactgtta ctagttattg agtataaccc

42001 aaacaaattt tagtttagaa ggttcattga taaaatataa gtgaaagacc tttatttgtt

42061 tattatgcta taaacctaca aaaggaaaaa tgattaggag acagtttatg tggggcacag

42121 accccttaaa gagtaaaagg gtgtatctaa aattataact tatatgttaa tataagttaa

42181 tttaatattt tattgtttgt tatttttttt aactttgtta tatttaatca tatacttttg

42241 tgtataaata tatacatgat attaggtaag atttccccta aagagaaatt agttgtaata

42301 aaaatattat atatatatat aatattttta tagttattat gatttatagt cattaataat

42361 tatgttaaat acttattaag ttaaatggct taatcttgct ttactgtttg attcacaaca

42421 aatcttacag tcgcgtaagc ggggcattag atcacaagat acaataagga aaggtcttgg

42481 attattggaa aagctacgct agggatgttt gtccttcaat atttttttaa taattgatat

42541 atattgggtt aatttcaaga attacttatg acattaataa gtaaatttga agcgaagttt

42601 attttattaa aaataaaatt gttcaacgac taaaggtgag ttatataaca ataatccttt

42661 attaatactc aacatttttt tttagtaagt tagtttagta ctattaaatt tataattgca

42721 aaaaaacaca aaattaaaaa aatttttaga tttaaataat aataataata ataatatatt

42781 tattaaaaat attaataata aatataaatt aataccattg aacactataa ataataatgt

42841 aggtgaaata aaatattttc cttctgattt taaagaatga actaataata tttattcttt

42901 taattctaat tatattaaaa attttcctgt ttatgattta aatataaata aattactgaa

42961 gggttatttt gatctatatt taaatcataa atttataaag tctagattta tctcatcaaa

43021 aaataaatct ttatccctta ataaaatctt tgttagtaaa actgaagtaa aacatactag

43081 ctctaaagct ataattactg tctatgtgta taacagagaa agagttgctt tattattaaa

43141 taatttaatt atattgaaga atataaaaat attaaaagaa aaaaaaatag gtataaaaaa

43201 gttatttaaa ttttattttt tttgtaaaag catatccatg gatttatatg ataaatattt

43261 taaaaatatt ttttataaag agttaatttc tataaaaaga agtaagttaa aattagatat

43321 aaatgaattt aaatttaaag atattttttt atataaatta agtaaattaa taggtaaatt

43381 ttataataaa aaagtagaat ttaatattat taatttaaaa tcaattgctt ataactctaa

43441 tatttttaca gaaattttga aaagaaaaat aagcgataga aaagatttaa tttttagaac

43501 aatggatatt attttaagta aaggtataat ttttgaagaa aaaaatatta ataaagaaaa

43561 aaaattagtt aaaaatataa attttaatct tatagaaaat aagtataaaa atttaaatat

43621 taattatata gtaaaaaata ttaatttaaa taaaactata aaagatttat acgatattga

43681 agattatgat aataaagata ttatatttga ttctattaaa tataaaaatt taggaggtat

43741 gaaattagag ataaaaggta gattaactcg acgtaataga gcagatagag caatatttaa

43801 attaaaacta aaaggaagat taaaaaatac agattcttct tttaaaggat tatcttctgt

43861 atatttaaga ggaaatacca gttctcattt agaatattca atgggtgtta ataaacgtaa

43921 aataggtgct tttgcagtta aaggttgaat taccggtaaa taataaggtt taataaaaat

43981 aaaaaaaaat gaagaaatag tctgaaccat tttgtgaaaa atggaaataa aaatttatga

44041 taacaagttg aacaggctaa tttgcgcaag agtgtgcaaa atgagtgcgc ggtttggcac

44101 ctcgatgtcg gcttaactta tcctcatgga tgcagaaact atgtagggta cgactgttcg

44161 tcgattaaaa agttacatga gctgggttaa atacgtcgtg agacagtatg gtttctatcc

44221 tctagaggga attagaatat aataaggatt aaccttgtac gaaaggaacc tgggcaacgc

44281 cttatatcca aaaggaataa tggttttgtt aacctctggt ttatctgttg tttatgtgca

44341 aaatattaat gtaatagtga ttattatata ataattacta ttatagaatg tttttttcac

44401 tcaattaaat gtatagcata ggcatggcag aaaagctaag ttagccagag ataagtgctg

44461 aaagcatata agtacgaagc tcaccttaag atatttctta aatatacgta aaataatatt

44521 acgcaatagg cttagtttgt aataatttag agattttcag atactaagta ctaattttat

44581 aaataactga atatttaacg tatcttatat ttagcttggt tagcataaaa gtaatgcaat

44641 tgttttgtaa tcaatagaca caagtgcgat acttgtactg agcttaggat tagtagacaa

44701 gtggctaagt catagctttt tcaatgctaa tatcacgggt tcgaatcctg tctagtctaa

44761 tttcaattta agttttaatt tatacctaaa ttagaggcca taacttaatt ggtagagtgt

44821 actgctcata acagtaatta taagtgttcg actcactttg gccttatatc gcgggttagt

44881 gtaacagtaa catttttgac tcatgatcaa aagaaaaagg tgcaaatcct ttatccgcat

44941 taaaatcatt atttttaatc ctagtgatga aattggtaaa cataacaagc ttaaaacttg

45001 taggctaatg ccttaaaggt tcgagtcctt tttaggatat aatagtattt tttttttata

45061 acaataaaat taaaatttat taaaaaaaaa taaataaaat tttataaaac taagatatta

45121 gtgtgatgat cttaaaaata aagtaaatat ttgtatttta actaataaat tttaatataa

45181 aacatctaac ttatacttta taagttgaaa tataaatata aaggttaaaa tatataattc

45241 ttcaaataaa ttataaaaat aaatcgtaaa tatggagagt gctgataata tataagtatt

45301 tatagtaata ctttttatat aaaataaata tatattatag ttattataca taaaaatata

45361 ttttattaac tacaggttaa taaaaatttt agaaagtgcc aaagaaacaa atatttatta

45421 taatctacct aaatagactt tatttgatta tattaaatca aataaattat ttaaaaataa

45481 atattatttt tgttattttt aatttaataa ataatatagc aactgtaatt taatggtaaa

45541 ataatcagtt tccacctgaa atttaagggt tcgattccct ttagttgtac ataagggaat

45601 atagtataat ggtattacag ttaggttgca tctaacttat ttaggttcaa atcctaatat

45661 ttccatgtat taatgttata atagaagggg tctaatgcaa tggtagtatt gtaatttagc

45721 ttattataaa tatatattta aatcttagta cacttataaa gataaaactt ttttttaata

45781 aaaattttta ctgttaaaca ttactttaat atttatcaaa ataaattttt aatttctaat

45841 atataatata gctcagataa ctcaattggc agagtgaaat attgaagcta tttatgttgt

45901 aagttcaaat cttactctga gcatattaaa tttgtatgta aaaaatttta tagtatagtt

45961 cgaatgttgg aattggtaga caaagtaagt ttaggtctta ttgattaaat tcatgaaggt

46021 tcaagtcctt cttcgaataa tataattaat ttattaacaa aaaaattttt ttttatttat

46081 tatctttttt tttttgagta tatttatata tatatatata tgttacattt aaatagtcat

46141 taatattgta tagataaaat attattgaag tattaataaa taaataagta agtaaaatta

46201 tatttaatat aaagaataca ataaaattaa caattttata taaaaaatat tataaattta

46261 atataaaagg ttatatttaa actaaaaata tattaggtat atatcttaat ttggagtaga

46321 ctaatgggta agtcctagat ttttgatatc taaaattgag tgttcgaatc acttctccaa

46381 aaaaaaggag ggtaaaattt attttctgtt ttaattttaa tgctattaat aaaaatagca

46441 tttttagact aaaatatatg attacaatag gataatatag ctcaattggt ggagcgactg

46501 tatgtggcac agtaggttct ctgttcgatt cagagtattt tccctatata gcttgaatag

46561 tttaaaggta aaaccttatt ttcatacgat aaagataggt gttcaattca cctttcaagc

46621 taagaataaa cataaattaa agtttttcaa ataaatataa atttattata tagtgccaga

46681 aaataaaata gcagtatgtt ataacgacgt atttaaataa aataaaaagt tattatttaa

46741 taacacatgt tatataaagc gtataaaatg attaacagaa aaatattata ttataaagtc

46801 aaggtagttt aataggctaa aactttaatt ttatatatta ataatgagaa ttcgattttc

46861 tctcttgatt tacttatgtt aatatatatt tatctatata tatacatata agtaaaaaaa

46921 aaaataatgc ttttaatttc aatattatct ttattacttt ctaatgccgt tactatacga

46981 cgagatatat caatactttt taatagagtg gctattattg ctttagttta ttctatacta

47041 catagtacaa taagtttatt tattataggt aaaggtatag gtttacacgg aggtttacta

47101 aatattacta gtttaacaca aatatttgat atttttatat tcttaattag tatattaata

47161 ttacaattaa caagtttttt ccctagaaaa gtatggatac cagaacattc ttcattaaca

47221 caattattac ttaataattt tatattttat agaacaaaaa ttattaataa aatgggagaa

47281 catttaagaa taatagaata tcctttaata ttattattta taatttgtgg tgcagtattt

47341 ttaatatcta ctggtgattt aatttctgtt ttcctttcta tagagttaca aagttatggt

47401 ttatatttat taagtactat atatagaaat tcagaattat ctactacagg gggattaatg

47461 tatttcttat taggaggttt aagttcatgt tttattttat taggtacaag tttattgtat

47521 gcaaattcag gtactactaa tttagatggt ttatatgcta tcactagtat tagcgatagt

47581 acggatttat gatataaacc ttattatata aatttatctt tacttgtttt tagtatagga

47641 tttttattta aagtaagtgc tgctccattt catttttgat cacctgatgt ttatgatgct

47701 attcctacaa tagttacaac atttgtagca attatagcta aaatatctat attagtattc

47761 tttttagaaa tagtttatta tactaaaaat tattttatag attttaattg aacatatggt

47821 ttattaatta gttctttatt ttcattaata atagggacag ttgcaggttt aactcaattt

47881 agaataaaaa aactttttgc ttatagtact atttctcata taggtttcat actattagct

47941 ttaagtatat ctagtgtaga atctactcaa gcatttatat tctatttaat gcaatattct

48001 attagtaatt taaatgcttt cttaatatta atagctatag gattttcttt atattattat

48061 attagtgata ataaagaata taaagagcta ttagataaag ataattctcc tatacaatta

48121 attagccaat taaaaggtta tttttatata aatcctacat tagcattaag tttagttatt

48181 actatttttt cttttgcagg tatacctcct ttagttggat tttttgctaa acagatggta

48241 ttaagtgctg ctttagataa cggttatata tttttatctt taattgctat acttactagt

48301 gtaataggtg ctgtatatta tttaaatata ataaaagaaa ttttttttta ttcaccacaa

48361 tataaaataa acccattatt agaaaaatta aattttaatg gtagaatata taacaaacaa

48421 aatattttag ttaaatctat tacttttaaa tataataaca ttgcaatatc tagttttctg

48481 tctataacaa tatctataat aacattaata atattattat ttatatttgt aaataaagaa

48541 tgattaagta tgagtaccat attggtacaa ttattattta attattaaat gagtagtatg

48601 agcatatttt ttatatttgt acttgttata gcagtattat ttttagttat taatttagta

48661 tttgcacccc ataatccata tcaagaaaaa tattctattt ttgagtgtgg atttcatagt

48721 tttttaggtc aaaatagaag tcaatttact attaaattct ttatatatgc acttatattt

48781 cttcttctag atttagaaat attattaata tttccttttg gagttagtgg ttatactaac

48841 gatttatacg gattaattat agtattagga tttataacta ttgttacaat aggttttgta

48901 tatgaattag gtaaaaatgc tttaaaaata gatagcaggc aatctataat aaaaaataaa

48961 agtaataata taaaaatttt tactttataa aaataaatat atatatatat atatttattt

49021 aaaattatgt gatattatat ctaaatacta aaataaatga tattcaactc attaatttaa

49081 gatatttttt aataataaat taaaaatggt attagttgca aaaataattg gaacaggatt

49141 agctacaaca ggtttaatag gtgctggtgt tgggattgga atagtatttg gtgctttaat

49201 attaggtgta gctagaaatc catcattaag aggtcaatta ttttcatatg ctatattagg

49261 atttgctttc tctgaagcta caggattatt tgcattaatg atggctttct tattattata

49321 cgttgtttaa ttatataagt aaaaagaaaa aaaacatgat attaacaaaa ttaaactcaa

49381 atcagataaa ttacactaat agaagtaatt tcagtactag tacacgttta tataaaatag

49441 acgctactgg attaacattt tctcagaaat ccattttagc ctctaatatt tcaacaggtc

49501 cagatatacc ttttagatta aatgatttat attctcttgg tagattatta ggtcaagagg

49561 ttcctattac acaaaatgac tttgtaggaa caccagactt aaggtcaact ctaacgatat

49621 cggcaacaag taatgaggct attaatatga ctagtagggt tacacatcta aataattatg

49681 aattattagc gattataaca gctttagcta tagtaggttt tggtgttatt gcgataactg

49741 ctcgtttaag acgacttact cgaatagttg aaaatgcagt tacagaagaa acacctgctt

49801 gagacatacg taatcaaaca aatggtttag aaagagatat tctcgatgaa tttagtttat

49861 taacggatag aatggatgaa gctgctctaa gtttagctcg atatattcaa gtacaagaac

49921 actcaaactt agatatgata cctagaagta ctatagctac tataaatgaa ttgcaacctc

49981 aatggaatga aatgttacga gctttaggtg agaatttagc atcgaataat acaataacct

50041 ttttaggacg agagaatcaa ttatatcttt atcaaaattg aatgcaaata cttactgaaa

50101 gtatgcaaga atttttagtt caatatgaaa tttttttaga ttttttattt ttttaattat

50161 tatttgcttt aattatgata ttaagtttaa tattataata aactttaatg ttattattta

50221 attctgtata atatgaaata aaataattta attattttag actacagtac agaaaattat

50281 acttaaacat aaactatgaa tttaatttta aataatttaa ttatgaaatt agatgcacct

50341 gaagcttgag gtatatattt ccaagatagt gctacacccc aaatggaagg gttagtagaa

50401 ttacatgata atataatgta ttatttagta ataattttat ttgctgtagg gtgaatatta

50461 ttatctgtaa taagaaattt tgttatgagt acttctccta tatctcataa atatttaaat

50521 cacggtacat taatagaatt aatttgaact ataacaccag ctgttatatt aatattaata

50581 gctttcccat catttaaatt attatattta atggatgaag ttagtgatcc ctcaatgtca

50641 attttagcag aaggtcatca atgatattga agttatcaat acccagattt tttagattct

50701 agtgaagaat ttatagaatt tgattcttat attgttccag attctgattt agaagaagga

50761 ggactaagaa tgttagaagt agataataga gtaataatcc ctgaattaac acatattaga

50821 tttattataa catcaggtga tgttatacat tctttttctt gtaattcttt aggtataaaa

50881 tgtgatgctt atcctggaag attaaaccaa gtatctgttt tcgtaaatag agaaggagtt

50941 ttctatggtc aatgctctga aatatgtggt atattacata gttctatgcc tattgttatt

51001 gaatctgttt ctttagaaaa atttttaaca tgacttgaag aacaatagat caataaagaa

51061 ataattatat tatatatatg tatatgtata tataattaaa agtgtgttaa cttaattggt

51121 taagtatagt actacggata ctaaaattat gagttcgaat ctcatacaca tttctcttaa

51181 ccttgtttaa gaaaaccttt ttcaaataat ttatttatct atatatattt attatgaata

51241 taactttaat acttttttta ataggaattt taggatttgt tttaaataga aaaaatatta

51301 tattaatgct tatttctata gaaataatgc ttttatctat aacattctta atattgataa

51361 gttcacttaa tattgacgac ataataggtc aaacatacgc tatttatata ataattgtag

51421 ctggggcaga atcagccata ggtttaggta ttctcgtagc cttttataga ttaagaggaa

51481 gtattgcaat agaatataaa taatgtattt aagtataata attttacctt tattaggatc

51541 tatagtttcc gggttttttg gtagaaaagt tggagttagt ggagcacaaa ttataacatg

51601 ttcaagtata atagttacaa ctatattggc tattattact ttttttgaag taggttttaa

51661 taatattgtt ttatctatca atttatttag atggatagat agtgaatgat ttaacattat

51721 ttgaggtttt caatttgata gtttaactgt atcaatgtta atacctgtat taattataag

51781 ttctttagtt catgtttact ctataggtta tatgaatggt gatccccata atcagagatt

51841 ttttagttat ttaagtttat ttacatttat gatgataata cttgtaacag ctaataatta

51901 tttattaatg ttcgtaggtt gagaaggagt tggtgtttgt tcttatcttt tagtaagttt

51961 ctgatttact agaatagcag caaatcaaag ttctatgtca gcttttttaa ctaatagagt

52021 aggagattgt tttttaacta taggaatgtt tgcaatatta tgatctttag gtaacttaga

52081 ttatagtaca gtattttcat tagcacctta tattaatgaa aatattatta taattatagg

52141 agtttgttta ttaataggtg ctatggctaa aagttcacaa gttggtcttc atgtttgatt

52201 acctatggct atggagggtc ctactcctgt ttctgcttta attcatgcag ctacaatggt

52261 tacagcagga gtatatttat taatgcgttc ttccccgtta attgaatata gttctactgt

52321 tttattatta tgtttatggt taggagcaat aactacagta tttagttcac ttgttggttt

52381 cttccaacaa gatataaaaa aagttatagc ttattctaca atgtctcaat taggaatgat

52441 ggttatagcg gtaggtttat cttcatataa tgttgccttg tttcatttag taaatcatgc

52501 tttttataaa ggattattat ttttaggtgc tggtgctgtt atacatgcaa tggctgataa

52561 tcaagatttt agaaaatatg gaggattaat atctttctta cctctaagtt attctgttat

52621 tttaatagca agtttaagct tagtagcctt ccctttcatg actggatttt atagtaaaga

52681 ttttatatta gaatctgctt atggacaata ttgttttagc agtataactg tgtacattgt

52741 agctgttata ggggcaatat ttactacttt atattcagtt aaagttcttt atttaacttt

52801 tctaactaac cctaatggac ctttaataaa ctataaacat gctcatgaaa gtgatatatt

52861 tatgagttta ccattagtta tattagctat attctctata ttctttggat ttataactaa

52921 agatattttt ataggtttag gttcaggatt ctttatagat aatagtatat ttattcaccc

52981 taactctgaa ataatgatag atacagaatt tggtgtttca acttattgaa aactattacc

53041 ttttattttt actgtgttat ttagtattat agctataatc ttatctgaat ttttctcaga

53101 tcttatagtt aattttaaat tatctacatt aggtagaact atttttgggt tttttaatca

53161 aaggttttta attgaatttt tttacaataa atatattaca aatttagttt taaatttagg

53221 aagtcaaacg gttaaagttt tagataaagg tagtatagaa ttattaggac catacggttt

53281 agaaaaaaat ttaattactt caagtaaaaa tattagtagt ctaaataaag gtattgtaac

53341 caattatgct ttatttattt tagttggatt tatattatat atgttttcta taagtttagg

53401 ttttactaat aatttaagct taataatatt aatattaatg tttagtatta gtttaatctc

53461 aaaaaaaaaa agtaaattta taataaataa ataaatttat aataaataaa tattttagta

53521 catgatacac aataatacct tttatattaa ttaacggtcc ttatgttaaa taaaaaaaaa

53581 ataaaaataa aaaaaaatga gaataataaa aaataatact atattaaaat tagttaatgc

53641 atatcttata gacgcttcac aaccaagtaa tataagttat ttatgaaact ttggttcatt

53701 attagcaatt tgcttaggaa tacaaattat aacaggtgta acattagcta tgcattatag

53761 tcccagtata ttagaagctt ttaactctgt tgaacatatc atgagagatg ttaataatgg

53821 atgattaatt cgttatttac atagtaatac tgcttcagct ttcttctttt tagtatattt

53881 acatatagct agaggtatgt attacggttc atatagatca cctagaacat tagtttgaac

53941 aataggtgta ataatactta tacttatgat agcaatagga ttcctgggtt atgaacttag

54001 cccaaaatgg tttaataaag aattaaaata ccataataat aataataata acaaattaat

54061 atcctttaat aaaagatatt actccacaac atctaatata aataatagta ctaattctaa

54121 gagattagat gagatccttg aagaactaaa attagattat atttatagtt ttgaagattt

54181 aaatttagaa actactaaaa aagaaatttt agataaaact aaaggtttaa gtggtattta

54241 tatgataata aataaaataa ctaaagatta ttatataggt tctgcctcaa ctaatagatt

54301 ttatgctaga tttagtaatc atcttatata ttttagagga agtaaagtag ttaaagcagc

54361 tgtaaataaa tatggtttag aaaactttac ttttattata ttagaattat atcctaatat

54421 aattaacaaa gaaaataata aagaattatt agatttagaa gataaatatt taaaattatt

54481 attacctaat tataatatat taacagaagc aggatctagt tttggttata aacatactga

54541 aattgaccgt caaaaaatga aagatatgta cactgatgaa agacgagaat taatacgtaa

54601 tttaaataaa ggtaaaaagc tttctcctga aacaatagag aaaattagag aaaaagcttt

54661 aaaaatacct attagatcta tagatactag aagtaattat attacacata ctagaccgat

54721 tactttatat aatttagatg gtactattta tggtagttat cctactatta tagaagcggc

54781 taaatctata aattgtggtg aaaaaacact aataagagca ctaaaaacag aaaaaggtct

54841 agtaaaaaga caatgaataa taaaagacac tctaaaataa aattattatt attattatta

54901 tagttattat tttaactatt tattaaatca tagtcccgtg agtaataata tttctgcaat

54961 ttttataaaa aaagaaatat taaagcggta tatctcgacg gagatataga atagtattaa

55021 atactatttg gcgatattag tgaaaacgat caaagaggtt tatacttcaa gatcgtcggt

55081 tatataaatg atcgcgacag actgggtcac taatgggtgg ctgaaatgct gcttaatgca

55141 cagtcggaat ttttaattaa tgtttattaa ttaataaaat atacgaattc aaagttattt

55201 taatttattt tgtagattta cagaataaat aaatttgtac gttcttccat acggtcaaat

55261 gtctttatga ggagcaacag ttattactaa ccttattagc gcaatacctt gaataggaca

55321 agatatagta gagttcattt gaggaggttt ctctgttaat aatgctactt taaatagatt

55381 ttttgcatta cattttgtat taccttttgt attagctgct ttagtactaa tgcatttaat

55441 agcacttcat gatacagcgg ggtcaggtaa tcctttaggg gtagcaggaa cttatgatag

55501 agtacctttt gctccttatt ttttatttaa agatttaatt acaatcttta tttttatatt

55561 tgttttaagc ttctttgttt tttttatgcc aaatatttta ggagatagtg aaaactatat

55621 aatggcaaat ccaatgcaaa cacctcctgc tattgtacca gaatgatatc ttcttccttt

55681 ttatgctata ttaagatcta ttcccaataa attgttagga gttattgcaa tgtttggagc

55741 tttattaata ttacttattt tacctattac agatttaggg agatctagag gtttccaatt

55801 tagaccttta agtaagtttg ttttatactt atttggtgta gactttttat tattagctcg

55861 tttaggtgct gtacatgtag aagacccttt tattcaaatt gggcaaattt gtgcatgtat

55921 ttattttgta ttttttattt tttcattacc tgtagcaagt gttattgaaa atagttttat

55981 ggatattgct tatgtaacag aagaaaataa aatttcaata aaaaataaat taaaataata

56041 attattttta tatgtataaa taaattttca tgacgcacct gatatgtcta ttaagcagct

56101 aaagtattta ttagagatct gttacctgga acattatcag caattatgtt ggcatacgca

56161 aatcatatta tttaagcagc aaatatggct aatattttta tggaaccaac taatgaacat

56221 attatatcta cagtacaagg aatgattgat tttcatgtta gtgtggagaa tacgtttagg

56281 aaaattgata atctactaat actttatttt acggagattt aatttttaat tattaactat

56341 aattatactt gctctaaata tgcttcaggg tactcaagtt gagatagcca ttttagaaaa

56401 ttataatatt caatttacac ttatttgtag tgaattaata taaaatcttg attttttaag

56461 ataacttaca accgattttg taattctaca tccttataat tttgcaatct tttactcaaa

56521 gatagagaat ttagataatc ttatagttaa cctttattta caagtagggg atacatatgc

56581 gaatagttat ttaaaataaa tgaggagttt atatgagaat aactatgcag aaatagtaag

56641 atcattttaa tgatttactt catttctttt aatttatttt tcataattat ttttttaaat

56701 aacattttta aattaaataa tttataatat tattatataa tattaggatt atgaagtaaa

56761 cggtttacac ttaaattgca aatttaaagt ttgaggttcg actcctccgt aatcttatgt

56821 actaaacata atataataga aatttaatat tttcttatta gcttaatggt aaagcaagat

56881 acttctaata tccagatttt agttcgaatc taaaatgaga attttttttt tatttttaat

56941 acataattta atactattta gttatataat ttattattat tattagtttg agtgataaac

57001 ggaaaaatat ttttaattta aaaagtacaa gtaatatatt aaattcatct atatct

//

LOCUS Graphilbum 25567 bp DNA linear 17-JAN-2019

DEFINITION .

ACCESSION

VERSION

KEYWORDS .

SOURCE Unknown.

ORGANISM Unknown.

Unclassified.

REFERENCE 1 (bases 1 to 25567)

AUTHORS Wai, Zubaer, Hausner

TITLE Direct Submission

JOURNAL Submitted (17-JAN-2019) Department of Microbiology, University of

Manitoba, 66 Chancellors Circle, Winnipeg R3T 2N2, Canada

FEATURES Location/Qualifiers

source 1..25567

/organism="Graphilbum fragrans"

/mol_type="genomic DNA"

gene 1..3784

/gene="cox1"

CDS join(1..1011,3311..3784)

/gene="cox1"

/EC_number="1.9.3.1"

/codon_start=1

/transl_table=4

/product="cytochrome c oxidase subunit 1"

/translation="MELSGPGVQYIADNQLYNSIITAHAILMIFFMVMPALIGGFGNF

LLPLLVGGPDMAFPRLNNISFWLLPPSLLLLVFSACIEGGAGTGWTLYPPLSGIQSHS

GPSVDLAIFALHLSGVSSLLGAINFITTIINMRTPGIRLHKLSLFGWSVVITAVLLLL

SLPVLAGGITMVLTDRNFNTSFFETAGGGDPILYQHLFWFFGHPEVYILIIPGFGIIS

TTISANSSKAVFGYIGMVYAMMSIGILGFIVWSHHMYTVGLDVDTRAYFTAATLIIAV

PTGIKIFSWLATCYGGSIKLTPSMLFALGFVFMFTIGGLSGVLLANAALDTAFHDTYY

VVAHFHYVLSMGAVFALFSGWYFWIPKILGLNYNMLLSKVHFWILFIGVNLTFFPQHF

LGLQGMPRRISDYPDAFAGWNLISSFGSIISVVASGLFLYVVYKQLLDNNPVGRFPWM

TPQYFTDVLQALLNRNYPSLEWALTSPPRPHAFVSLPLQSGVKI"

exon 1..1011

/gene="cox1"

/number=1

intron 1012..3310

/gene="cox1"

/note="Group IB"

/number=1

gene 1012..2058

/gene="orf348"

CDS 1012..2058

/gene="orf348"

/codon_start=1

/transl_except=(pos:1012..1014,aa:Lys)

/transl_table=4

/product="hypothetical protein"

/translation="KMGLNYLKSGYEFGYMLEVLLLTYYLLYKFYYYLINIDGKRKHN

FLTLYSQNNNIVDDFYNNIISVEDYSSKPAENCKGFSEALRRISYKKWIPRTFFHRLA

GYIDGGGNFEIKIIDSKLEIIAIKIKVLNRDIRILNFIQNKLHMGRIISDKSKNHSVW

IVTDKTEIKFLVKKLNGLIKLKVENFTKYCEYFNLKYKKANYNIEFKDSYFAGLIDTR

GSIVYNYSQNRIECNLELRNNEYSNKLNLDDTIPNYKPRVLYKKTHDTRLIKYQNVMG

MIYLHNYFLHHKLYSDFKCFRVSKIKNFIEIRHFKSEPKDSILYKKYSEFIINWIQYQ

NPNWFEVSFLKEVR"

gene 2516..3256

/gene="orf246"

CDS 2516..3256

/gene="orf246"

/codon_start=1

/transl_table=4

/product="hypothetical protein"

/translation="MYILCLYISKILFMISILRILIVFNKYWIKNSFIKNNFPIFHFI

IKFVLVILFVLSLCQLIIIMYNLLKYIITKYILKSNTINNLKIKLNNKWKSIKSSLFK

KPKTPKDFNGPILSSDDKESRRKQALELKEQLLEKQKDILKRKYNNSNLTSNSLQGKR

NWNETVEIQEVPDFSLDKQLKNVREEFIAYDKQVDKFKNIVDNIDKGKENFFPDESKT

LFNDYIYMIKLLKNNLKDIENNIKNLKK"

exon 3311..3784

/gene="cox1"

/number=2

gene 3818..3887

/gene="trnN(att)"

tRNA 3818..3887

/gene="trnN(att)"

/product="tRNA-Asn"

/anticodon=(pos:3850..3852,aa:Asn,seq:att)

gene 3969..5039

/gene="nad1"

CDS 3969..5039

/gene="nad1"

/EC_number="1.6.5.3"

/codon_start=1

/transl_table=4

/product="NADH dehydrogenase subunit 1"

/translation="MLYFPTLISILEVVLVLVPALLAVAYVTVTERKTMASMQRRLGP

NAVGYYGLLQAFADALKLLVKEYVAPTQANIILFFLGPIITLIFSLLGYGVIPYGPGL

AISDFSLGILYLLAVSSLSTYGILLAGWSANSKYAFLGSLRSTAQLISYELVISSTIL

LVIFISGNLNLTVNMESQRAVWFILPLLPIFIIFFISSVAETNRAPFDLAEAESELVS

GFMTEHAAVIFVFFFLAEYGSIVLMCILTSILFIGGYLFIGNSIIDLILYFSNTSILI

DYIMNSPILEGLFYGLSLGIKSAFMIFVFIWVRASFPRIRFDQLMAFCWTVLLPIIFA

VIILIPAILYNFILFPININLL"

gene 5155..6618

/gene="nad4"

CDS 5155..6618

/gene="nad4"

/EC_number="1.6.5.3"

/codon_start=1

/transl_table=4

/product="NADH dehydrogenase subunit 4"

/translation="MLLSFLLLIPLIGIFFIAGNISYENNTVNNIYYKNIALVTSIVN

LIVSLIVYMLYDSSNNQYQFVQEHYNLGFFDIYLGIDGISMYFVLLTTIIMPIAILSN

WNSITENIRSYLIIMLLLETLLLAIFLVLDILLFYVFFESTLPPLFLLIGLFGSNNKV

RASFYIFLYTLWGSLFLLLCILSMSSIMGTTDFDALFKTNFDYTTQLFLFAGIFLSFA

VKTPTIFLNNWLLKAHVESPLGGSIVLAAIVLKTSLYGICRLILPILPKASINYTYIV

YVIGVITIIYASFSTLRTTDIKELIAYSSVSHAAVYLIGIFSNTIQGIEGSILLGLAH

GFVSSGLFICAGGILYDRSGTRAIYFYKGIAQVMPLFSILFFILSLGNCGVPLTLNFV

GEFMSLYGVFERLPLLGVLASSSIIFSAAYTIYMFNRIGFGGTFSKFFSENICDVTKR

EFFLLFILVLFTVVLGIYPSFILDGLHYSVTGLIYYS"

gene 6682..6840

/gene="atp8"

CDS 6682..6840

/gene="atp8"

/EC_number="3.6.3.14"

/codon_start=1

/transl_table=4

/product="ATP synthase F0 subunit 8"

/translation="MPQLVPFYFVNEVVFTFAAITIIVYLLSKYILPRFVRLFLSRTF

ISKLFDNK"

gene 6904..7695

/gene="atp6"

CDS 6904..7695

/gene="atp6"

/EC_number="3.6.3.14"

/codon_start=1

/transl_table=4

/product="ATP synthase F0 subunit a"

/translation="MNTLSINTTNLEILSPLSQFDIRDLLSIDAPLLGNLHISLTNIG

FYVTISLFLILALNLLSTNYNKLISNNWSIGQESLYATVHSIVTSQINPRNGQIYFPF

IYTLFIFILINNLVGLVPYSFASTSHFVLTFSLSFTIVLGATILGFQKHGLEFFSLFV

PAGCPLPLLPLLVLIEFISYLARNISLGLRLGANIMSGHMLLNILAGFTYNIMSSSIL

LFFVGLLPLSFIIAFSGLELGIAFIQAQVFIVLTSSYIKDGLDLH"

gene 7731..7897

/gene="rnpB"

gene 7960..9458

/gene="rns"

rRNA 7960..9458

/gene="rns"

/product="small subunit ribosomal RNA"

gene 9498..9582

/gene="trnY(gta)"

tRNA 9498..9582

/gene="trnY(gta)"

/product="tRNA-Tyr"

/anticodon=(pos:9533..9535,aa:Tyr,seq:gta)

gene 9628..9698

/gene="trnN(gtt)"

tRNA 9628..9698

/gene="trnN(gtt)"

/product="tRNA-Asn"

/anticodon=(pos:9660..9662,aa:Asn,seq:gtt)

gene 9771..10580

/gene="cox3"

CDS 9771..10580

/gene="cox3"

/EC_number="1.9.3.1"

/codon_start=1

/transl_table=4

/product="cytochrome c oxidase subunit 3"

/translation="MNNLVRSNFQDHPFHLVSPSPWPLYTSLSLFSLTTSAALSMHNF

ENAYYLVYVGLFLVPTTMFFWFRDIISESTFLGDHTLAVQKGLNLGVILFIVSEALFF

LAIFWAFFHSALTPTVELGAQWPPLGIEPVNPFELPLLNTVLLLSSGATVTWAHHALI

QGDRKGAIYGSIFTVLLAMVFTGFQGVEYSVSSFTISDGAFGSCFFFGTGFHGLHVII

GTIFLLIALWRIYAYHLTDNHHLGFEAGILYWHFVDVVWLFLYISVYYWGS"

gene 10648..10719

/gene="trnK(ttt)"

tRNA 10648..10719

/gene="trnK(ttt)"

/product="tRNA-Lys"

/anticodon=(pos:10680..10682,aa:Lys,seq:ttt)

gene 10754..10826

/gene="trnD(gtc)"

tRNA 10754..10826

/gene="trnD(gtc)"

/product="tRNA-Asp"

/anticodon=(pos:10787..10789,aa:Asp,seq:gtc)

gene 10834..10914

/gene="trnS(gct)"

tRNA 10834..10914

/gene="trnS(gct)"

/product="tRNA-Ser"

/anticodon=(pos:10866..10868,aa:Ser,seq:gct)

gene 10921..10991

/gene="trnW(tca)"

tRNA 10921..10991

/gene="trnW(tca)"

/product="tRNA-Trp"

/anticodon=(pos:10953..10955,aa:Trp,seq:tca)

gene 11061..11720

/gene="nad6"

CDS 11061..11720

/gene="nad6"

/EC_number="1.6.5.3"

/codon_start=1

/transl_table=4

/product="NADH dehydrogenase subunit 6"

/translation="MNNLFIITENFTNGYKNEVLDIISILVILSGIFVIISKNPIISL

LFLIGLFAGIASYLIIIGLSFLGISYLVVYIGAVSILFLFILMLINIRMSELQSNTNN

SIPLAISIAILFNYPLFQLLPYDIAILNNYNNYLNNLLYNVSFNKINTIQETNINLND

NDTLFVTSKVWDNNLAEMGHITSIGNVMYTNYNIWLIITSFILLLAMVGAIVITIKQK

N"

gene 11752..11822

/gene="trnV(tac)"

tRNA 11752..11822

/gene="trnV(tac)"

/product="tRNA-Val"

/anticodon=(pos:11784..11786,aa:Val,seq:tac)

gene 11835..11905

/gene="trnI(gat)"

tRNA 11835..11905

/gene="trnI(gat)"

/product="tRNA-Ile"

/anticodon=(pos:11867..11869,aa:Ile,seq:gat)

gene 11910..11995

/gene="trnS(tga)"

tRNA 11910..11995

/gene="trnS(tga)"

/product="tRNA-Ser"

/anticodon=(pos:11944..11946,aa:Ser,seq:tga)

gene 12035..12106

/gene="trnP(tgg)"

tRNA 12035..12106

/gene="trnP(tgg)"

/product="tRNA-Pro"

/anticodon=(pos:12068..12070,aa:Pro,seq:tgg)

gene 15077..15841

/gene="orf254"

CDS 15077..15841

/gene="orf254"

/codon_start=1

/transl_table=4

/product="hypothetical protein"

/translation="MINNFFYKCLNTSSDLYNTYLKKILYKEFLFLNKWKLKLNLNDL

RFKNKFLMRLNKFISKIYKKKIEFNIINLKSIVHHPDIYTDIMQRKLRKQNTNVLRLI

KTFLNKGHILENNDLKERFRVIKSVNFNLLENKYKNININSIVENTNLNKTIIDSYDT

NDSNNKDIIFESIKYKNLGGIRLELKGRLTRKFKSEKAMFKIKWKGGLKNIDSSFKGL

SSVNYRGNVNSNLEYSLNKSKRRIGAFAIKGWISGK"

gene 16493..16563

/gene="trnT(tgt)"

tRNA 16493..16563

/gene="trnT(tgt)"

/product="tRNA-Thr"

/anticodon=(pos:16525..16527,aa:Thr,seq:tgt)

gene 16578..16650

/gene="trnE(ttc)"

tRNA 16578..16650

/gene="trnE(ttc)"

/product="tRNA-Glu"

/anticodon=(pos:16611..16613,aa:Glu,seq:ttc)

gene 16660..16732

/gene="trnM(cat)"

/note="copy 1"

tRNA 16660..16732

/gene="trnM(cat)"

/product="tRNA-Met"

/anticodon=(pos:16693..16695,aa:Met,seq:cat)

gene 16736..16806

/gene="trnM(cat)"

/note="copy 2"

tRNA 16736..16806

/gene="trnM(cat)"

/product="tRNA-Met"

/anticodon=(pos:16768..16770,aa:Met,seq:cat)

gene 16837..16919

/gene="trnL(taa)"

tRNA 16837..16919

/gene="trnL(taa)"

/product="tRNA-Leu"

/anticodon=(pos:16871..16873,aa:Leu,seq:taa)

gene 16938..17008

/gene="trnG(tcc)"

tRNA 16938..17008

/gene="trnG(tcc)"

/product="tRNA-Gly"

/anticodon=(pos:16970..16972,aa:Gly,seq:tcc)

gene 17013..17085

/gene="trnA(tgc)"

tRNA 17013..17085

/gene="trnA(tgc)"

/product="tRNA-Ala"

/anticodon=(pos:17047..17049,aa:Ala,seq:tgc)

gene 17089..17161

/gene="trnF(gaa)"

tRNA 17089..17161

/gene="trnF(gaa)"

/product="tRNA-Phe"

/anticodon=(pos:17122..17124,aa:Phe,seq:gaa)

gene 17227..17309

/gene="trnL(tag)"

tRNA 17227..17309

/gene="trnL(tag)"

/product="tRNA-Leu"

/anticodon=(pos:17261..17263,aa:Leu,seq:tag)

gene 17352..17424

/gene="trnQ(ttg)"

tRNA 17352..17424

/gene="trnQ(ttg)"

/product="tRNA-Gln"

/anticodon=(pos:17385..17387,aa:Gln,seq:ttg)

gene 17515..17588

/gene="trnH(gtg)"

tRNA 17515..17588

/gene="trnH(gtg)"

/product="tRNA-His"

/anticodon=(pos:17549..17551,aa:His,seq:gtg)

gene 17597..17667

/gene="trnM(cat)"

/note="copy 3"

tRNA 17597..17667

/gene="trnM(cat)"

/product="tRNA-Met"

/anticodon=(pos:17629..17631,aa:Met,seq:cat)

gene 17755..17827

/gene="trnM(cat)"

/note="copy 4"

tRNA 17755..17827

/gene="trnM(cat)"

/product="tRNA-Met"

/anticodon=(pos:17789..17791,aa:Met,seq:cat)

gene 17869..19530

/gene="nad2"

CDS 17869..19530

/gene="nad2"

/EC_number="1.6.5.3"

/codon_start=1

/transl_table=4

/product="NADH dehydrogenase subunit 2"

/translation="MLLVSILSLLLSNAVTIRRDISILFNRVAIIALAYSILHSAISL

FIINKGIGLHGGLLNITNITQIFDIFIFFISILILQLTSFFPRKVWVPEHSSLPQLLF

NNFVFYRTKIINKMGEHLKIIEYPLILLFIISGAVFLISTSDLISVFLSIELQSYGLY

LLSTIYRNSELSTTGGLMYFLLGGLSSCFILLGTALLYANSGTTNLDGLYVITSISDN

LDFWYKPYYINFSLLIFSIGFLFKVSAAPFHFWSPDVYDAIPTIVTTFVAIIAKISIF

VFLLEIVYYTRNYFTDFNWTYGLLISSLFSLIIGTVVGLTQFRIKRLFAYSTISHVGF

ILLALSISSIESTQAFIFYLMQYSISNLNAFIILITIGFSLYYYTSDNKEYKELLDKN

NSPIQLISQLRGYFYINPTLSLSLVITIFSFAGIPPLVGFFAKQMVLSAAIDNGHIFL

SLVAILTSVIGAVYYLNIIKEIFFFSPDYKLNPLLKDLKFSGNIYNKQNVLVKSITFK

YNNIVISSPIAITISVITMIILLFIFANKEWLSMSTIVVQFLFNY"

gene 19531..19932

/gene="nad3"

CDS 19531..19932

/gene="nad3"

/EC_number="1.6.5.3"

/codon_start=1

/transl_table=4

/product="NADH dehydrogenase subunit 3"

/translation="MSSMSIFFIFVMIIAALFLVINLLFAPHNPYQEKYSIFECGFHS

FLGQNRSQFTIKFFIYALIFLLLDLEILLIFPFGVSGYANDLYGLIIVLIFTTIVTIG

FVFELGKNALKIDSRQALIKNKNNNIKIFTL"

gene 20037..20261

/gene="atp9"

CDS 20037..20261

/gene="atp9"

/EC_number="3.6.3.14"

/codon_start=1

/transl_table=4

/product="ATP synthase F0 subunit c"

/translation="MVQVAKIIGTGIATTGLIGAGVGIGVVFGALILGVARNPSLRGQ

LFAYAILGFAFAEATGLFALMMAFLLLYVA"

gene 20503..21255

/gene="cox2"

CDS 20503..21255

/gene="cox2"

/EC_number="1.9.3.1"

/codon_start=1

/transl_table=4

/product="cytochrome c oxidase subunit 2"

/translation="MNLLLNNLIFKLDAPEAWGIYFQDSATPQMEGLIELHDNIMYYL

VIILFSVGWVLISIIRNFVSTTNPISHKYLNHGTLIELIWTITPAVILILIAFPSFKL

LYLMDEVSDPSMSILAEGHQWYWSYQYPDFLDSSDEFIEFDSYIIPESDLEEGALRML

EVDNRVIIPELTHIRFIITSGDVIHSFSCNSLGMKCDAYPGRLNQLSVFVNREGVFYG

QCSEICGILHSSMPIVIESVSLEKFLTWLEEQ"

gene 21299..21371

/gene="trnR(acg)"

tRNA 21299..21371

/gene="trnR(acg)"

/product="tRNA-Arg"

/anticodon=(pos:21332..21334,aa:Arg,seq:acg)

gene 21434..21703

/gene="nad4L"

CDS 21434..21703

/gene="nad4L"

/EC_number="1.6.5.3"

/codon_start=1

/transl_table=4

/product="NADH dehydrogenase subunit 4L"

/translation="MNITLILFLIGILGFVLNRKNIILMLISIEIMLLSITFLILVSS

LNIDDIIGQTYAVYIIIVAGAESAIGLGILVAFYRLRGSIAIEYK"

gene 21703..23799

/gene="nad5"

CDS 21703..23799

/gene="nad5"

/EC_number="1.6.5.3"

/codon_start=1

/transl_table=4

/product="NADH dehydrogenase subunit 5"

/translation="MYLSIIVLPLLGSIISGFFGRKVGVTGAQIITCSCILLTTTLAI

ITFFEVGFNNIVLSINLFRWIDSEWFNIIWGFQFDSLTVSMLIPVLIISSLVHIYSIG

YMSADPHNQRFFSYLSLFTFMMIILVTANNYLLMFVGWEGVGVCSYLLVSFWFTRIAA

NQSSMSAFLTNRVGDCFFTIGMFAILWSLGNLDYSTVFSLAPYINENIVIIIGVCLLI

GAMAKSSQVGLHVWLPMAMEGPTPVSALIHAATMVTAGVYLLMRSSPLIESSQVGLHI

WLPMAMEGPTPVSALIHAATMVTAGVYLLMRSSPLIEYSSTVLLLCLWLGAITTVFSS

LVGFFQQDIKKVIAYSTMSQLGMMVIAVGLSSYNVALFHLVNHAFYKGLLFLGAGAVI

HSMADNQDFRKYGGLISFLPLSYSVILIASLSLVAFPFMTGFYSKDFILESAYGQYSF

SSISVYVIAVIGAIFTTLYSVKVLYLTFLANPNGPLINYKHAHESDIFMSLPLVVLAI

FSIFFGYITKDIFIGLGSGFFIDNSIFIHPNSEIMIDTEFGVSTYWKLLPFVFTVSFS

IIAIILSEFLSELVINFKLSTLGRTIFGFFNQRFLVEYFYNKYITNLILNIGGQTVKV

LDKGSIELLGPYGLEKKLISSSKNISSLNKGIVTNYALFILVGFIVYMFSISLGFTNN

LSILILILMFIISSLY"

gene 23944..25101

/gene="cob"

CDS 23944..25101

/gene="cob"

/EC_number="1.10.2.2"

/codon_start=1

/transl_table=4

/product="apocytochrome b"

/translation="MRILKNNGLLKLVNSYLIDASQPSNISYLWNFGSLLALCLIIQI

ITGVTLAMHYSPSVLEAFNSVEHIMRDVNNGWLIRYLHSNTASAFFFLVYLHIARGMY

YGSYKSPRTVAWSIGVVILILMIGTGFLGYVLPYGQMSLWGATVITNLISAIPWIGQD

VVEFIWGGFSVNNATLNRFFALHFVLPFVLAALVLMHLIALHDNAGSGNPTGLAGTYD

RVPFAPYFLFKDLITIFIFFFVLSVFVFFMPNFLGDSDNYIMANPMQTPAAIVPEWYL

LPFYAILRSIPNKLLGVITMFGALAILLVLPKTDLGRSRGFTFRPLSKVVIFMFGAVF

LILLNLGQAHVEDPFIVLGQVFAVLYFSYFLVILPVVSVCENSLMDISHND"

gene 25152..25222

/gene="trnC(gca)"

tRNA 25152..25222

/gene="trnC(gca)"

/product="tRNA-Cys"

/anticodon=(pos:25184..25186,aa:Cys,seq:gca)

gene 25261..25331

/gene="trnR(tct)"

tRNA 25261..25331

/gene="trnR(tct)"

/product="tRNA-Arg"

/anticodon=(pos:25293..25295,aa:Arg,seq:tct)

ORIGIN

1 atggaactta gcggaccagg tgttcaatat atcgcagata atcaattata caatagcata

61 ataacagctc acgctatatt aatgatattc tttatggtta tgcctgcttt aataggtggt

121 ttcggtaatt tcttattacc tttattagta ggtggacctg atatggcatt ccctagatta

181 aataatataa gtttctgatt attacctcct agtttattat tattagtttt ctctgcatgt

241 atagaaggtg gagcgggtac aggatgaact ctttaccctc cattatctgg aatacaaagt

301 catagtggac ctagtgtaga tttagctatt tttgctcttc atttatcagg agtaagtagt

361 ttattagggg ctattaattt cataacaact ataataaata tgagaactcc aggtattaga

421 ttacataaat tatctttatt tggatgatct gttgttatta cagctgtttt attattatta

481 tctttacctg ttttagctgg aggtattaca atggttttaa cagatagaaa cttcaatact

541 tctttctttg aaactgctgg aggaggtgat cctatattat accaacactt attctgattc

601 ttcgggcacc ctgaagttta tattttaatt atcccaggat ttggaataat tagtacaact

661 atttctgcta actctagtaa agcagtattt ggttatatag gtatggttta tgctatgatg

721 tctataggaa tattaggatt catagtatga agtcatcaca tgtatacagt tggtttagat

781 gttgatacaa gagcttattt cacagctgct actttaatta ttgctgtacc aacaggtatt

841 aaaatattct cttgattagc tacttgttac ggaggttcta taaaactaac tccttcaatg

901 ctatttgcat taggatttgt atttatgttt acaataggag gattaagtgg agttctttta

961 gctaatgctg cattagatac agcattccac gatacttatt acgtagttgc taagatgggt

1021 cttaactact taaaaagtgg ttatgaattt ggctatatgc tggaagttct gctactgacg

1081 tattatttac tatataaatt ttattattat cttataaata tagatggaaa aagaaaacat

1141 aattttctta ctttatatag tcaaaacaat aatattgttg atgatttcta caataatata

1201 attagtgtag aagactatag tagtaaacca gcagaaaact gtaaaggatt ctcagaggct

1261 ctacgccgaa tatcttacaa aaaatgaata ccaagaacat tttttcatag actagcaggt

1321 tatatagatg gtggtggtaa ttttgaaatt aaaattattg attctaaatt agaaataata

1381 gctataaaaa ttaaagttct taatagagat attagaattt taaattttat tcagaataaa

1441 ttacatatgg gtagaattat atctgataaa agtaaaaacc attcagtatg aatagtaact

1501 gataaaactg aaataaagtt tttagttaaa aaattaaatg gtttaatcaa acttaaagta

1561 gaaaatttta ctaaatattg tgaatatttt aatttaaaat ataaaaaagc taattataat

1621 attgaattta aagattctta ttttgcagga ttaattgata caagaggtag tattgtttac

1681 aactactctc aaaatagaat agaatgcaat ttagagttaa gaaataatga atattctaat

1741 aagcttaatt tggatgatac catacctaat tataaaccta gagttttata taaaaaaact

1801 catgacacta gacttataaa atatcaaaat gtaatgggaa tgatatattt acataattat

1861 tttttacatc ataaactata ctcagatttt aaatgtttta gagtatcaaa aattaaaaat

1921 tttatagaaa taagacattt taaatctgaa cctaaagata gtatattata taaaaaatat

1981 tctgagttca taataaattg aattcaatat caaaatccta attgatttga agtatctttt

2041 ttaaaagaag taagataaag atcgagtccg taccctttcg agggattgtt caattaatct

2101 ataatttaat ttaattagac attaattgta cagagaatgc atatttattt tggatataat

2161 atatatatat attatatcta agatatttgt actttttata ataataccct ggacctggtt

2221 attataaaaa tattcttgtt tttagagtat aaattaataa tatagtaagg ttaactatac

2281 tatactatat cttataacta tgcatttctt caggttcaaa catatataac agcatttaaa

2341 cgctggaata tgcttaatta tattttactg atctctaata tctaaaaata aaattttaga

2401 cgataaatta gacaaatttt tagataattt gtttactaaa ttttatatta atactaatat

2461 tataaaccca aaatgattgt ttattatatc ttctgtggct ttttattgag gcgaaatgta

2521 tatactctgt ttatatatat ctaaaattct ttttatgatt agtattttac gtattttaat

2581 tgtttttaat aaatactgaa ttaaaaacag ttttataaaa aataattttc ctatatttca

2641 ttttattatt aagtttgttt tagttatatt atttgtatta agtttatgtc aattaataat

2701 aatcatgtac aacttattaa aatatattat aactaaatat attttaaaaa gtaatacaat

2761 taataattta aaaattaaat taaataataa atgaaaaagt ataaaatcat ctttattcaa

2821 aaagccaaag actcctaaag actttaatgg tccgatttta tcttcggatg ataaagaaag

2881 tagaagaaaa caagctttag aattaaaaga acaattatta gagaaacaaa aagatatttt

2941 aaaaagaaaa tataataact ctaatttaac ttctaattct ttacaaggaa aaagaaattg

3001 aaacgaaact gttgaaatac aggaagttcc tgatttttcc ttagataaac aattgaaaaa

3061 tgttagagag gaatttatag catatgataa acaagtagat aagtttaaaa atattgtaga

3121 taatattgat aaaggtaaag aaaatttttt tcctgatgaa tctaaaacct tgtttaatga

3181 ttatatttat atgattaaat tattaaaaaa taatttaaaa gatatagaaa acaatataaa

3241 aaatttaaaa aaataagttt tagtgaaaac agaatataaa ataatttttt atataatgag

3301 gatgtttcag catttccact atgtactaag tatgggagct gtgtttgctt tatttagcgg

3361 atgatacttc tgaataccta aaatactagg tttaaattat aatatgttac tatctaaagt

3421 tcatttctga attttattta ttggggttaa tcttacattc ttccctcaac atttcctagg

3481 attacaaggt atgcctcgta gaataagcga ttaccctgac gcttttgcag gatgaaattt

3541 aattagtagt tttggttcta taataagcgt agtagcatca ggattattct tatacgttgt

3601 ttacaaacaa ttattagata ataatcctgt aggaagattc ccatgaatga ctcctcaata

3661 cttcactgat gtattacaag ctctattaaa tagaaattat cctagtttag aatgagcttt

3721 aactagccca cctagaccac atgcatttgt aagtttacct ttacaatcag gtgtaaaaat

3781 ataatagttt aattatctat agtgtaatta tactatatta ttattagctt aacggtaaag

3841 caaaatataa ttaatattta attctagttc gaatctagaa taataataaa aacatatttt

3901 attatgatta taataaaaat tattattatt atcaattttt ataaaataaa aaaaaaataa

3961 actttttaat gttatatttt cccactttaa tttctatttt agaagttgtt ttagtattag

4021 ttcctgcttt attagctgta gcatatgtaa cagtaacaga aagaaaaaca atggctagta

4081 tgcaaagaag attaggtcca aatgctgtag ggtactatgg attattacaa gcatttgcag

4141 atgcattaaa acttcttgta aaagaatatg tagcacctac tcaagctaat attattttat

4201 ttttcttagg tcctattata actttaatat tctcattatt aggatatggt gttatacctt

4261 atggaccagg gttagccatt agtgatttta gtttaggtat attatattta ttagcagttt

4321 catctttatc tacatatgga atattattag caggatgaag tgctaatagt aaatatgctt

4381 tcttaggttc tttaagaagt acagcacaat taattagtta tgaattagtt ataagttcta

4441 ctatattatt agtaatattt atatcaggaa atttaaattt aactgttaat atggaatcac

4501 aaagagcagt atgatttata ttacctttat tacctatatt tataatattc tttataagtt

4561 ctgtagctga aactaataga gctccatttg atttagcaga agccgaatca gagcttgtta

4621 gtggatttat gacagaacac gccgcagtta tttttgtgtt cttcttctta gctgaatacg

4681 gtagtattgt attaatgtgt atactaacaa gtatattatt tataggtgga tacttattta

4741 ttggtaattc tattatagat ttaattcttt atttttcaaa cacaagtata cttattgatt

4801 atataatgaa tagtcctata ttagaaggat tattttatgg tttaagctta ggaataaaaa

4861 gtgctttcat gatatttgta tttatttgag taagagcttc tttccctaga atacgttttg

4921 accaattaat ggctttctgt tgaacagttt tattacctat aatcttcgca gttattatat

4981 taatacctgc aatattatat aatttcatat tattccctat caatattaat ttactttaat

5041 aaatttaaat atttatttat atttattacc ataataatta tatatatatg gtgtatattt

5101 ttacagacaa tttaatattt taggttaact tataaactac aacaataaaa aagaatgtta

5161 ttatcttttt tattactaat acctttaata ggtattttct ttatagctgg taatatttct

5221 tatgaaaata atacagtaaa taacatatat tacaaaaata tagcattagt tacttcaata

5281 gtaaatttaa tagtatcttt aattgtatat atgttatatg attcaagtaa taaccagtat

5341 caatttgtac aagaacatta taatttaggt tttttcgata tttatttagg tatagatggg

5401 atatctatgt attttgtatt attaacaaca ataattatgc ctatagctat tttatctaat

5461 tgaaattcaa taacagaaaa tataagatca tatttaataa taatgttatt attagaaaca

5521 ttattattag ctatattttt agttttagat attttattat tctacgtatt ctttgaaagc

5581 acattacctc cattattctt attaataggt ttattcggat ctaataataa agtaagagct

5641 agtttttata tattcttata tacattatga ggttcattat tcttattatt atgtatttta

5701 agtatgtctt ctataatggg tactacagat tttgatgcct tatttaaaac taattttgat

5761 tatactactc aattattttt atttgcagga atattcttat cttttgcggt aaaaactcct

5821 acaatttttt taaacaattg attattaaaa gctcacgttg aatctccatt aggtggtagt

5881 atagtattag ccgctattgt attaaaaact agtttatatg gtatatgtag attaatttta

5941 cctatattac ctaaagcttc aataaattat acttatatag tttacgtaat aggagtaata

6001 acaataatat atgctagttt tagtacatta agaactactg atattaaaga attaatagct

6061 tatagttctg tatctcacgc tgccgtatac ttaataggaa tattcagtaa tacaatacaa

6121 ggtatagaag gaagtatact tttaggttta gcacacggat ttgtatcaag tggtttattt

6181 atatgtgcag gaggtatatt atacgataga tcaggaacta gagctattta tttctacaaa

6241 ggtatagctc aagttatgcc tctattctct atactattct tcatattatc tttaggtaat

6301 tgtggtgtac ctttaacatt aaattttgta ggagaattta tgtccctata cggagtattt

6361 gaaagattac ctttattagg tgtattagct agttcttcta tcatattctc agctgcatat

6421 acaatttata tgtttaatag aataggattt ggaggtacat ttagcaaatt cttctcagaa

6481 aatatttgtg atgtaacaaa aagagaattc tttttattat tcatattagt attattcaca

6541 gtagtattag gaatatatcc ttcatttatt ttagatggtt tacattattc tgttacaggt

6601 ttaatctact attcatagaa aatctttcat taaaaaatta taattaatat ttaatattta

6661 attaaaaaat acatatatat aatgcctcaa ttagtacctt tttattttgt taatgaagta

6721 gtatttactt ttgctgctat tactataatc gtttacttac tatcaaaata cattttacca

6781 agatttgtac gtttattctt atcacgtaca tttatatcaa aactttttga taataaataa

6841 attatataca atctaaatat tgtataagga tttttcaaga catataatat agcttaaaga

6901 actatgaata ctttaagcat aaacacaaca aatttagaaa ttcttagccc cttaagtcaa

6961 tttgatataa gagacttatt aagtatagac gcccctttat taggaaattt acacatatct

7021 ttaacaaata taggattcta tgttacaatt agtttatttt taatattagc cttaaattta

7081 ttaagtacaa attacaataa attaataagt aataactgat ctataggtca agaatcttta

7141 tatgctactg ttcacagtat agttacaagt caaataaacc ctagaaatgg tcaaatttat

7201 ttcccattta tttacacatt atttatattt atattaataa ataatttagt aggattagtg

7261 ccttacagtt ttgcttcaac tagccacttt gtgttaacat tctctcttag tttcacaata

7321 gttttaggtg ctacaatatt aggattccaa aaacacggat tagaattctt ttctcttttc

7381 gtaccagctg gttgtccttt acctttatta cctttattag ttttaataga atttatttca

7441 tacttagcta gaaatatttc attaggatta agattaggag ctaatattat gagtggtcac

7501 atgctactta atatcttagc aggatttaca tataacataa tgtcttcaag tatattatta

7561 ttctttgtag gattattacc attatcattt attatagctt tctcaggatt agagttaggt

7621 atagcattta tacaagctca agttttcata gttttaactt catcttatat taaagacgga

7681 ttagatcttc attaaaacta aaaaaaaaac caaaatattt agtgatataa taggaaagtc

7741 tacggatata ttaattttat aaataaatta taagtattaa aaagaaaata aaaataaata

7801 aatagataat taattattaa ataacaaatt tattatgcga accttcaagc ttataaatat

7861 atcttttgtt aagtaagttt cacaagaact agcttattat tatcctaaat aaaaaaatta

7921 taaactttta agtaatgtaa ttcattacga attacattaa ataagacgag ttcgatgatg

7981 gctctgaatg aacactgtcc atatgcttga cacatgctaa tcgtacgatt aatttaatga

8041 ataattttat taatagtggt gaacaggtga gtatttgata atcaaactac cttaaagtaa

8101 ggggaaaaat cccttatatt aacaaaggaa aattctgctt taagatgact cgatatatca

8161 tagagatagg tagttgttaa ggtaatgact taacaagccc ttgattctct tagtcgtaat

8221 tgaaatagtt gatcgaccac attggggatg aaaaaatccc aaaacaacca gtgtacagca

8281 gtggggaata ttggtcaatg gcctaacggc tgaactggca atttggaaga atagcctaat

8341 ttttttattt aaatttataa aaaaatatag tgaaaaggct aaatcgttta tcgaataaaa

8401 ttcgaaatat atattactat aatgattata tatatttatg tcttgactaa tcacgtgcca

8461 gcagtcgcgg taatacgtga gagactagtg ttatttatta ttaataggtt taaagggtac

8521 tcaagctgaa gagatttcct tgtcaatggg actatctcta tctagagttt aatataagaa

8581 ggtcgtactc gaggagtaaa gattaaattt tttgatacct ataagaggac tggttaaggc

8641 gaaagcaacc ttttatgtaa aaactgacgc tgaagtacga aggcttaggt cacgaatagg

8701 attagatacc ctagtagtct atgcagagac tcctaaatgc tgtaggttag gttaacactt

8761 agtctaagat tgaaaaaata agcatttcac ctcaagagta acgtggcaac gcgggaactg

8821 aaatcattag accgtttctg acaccagtag tgaagtatgt tgtttaattc gatggtcctc

8881 gaaaaacctt accacaacta gaattgtatt aaaaatacat gtatttttaa tataacgagt

8941 gttgcacggc tgtttacagt taatgttgcg agattttggc ttcggtcatt aaattaacga

9001 aaacccttgc tttatttata atattttcat tataaagcag tttttatcat tataccgata

9061 aaataatagg gcaaaagaca agtcatcatg gcctttatgt tgtgggctat agacgtgcca

9121 catataccta gacaaagaga tgctaaattg agaaattaag ctaatctata aaatagggta

9181 aaaaggataa ggattgtagt ctgaaactcg actatatgaa taagtaatta ctagtaatcg

9241 tgaatcacca cgtcacggtg aatttaacct cggattggta ctaaccactc gtcgcatgct

9301 gaaaggagtt cgtgcagtaa gtttgctttt attattataa gtaagtaaat taattggatt

9361 tagattttat aataaatttc ttcgtatgtt cggctctgat tagtgttaag tcgaaatacg

9421 gttcgtgtag tggaagttgc acgggattta ttaattttaa ctttattagt tatgtataat

9481 ttttatatta tacattaagg agggttcctt tattggtaag aagggttgag ctgtaaactc

9541 aatagctaat ttgctttaag agttcgaatc tcttgtctcc tatataatta atatatccat

9601 atataaatat acatatattt atattaacct tctatagctc aatggtagag cataatactg

9661 ttaatattag gataaatgtt cgattcattt tagaagggag taatatgtat attctgacta

9721 ttacaattct gtatatatta aattaactat taactttaac tttataataa atgaataatt

9781 tagtgagaag taattttcaa gatcatccat ttcatttagt ttctccctca ccttgaccac

9841 tttatactag tttatcttta tttagtctaa ctacaagtgc tgcattatca atgcataatt

9901 ttgaaaatgc atactactta gtttatgttg gtctattctt agtacctaca actatgttct

9961 tttgattccg tgatattatt agtgaaagta ctttcttagg agatcatact ttagctgtac

10021 aaaaaggatt aaacttaggt gttatattat ttatagtatc tgaagcatta ttcttcttag

10081 ctatattctg agctttcttt catagtgctt taacaccaac tgttgaatta ggtgcacaat

10141 gacctccttt aggaatagaa cctgttaatc cttttgaatt accattactt aatacagttt

10201 tattattatc tagtggagct actgtaacat gagcacatca tgctttaata caaggagata

10261 gaaaaggagc tatatatggt tctatattta cagtattatt agctatggtt ttcactggat

10321 tccaaggtgt tgaatacagt gtatcatctt tcactattag tgacggtgct tttggttcat

10381 gtttcttctt tggaacagga ttccatggat tacacgttat aataggtaca atattccttc

10441 ttattgcctt atgaagaatt tatgcatatc atttaacaga taatcatcat cttggatttg

10501 aagctggaat attatactga cattttgtag acgttgtttg attattctta tatatctcag

10561 tttactactg aggatcataa atattcaaat taaaatattt atattttaat aataatattt

10621 aaatatatta attaatatat ttaaataggg atctttgtat aatggtaata catgtgactt

10681 ttaatcatta aattgttggt tcgaatccga cagatcctat aatttaaata tattgttcca

10741 tttttcttgt taagaattaa taacttaatt ggtaaaggac ttctttgtca cagaagtaga

10801 tatcggttcg actccgattt aattcgatta agaggaaaaa tttccattgg tagggtaact

10861 taattgctat ttaaggtcta tttagacttg gaagttcgag tcttcctttt tccgatatat

10921 aagagtataa tttaatggta aaatattgag cttcaacctc aagcttctct gttcgaatca

10981 gagtactctt gaattaaata cataataaat attttatgta aattcctatt aatttaggta

11041 ctaaatattt agaattaaag atgaataatt tatttataat aacagaaaat tttacaaatg

11101 gttataaaaa tgaagtatta gatataattt ctatattagt aatattatca ggtatatttg

11161 ttattattag taaaaatcct ataatatctt tattattttt aataggatta tttgcaggaa

11221 tagcttcata tttaataata ataggattaa gttttttagg gatatcttac ttagttgttt

11281 acattggggc agtttctatt ttgtttttat tcatattaat gttaattaat attagaatga

11341 gtgaattaca aagtaatact aataatagta tacctttagc aattagtatt gcaatacttt

11401 ttaattaccc attattccaa ttattacctt atgatatagc tattttaaat aattataata

11461 attatttaaa taatttatta tataacgtat catttaataa aattaatact atacaagaaa

11521 ctaatattaa tttaaatgat aatgatactt tatttgttac aagtaaagta tgagataata

11581 atttagcaga gatgggtcat ataacaagta taggtaatgt tatgtatact aattataata

11641 tatgattaat aataactagt tttatattat tattagcaat ggtaggtgct atagttataa

11701 ctataaaaca aaaaaattaa tataaatata tacatatata tatatttata aaagggatta

11761 acttaatggt agagtaattg ttttacacac aataagcata ggttcgattc ctatattcct

11821 tattatatgc ctatagttcc ttaacttaat ggtaaagtgt acttttgata aaagtattat

11881 cagcgttcaa ttcgctgagg aattatataa gatgattggc tgagtggttt aaagcgactg

11941 tcttgagtac agttaaggag tatatccttc aggagttcga atctcttatc ttctgtaaaa

12001 aatatataat taaaataaat ttaactaatt aaaacagatc ctagtttaat tagtaaaacg

12061 tattctttgg gagaatataa tctgagtgcg attctcgggg atctgaaaat ctataattaa

12121 ttataaactt ataagtaata taattgatac gaaaaaaata tttatacaat ataattatat

12181 attgtgagta tataaagaga ctattaggta gcatagctat gtattaaaga gatatatatt

12241 tattctaatt aattaagaat aataaactct aagagaaatc agatactaaa cgaagtgaat

12301 tgaattatct tattagcttc aggaaaagaa atcaaaagag attctatgat tagcgagagc

12361 aaaaatagaa aagtttaatt attaagtaaa atggtcacaa aactgtttga acataatggg

12421 gaaccttcct caaaaactaa atataataca taagcgagta gcgaaaagta ccgtgaggga

12481 agcagtgtga agaattagta gttttataag cagctcaagc taaagcgaga gcgtaccttt

12541 tgcataatgg gtcaccaagt taaatttagg tgcgagctaa cgcgtagtta aaccgatcat

12601 aaaaataatg aatagtatct aaatttagac ccgaagtaca gtgatcttac catagtcagg

12661 actataaagg tccgaacggg ttatcgttgt aaagatatcc gaagaactat ggtaagtata

12721 gtgaaagaca atccgtgact gtattagctg gttttctacg aaacctataa tagtaggcaa

12781 tttaaattat atctttacag gtacagaact taatctcaga caagatgtat gtaaaaatac

12841 attttatttt gtatagatcg ggggatcatg aagattttac cggtgagttt gtggactcga

12901 aatggtaaag ataaatttta aatcatcaga catagaatga taaggttgta tgtcaaaagg

12961 gaaacagccc agaacaagag ttaaggttcc aaaattatta ttaagtgaaa ttaagaaagt

13021 ttttttattc gtcgataagg aggtaggctt agaagcagcc ataatttaaa gatctcgtat

13081 cagagcactt attaaatgta aaaagcgtcg aaaatttaac ggatctaaat aatataccga

13141 taccttgtcc ataaaatata aatatttatt tatttttatg gggtagtaga acgttgagtt

13201 aatataagca acttttttta taaaaatttt tataatataa ctcaagtgag aatggtgaca

13261 tgagtaacga aaaagaataa ctcgcctaaa gcttatggta gaatttaagt aacggcctct

13321 aagtttataa cctgaaggtt aaaacgatga gaaaatctta ttactaataa agacgacatt

13381 ttcaagtgaa aagtgtacag gaaataaata gtaatattgt atatttaata tacaaaaacc

13441 gttcctaggt actaaaacaa gtaagctagt agagaatacg aaggcgcatg agataacaat

13501 cttaaaggaa ctcggcaaat tgactctgta acttagggaa aaggagtgct cattagtcct

13561 gattaatatc aggtaaaaag gaagaggcat ggaataatgt tgtacgactg tttaattaaa

13621 acaaagcact ttgcaaaaga tgataaatca aagtattgag tgtgatgtct gcccgatgtc

13681 ggctggttaa cgaaatctac taaacattat aagtttggtt tttaaggaac ccccgatcaa

13741 tggcggtctt aacgtgagga tcctaaggta gcgaaatgcc ttggccgtta aatgcggtct

13801 tgcatgaatg atgtaacgat acaacagctg tctctaagat tgactcagtg aaattggagt

13861 aactgtgcag atacagttta cccctaggta gacgagaaga ccctatgcag ctttactgtt

13921 actaattatt ggatataatt caagcaaatt ttagtttaga aggttcacag attatgtaaa

13981 agtgaaagac ctttatttgt ttattatatt ataaacctta aattttaagg aacagttgtt

14041 agaagacagt ttatgtgggg cacagacccc gtaaagagta aacgggtgta tctaaaatta

14101 taacttatat tatataagtt tatttatatt tatttgtttg taattaactt taacaatgtt

14161 aaagttaatc ttataattta gtgtataatt atatacatgt tattaggtaa gatttttcct

14221 atagagaaat tagttgtatt taaaatatta taaaatctat aatatttatt agttattatg

14281 atgaataatc attaataatc atgattaata cttataaagt ttaatggctt aatcttgctt

14341 tactgtttga ttcactacaa atcttacagt cgcgtaagcg gagcattaga tcacaagata

14401 caaaaaggaa aggtcttgga ttattggaaa agctacgcta gggatgtttg tccttcaata

14461 ttttttaata attgataaca tattgggtta atttcaagaa ttacttataa aattaagtaa

14521 atttgaagcg aaatttataa aaaaataaaa tcgttcaacg actaaaggtg agttacataa

14581 caataatcct ttattaatgc ccaacatttt tcttttatta tataaagttt atataataaa

14641 aataaaataa ttgaaaaaag atacaaaatt attaaacaat aatatattta taaaaaatat

14701 taataataaa tctaaattaa tacctttaaa tattataaac aattatgtag gggaagctaa

14761 atacttccct tccgaatata aagaatgaaa taataatgct tattatttta attctaataa

14821 tataaaaaat tttccagttt ataatattaa tttaaataaa ttaataaaat tttattttga

14881 tttttactta aatcctttaa aagttaaatc taattttatt aataaaagaa aaagttattt

14941 ttctttaaat aaaatctttg taagtaaagc tgaattaaaa catactaatt ctaaagttat

15001 aataactgtt tatgtttata atagagaaag aattatttta ttaaataaaa ttaataaaat

15061 aaaaaattct ttatttatga taaataactt cttttataaa tgcttgaaca cttcatcaga

15121 tttatataat acttatttaa aaaaaatttt atataaagaa tttttatttt taaataaatg

15181 aaaattaaaa ttaaacctta acgatttaag atttaaaaat aaatttttaa tgagattaaa

15241 taaatttata agtaaaattt ataaaaaaaa aatagaattt aatattatta atctaaaatc

15301 tattgtacat catcctgata tttatacaga tataatgcaa agaaaattaa gaaaacaaaa

15361 cactaatgtt ttaagattaa taaaaacttt tttaaataaa ggtcatatat tagaaaataa

15421 cgatttaaaa gaaagattta gagtaattaa aagtgtaaat tttaatttat tagaaaataa

15481 atataaaaat attaatataa attcaatagt tgaaaatact aatttgaata aaacaataat

15541 agattcatat gatactaatg atagtaataa caaagatatt atatttgaat ctataaaata

15601 taaaaattta ggtggtataa gattagaact taaaggaaga ttaactagaa aatttaaatc

15661 agaaaaagct atgtttaaaa taaaatgaaa aggaggttta aaaaacatag attcttcatt

15721 taaaggttta tcttctgtaa attacagagg aaatgttaat tctaatttag aatattcatt

15781 aaataaatct aaacgtcgta ttggagcttt tgctataaaa ggttgaatta gtggtaaata

15841 gtaattatta gtatttaaat ataataaaag aaaaatgaag aaatagtctg aaccattttg

15901 tgaaagatgg aaataatata tttatgataa caagttgaac aggctaattt gcgcaagagt

15961 gtacaaaatg agtgcgcggt ttggcacctc gatgtcggct taacttatcc tcatggatgc

16021 agtaactatg tagggtacga ctgttcgtcg attaaaaagt tacatgagct gggttaaata

16081 cgtcgtgaga cagtatggtt tctatcttct agggggaatt agaatataat aaggagtaac

16141 cttgtacgaa aggaacctgg gaaatgcctt attctcaaaa agaataacgg ttttattaac

16201 ctctggttta tctgttgttt atgtgcccag atattaattt aggtataata tttatattat

16261 acagggatgt tactttcact taagtaaata tatagcatag gcatggcaga aaagctaagt

16321 tagtcagaga taagtgctga aagcatataa gcacgaagct caccttaaga tatttcttaa

16381 atatacgtaa tataatatta cgtgataggc ttagtttgta ataatctaga gatttttagg

16441 tactaagtac taattttata aataactgaa tatttatcgt atcttatatt tagcttggtt

16501 agcataaaag taatgcaatt gttttgtaat caatagacac aagtgcgata cttgtactga

16561 gcttaaattt ataataagga ctagtagaca agtggctaag tcatagcttt ttcaatgtta

16621 ctatcgcagg ttcgaatcct gtctagtcta gttatataag aggctataac ttaattggta

16681 gagtgtactg ctcatgacag taattgtaag tgttcaactc actttagcct tatattgtgg

16741 attggtgtaa tagtaacatt tttgactcat gatcaaaaga agaaggtgcg aatcctttgt

16801 ccgcgttaat aaaataaaaa taaaaaataa tttaatatcc gaatgctgga attggtagac

16861 agtacaaact taagctttgt tggtgaataa ccgtgaacgt tcgaatcgtt tttcggataa

16921 aaaaaatatt tttattagca actgtaattt aatggtaaaa ttatcgtctt ccaaacgaag

16981 catatcggtt cgattccgat tagttgtata tcggggttat agtataattt ggtagtacca

17041 caattttgcg tattgtttgt ttaggttcaa gtcctaatat ctccataagc tcagataact

17101 caattggtag agtgaaatat tgaagctatt tttgttgtaa gttcgagtct tactttgggc

17161 ataaaaagta ttaatgggat tttaatactt ttatattata ctttatatta atatattatt

17221 ttagagattc gaatgctgaa attggtagac agggtaagct taggacttat taattaataa

17281 ttgtgaaggt tcaagtcctt cttcgaataa atttgtttta attcttccta aatagtaaaa

17341 attgtatttt ctatttcgta gagtaatggg taactcatgg atttttgatg tctaaaactg

17401 ggtgttcgaa tcacctcgaa ataataaata tatatatata gatatattta tttttaataa

17461 ataaattttt tgataaaata gtaaaagata tttaatttgc tatatattat aacaggatga

17521 tatagctcaa taggtagagc aactgtatgt ggcacagtag gttctctgtt cgacccagag

17581 tattttccct tatatagctt gagtagttta acggtaaaac cttattttca tgcgataatg

17641 ataggtgttc gattcgcctc ccaagctaaa catgtgatta tatattaaaa ataatttact

17701 attaattaat attaatgtaa aattaaaatc aagaatttat atatatatta aaaagttggg

17761 atagtttaat tggttaaaac tttaatttca tacattaaca atgagaattc gattttctct

17821 cttggcttac ttatatataa tatgtaagtt caaaattaaa aaataaaaat gcttttagtt

17881 tctatattat cattattact ttctaatgcc gttactatac gacgagatat atctatactt

17941 ttcaatagag tagctataat tgcattagct tattctatac tacacagtgc aataagttta

18001 tttattataa ataaaggtat aggtttacac ggtggtttat taaatattac aaatataaca

18061 caaatatttg atatttttat atttttcata agtatattaa tattacaatt aactagtttt

18121 ttccctagaa aagtttgggt accagaacat tcttcattac cacaattatt atttaataat

18181 tttgtttttt atagaacaaa aattattaat aaaatgggag aacatttaaa aataatagaa

18241 taccctttaa ttttattatt tataataagt ggtgcagtat tcttaatatc tacaagtgac

18301 ttaatttctg tattcctttc aatagaatta cagagttacg gattatactt attaagtact

18361 atatatagaa attcagaatt atctactaca ggtggattaa tgtatttctt attaggaggt

18421 ttaagttcat gttttattct attaggtaca gctttattat atgcaaattc aggtactaca

18481 aatctagatg gtttatatgt tataactagt ataagtgata acttggattt ctgatacaaa

18541 ccttattata ttaacttttc tttactaata tttagtatag gattcttatt caaggtaagt

18601 gcagctccat tccatttctg atctcctgat gtttacgatg ctatccctac aatagttaca

18661 acattcgtgg caattatagc taaaatatct atatttgtat tcttattaga aatagtatat

18721 tatactagaa attacttcac agattttaat tgaacatacg gattattaat tagttcttta

18781 ttctcattaa taattggtac agtagtaggt ttaactcaat ttagaataaa aagattattt

18841 gcttatagta ctatttctca tgtaggattt attttattag ctttaagtat atctagcata

18901 gaatcaactc aagcatttat attctactta atgcaatatt ctataagtaa tttaaatgcc

18961 tttataatat taattacaat aggattctct ttatattatt acacaagtga taataaagaa

19021 tacaaagaat tattagataa aaataattca cctatacaat taattagtca attaagaggt

19081 tatttctaca taaaccctac tttatcttta agtttagtta taactatatt ctcttttgca

19141 ggtatacctc ctcttgtagg gttctttgca aaacagatgg tattaagtgc tgctatagat

19201 aatggacata tattcttatc tttagttgct atacttacta gtgtaatagg tgctgtatat

19261 tatttaaata taataaaaga aatattcttc ttttctccag attataaatt aaatccttta

19321 ttaaaagatt taaaatttag tggtaatatt tataacaaac aaaatgtttt agtaaaatct

19381 attacattta aatataataa tatcgtaata tcaagtccta tagctattac aatatctgta

19441 ataacaatga taatattatt atttatattt gcaaataaag aatgattaag tatgagtacc

19501 atagtggtac aatttttatt taattattaa atgagtagta tgagtatatt ttttatattt

19561 gttatgatta tagcagcact ttttttagtt attaatttac tatttgctcc ccataatcct

19621 tatcaagaaa aatattctat ttttgaatgt ggattccata gtttcctagg tcaaaataga

19681 agtcaattta ctattaaatt ctttatatat gctttaatat tcctactttt agatctagaa

19741 atactattaa tattcccttt tggagttagt ggatacgcta atgatttata tggattaatc

19801 atagtactaa tatttacaac aatcgtaaca ataggttttg tatttgaatt aggtaaaaat

19861 gctttaaaaa ttgatagcag acaagcttta attaaaaata aaaataataa tattaaaata

19921 tttactttat aatatataat attattatta aaaaattatg tgataaaata tctaattaat

19981 aatataaatg atatataatt cattgtagat aagaaattat aacaataaca atacaaatgg

20041 ttcaagttgc aaaaatcata ggaacaggta tagctactac aggattaata ggagctggag

20101 ttggaatagg agtagttttc ggtgctttaa tattaggtgt tgctagaaat ccttcattaa

20161 gaggacaatt atttgcatac gctatattag gatttgcttt tgctgaagct acaggattat

20221 tcgctttaat gatggctttc ttattactat acgtagctta atttatttta ttttatgaaa

20281 taattattta taaaaaattt tataaataaa ccctaatagc ttaaagacaa agcataattt

20341 aatgtaaatt ataatgagtg tttgaatcac tcaaagggta ccctaaaatt ttaagaatat

20401 ttttaataaa tattaaaaaa gtatttatta aaaatttcta atgttattac ttaattctat

20461 ataattaatt taaccatata gaaaataaat aaaaataaaa agatgaattt acttttaaac

20521 aatttaattt ttaaattaga tgctccagaa gcttgaggaa tatatttcca agatagtgct

20581 acacctcaaa tggaaggatt aatagaatta catgataata ttatgtatta cttagttata

20641 atattattta gtgtaggatg agtattaata tctatcataa gaaatttcgt tagtacaaca

20701 aatcctattt cacataaata ccttaatcac ggtacattaa tagaattaat atgaacaatt

20761 actcctgctg taatattaat attaatagct ttcccgtctt ttaaattatt atatttaatg

20821 gatgaagtta gcgatccttc tatgtcaata ttagctgaag gtcaccaatg atactgaagt

20881 taccaatatc ctgatttctt agattctagt gatgaattta ttgaatttga ttcttacata

20941 attcctgaat cagatttaga agaaggagca ttaagaatgt tagaagtaga taacagagtt

21001 ataatacctg aattaacaca tatcagattt attataacat caggtgatgt tatacattct

21061 ttctcatgta actcattagg tatgaaatgt gatgcttacc ctggaagatt aaatcaatta

21121 tctgtatttg taaatagaga aggagttttc tacggacaat gttcagaaat ttgtggtata

21181 cttcatagct caatgcctat cgttatagaa tctgtttctt tagaaaaatt cttaacatga

21241 ttagaagaac aataattaaa taataataat aaaatattaa acttaatatt ttattaaagg

21301 tgtgttagct taattggtta agtatagtac tacggttact aggattagga gttcgagtct

21361 cttacacatc tttcttattt ttaagaaata aagaattttt tgtaaccaaa tgatttactt

21421 aacaatttat gttatgaaca taactttaat acttttttta ataggaattt taggatttgt

21481 tttaaataga aaaaatatta tattaatgct tatttctata gagataatgc ttctatctat

21541 aacattttta attctagtta gttcacttaa tattgatgac ataataggtc aaacttatgc

21601 tgtttatata ataatagtag ctggggctga atctgctata ggtttaggga tattagtggc

21661 cttctataga ttaagaggaa gtattgctat agaatacaaa taatgtattt aagtataata

21721 gtattacctt tattagggtc tataatttca ggattttttg gtagaaaagt aggagttact

21781 ggagcacaaa taataacatg ttcatgtata ttattaacaa ctactttagc tataataact

21841 tttttcgaag taggtttcaa caatatagta ttatctatta atttatttag atgaattgat

21901 agtgaatgat ttaatattat atgaggattt caatttgaca gtttaacagt ttctatgtta

21961 atacctgtat taataattag ttctttagtt catatttatt ctataggtta tatgagtgca

22021 gatcctcata atcaaagatt tttcagttat ttaagcttat tcacatttat gatgataata

22081 ttagtaacag ctaataatta tctattaatg tttgtaggat gagaaggggt tggagtttgt

22141 tcttaccttt tagttagttt ctgattcact agaatagctg caaatcaaag ttctatgtct

22201 gcattcttaa ctaatagagt aggtgattgt ttctttacta tagggatgtt tgcaatacta

22261 tgatctttag gtaatttaga ttacagtaca gtattttcat tagctcctta tattaatgaa

22321 aatatagtaa taataatagg agtatgttta ttaataggtg ctatggctaa aagttctcaa

22381 gtaggtcttc atgtttgatt acctatggct atggaagggc ctactcctgt ttctgcttta

22441 attcacgctg ctacaatggt tacagcagga gtatacttat taatgcgttc ttctccttta

22501 attgaaagtt ctcaagtagg tcttcacatt tgattaccta tggctatgga gggtcctact

22561 ccagtttctg ctttaattca cgccgctaca atggttactg caggtgtata tttattaatg

22621 cgttcttctc cattaattga atatagttct actgtattat tattatgttt atgattaggt

22681 gctataacaa ctgttttcag ttctcttgta ggatttttcc aacaagatat aaaaaaagtt

22741 attgcctatt caactatgtc tcaattaggg atgatggtta ttgctgtagg tttatcatca

22801 tataatgttg ctttattcca tttagttaat catgctttct ataaaggatt attattctta

22861 ggagcaggtg ctgttataca ttcaatggca gataatcagg attttagaaa atatggagga

22921 ttaatatctt tcttaccttt aagttattct gttatattaa ttgcaagttt aagtttagta

22981 gccttccctt ttatgacagg attctatagt aaagatttta tattagaatc tgcttatgga

23041 caatattctt tcagtagtat atctgtttat gttatagctg tgataggtgc aatatttact

23101 actttatatt ctgtaaaagt tctttactta acattcttag ctaaccctaa tggtcctcta

23161 ataaattata aacacgcaca tgaaagtgat atatttatga gtcttccttt agttgtatta

23221 gctatatttt ctatattctt tggatatatt actaaagata tatttatagg tttaggttca

23281 ggattcttta ttgataatag tatatttatt caccctaatt ctgaaatcat gatagatact

23341 gaatttggtg tttcaactta ttgaaaacta ttaccatttg ttttcactgt ttcatttagt

23401 ataatagcta ttatattatc tgaattctta tctgaacttg ttattaattt caaattatct

23461 acattaggta gaactatctt tggattcttc aatcaaagat tcttagtaga atatttctat

23521 aacaaatata ttactaattt aatattaaat attggaggac aaactgttaa agttttagat

23581 aaaggtagta tcgaactatt aggaccatat ggtttagaaa aaaaattaat tagttcaagt

23641 aaaaatatta gcagtttaaa taaaggtatt gttacaaact atgccttatt tatactagta

23701 gggtttatag tatacatgtt ttctatatct ttaggtttta ctaataattt aagtatatta

23761 atattaatat taatgtttat tattagttca ttatattaat aaaatatcaa aaaaaaaatt

23821 ttatatttag tacacgatac ataataacac cccttacatt aaacaacggt ccttataatt

23881 aataataatt tatatgaaaa ttttcatata tgtatatata aaaaaaaata aaaataaaaa

23941 aaaatgagaa tattaaaaaa caatggttta ttaaaattag ttaattcgta tcttatagat

24001 gcttcacaac caagtaatat aagttattta tgaaactttg gttcattatt agctttatgt

24061 ttaattatac aaattataac aggtgtaaca ttagctatgc attacagtcc aagtgtttta

24121 gaggctttca attctgtaga acatattatg agagatgtaa ataatggttg attaattcgt

24181 tacttacata gtaatacagc ttcagccttc ttctttttag tttacttaca tatagctaga

24241 ggtatgtact acggatcata caaatctcct agaacagtag cctgatcaat aggtgtagta

24301 atacttatac ttatgatagg aacagggttc ttaggttacg ttttaccata cggtcaaatg

24361 tcattatgag gtgcaacagt tattacaaat cttataagtg ctattccttg aataggacaa

24421 gacgtagttg aatttatatg aggaggattc tcagttaata atgctacttt aaatagattc

24481 tttgctttac actttgtgtt accattcgta ttagctgctt tagtgttaat gcatttaata

24541 gctcttcacg ataacgcagg atcaggtaat cctacaggat tagctggtac ttacgataga

24601 gttccttttg ctccttattt cttattcaaa gatttaatta ctatatttat tttcttcttt

24661 gtattaagtg tatttgtttt ctttatgcct aatttcttag gagatagtga taattacata

24721 atggcaaatc caatgcaaac acctgctgct attgttcctg aatgatactt acttcctttc

24781 tatgctattt taagatctat acctaataaa ttattaggtg ttattacaat gtttggtgcc

24841 ttagctattt tattagtatt accaaaaaca gatttaggaa gatcaagagg tttcactttc

24901 agacctttaa gtaaagtagt tatattcatg tttggagctg tattcttaat attattaaac

24961 ttaggacaag ctcacgtaga agatcctttc atagtattag gacaagtatt tgctgtatta

25021 tatttctcat acttcttagt aatactacca gtagtaagtg tatgtgaaaa tagtcttatg

25081 gatatatctc ataatgatta ataaatgttc aaaaaataaa tataaaatat gtatatatat

25141 atattttata aagattatgt tgtaagtggt ttacactttg attgcagatc ttaagtctga

25201 ggttcgattc ctccataatc tttatttatt ttaattaata tactattcta atatattaaa

25261 ttcttattag cttaatggta gagcaagata cttctaatat caagattcta gttcgaatct

25321 agaataagaa ttttctttcc agtcaaattt ccaatttcaa aatttaatac tatttagtca

25381 cttattttta ttagctatta attattttag taatgaacaa aaaggattat ttacattaaa

25441 atcatcaata tctgtgtcaa tagaaagatg attcttatca actaatgcaa aagatatagg

25501 tacactttat ctaatatttg cattattttc aggattatta ggtacagctt tctctgtatt

25561 aataaga

//

LOCUS Grosmannia penicillata 150891 bp DNA linear

DEFINITION .

ACCESSION

VERSION

KEYWORDS .

SOURCE mitochondrion Grosmannia penicillata

ORGANISM Grosmannia penicillata

Eukaryota; Fungi; Dikarya; Ascomycota; Pezizomycotina;

Sordariomycetes; Sordariomycetidae; Ophiostomatales;

Ophiostomataceae; Grosmannia.

REFERENCE 1 (bases 1 to 150891)

AUTHORS Wai A. and Hausner G.

TITLE

JOURNAL

COMMENT

FEATURES Location/Qualifiers

source 1..150891

/organism="Grosmannia penicillata"

/organelle="mitochondrion"

/mol_type="genomic DNA"

/strain="CBS 134055"

/isolation_source="wood"

/host="Picea abies"

/culture_collection="CBS:134055"

/db_xref="taxon:360150"

/country="Slovenia: Dravograd"

gene 1..26757

/gene="cox1"

CDS join(1..108,2565..2668,5539..5607,6854..6958,8247..8353,

9960..10081,11213..11306,12739..12836,16185..16244,

17379..17411,18409..18565,19868..19935,21014..21150,

22164..22182,25018..25032,26449..26757)

/gene="cox1"

/codon_start=1

/transl_table=4

/product="cytochrome c oxidase subunit 1"

/translation="MSIERWFLSTNAKDIGVLYLIFALFSGLLGTAFSVLIRLELSGP

GVQYIADNQLYNSIITAHAVLMIFFMVMPALIGGFGNFLLPLLVGGPDMAFPRLNNIS

FWLLPPSLLLLVFSACIEGGAGTGWTLYPPLSGIQSHSGPSVDLAIFALHLSGVSSLL

GAINFITTIANMRTPGIKLHKLALFGWAVVITAVLLLLSLPVLAGGITMVLTDRNFNT

SFFETAGGGDPILYQHLFWFFGHPEVYILIIPGFGIISTTISANSSKSVFGYIGMVYA

MMSIGILGFIVWSHHMYTVGLDVDTRAYFTAATLIIAVPTGIKIFSWLATCYGGSIKL

TPSMLFALGFVFMFTIGGLSGVLLANAALDTAFHDTYYVVAHFHYVLSMGAVFAVFSG

WYFWIPKILGLNYNMLLAKVHFWLLFIGVNLTFMPQHFLGLQGMPRRISDYPDAFAGW

NLVSSFGSLVSVVASVVFLYVVYKQLLDGSPATRFPWSTPQYFTDVLQALLNRNYPSL

EWAISSPPKPHAFVSLPLQSNVVNLH"

intron 109..2564

/gene="cox1"

/note="Group II"

/number=1

gene 109..2421

/gene="orf770"

CDS 109..2421

/gene="orf770"

/codon_start=1

/transl_table=4

/product="hypothetical protein"

/translation="MRRCVILYLVESFNRQSTNLSIASGLARIERYLVNLACLLKALE

NRIQVDPTSQAIVLTVKIILFELYLKVISRKGWTLACHRCRMLIILLLVRGIIGSNIN

QITRSNKREEGGPKRATGYKMELRDSLRGMQISYGYGGSVIGKNLPKGTRAFSSDSSQ

EGSGVRPTKLPTRFVKLMSICEKRTSNFKVSDIYELMFNINMYEIAYNKLKSNLGNMT

PGIDKITLDGISTEVFTNIIEAMKDESFKFKPGIRVHIPKSNGKMRPITVAPPRDKIV

QEVMRMILEAIFEPTFSINSHGFRPNRSCHTALRQVKTQFGAASFFIEGDISNCFDSF

NHELLIQTLEKRISDVRFIRLIHKSIKAGYVEFNRTKLSIIGTPQGSIVSPILANIFL

NPLDKYIDELKSEFDRGKAAKINPEYKRLDYYRNKAAKTSNPTLATQLRKKMQSISAR

NPKDPDFRRLYYVRYADDWLIAIRGSRTEVQKILDQIRLFLKNSLALDLSTEKTLVTN

PRERSALFLGTQIKISNHTYFYKHDNGIKRRAASQLVFTAPLDRIYKKLEEAGFYDKN

SKRSTARMLWYHESKDTIIILYNSVLRGYLNYYSFTRNIGRVAASIEWILKNSCGQLL

AAKFKLNSTQGVIDKFGPDFKGSDNHAFFKPSYKMNQWDFKMTGVKTNIKALYSSGLS

RASLDGLACSKCGSDVRVEMHHVRMLADLNPKLLEIDKIMVKKRRKQVPLCRPCHMEQ

HKQMTLNKHNTKNSQNSKKTRFSDRKSNRK"

intron 2669..5538

/gene="cox1"

/note="Group IB"

/number=2

gene 4354..4653

/gene="orf99"

/note="copy 1"

CDS 4354..4653

/gene="orf99"

/note="GIY"

/codon_start=1

/transl_table=4

/product="hypothetical protein"

/translation="MPSILKTKSRRVLRYFNKYGFDNANLTIYIMNENSSLDEVVRLE

QYFINTLKPSLNVDLVASSSGYHEPMSQEIRERLHTQPSVSSETYKEVLLFICII"

intron 5608..6853

/gene="cox1"

/note="Group IB"

/number=3

gene 5609..6496

/gene="orf295"

CDS 5609..6496

/gene="orf295"

/note="LAGLIDADG"

/codon_start=1

/transl_table=4

/product="hypothetical protein"

/translation="-

KKGHLGINIRNYSSNFKNEKFKGYLAGLFEGDGHIWIQKPSEKKKQNPRFCITFGMKN

EPLAKKLLELVGSGFIRYKLQDNACVLVVSPVIGLKKLVNLINGELRTPKIHQLHSLI

DWLNKNHSTNIAKLPLKTSSLSEDGWLSGFIDSDGSFSVLHTKLENGATKRKIACRLR

IEQRMFEPITNESYEQVLSSITNFLNCSLLTKTQKSTGNTYYTLAASSKISLNIIIDY

LDKYPLFSSKYLDYKDWKKVALLILESKHLTEEGLVITDTVRNNMNRQRTFFNWDHLN

ELSI"

intron 6959..8246

/gene="cox1"

/note="Group IB"

/number=4

gene 6960..8093

/gene="orf377"

CDS 6960..8093

/gene="orf377"

/note="LAGLIDADG"

/codon_start=1

/transl_table=4

/product="hypothetical protein"

/translation="-

CKELPWGNSGAIKLYSMHKTLDVIFDRAVYYSLSISLAVRMYIVYLILFLKFSSRQYA

WDQTITLFSSHQRLNVEHPNIHNHSNEINKSKYSENKDNFHQWLVGFTDGDGTFNIYR

SKEGKWSLYFKLTQSTYNLRILYFIKTQLGVGSVYVNFDNLNGDFRIRDRKTIGSVIL

PVFDKYPLLTSKYFSYLKFKEAYNILENNTLTTQEKDKLLLDLTSKEMGENYVSPAWE

ILNYEVNNTNDAKLVMSKYWLVGFTEAEGSFYLVSKESTRIVHAFEITQKLDEIVLKA

ISLILGINFSKKKTHYSVVTTNSRAIENIIAYFKSNIKGMKSVEYRIWARSYTKHKGN

YENLKKIREQIRNLRTIRLNKDLKIRRY"

intron 8354..9959

/gene="cox1"

/note="Group IB"

/number=5

gene 8356..9681

/gene="orf441"

CDS 8356..9681

/gene="orf441"

/note="GIY"

/codon_start=1

/transl_table=4

/product="hypothetical protein"

/translation="-

FIFLVNLFFFMFTGGANKLCSLPPAAFYSICRNYIYSVILIILVAGSQFKDLGSYLIS

AQNIQDRGDELDDINSDLDDEVDLPQNFNDDDPEEPNENDPEEPNDNKNKEKDWGLIL

GRKGKNVYVHQLAMAQLNSGKPVTLKVLNEILAYSNILVSEETLNSLLNMPRLVYYNL

HKQETINLIYEELGSPYSKTQVRGIYIFTCLSTNQKYVGSSNQLFFRLKGYLNFTHKS

IGKLIPLMKELGLSEFKLEVICLPYYPEFRPEIVLEQYHLLDPSFNLNTIRVSNNPSG

SASKALYMYNRDKSILYYSTDQQKDFISKLSISHFTFTKHLTNGTYYLGKYLFLRERI

DTAKFTEMSLPEIALMLTKDRIKFNVEKPVNSLSKTVVLINVLSEEELEFESLGQCVK

FLRSKGFSATQTTLAKYLNTDKSYKGYICITKKK"

intron 10082..11212

/gene="cox1"

/note="Group IB"

/number=6

gene 10082..11092

/gene="orf336"

CDS 10082..11092

/gene="orf336"

/note="LAGLIDADG"

/codon_start=1

/transl_table=4

/product="hypothetical protein"

/translation="-

ILPALYLANCWKEIYIISLSAGCFIILFLWSIFRDYMPKFICYNKLYSTSSTFNSNFD

SKFASYLAGLIEGDGTIVTPKVERSPKGKLYYPTIQIVFDLRDFPLAQVIQSKLKHGS

LARKKGTNAYILTINSFEGMILIVNIINGYMRTPKIISLYKLIDFLNHKFDLKIEKKD

KDNSSINSNSWLAGFIDADGHFSVRTTLEGKYPKVECKFELSQRQNDHNNENNYEFLN

LIADFLGANVKEVRMYKPKPEYRVRTTNLKSNLVLVDYLEKFPLYSSKYLNYKDWKII

LNYFEEKLHTNPDIILKIVAIKAEMNNNRTNFNWDHLEYFYNLYK"

intron 11307..12738

/gene="cox1"

/note="Group ID"

/number=7

gene 11309..12235

/gene="orf308"

/note="copy 1"

CDS 11309..12235

/gene="orf308"

/note="LAGLIDADG"

/codon_start=1

/transl_table=4

/product="hypothetical protein"

/translation="-

KKIKYIYILSILTLSSLIFNKLHNSILKSNYSTVAENFHFPSFYDKFSLHLPHITPPS

SKFLTWFIGFTEGEGSFRVNNRGDLAFVITQGNADIQVLEFIKETLGFGKVIAQSANT

SRYVTQNKKEIELIIHLFNGNIILPSRKTKFENFVKGFNIWVSKGRIRLNTVELKHTN

ILPSLNDSWLAGFTDAEGCFTCSIGKDKGFSFNFNISQKWEENIGILQHLCVLFNAGI

VSKHSADNVNEYRIGGVQNCKNIFPYFDSHLLYSKKAISYYLWKEIHTDLLNKHHLDP

VKRIEMIERSRLINKFD"

intron 12837..16184

/gene="cox1"

/note="Group IB"

/number=8

gene 12846..14924

/gene="orf692"

CDS 12846..14924

/gene="orf692"

/note="LAGLIDADG"

/codon_start=1

/transl_table=4

/product="hypothetical protein"

/translation="-

SLIKFYLLTQQTICRKVSPFLGILLGTPFNWKFIISIVKILVIYDNPQITKARSENFK

PDIKEVIGLSMLVGISEAILTQVFYYLRINNNFFNSFVKILRLKNINKQFKGLHNKAF

KLNPWYVTGFVDGDGSFLINIRPKSGRNLNYSVELGFRINLHSIDRSLLENIKEFFGV

GRLTAESENYAQYFVGSLKDLSVIINHFDNYPLVSQKWSDYQLFKLVFEMMQNKEHLT

TEGLNKIVSIKSVMNQGLSENLNSVFSNNIPWPKPELKKKRISIPDPNWITGFTDAEG

CFMVRLIKTSSSYSVNFRFTLTQHIRDAELLKILVDYFKCGRYNVRSSLLHGDFTVTK

FDDIKEKIIPFFEKYPLQSNKLLDFSDFKKVVTMKGKTYSQFSDKSLAEVNLIKSRMN

KGRKFKSDDPQAPFNPSGNRRHYSTKRTIPSNAFLLQQALRPLTCKANYTTNRDDKFK

EWLAGLIDGEGQFFKFKKGLYGLKVITNKKNKSLLYEIQHKYGGAVKEISGSKAFKYK

LVNPLGLDILVKDINGLIRNPLRMIKLNKLLLEKHLVLKEPQPLSYSNGWFSGILDTD

GSIYIDEESWQVIINLTQKNRFLLEPLQILYGGKIVTTPDAFKLSIFKKDEILKLINN

YFKVNPLRSHNALKIELIKNFYRLGHHSNLWNNRTDKFQEWILFKEKWDRIVY"

gene 14979..15914

/gene="orf311"

CDS 14979..15914

/gene="orf311"

/note="LAGLIDADG"

/codon_start=1

/transl_table=4

/product="hypothetical protein"

/translation="MFVSTLLFTNYLSILILKLNYVLFGILPDTVLSEKLVLIIKESD

SPEIQEEDSINDEVYPDENSEEPSNNFNEKENERFFEWLAGIIDGDGCFLVSKKGYCS

LEIVTQLRDKKTLYLIKQKFGGSVKLISGNNHLRYRLHHKEGLLKLINKINGLIRNPI

RILQLGRICEIYGIVLKDPKPLTYNSGWLAGFIDTDGSVYFSEASGQLFITAVQKNRF

ILEALVELYGGTIYPMIKQEAFKWNCYKKKEVLALANDYFKINPCRSEKMMRISMIND

FYKLRNLHAHTAPVNSDLGKAWKYYMIKWNSFMDK"

intron 16245..17378

/gene="cox1"

/note="Group IB"

/number=9

gene 16245..17294

/gene="orf349"

CDS 16245..17294

/gene="orf349"

/note="LAGLIDADG"

/codon_start=1

/transl_table=4

/product="hypothetical protein"

/translation="-

VKMASPYSDIGILINFAICWNSLVLISTLYGKNRISYTQSADNLSLYSFNDNKQSVSE

TTRETSFNFSAFHAYYNTLFSNKDPLSDEWLTWFIGFAEGDGAIQTYDEGKRVRFVLT

QKESDILYKLQFKFNIGVVKHFPQGKSGKDNDFYRWMVDSPSHILLLAFLFNGNLAQN

HRIEQLAKWVNALNNRFGNETIKLNNTPAKVTLQDAWLSGFTDAEGCFNVSITANSRY

TLGHVIKMRYILDQKNSVILNKVSELFGLGKVTLRSGTDNVYRYTVTGFKPINNIIAY

FKLFPLQTKKAFSFEKWLTIHNLVSNKLHLTEEGLSQIRTLQKQINLSNSMTNKTGKA

"

intron 17412..18408

/gene="cox1"

/note="Group IB"

/number=10

gene 17412..18368

/gene="orf318"

CDS 17412..18368

/gene="orf318"

/note="LAGLIDADG"

/codon_start=1

/transl_table=4

/product="hypothetical protein"

/translation="-

LVSTVKILLYAGNSWLSSPLVLIALGKIYLYFTRQSAGNFSFSTNAKAVTKNTYVKFS

ELPKISVHVPKHNDFNDEEFGYFLAGLIEGDGWFGKKELHIIFSETDTPLAYFIKKRI

GYGNVYNIKDKKAVRYICKNKEGLYIILSLINGKFVSNYKYDQLIKHNYSEDFNINIL

PPLNSLSLDNPWLAGFTQADGCFFISVVKSKTHKSGYSVRLEFSLKQNDELPLRLLYN

NLGMGNISQYHTGVWCYKSSGYKTAAALINYFDKYNVFAGKYVDYLKFRKVYIMITEG

KHLDIGGILKIKSITTKGSSETSTQEI"

intron 18566..19867

/gene="cox1"

/note="Group IB"

/number=11

gene 18568..19854

/gene="orf428"

CDS 18568..19854

/gene="orf428"

/note="GIY"

/codon_start=1

/transl_table=4

/product="hypothetical protein"

/translation="MIHLVLPLKITICWELLTIILLVIYLITVTMYNFEQSAGNQQIS

KNILVGTSETTRDPCNVVAWRYSPWNKKPLLPLYNYNKIIIRYYSHSQLTGRRSLILR

ISDEDNTNNLKPLVIYENFKEDRSKLLMEQKDKSGVYCLINKINNHSYVGSSINLSSR

MKNYLNNTFLKNKKNINMPVVKALLKYGKDNFTLYILEYVDVKSLNIRETYFITSVVP

YYNVLKQGYSSLGYKHTEETKKLLSELAKNRVHSDTTKGLISKALTGENNPFYNQNHS

MESKVRMMEANSAYPVYVYNSFKKLLVIYPSVTTLAKLIKSNHPTIVSNIKEQTLFRG

EWYFTNLPFSIKDIPLIANWNSGEAEKLISDMNKNSHIKKGIFVYDENKNFICKYEGV

TDAQKALNINHSTIKKYAKINGNYNGYIFSYERLNY"

intron 19936..21013

/gene="cox1"

/note="Group IB"

/number=12

gene 19936..20967

/gene="orf343"

CDS 19936..20967

/gene="orf343"

/note="LAGLIDADG"

/codon_start=1

/transl_table=4

/product="hypothetical protein"

/translation="-

VGLNNCYDYFAFGYMLGTMCLGIYLLYIAYCYLLKKIDVSASSRKLNFLTMYSENDKL

PDTLKSTDIQLAENCKGFSETTRQISDFDDKDFFKWLAGIIDGDGNFDVRNINSNLVL

KAIRIKLHNRDVRILSRIQNTLHLGRIRSDANKPHSMWIISKKEEMFFLINNINGLIR

LKVPGLKKSCDYLGIDFIEADYNIQANDPYLSGLVDTDGTIVFNYTGNRIECNLEFEH

NEYTSKLNLDNVIPNYKPSKIFRENRNTLAFRFQNVKEMVFLYDYFMKNRLYSDFKFY

RVSKIKEFIPIRSYKNNPKDSLEFKKYSEFVLNWIQYKNPTWHKVTFIDKIR"

intron 21151..22163

/gene="cox1"

/note="Group IB"

/number=13

gene 21152..21958

/gene="orf268"

/note="copy 1"

CDS 21152..21958

/gene="orf268"

/note="GIY"

/codon_start=1

/transl_table=4

/product="hypothetical protein"

/translation="-

LKGSTFVAKGQRGFSATPKNSPGPNEFVLFFENVNKEKINIYKELRKKAGVYLFQNKI

TNDFYIGSSTNLTSRMVSYYYYTNSRRSHPSTMVIIRAIKKYGLDNFSLGILEFCEKD

VCLMLEQKWLDHYKPKYNVLSTAGNSLGYKHSIETINKLKEKLSKELHPKFGTVTSSE

TREAISESIKEFYTKNSHPSKGLKGKLSPQYGIGGQFVFCYNKNGEELIFPSINGARQ

HFKVRWTLIKSNIDKNKWITLNGEDWLIQSSPRQI"

intron 22183..25017

/gene="cox1"

/note="Group IB"

/number=14

gene 22549..23184

/gene="orf211"

CDS 22549..23184

/gene="orf211"

/note="GIY"

/codon_start=1

/transl_table=4

/product="hypothetical protein"

/translation="-

RIAAHSVYTDLHLSETLVKIRKDLKSVSGVYALGIPGFEMVYVGSSVNLASRALDHIK

NHQSNIYLQNAIVKYGLNKFYFYVLELLPEDLSSYDDLLKLEQKYLDLFKSKYNFENF

ARKSRAGTFSSNKARQLMSDQKKASYTEERRTSISEQFRKELFIYDATTLNLIKKYDK

QGEFIQEFKVSFKTVIKYRDSGKVFRDKYLLSSKLM"

gene 23363..23433

/gene="trnR(cct)"

tRNA 23363..23433

/gene="trnR(cct)"

/product="tRNA-Arg"

gene 23754..24989

/gene="orf411"

CDS 23754..24989

/gene="orf411"

/note="GIY"

/codon_start=1

/transl_table=4

/product="hypothetical protein"

/translation="-

ISTYLLSKLDSPKSSSSSKYSLSPKIHKLGLSSRFYSTNTVNKSLPEIDIINSKSIEF

DSLEQACEEIKFKYLGISGVYKLTNVKDPIRFYIGSSNNLARRMDEYLKLTKGLRKPR

SSSELEISKCSALDWSLEFIYLTAPQTSLAYEQYAIIKFKPTINNYLNVIPRVNPQWG

ENLDNAILEIENLLSLFNKGSEGYIRLNVFLQTFKIANNLDYSLEDLDGKYYCFLIFV

YDINYPNNRPLVYSSINRALKGLQISPSTLLDYVNNKYIYNSSFILSFEPLESFNGYQ

EKLSGDNQLRKHITVYNQDNEIVTEFKSGRELARYFQIDGKVARAAITKGEYQDFLLV

SKEVSNRKLIYVFDSNTKELLERINGLSKALKYAKVNYYTLKSLIENGNSLGGKIYSY

KDKL"

intron 25033..26448

/gene="cox1"

/note="Group IB"

/number=15

gene 25455..26093

/gene="orf212"

CDS 25455..26093

/gene="orf212"

/note="GIY"

/codon_start=1

/transl_table=4

/product="hypothetical protein"

/translation="-

CIQELEQNEVNSNNFITIEPEYQFHKNYKLDLVNLVASLYTASSSIFVSPIYNFLDDK

ETILNESLRLKNKSGVYLIHNGVNGKEYIGSGIDLKRRLATYYFPSRLIDNRYISNSI

LKYGHGNFSLVILYIYLMKLGGAENKETSLIEKEQEYIDLYKPVLNLNPKAGSSLGFK

HSEESKKLISKIRKGKSLSEETKKKLSLLFSKELNLI"

gene 26179..26433

/gene="orf84"

CDS 26179..26433

/gene="orf84"

/note="GIY"

/codon_start=1

/transl_table=4

/product="hypothetical protein"

/translation="-

FIAQMYKDKRGANNPMFGKTKSEETLIKLRKKVYIYDSNKQFIKCYDSVGFAVKDLHI

AAETIKKYLNTNRAYKDKYFYSNLQ"

gene 26960..33971

/gene="nad1"

CDS join(26960..27103,28791..28937,30232..30328,32506..32753,

33534..33971)

/gene="nad1"

/codon_start=1

/transl_table=4

/product="NADH dehydrogenase subunit 1"

/translation="MLYFSTLISILEVVIILVPALLAVAYVTVAERKTMASMQRRLGP

NAVGYYGLLQAFADALKLLLKEFVAPTQANMILFFLGPVITLIFALLGYAVIPYGPGL

AISDFNLGIFYLLAVSSLSTYGILLAGWSANSKYAFLGSLRSTAQLISYELVLSSAIL

LVIFLTGSLNLTVNIESQRAVWFILPLLPIFLIFFIGSVAETNRAPFDLAEAESELVS

GFMTEHAAVVFVFFFLAEYGSIVLMCILTSSLFLGGYLTLGNSILDSIFNFYGTENFI

NTIISNSSLLEGLIYGLTLGIKSSMMIFVFIWTRASFPRIRFDQLMSFCWTVLLPIIF

AVIILIPAIMYNFTLLPVNISLL"

intron 27104..28790

/gene="nad1"

/note="Group IC1"

/number=1

gene 27104..27910

/gene="orf268"

/note="copy 2"

CDS 27104..27910

/gene="orf268"

/note="GIY"

/codon_start=1

/transl_table=4

/product="hypothetical protein"

/translation="-

LPNIPLKKFYHTSTIDKLTNDLYLNRKAPIKPFKDNVLKTCKDLLSPSDLNIFFRSIR

SKGGIYIFTYKENSDIFYIGRTKNFRNRFKAHLNVNLKDKFHVFANAVGWDKFQFSII

QICSLVTQKEREDYYLQKYLPLLNTIYKGNMNTTPNYDSLYEILKLKQLELNKDNKYL

GIPIYLYTYTEGSLEANYIKFDSINQLSQYLNISRETINIYLNTYVPYKSNIFFTDLI

EDNELIDKLISDTCIHQGLDLDRNISKRVLVYSRY"

intron 28938..30231

/gene="nad1"

/note="Group IC2"

/number=2

gene 29338..30204

/gene="orf288"

CDS 29338..30204

/gene="orf288"

/note="LAGLIDADG"

/codon_start=1

/transl_table=4

/product="hypothetical protein"

/translation="MDPWFITGFTDAEGCFTCSILKSSGYKLGWEIQPAFQIKLHVKD

YPLILGIQHSLGNIGTVISSQSTCTFKVRKLKELLELIKFFDNYPLISNKKGDYLMFK

QIVSIIQLKEHLTTEGIQKIVNLKATLNFGLSKELQLMFPETIPVARPLREPCVIPHS

QWLAGFTAGEGNFSVSLDNGIFKSLLFKITQHEKDEVLLIAIKDYLNCGNCYLRKKEN

TMDFKVTKFSDVTEIIIPFFINNSILGVKSLDFKDWCLVSEIVKKREHKLEEGIKKIK

EIQKGMNRGRSF"

intron 30329..32505

/gene="nad1"

/note="Group IA"

/number=3

gene 30331..31668

/gene="orf445"

CDS 30331..31668

/gene="orf445"

/note="GIY"

/codon_start=1

/transl_table=4

/product="hypothetical protein"

/translation="-

LSPSFIKIKGYNSKEFQEKLSLSQLEAKYIFFLRTFVENKLCMRSTTSRLILPRVFEI

LHYIFIIFWILFLYTIIFSYNMAWLNLNIFYIIFIISLTITSFFYNATSRIFVVESHN

SDEAYYSKNSGFSNYNFSGSIYAGTKTPNNVNFNKILLNRSYSTKAKPEISDTDNYNL

PQELKALHTLYINDLTKDRLAPVTSFENDQVFASFNLSNEEEKSEFLREWGSKGGIYI

IQYLYNPCIYYIGRTTLFKRRINNHLRAETNSKFHVFLSLVGKEYFSFGIIEICTKDE

QGKRENFYLQKYLPLLNTTFSSSYTETSIYKSLTEKLKSLKSPVNSEKSNKPMPIYVY

SVSENNNIINQDYIKYNSLAETCKMEDISYNTLLLFRDTKIPFRGKLYLTNPILEFDS

ILEEMNNNLKDITLRSSTAKKVWVYDAKTLNQVEGSPF"

intron 32754..33533

/gene="nad1"

/note="Group IB"

/number=4

gene 34205..34963

/gene="orf252"

CDS 34205..34963

/gene="orf252"

/note="GIY"

/codon_start=1

/transl_table=4

/product="hypothetical protein"

/translation="-

NINLEFEQVFTNLQDELSILKEVKECLSSLSGIYAFLHNDTQKAYIGSSGNLAVRISD

HIKGKHSNVYLQRAFVKHGIENFSLYILEILPEDTDLSDVENLAELIQLEQKYIDLFE

DKYNINPVAGKSRLGAKHSEATRELMSKWRKENPAFLNKTHSNEYIETLRERMSGSSN

PMYGKPVTEENKKLISELFRKYVYLYDANSLELIGEFSKHADLVKELSISSKTLIKYK

DSGEVFRGKYIISSLKINK"

gene 35263..38068

/gene="nad4"

CDS join(35263..35773,37116..38068)

/gene="nad4"

/codon_start=1

/transl_table=4

/product="NADH dehydrogenase subunit 4"

/translation="MLLSFLLLVPLIGIFFIAGTISYENNVVSATYYKNIALITSIVN

LVISLIVYLLYDSNNNQYQFVQEYYNLSFFDIYLGVDGISIYFVLLTTIIMPIAILAN

WNSITENLKSYLIIMLLLETLLLAIFLVLDILLFYIFFESTLPPLFLLIGLFGSNNKV

RASFYIFLYTLWGSLFLLICILAMSSIMGTTDFDALFKTNFDYTTQLFLFGGVFLSFA

VKTPTIFLNSWLLKAHVESPLGGSIVLAAIVLKTSLYGICRLILPILPKATMNFTYIV

YVIGVITIIYASFSTLRTTDIKELIAYSSVSHAAVYLIGIFSNTIQGIEGSILLGLGH

GFVSSGLFICAGGILYDRSGTRAIYFYRGIAQVMPLFSILFFILSLGNCGVPLTLNFA

GEFMSLYGAFERLPLLGVFASSSIVFSAAYTIYMFNRIAYGGTFSRYFKENISDVTKR

EFFLLFILVVFTVIFGIYPSFILDGLHYSVTSLIYYS"

intron 35774..37115

/gene="nad4"

/note="Group IC2"

/number=1

gene 35776..37038

/gene="orf420"

CDS 35776..37038

/gene="orf420"

/note="LAGLIDADG"

/codon_start=1

/transl_table=4

/product="hypothetical protein"

/translation="MLKCKRAKHRGSPKALVTKALKEIFKLAWLMTQGMVKSLVFYLE

KFFFITDMWVIAVLSHLFFKDVKEQRVDGSSIPRNLDIVRCTLVAGKPVLRRKIYTDS

NKSIIINKFKRFSSSKSNLNPWFVTGFTEAEGCFMLGIFKSNNYKTGYQIQGIFKITS

SSEASLHKKDYDLLSQIKDYFGVGTITNHGDTTLQYTVKSIKDLEKIIYHFDKFTLLS

QKSVDYLLFKNAIMLIKNKEHLSNEGFRKILSIRAAMNLGLPEELKLNFPDIIPFSRP

SPLKLNSIDYNYISGLTSGDGCFYVSIRNAPTTKTGKSVTFKFHIVQHSRDIELMKKL

ISTLNCGRIELNLKQSAVYFVVTNFQDIVDQIIPLFDKYPIKGIKSLDYEDFKLIVNL

MQTKEHLTEEGLSKIQSLKLNMNLFRKL"

gene 38128..38283

/gene="atp8"

CDS 38128..38283

/gene="atp8"

/codon_start=1

/transl_table=4

/product="ATP synthase F0 subunit 8"

/translation="MPQLVPFYFLNEVIFAFAVITIVLYISSKYVLPRFVRLFLSRTF

ITKLFDK"

gene 38379..46260

/gene="atp6"

CDS join(38379..38471,40045..40307,41681..41857,43571..43621,

46053..46260)

/gene="atp6"

/codon_start=1

/transl_table=4

/product="ATP synthase F0 subunit a"

/translation="MNTLSINTVNFEILSPLSQFEIRDLLSIDAPILGNLHISLTNIG

FYLTISLVLILALNLLSTNYNKLVSNNWSIGQESLYATIHSIVTNQINPRNGQIYFPF

MYTLFIFILINNLIGMVPYSFASTSHFIMTFSLSFTIVLGATILGFQKHGLEFFSLLV

PAGCPLPLLPLLVLIEFISYLARNISLGLRLGANIMSGHMLLHILAGFTYNIMSSGVI

FFVLGLLPLSFIIAFSGLEIGIAFIQAQVFVVLTSSYIKDGLDLH"

intron 38472..40044

/gene="atp6"

/number=1

gene 38472..38810

/gene="orf112"

/note="copy 1"

CDS 38472..38810

/gene="orf112"

/codon_start=1

/transl_table=4

/product="hypothetical protein"

/translation="-

VKSGKLNTMWDKLPNSGDTLKLLVPSYNLKIVNGWTNHSCTVTSQKIFERLIGYRGSK

LVTFILLALCFNVMFNFNSLFDSLLISLLFFIMWNNKYCYYKRATSRRQLTKR"

gene 39079..39921

/gene="orf280"

CDS 39079..39921

/gene="orf280"

/note="LAGLIDADG"

/codon_start=1

/transl_table=4

/product="hypothetical protein"

/translation="MVSVRKATAYRQGWKVQAAFVITQHKRDIELIKNIRAYLGGIGS

IDPRDEDVIQLRVFSIEQITNIVIPFFDKYPLVTQKKADFELFKLAVNLINSKRHLTE

EGLLEIVNIKASMNKGLSQSLKEAFPDYIPVTRPLIEGETQKIWNPYWLAGFVTGEGC

FYIKQRESAAHNKKIVELIFTITQHSKDKALLQSLVSYLGVGRFSLSKNVAYYTCSKL

SDNFSKILPFFQKYPILGIKSNDFNDWKKACEIVKAKDHLKEEGYHQIKLIKEGMNKG

RSTR"

intron 40308..41680

/gene="atp6"

/note="Group IB"

/number=2

gene 40309..41355

/gene="orf348"

CDS 40309..41355

/gene="orf348"

/note="LAGLIDADG"

/codon_start=1

/transl_table=4

/product="hypothetical protein"

/translation="-

RCLRIFLLFISNNLLKNKIRLYSSYSRSNIHNVTSLDQTRYSFNNKNTFYLNPDYITG

FVDGEGCFSISIFKDSRRLSGLQVKPIFSISLHNRDIDLLNAIQRTLNVGKIYKHGSD

SMQYRVSSLKDLQTVIEHFDKYPLISQKRADYLLFKEAMSVIKNKEHLSTVTGGCGLL

KIVGIKAALNWGLSEKFKESFPNVEPVNRPKVEPTSLINLNWIRGFTEAEGSFQVVVY

QETKNRTTVSLRLSITQHSRDEALLSNIATCLNCGRYYKSPTRNEGQYLVTVFADIHT

KIIPFFNEYPLIGSKKEDYLDFVQIADLIKSKDHLTVEGLDKIKFISNNMNKRRIYL"

intron 41858..43570

/gene="atp6"

/note="Group IC2"

/number=3

intron 43622..46052

/gene="atp6"

/note="Group IC2"

/number=4

gene 44812..45183

/gene="orf123"

CDS 44812..45183

/gene="orf123"

/note="GIY"

/codon_start=1

/transl_table=4

/product="hypothetical protein"

/translation="MDLFKPTYNLNSTAGSRLGSSHSETTKEKMSNLVKGRKLTEQTK

NLLSLANKGINNANFGKRHSAETKALISLARLGKSFLSESIKDKMSAERGMSIKVLDL

DTSLVSVYTSITRAAKAIGVS"

gene 45343..45852

/gene="orf169"

CDS 45343..45852

/gene="orf169"

/note="GIY"

/codon_start=1

/transl_table=4

/product="hypothetical protein"

/translation="MPIYTALLKYGYKNFRLTILEYCEKDNLILREKHFFEVYSPEYN

ILKTPGSPDRGSGWKHSEATIEIMSIAAKKRNESSDYLTKLSLAQSNSTEIEVFDLKT

NTTTKYHVIRAAARALNIDKRYIEQYIYLNQDGDVVAPVLDRYTFKLLSFKEKSGWCN

LPKIYKKLL"

gene 46307..46480

/gene="rnpB"

gene 46544..50543

/gene="rns"

rRNA join(46544..47393,49908..50543)

/gene="rns"

/product="small subunit ribosomal RNA"

intron 47394..49907

/gene="rns"

/note="Group ID"

/number=1

gene 48234..48974

/gene="orf246"

CDS 48234..48974

/gene="orf246"

/note="LAGLIDADG"

/codon_start=1

/transl_table=4

/product="hypothetical protein"

/translation="-

IRKMKKQLHRTNTNLYTPRFFNFKLSSLAQNNVISLYTDLLIFQQWFVGFSDAEACFI

IHRILDKNGNIGKFSFMFSIELHIDDLHVLEFIKDKLGLGNIRTFKDKCIFTVSDKQG

IYDLITIFDKFNLNTTKYLDYLDFKKAFLLYHERTGKIKISESKELVDSVLELKNNMN

KNRKVLSWEDFEKNINITKYWLLGFIEGDGSFFISRTGIEPTFSIELSDDQYLVLLKI

KEFTVKPPFGIGR"

gene 49120..49596

/gene="orf158"

CDS 49120..49596

/gene="orf158"

/codon_start=1

/transl_table=4

/product="hypothetical protein"

/translation="MSSPEMVFISKKGKDFLDFKIICGAVYKGSHKLDEIRSLILKLS

YNMNNYRLSTNIKPVVVVSEEEKNIIINAESVFDYLEDGRIINKLTNMPIPSVRNSVY

EITLPNGEVILVDTLKETLNKVNVSFKKLKNLLDMEQSVKLQSFEVKRIPIFINHN"

gene 50585..50670

/gene="trnY(gta)"

tRNA 50585..50670

/gene="trnY(gta)"

/product="tRNA-Tyr"

gene 50998..51069

/gene="trnN(gtt)"

tRNA 50998..51069

/gene="trnN(gtt)"

/product="tRNA-Asn"

gene 51324..61308

/gene="cox3"

CDS join(51324..51650,54560..54654,56322..56443,58070..58159,

61139..61308)

/gene="cox3"

/codon_start=1

/transl_table=4

/product="cytochrome c oxidase subunit 3"

/translation="MTHRSNFQNHPFHLVSPSPWPLFTSVSLLSLTTSGTLAMHNFGS

AYILVYFSLFSLVATMSLWFRDIISEATFLGDHTLAVQYGINLGVILFIVSEALFFVA

IFWAFFHSALTPTVELGAQWPPLGIEPVNPFELPLLNTVLLLSSGATITYAHHAFIDG

NRAGGIYGSVLTVVLAGIFTCFQGVEYGVSSFTISDGVYGACFFLSTGFHGLHVIVGT

IFLSIALWRVYAYHLTDNHHLGLEAGILYWHFVDVVWLFLYISIYYWGS"

intron 51651..54559

/gene="cox3"

/note="Group IC2"

/number=1

gene 51651..52997

/gene="orf448"

CDS 51651..52997

/gene="orf448"

/note="LAGLIDADG"

/codon_start=1

/transl_table=4

/product="hypothetical protein"

/translation="-

RKALWIRTKFRGSPKALITKLFIEKFIVASLMIEGIVTSLEIIDLGQWMDNRGSKSDS

PISVKEQRADGSSIVSNYCKVCSESQGNLVFIHPKRDVNTVILTIPFRIRSRSTKNIV

YSNIIQKSLFSSNAYIPQRETFNNSALTLNPNYVTGFTDGEGCFFIGGFHREDVKFKT

GYRIKATFQIGLHEKDMALLEQIKLFFGVGKITRLGTESVQYRVSALDDLNTIIHHFD

NYPLLTRKHSDYLLFKEVLNLMKEGKHLTLEGLNKIVSIKSTLNNGVLSNSLTLAGFA

ANLEPVLIPEVPSRDMQDLHWLAGFTEAEGCFFIALKKSPASKQGETVWLRFILTQHI

RDKELLQSLIQTLNCGRYITKPDCGEFIVEKFTDVRDKIIPIFEEFKLHGAKSLNFED

FKKAALLIGNKAHLTREGLDEIKKIKGRMNKQRKSVLRKDV"

gene 54098..54436

/gene="orf112"

/note="copy 2"

CDS 54098..54436

/gene="orf112"

/note="LAGLIDADG"

/codon_start=1

/transl_table=4

/product="hypothetical protein"

/translation="MNSKRLSTKVEGDITSREILLAKLNNLLSQESNFENKDGKIFIK

STGTYYYNNTKSLTIKLIDKNESVIKTWSTLTSCAKELELSKSGIQKRLKNQTRFNYN

DQIVYLLKDN"

intron 54655..56321

/gene="cox3"

/note="Group ID"

/number=2

gene 54656..56014

/gene="orf452"

CDS 54656..56014

/gene="orf452"

/note="LAGLIDADG"

/codon_start=1

/transl_table=4

/product="hypothetical protein"

/translation="-

FNTIDIIYINIAVWVQISLYTFIIHNNFNSYFIFSHYPHSIDRLSPKSKRYFSTSNNI

DVEFYKWFSGFTDAEGSFMITPLAKGFSFRFSIGLHIDDLNVLNYIKDKLGFGKVYSS

DNTCYFNVTKKEDILKLINIFDAYLLNSTKRFNFWDFKKAYFLYCDRDLLTPELTNQI

LDIKNNMNNLRENFHNLQEFNISKEWLLGFIEGDGSFSLSRSTMEPIFSIKLSETELS

LLYAIKEYLINNLGLDMYSVNKLEGSSVISIGKGKAVNNSKPLATLTIKNIHFNNNVF

IPFFDESQFRTKKGLDFKDFKLLCHIIYIGGYRTERIKNLLIKLSMTMNNFRLSNYIG

EKVSISPTDLKDILASEETIQHLSDGRELDINTKKLIHRRSSSSVFEILKPSGEILIK

QNLAESAKEIGVGFNTLKKQLNNELSEVEYKGYKIKRIGVFKNKM"

intron 56444..58069

/gene="cox3"

/note="Group IA"

/number=3

gene 56446..57555

/gene="orf369"

CDS 56446..57555

/gene="orf369"

/note="LAGLIDADG"

/codon_start=1

/transl_table=4

/product="hypothetical protein"

/translation="-

LDLTFFVLVVIYYTVYGASSSLLSYYTNYLVDHKSLVNRSISNSHDKTLYSSSAAHKG

KRFYTTDVKDSTLPFYWVTGFIDAEGSFSLKVSKSSSTSSGYNVIPEFKIELHNRDLL

LLRKIHAFFGVGIISEYESNNKASYSVQSAKDLVNVIIPHLDKYPLLTKKKEDYLLFK

EALIFLLTGKARSSYEGISKILSLKGSMNAGLSDKLKISFPNLKCVPRPVINTQEIPD

LNWLAGFVDGEGYFYIKSLKNPRYSTGYSVTLVFSLTQHARDEALLTKFIDLLGCGNI

EKAFTRPYEVNYRVSKFNDIKEKIIPLFNKYPLQGIKFRDFLDFSEAAVIVNNKDHLT

VEGIKKINSLKSGMNRNRLI"

intron 58160..61138

/gene="cox3"

/note="Group IA"

/number=4

gene 58162..59187

/gene="orf341"

CDS 58162..59187

/gene="orf341"

/note="LAGLIDADG"

/codon_start=1

/transl_table=4

/product="hypothetical protein"

/translation="-

ILNFNYNTNIHSIHTLKSKAFALKNFNGKRFYSTIKVNDVELCPNWVTGFADAESSFS

LKISEKSTAKSGWNVIPEFRIELHNKDIILLRKIHSFFGVGIISERLDRNAVVYSVQS

VRDILNVIIPHFDKYPLITQKRADYLLFKQAINLLDLKVHLNIEGFYQIIAIKASMNL

GLSDKLKNSFSTVYPVGRPFVNFEGDFHPNWLTGFTDGEGCFYVNTKKAKTSTGYQII

MSFSITQHVRDENLLNKIADYLDCGNIEKVSTRSTTVTLVVYKFFNIKEKIIPFFQKY

PLQGVKSLDFNDFCKIANIMITKEHLTLEGVKKIKSLKSGMNSSRIFFNV"

gene 60022..60870

/gene="orf282"

CDS 60022..60870

/gene="orf282"

/note="LAGLIDADG"

/codon_start=1

/transl_table=4

/product="hypothetical protein"

/translation="MLIKNKEHLTDEGKLAIINYKKEMQSMSGKWVPESIYNKINITK

YWLAGFIDGEGTFSTNKYVPRFKLENHIKESELYHKIKEFMGTDKLIYSSDRSDKRLE

SNPTIILEINKVKYIKEILIPLMYDSNNKLLLKTLKSKNFLLWLNLVDIYYLGYHTIL

EGKQLFDIIKNNINKYSLTTNDSLKRGLKTRAVYSAPEIKILLSELYLLDSPYEVKDG

VRYYRGTNNLVSDSNQIISIDDNNSRVVHNSMSEAANSLNISRKTIKECLTTGKSYKG

ITFIFK"

gene 61266..62423

/gene="orf385"

CDS 61266..62423

/gene="orf385"

/codon_start=1

/transl_table=4

/product="hypothetical protein"

/translation="MAISLHFYILLGFIVPLCDLLLIFNVVLIKLDIVIYCIYSKIYN

IFLTIKSNKLKFVFVLLCAYGITHSLNLIDCYSLFKNFYLLSTFFMVLYAFIFYSFKD

NCSLIKSSLLFILKLNTIIIIASYSISLLFDLCFLNSFFQTRNIVGIFILCKLCIGIV

NMEWTTSNFKGLLNKLSFINVISFLTLSLLFGLGTGIVFMNTGSNEPGNFNNSSGPGG

GNGGGPQPPHNSWSDILYPDHYHDKNIDKELLKKNPSEKNEDYYKRLEGIKEHHDLGL

KKLLDNSHNNIYKSYDFYNEKYNKLIYLEKIRNMPEKAAEYEHCVAKWTKTLNGKTVE

VKSNLDIETLKETSGYYEEVLNKQKQINKECQTFHRSILGRMNNIRKKMNN"

gene 62649..62720

/gene="trnK(ttt)"

tRNA 62649..62720

/gene="trnK(ttt)"

/product="tRNA-Lys"

gene 62749..62821

/gene="trnD(gtc)"

tRNA 62749..62821

/gene="trnD(gtc)"

/product="tRNA-Asp"

gene 63315..63395

/gene="trnS(gct)"

tRNA 63315..63395

/gene="trnS(gct)"

/product="tRNA-Ser"

gene 63398..63468

/gene="trnW(tca)"

tRNA 63398..63468

/gene="trnW(tca)"

/product="tRNA-Trp"

gene 63544..66086

/gene="nad6"

CDS join(63544..63776,65615..66086)

/gene="nad6"

/codon_start=1

/transl_table=4

/product="NADH dehydrogenase subunit 6"

/translation="MNNLFIISENFTNGYKSEVLDIISVLVILSGIFVIISKNPVISL

LFLIGLFGGIASYLLIVGLSFLGISYLVVYIGAVSILFIFILMLINIRMSELQSHTSN

SIPLAISIAILFNYPLFQLLPYDIAILNNSNNFLNNLLYNVSLNKINDVSATPETNID

INDPLFVTSKMWDGNLTEFSHISSIGNIMYTSYNMWLLITGFILLLAMVGTIRIVIKD

RSMDTNAHTGHSGTHR"

intron 63777..65614

/gene="nad6"

/note="Group ID"

/number=1

gene 63778..65262

/gene="orf494"

CDS 63778..65262

/gene="orf494"

/note="LAGLIDADG"

/codon_start=1

/transl_table=4

/product="hypothetical protein"

/translation="-

NRIIRLLSSNGAAWVKISLYMVILIINKNLFLLCETWNKFGGLQYTAKRPDSKYITVN

LIPSHKLNSLCTRSNKTFFSTLSINTTSFYQWFVGFSDAESNFNIVIYKDSTGHITSV

TFRFIIELHIDDLDALYYIKDQLNIGNKIATYGNSCKFTVTHPNDIYKLIEIFDKYSL

NTTKYLDYLDFKKAFILYRERDKTIDNKEALISQILEIKSGMNSNRTNFNYPTEHKIA

ISDYWLLGLIEGDGSFYLDRKEMEPIISIARRRSRRESQSSLLENIKIYLENNLGFDK

YSKFKLDNSSVISILEGKAKNNSKALSVLRVKNVNILNNYLIPYLDKMQFITKKGKDF

EDFKLICQVIYKGAYRVDEIKSLILNLSYTMNNYRLSTNLESEKVNNFSNEDREKLIN

AKFTIKHLKDGRQLDIITSKSINRRWTNCVFEIIKENGDILLASTLKDAAEILSLDFR

TVKRYLESESLNGEYVLISSCKVRRVAVF"

gene 66392..66964

/gene="orf190"

CDS 66392..66964

/gene="orf190"

/note="GIY"

/codon_start=1

/transl_table=4

/product="hypothetical protein"

/translation="-

ACSADFILRGFHQPLYKDPYFIEKLGKVNSSEVKGVYIFTHKTTGDKYVGSSTQLAVR

LQRYLAGTYKEYGKFVPFLRKEGIEKFRLEVMPIYSSVTFKSELILEQFFLLDPSFNL

NTSRIVNVPKYNSREIFMYNKEKTLLIYSSIYMKDFLVKLGINNSLDSDSDNESEAYS

VYTVILDPVDNTSKN"

gene 67144..67464

/gene="orf106"

CDS 67144..67464

/gene="orf106"

/codon_start=1

/transl_table=4

/product="hypothetical protein"

/translation="MLNQQYLLQQEQKHYDSLISNLNEKKIDQDLVQKEVDELLVNVH

KLKNNVSVNSNNETSMLIEAENTSLSNKLLQDKDTKIKESEDKSSPSEDPENNDLLDE

KKNK"

gene 67551..67622

/gene="trnV(tac)"

tRNA 67551..67622

/gene="trnV(tac)"

/product="tRNA-Val"

gene 67634..67704

/gene="trnI(gat)"

tRNA 67634..67704

/gene="trnI(gat)"

/product="tRNA-Ile"

gene 67709..67795

/gene="trnS(tga)"

tRNA 67709..67795

/gene="trnS(tga)"

/product="tRNA-Ser"

gene 67870..67942

/gene="trnP(tgg)"

tRNA 67870..67942

/gene="trnP(tgg)"

/product="tRNA-Pro"

gene 67942..81140

/gene="rnl"

rRNA join(67942..68367,69190..69324,71211..71475,72970..73590,

74936..75261,77438..78005,80599..81140)

/gene="rnl"

/product="large subunit ribosomal RNA"

intron 68368..69189

/gene="rnl"

/note="Group II"

/number=1

intron 69325..71210

/gene="rnl"

/note="Group IB"

/number=2

gene 70263..70568

/gene="orf101"

/note="copy 1"

CDS 70263..70568

/gene="orf101"

/note="GIY"

/codon_start=1

/transl_table=4

/product="hypothetical protein"

/translation="-

IHNLRLRMWEHNLLAPPRLYSVTQPWVNKINGNTYVGSSINISVRMYTYFSLGSLAKS

NRPIDRALLKHGFSNFTLEILEYTTLRGDVVSSSGLVIKIMF"

intron 71476..72969

/gene="rnl"

/note="Group IC1"

/number=3

intron 73591..74935

/gene="rnl"

/note="Group IA"

/number=4

gene 73877..74803

/gene="orf308"

/note="copy 2"

CDS 73877..74803

/gene="orf308"

/note="LAGLIDADG"

/codon_start=1

/transl_table=4

/product="hypothetical protein"

/translation="-

MKNIKFINLKFTEWLVGFIDAEGCFHIKRKVMSNGNIRYYFEFVIKLHIDNVPLLKFI

YETLDIGRIAVRPNSNSCTFEIGSEKDLRIFIGLLDKLPLMGSKYLDFLSFRKAFFLY

FDRPGLVTDDIIDAIENIRNNHNTRRTNFELPLDFKCIITDYKLLGLIEGDGSFIVRA

QDLTPKFEIELTAVQKFLLEAIREFLINYFLQLGLSIDQLEKAIKLSDLKAKGNSKAT

VRLTISGINFLHNYFNVYLKRLHFYSDKSTDFETFCTICESLFNKLHINNEKVKNDLL

NLAKGMNNARYSTFKDK"

intron 75262..77437

/gene="rnl"

/note="Group ID"

/number=5

gene 75931..76323

/gene="orf130"

CDS 75931..76323

/gene="orf130"

/note="LAGLIDADG"

/codon_start=1

/transl_table=4

/product="hypothetical protein"

/translation="-

SSFTSLGGEDMFDLQAWFVGFSDAESNFSIVPKEDSKGNVNRFTFVFGIGLHIDDVEA

LEYIQSILNIGIVRTYKDECKFIVTKREDINKLISIFDTYNLNTSKYLDYLDFKKAFN

LYERRIFNRGIKK"

gene 76286..77176

/gene="orf296"

CDS 76286..77176

/gene="orf296"

/codon_start=1

/transl_table=4

/product="hypothetical protein"

/translation="MKEEFLTEELKNKILELKEGMNTKRKNFNMPNEHKIVITKSWLL

GLIEGEGSFQLWRKDLVAVFSLVLTEKQLPVLEEIKKFLVNNLGFDKYSKFKLEHSSA

IAINHQKARNNSKGSVLFIIKNIHVLNNYLIPYLENELFITKKGKDFKDFKLICKALY

IGAHKINDIKSLILKLSLTMNNYRLSTNPESTELLSSLERDTLANVSPCIAHLSDGRQ

RDLVTGKIIHQHSSSIYEIIKPNNELSLKQTLFEAADIAEVNIKTLSKLLDLSDNGEI

EIKGNIIKRIPVFYKDKGKI"

intron 78006..80598

/gene="rnl"

/note="Group IA"

/number=6

gene 78202..80391

/gene="orf729"

CDS 78202..80391

/gene="orf729"

/note="LAGLIDADG"

/codon_start=1

/transl_table=4

/product="hypothetical protein"

/translation="-

INKMQKYTKFLSNIFVKNINNKYKSIPLNTRINFVGETRYFPSDFKEWTNSVYYFNSN

NIKNFPIYDLNVSKLLKGYFDLYFDRENIKLNYKSYEKKDFTAQALNKIFVSKPEIKH

TNSKAIITIYVYNRERVILLNKLNQLNIGILGLNKFFYLCKKISGDLYSKYIKQVLYK

EFLFLRRSKLKLNLNGLKFQDKFLFKLSKLISKFYKKKVEFNIINLKSINLNPNIFTE

IMAKKFMNRNASIMQIMKFILDKSIILNEDGGIVSLASSGVTSSGSGLEKSRKIKNVN

LNLIENKYKNLNINSIVTDGDINDNIKEFYTKNSEDTIFDSIKYKNLGGIRLEAKGRL

TRRYRADRAVSKVNIKGGLKNIDSSYKGLSSINYIGKINSSMEYSMDISKRRVGAFAI

KGWISGRSYSTTANPTRNESINPWVLTGFADAEGSFILRIRNNNKSSAGYSTELGFQI

TLHKKDISILENIQSTWKVGVIANSGDNAVSLKVTRFEDLRVVLNHFEKYPLITQKLG

DYLLFKQAFSVMENKEHLKIEGIKRLVGIKANLNWGLTDELKEAFVASGGENIFVASG

GERSLINKNIPNSGWLAGFTSGEGCFFVSLIKSKSKLGVQVQLVFSITQHARDRALMD

NLVTYLGCGYIKEKKKSEFSWLEFVVTKFSDIKDKIIPVFQVNNIIGVKLEDFEDWCK

VAKLIEEKKHLTESGLEEIRNIKLNMNKGRVL"

gene 81158..81228

/gene="trnT(tgt)"

tRNA 81158..81228

/gene="trnT(tgt)"

/product="tRNA-Thr"

gene 81615..81920

/gene="orf101"

/note="copy 2"

CDS 81615..81920

/gene="orf101"

/note="LAGLIDADG"

/codon_start=1

/transl_table=4

/product="hypothetical protein"

/translation="-

FKTGWKVLAFFELKLHRRDLELLKYIQAELGGIGRIVPNGEKAYSLRVNILNELLDVI

IPHFNKYPLVTQKLADFMLWKEVVNKMKNKEHGDVVALVKDF"

gene 81884..82459

/gene="orf191"

CDS 81884..82459

/gene="orf191"

/note="LAGLIDADG"

/codon_start=1

/transl_table=4

/product="hypothetical protein"

/translation="-

RRRSRPREGFLDIVSIRASINTGLSDVLNNEFSKIIPVLRPLIEKQQIPHEEWLAGFI

SGEGSLFVRIAKSRTNIGSRVQLVFTITQHTRDIKLLESFIGYLKCGTVRAYNNRNLC

DFVCIKLKDITDIIIPFLNKHPILGVKSEDFKDWCQIAEEMKLGNHLTSVTGGCGLDR

IIKIKTNMNRGRLLNL"

gene 82495..82567

/gene="trnE(ttc)"

tRNA 82495..82567

/gene="trnE(ttc)"

/product="tRNA-Glu"

gene 82675..82747

/gene="trnM(cat)"

/note="copy 1"

tRNA 82675..82747

/gene="trnM(cat)"

/product="tRNA-Met"

gene 82751..82821

/gene="trnM(cat)"

/note="copy 2"

tRNA 82751..82821

/gene="trnM(cat)"

/product="tRNA-Met"

gene 82943..83024

/gene="trnL(taa)"

tRNA 82943..83024

/gene="trnL(taa)"

/product="tRNA-Leu"

gene 83286..83561

/gene="orf91"

CDS 83286..83561

/gene="orf91"

/note="GIY"

/codon_start=1

/transl_table=4

/product="hypothetical protein"

/translation="-

TTTTTSSSSSSWTNLITNKAYIGSSINLASRFKQYYSTKFLEYEIIKNNSKIYRSLIK

NGYSNFKLEILEYTTLRGDVVSSSGLVKGMYF"

gene 84338..84408

/gene="trnG(tcc)"

tRNA 84338..84408

/gene="trnG(tcc)"

/product="tRNA-Gly"

gene 84411..84482

/gene="trnA(tgc)"

tRNA 84411..84482

/gene="trnA(tgc)"

/product="tRNA-Ala"

gene 84655..85128

/gene="orf157"

CDS 84655..85128

/gene="orf157"

/note="GIY"

/codon_start=1

/transl_table=4

/product="hypothetical protein"

/translation="-

FLYNLTHIIYYNLLNLNLKHTMTKYEKIYTNILEQKALINKECAGLSGIYCWRNMENG

KLYVGSAVDLKNRFSIYFSEVSLNRNIKKGTSLIYSSILKHGYSKFYLYILEYCDPKN

WISREQYYIDKFDPEYNILKVAGSRLGSKQTTKTKELIRN"

gene 85531..85603

/gene="trnF(gaa)"

tRNA 85531..85603

/gene="trnF(gaa)"

/product="tRNA-Phe"

gene 85751..85834

/gene="trnL(tag)"

tRNA 85751..85834

/gene="trnL(tag)"

/product="tRNA-Leu"

gene 85896..85968

/gene="trnQ(ttg)"

tRNA 85896..85968

/gene="trnQ(ttg)"

/product="tRNA-Gln"

gene 86291..86755

/gene="orf154"

CDS 86291..86755

/gene="orf154"

/note="LAGLIDADG"

/codon_start=1

/transl_table=4

/product="hypothetical protein"

/translation="-

FYWAIKPLNFKWVYRYCAPRGCFTVNIGYNKTHKKGYQIRPMFTINLNKKDIAILEQI

KIYFCGYFIGDYWFHQPGVITLSGKDCFKYRIADLNMLIYKVLPHFYKYPLITQKRAD

YLLFKKIVLLMKDKKHGDVVALLKDLMKLFLLKHLLI"

gene 86847..87095

/gene="orf82"

CDS 86847..87095

/gene="orf82"

/note="LAGLIDADG"

/codon_start=1

/transl_table=4

/product="hypothetical protein"

/translation="MIRICSRWWLYRSALKLIFLRVQVVYRSALNKRVILRFRITQHS

RDTELMTNLITYLDCGKINETKDARISSCADFISHDIK"

gene 87038..87283

/gene="orf81"

CDS 87038..87283

/gene="orf81"

/note="LAGLIDADG"

/codon_start=1

/transl_table=4

/product="hypothetical protein"

/translation="-

KRCADFILRGFHQPWYQVTKILDINEKIIPFFNKYPIIGVKSKDYSCFKEVAKLIELK

KHLTEEGFKEIEQIKSKLTVNN"

gene 87479..87552

/gene="trnH(gtg)"

tRNA 87479..87552

/gene="trnH(gtg)"

/product="tRNA-His"

gene 87887..87959

/gene="trnM(cat)"

/note="copy 3"

tRNA 87887..87959

/gene="trnM(cat)"

/product="tRNA-Met"

gene 87997..102827

/gene="nad2"

CDS join(87997..88374,91105..91296,93672..93842,95168..95185,

96551..96778,99187..99384,100776..101219,102795..102827)

/gene="nad2"

/codon_start=1

/transl_table=4

/product="NADH dehydrogenase subunit 2"

/translation="MLLISILSLLLSNAVTIRRDISILFNRVAIIALAYSILHSAVSL

FIASKGIGLHGGLLNITSITQIFDIFIFLVSILILQLTSFFPRKVWVPEHSSLMQLFF

NKFVFYRTKIINKMGEHLTIIEYPLILLFIISGAVFLISTSDLISVFLSIELQSYGLY

LLSTIYRNSELSTTGGLMYFLLGGLSSCFILLGTSLLYANSGTTNLDALYAITSISDA

SDLWYKPYYINFALLIFSIGFLFKVSAAPFHFWSPDVYDAIPTIVTTFVAIIAKISIF

VFLLEIVYYTRNYFSDFNWTYGLLISSLFSLIIGTVVGLTQFRIKRLFAYSTISHVGF

ILLALSISSVESTQAFIFYLMQYSISNLNVFIILIAIGFSLYNYISENKEYKELLDKN

NSPIQLISQLRGYFYINPTLSLSLVITIFSFAGIPPLVGFFAKQMVLSAAIDNGYIFL

SLIAILTSVISAVYYLNVIKEIFFYSPEYKLNPLLENLNFNGNIYNSNNVLIKSVTFK

HNNISISSPFTITISVITLIILLFIFVNKEWLSMSTILVQILFNY"

intron 88375..91104

/gene="nad2"

/note="Group IC2"

/number=1

gene 88375..89307

/gene="orf310"

CDS 88375..89307

/gene="orf310"

/note="LAGLIDADG"

/codon_start=1

/transl_table=4

/product="hypothetical protein"

/translation="-

RKGSKDLNLNSLYFTRRITSFNVLFCTNTTQYTRFCNSKSKLFNLCFSSSAAISTKSS

VAIEDTNLNPWALTGFIDGDGSFSILVTKATNGKLMCKVQPVFTIGLHIKDLSVLHLI

KKYFKDAGNIYIRKDDKVVYYNVSSVKEIIKYIIPHFYSFPLVTQKQADYLLFKEIVS

RMNNKEHLTNNGLIEILSLKSSLNLGLKGWVADLMKDIEAAPRPEVQELDSLQPDWLS

GFISAEGCFSVVLSINNKLKTGYRAGIRFILTQSDRDILLINQIRDFFGSGRVVPSNR

SNTVELWITDFEAIKLCPS"

gene 89455..90888

/gene="orf477"

CDS 89455..90888

/gene="orf477"

/note="LAGLIDADG"

/codon_start=1

/transl_table=4

/product="hypothetical protein"

/translation="MMKNKKNFRIVNIMNLGLFRSTEQLISKILRDELSNSGELLKLL

IPNLENIFRGGWTNYSGMVTSQKMVLLFYLIKYNIYGMCFPGTLEIKTRKMDNRGSKS

IISKDIVVKEQRVDGSWLLNFTTFNSLRCTLRRFERITLSGSISYQILNKRLYSTKTT

IMATKDPKNIKSFIQEQALDGFSLNPWFVTGFTDGDGSFAVSITKKKEGIGWKIVPMF

TIGLDQKDLDLLVQIKDFFKTGNIYTSKRGVTYYTVGSVKDIVKYILPHFDKFPLVTQ

KLKDFILFKEIVLIMEKGEHNTLPGLLKVFSLKVNLNKGLSTIVKEEFPDIKPTVLGA

SSEFKVPLSFNPNWIAGFITAEGSFFISLYEKEKRKAGYAVSLSFSLSQHLKDIKLLE

RLAIFLDCGVVKIPSNREAAELIITKSSDLNQKLIPLLKKYNLSGVKLLDFERFREVS

LLIENKMHLTSEGIVLIKTIKDAMYNR"

intron 91297..93671

/gene="nad2"

/note="Group II"

/number=2

gene 91420..93540

/gene="orf706"

CDS 91420..93540

/gene="orf706"

/codon_start=1

/transl_table=4

/product="hypothetical protein"

/translation="-

GNGQNQISLRAHRAESPMRRAILPKFHSLGALTWGLNHFWDRIYELCKTLKWRVLQGN

TFNQDTISILNVKSGVSKGDVKITRRTGGLPTALKGHGNRGVVVLLAGRAPASGVCMM

STFADSISIVSTNGLAKIQKINELCSENKNFVVTDKLYNIMYNKDVFFAAYHKLKSKP

GNMTPGIVPTTLDGMSDEEVMKIISSLRDGSFKFNPGRRVYIPKNNGGERPLTIAPPR

DKLVQEVMRMILEAIYEPAFLNCSHGFRPKKSCHTALRDVRQRLGMAKWFIEGDISKC

FDSVDHNILMGIISSRVKDQRFLDLIRKALKAGYMEARVYSHSLAGTPQGSIISPILA

NIYLDKLDNFIMELKTRFDKGGKATINPEYKRLSSRKDRAKDTLVKRMINLVLLKTSS

KLHIDPNFKKLEYVRYADDWIIGIRGSKRDCEILITEIRDFLDKTLNLRLSEEKTKIT

NVSKDLATFLSVGIQRKTHRTLRRMEQGHSRRNVNNLRLLAPVRKITSKLAENGFMKN

GDPYPRFKWMGEEKDAIILLYNSVYRGIINYYRFVDNFNILSSKVHFILKNSCARLLA

AKYKSTQAKIYKEYGKNMKGDNKHGFIDITLGIKLAAFSPKTDDVLFKFNAEGISRTS

LEDLKCSVCKSDYRVEMHHIRMMKDLDPKKSLVDRLMIKRRRKQIPLCRNCHMELHNK

DNQTKNKQN"

intron 93843..95167

/gene="nad2"

/note="Group IC2"

/number=3

gene 93843..95111

/gene="orf422"

/note="copy 1"

CDS 93843..95111

/gene="orf422"

/note="LAGLIDADG"

/codon_start=1

/transl_table=4

/product="hypothetical protein"

/translation="-

LWFRESHFWVYLSNSGNTLKITVPSYSWKAIYGWANYLCTVIIRNIIERLMGNRGSKS

VIFGTFSTNAVKEQRVYGRFRDDLRVLRRRCTLTGFERNYQIKTLSNHINILTKSYSV

LASAGQIFNNLSLNPWFISGFTDAEGCFSINIRKVSKYKIGWRVSVEFRIKLHEQDVA

LLELLKNYFGFGFITKEMKSVVYRVSALEDILKLISHFDQYSMITHKQIDYLLFREVV

KMMQLKEHLTQEGLEKIVSLRAQLNWGLSDELKAAFPNYNQNTRPKLDSQIIPHPEWV

AGFASGEGCFHVRITESPSLNCGYSVLLVFVIGQHIREELLMKSFSSYWKCGKYYNRG

SSLGVFNCGKFLDIYTKIIPFFREHLILGKKSSDFEKWCQIAEIIKDKDHLTIAGVDR

IRAIQSDMNKPKFQG"

intron 95186..96550

/gene="nad2"

/note="Group IC2"

/number=4

gene 95186..96460

/gene="orf424"

CDS 95186..96460

/gene="orf424"

/note="LAGLIDADG"

/codon_start=1

/transl_table=4

/product="hypothetical protein"

/translation="-

RGLGKSSTVGSKLPNSGNTLELQVPSDIRKDIRGWTNYSGMVTSLKASEKNVGNRGSK

SVFNTYNTVKEQRVNGSWCSTSLLHLRCTLMGFERNYQIKIPSNQIIQRRLYSIESNL

NNNSNKLIQQLEPWFVSGFADAEGCFLVLIRKSPKNKLGWQLETNFTINLHARDLDLL

KQIQIYFGGVGRIGKERNGCCDFVIGSLDQIVTKVIPHFDKYPLKTNKYSDYILFKEV

VSMMQGGEHLTAEGLQKIVNIRASLNKGLSSSLIESFPNNTPVLRPPLPISNTNQLHP

QWVAGFTSGDGSFKVSVRESKLYKAGSRVALVFVLTQHIRDELLLKSLIDFFKCGHIY

SYKDYVEFRCQSFIDIYEKILPFFFKYPILGVKAQDFEDWTKIAKMIQTKVHLTSEGF

DEIRKIKIGMNKGRYLK"

intron 96779..99186

/gene="nad2"

/note="Group II"

/number=5

gene 96779..99097

/gene="orf772"

CDS 96779..99097

/gene="orf772"

/codon_start=1

/transl_table=4

/product="hypothetical protein"

/translation="MRRCALFIYVTLGHMHCSNMHPTSELGVGEIPTLNLASPPKRDI

RLNQYSWLGFWVAFSKIKTKLFEVQLQGSSTIGNYQVWDRVARLIKILKWCILIWTDL

FLGIINTYIAMSGQSKENYEGISNRTAGLPKAKNGYGNRGIVVPLTLLTMSSIGPKGP

VAMGRIPGLWCCSYSSTAGDSSTVKSDAIRKLQWLEGKCLDNQNFIVSDIYSLMYNKN

IYEIAYDKLKSKPGNMTPGFNPTTLYGFSSEVIQEIILSMKNDTFKFKPGRRVQIPKA

SGIGLRPLTIAPPRDKIVQEVMRMILEVIYEPSFSPNSHGFRANKSCHSALKQIFTTF

GVATWYIEGDISKCFDSFDHDILIGIIRKRVKDERFIRLITKALKAGYFEFNEFKHSI

VGTPQGSIISPILCNIYMDGLDKFIEDLIRSFSKGSRPRGNPIWISYSNKKTRAKSLT

DKIKWHKLMLTVPSKDPLDPSFKKLVYVRYADDWILGVRGSREECVEILNCIRVYLKE

ELKLNLSEEKTLITNANKEKALFLGTQIYRSRHQSYSRSLGFTKRVGKEIRLMAPLER

ITKKLKEANFIVNGVSAPRFLWLHNSKDQIVALYNSVYRGFMNYYSFAHNFGNISGHT

HNILLSSCAKLLAAKFNSDSQKKIFEVYGKNLKGNDKIAFVEPTYKIKVWDFKTPANV

EDSEISKNGLIPTLYADKISVASLDNLKCSVCNSDYRVEMHHVRMLKDLNPTISKIDQ

LMVKSRRKQIPLCRKCHMEHHSPKSKLSKPSS"

intron 99385..100775

/gene="nad2"

/note="Group IC2"

/number=6

gene 99385..100680

/gene="orf431"

/note="copy 1"

CDS 99385..100680

/gene="orf431"

/note="LAGLIDADG"

/codon_start=1

/transl_table=4

/product="hypothetical protein"

/translation="-

LRFGEPLLRDLWPNSRELLKLKVPNCSLKTISGWTNHSCMVTSLSTLKVDLVFWLNKI

IERAMEYRGSKSVLINNTVKEQRVDGHRCEKLNFSHLRYALMGFERSYQIRTPSNHLI

LYKNYSTSSNTDQSIYNLNPWFITGFSDGESNFTIRITKSNSILGWNVQPVFQIGLHK

KDLNLLKKIKASFGVGEIYFKEKSCNFIVQSLNGVKVIINHFEKYPLFTKKQEDFLLF

TQIVALISQKEHLTLEGLQKIISLKASMNLGLPESLKTAFPEVLPAIRPKRLDEELLN

SKLDPNWVVGFTAAEGCFSIRVAKSLTTKTGYQVQLRYQITQHSIDKIFMNSLVKFWG

CGKVFLRYRENKVDFQILKIKDLSDKVVPLFNNISLESEKSKDFLDFCKAIDIMKRKD

HVTNKGLSEIRRLKEGMNTGRNWE"

intron 101220..102794

/gene="nad2"

/note="Group IA"

/number=7

gene 102828..103313

/gene="nad3"

CDS 102828..103313

/gene="nad3"

/EC_number="7.1.1.2"

/codon_start=1

/transl_table=4

/product="NADH dehydrogenase subunit 3"

/translation="MSSMSIFFVVVIIIALLFIVINLIFAPHNPYQEKYSIFECGFHS

FSQSRQPFDISFFIYALLFLLFDLEILLLFPYSVSSHTNDIYGLVVMLGFTTIVTIGF

VYEIGKGALKISSRQNNPTVKKHNVKIISLGESFGQRRNYSSLTPNYFPAKVYENAQK

W"

gene 103267..103509

/gene="orf80"

CDS 103267..103509

/gene="orf80"

/note="GIY"

/codon_start=1

/transl_table=4

/product="hypothetical protein"

/translation="-

TIFLQKCMKMHKNDKAQILSDNKDKSGIYRWVNLESGKCYVGSSVDLKERLTSYFNIN

HLKENYNMLICRALIKYGGPD"

gene 104147..105657

/gene="atp9"

CDS join(104147..104327,105614..105657)

/gene="atp9"

/codon_start=1

/transl_table=4

/product="ATP synthase F0 subunit c"

/translation="MIQVAKIIGSGLATTGLIGAGVGIGVVFGALILGVARNPSLRGQ

LFAYAILGFAFSEATGLFALMMAFLLLYVA"

intron 104328..105613

/gene="atp9"

/note="Group IA"

/number=1

gene 104824..105126

/gene="orf100"

CDS 104824..105126

/gene="orf100"

/note="GIY"

/codon_start=1

/transl_table=4

/product="hypothetical protein"

/translation="-

TFMFSCKFSSHSSLFNYGLKIYNDPLNQRELIRDENNGKIGIYCWINNINGKYYIGSG

DPLYIRISDYFQNWYILSRTNLYIVRSLSKYGMTNFSFLLY"

gene 105108..105608

/gene="orf166"

CDS 105108..105608

/gene="orf166"

/note="GIY"

/codon_start=1

/transl_table=4

/product="hypothetical protein"

/translation="-

FFIILEYSNSNSVIECEQKWIDLLKPQYNLNPTAGSTKGYKHTTESIEKIQKKALGRK

HTEEVKQVMSKTRQGDNNPFFGKNHNDKSLELLRTVAMNRFKPPVAGIEVEITDLETK

LTHTFESIRKAASFMSSDIKIIWRREELQNTFGINTPYKKRYIIVIKRS"

gene 105912..120274

/gene="cox2"

CDS join(105912..105995,107387..107413,108886..108978,

110327..110353,111493..111582,113557..113790,

115414..115453,118184..118242,120176..120274)

/gene="cox2"

/codon_start=1

/transl_table=4

/product="cytochrome c oxidase subunit 2"

/translation="MNFLLNNLIIKLDAPSAWGLYFQDSATPQMEGLIELHDNIMYYL

VIILFAVGWILLSIIKNYVSTASPISHKYLNHGTLIELIWTITPAVILILIAFPSFKL

LYLMDEVSDPSMSILAEGHQWYWSYQYPDFLDSSDDFIEFDSYIVPESDLEEGALRML

EVDNRVIIPELTHIRFIITSGDVIHSFACPSLGIKTDAYPGRLNQVSVFVNREGVFYG

QCSEICGILHSSMPIVIESVSLEKFLTWLEEQ"

intron 105996..107386

/gene="cox2"

/note="Group IC2"

/number=1

gene 105996..107264

/gene="orf422"

/note="copy 2"

CDS 105996..107264

/gene="orf422"

/note="LAGLIDADG"

/codon_start=1

/transl_table=4

/product="hypothetical protein"

/translation="-

PYCSGKTITLGIKLSNSGNTLKHIILNYIWKYISGWSNYSGKVTSYMISENAMGNRGS

KSVFINNNIVKEQRVNGSWRGINLPCLRYTLMGFERSYQNKILTTQLIRKISYLQINE

HKINNNIKLTIDPNFFSGFADAEGSFVLSITKSNNVSSGWVIKPRFKIHLHKKDLFIL

EAIQNFLGVGRIYKRDINAVEFRVFSIKELGVVLAHFDKFPLLSQKYADFFLFKQAYQ

LIVNKEHLTAKGLRNIVSIIASINNGLSEDLKKAFPDITYIPRPVKENVSIYDPQWLA

GFTSGEGSFGAKIRNADYVGNTKAYVELIFQINQHVRDSLLLNCIVKYLNCGKIYKHS

VKAEVLRVSKTSDLIEKIIPFFVKYPILGIKALDFKDFCSILELNLNKKHQTEEGLNK

IIDIKTNMNTGRKYE"

intron 107414..108885

/gene="cox2"

/note="Group IC2"

/number=2

gene 107858..108760

/gene="orf300"

/note="copy 1"

CDS 107858..108760

/gene="orf300"

/note="LAGLIDADG"

/codon_start=1

/transl_table=4

/product="hypothetical protein"

/translation="MEKFKNKYSGLNPHYVTGFSDGEACFHLAIGKNSKYKIGYYVNP

GFSIVLHKKDEQLLINIQSFFCGIGNLKVKSNIVQYRIFSLEELNILLDHFDKYPLIT

KKLVDYKLFKEALELMKTKEHLTIEGFNKIIALRASMNLGLPETLKEAFPDIKERDII

PNDLPSLLNPYWVAGFTDAEGCYWIKTLKDSTKNKVSTVIGFQITQHSRDLLLLKKLI
[truncated: 1,866,460 more chars]
